# Kinetic resolution of racemic 2-substituted 1,2-dihydroquinolines *via* asymmetric Cu-catalyzed borylation†
†Electronic supplementary information (ESI) available: General experimental procedures. Compound characterization data, analysis of enantioselectivities of products and crystal parameters. CCDC 1542782–1542784. For ESI and crystallographic data in CIF or other electronic format see DOI: 10.1039/c7sc01556a



**DOI:** 10.1039/c7sc01556a

**Published:** 2017-04-19

**Authors:** Duanyang Kong, Suna Han, Rui Wang, Meina Li, Guofu Zi, Guohua Hou

**Affiliations:** a Key Laboratory of Radiopharmaceuticals , College of Chemistry , Beijing Normal University , No. 19 Xinjiekouwai St. , Beijing 100875 , China . Email: ghhou@bnu.edu.cn

## Abstract

A highly efficient kinetic resolution of racemic 2-substituted 1,2-dihydroquinolines *via* asymmetric Cu-catalyzed borylation has been realized for the first time. Under mild conditions, a variety of chiral 3-boryl-1,2,3,4-tetrahydroquinolines containing two vicinal stereogenic centers as well as the recovered 2-substituted 1,2-dihydroquinolines were afforded after 30 minutes in high yields with up to 99% ee (dr > 99 : 1) and over 98% ee values, respectively, corresponding to kinetic selectivity factors of up to 569. Moreover, this protocol was successfully applied to the asymmetric synthesis of a selective estrogen receptor modulator.

## Abstract 2

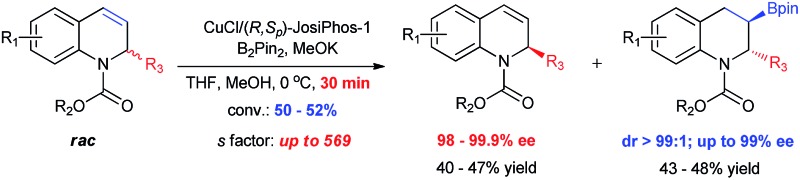
A highly efficient kinetic resolution of racemic 2-substituted 1,2-dihydroquinolines *via* asymmetric Cu-catalyzed borylation has been realized for the first time to afford chiral 3-boryl-1,2,3,4-tetrahydroquinolines and recover 2-substituted 1,2-dihydroquinolines with excellent enantioselectivities and selectivity factors of up to 569.

## Introduction

**Fig. 1:**
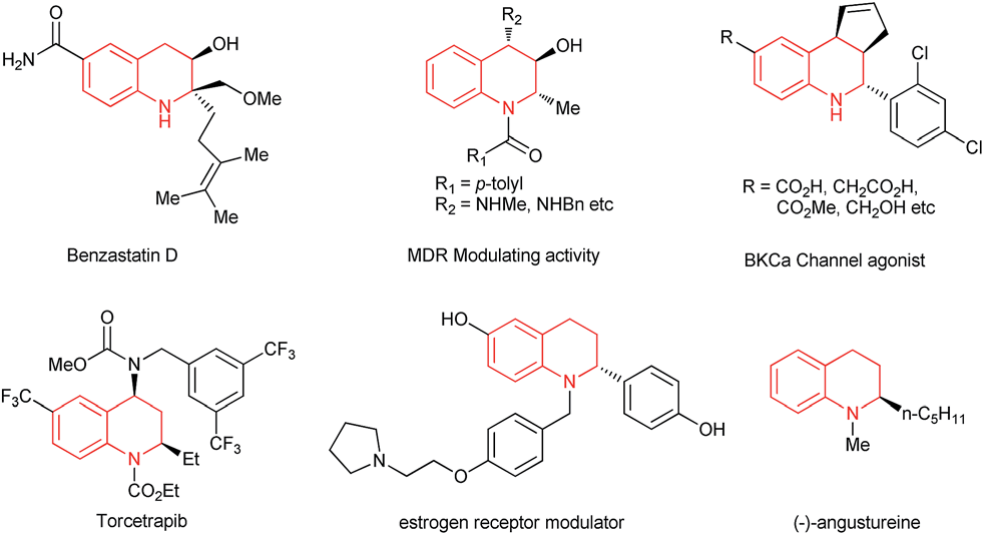
Selected bioactive compounds containing tetrahydroquinolines.

The optically active 1,2,3,4-tetrahydroquinoline framework constitutes a key synthetic intermediate in organic synthesis and is a privileged structural motif in a broad range of natural products, biologically active compounds,^1^ and pharmaceuticals such as benzastatin D, angustureine and the antihypercholesterolemia drug torcetrapib (Fig. 1).^2^ Therefore, optically active tetrahydroquinolines have elicited much interest, and a great deal of effort has been devoted to the development of convenient and general synthetic approaches,^1,3^ including intramolecular cyclization by Friedel–Crafts reactions, hydroamination, allylic amination or aza-Michael addition,^4^ asymmetric hydrogenation of 2-substituted quinolines,^5–8^ and enantioselective transition metal-catalyzed nucleophilic addition of quinolinium salts.^9–11^

On the other hand, chiral organoboron compounds, because of their utility in C–C and C–heteroatom bond formation, have greatly expanded the potential applications in organic chemistry, specifically as key intermediates in asymmetric synthesis. The direct catalytic enantioselective synthesis of organoboron compounds can be achieved by a number of reported methods,^12^ such as hydroboration,^13^ diboration,^14^ arylborylation,^15^ conjugate boron addition,^16^ and allylic substitution.^17^ Among these approaches, transition metal-catalyzed boration of prochiral C–C multiple bonds has recently attracted much interest in chemistry.^12c,18^ For instance, Ito and coworkers made an outstanding contribution to the Cu-catalyzed asymmetric hydroboration reaction and recently reported the stepwise dearomatization and enantioselective borylation of pyridines and quinolines with excellent enantioselectivities.^18b,c^

**Scheme 1:**
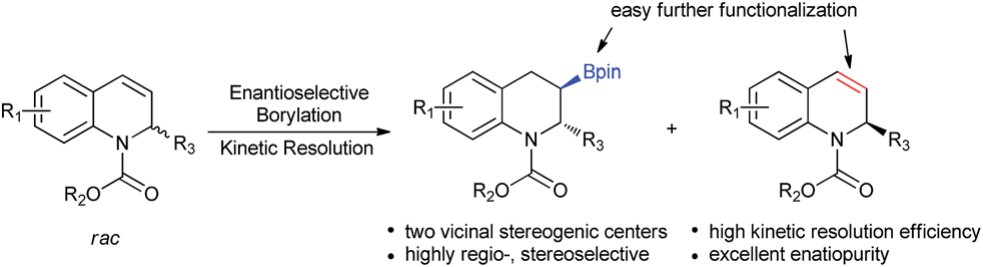
Kinetic resolution of 1,2-dihydroquinolines *via* Cu-catalyzed borylation.

Kinetic resolution, which provides a simple and efficient way to access both the chiral products and recover the starting materials, has attracted considerable attention from academy and industry.^19^ Most recently, several exciting developments in kinetic resolution have been reported.^20^ Transition metal-catalyzed allylic substitution, C–H iodination, C–H olefination, C–H cross-coupling and C–P coupling reactions have been successfully applied to kinetic resolution.^21^ In addition, tremendous efforts have also been made for the development of organocatalysts, such as phase-transfer catalysts, *N*-heterocyclic carbenes and Brønsted acids, for kinetic resolution.^22^ Despite this significant progress, to date, the number of reactions, catalysts and substrates suitable for kinetic resolution is still limited and a higher resolution efficiency is highly required. To the best of our knowledge, thus far, kinetic resolution *via* asymmetric borylation has not been explored. Therefore, we hypothesized the kinetic resolution of 2-substituted 1,2-dihydroquinolines *via* Cu-catalyzed borylation (Scheme 1), which can simultaneously afford both chiral organoboron compounds bearing two vicinal stereogenic centers and the enantiomerically enriched recovered 2-substituted 1,2-dihydroquinolines,^23^ which can be transformed to the corresponding tetrahydroquinolines by simple methods. Herein, we present the kinetic resolution of 2-substituted 1,2-dihydroquinolines *via* Cu-catalyzed borylation for the first time. With kinetic resolution selectivity factors of up to 569, the chiral organoboron compounds were obtained with excellent diastereoselectivity and enantioselectivities (dr > 99 : 1 and up to 99% ee), while the unreacted 2-substituted 1,2-dihydroquinolines were recovered with extremely high enantiopurities (98–99.9% ee) and in high yields.

## Results and discussion

**Fig. 2:**
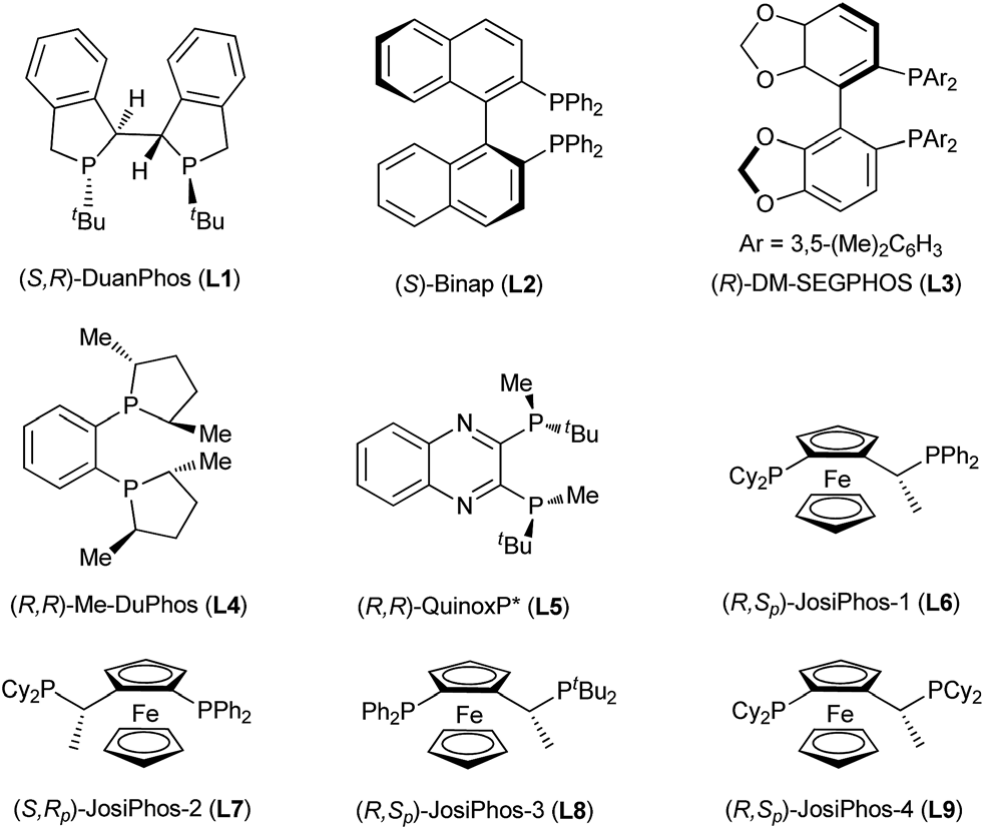
Structures of the phosphine ligands screened.

The asymmetric borylation/kinetic resolution of racemic methyl 2-(naphthalene-1-yl)quinoline-1(2*H*)-carboxylate (*rac*-**1a**) was initially evaluated with B_2_pin_2_ in THF at 0 °C, catalyzed by a complex formed from CuCl and (*S*,*R*)-DuanPhos in the presence of MeOK and MeOH as additives (Table 1, entry 1). We were pleased to find that the borylation product **2a** as a single diastereomer (dr > 99 : 1) and the recovered **1a** were obtained with 60% ee and 98% ee, respectively, corresponding to a selectivity factor (*s*) of 17.^19*a*^ Although the determination of the conversion-independent selectivity factor (*s* = ln[(1 – *C*) (1 – ee**_1a_**)]/ln[(1 – *C*) (1 + ee**_1a_**)]) was not straightforward due to the product containing two stereogenic centers, the extraordinary diastereoselectivity (dr > 99 : 1) allowed for the calculation of the true selectivity factor.^22*h*^ Then, we examined the effect of solvents including toluene, DME and DCE (entries 2–4). According to the enantioselectivity and efficiency of the kinetic resolution, THF was a better choice albeit slightly higher ee values of the recovered **1a** and borylation product **2a** were observed in toluene and DME, respectively. The screening of various chiral ligands, shown in Fig. 2, which are often used in Cu-catalyzed asymmetric boration reactions of C–C double bonds was subsequently carried out (entries 5–12). The use of (*S*)-Binap could provide good enantioselectivities for both the borylation product and the recovered starting material (entry 5), whereas (*R*)-DM-SEGPHOS, (*R*,*R*)-Me-Duphos and (*R*,*R*)-QuinoxP* afforded the recovered **1a** with very poor ee values, albeit with moderate to high enantioselectivities for the borylation product **2a** (entries 6–8). To our delight, in addition to a satisfactory conversion, (*R*,*S*_p_)-JosiPhos-1 dramatically increased the enantioselectivity of both product **2a** and recovered **1a** to 97% ee and 99.4% ee, respectively with an outstanding selectivity factor (*s*) of 251 (entry 9). Some other similar ligands proved to be inefficient for this kinetic resolution, with very poor enantioselectivity and selectivity factors (entries 10–12). In the end, the optimal reaction conditions were selected as CuCl/(*R*,*S*_p_)-JosiPhos-1/MeOK/THF.

**Table 1:** Optimization of reaction conditions^*a*^

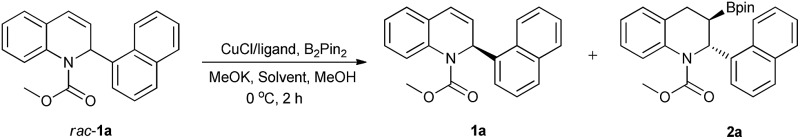						
Entry	Ligand	Solvent	Conv^*b*^ (%)	**1a**	**2a**	*S* ^*e*^
				ee^*c*^ (%)	ee^*c*^ ^,^ ^*d*^ (%)	
1	L1	THF	64	98	60	17
2	L1	Toluene	71	99	42	11
3	L1	DME	43	63	74	12
4	L1	DCE	44	45	57	5
5	L2	THF	53	92	83	29
6	L3	THF	20	7	50	1
7	L4	THF	21	24	88	25
8	L5	THF	29	38	95	40
**9**	**L6**	**THF**	**51**	**99.4**	**97**	**251**
10	L7	THF	37	37	64	6
11	L8	THF	39	23	36	3
12	L9	THF	31	5	31	2

^*a*^Reaction conditions: CuCl (0.025 mmol), ligand (0.025 mmol), *rac*-**1a** (0.5 mmol), B_2_Pin_2_ (0.6 mmol), MeOK (0.1 mmol), solvent (1.5 mL) MeOH (1.0 mmol), 0 °C, 2 h.

^*b*^Calculated conversion, *C* = ee**_1a_**/(ee**_1a_** + ee**_2a_**).

^*c*^Determined by chiral HPLC and SFC analysis.

^*d*^Diastereomeric ratio (dr) > 99 : 1 (determined by ^1^H NMR).

^*e*^Selectivity factor (*s*) = ln[(1 – *C*) (1 – ee**_1a_**)]/ln[(1 – *C*) (1 + ee**_1a_**)].

**Fig. 3:**
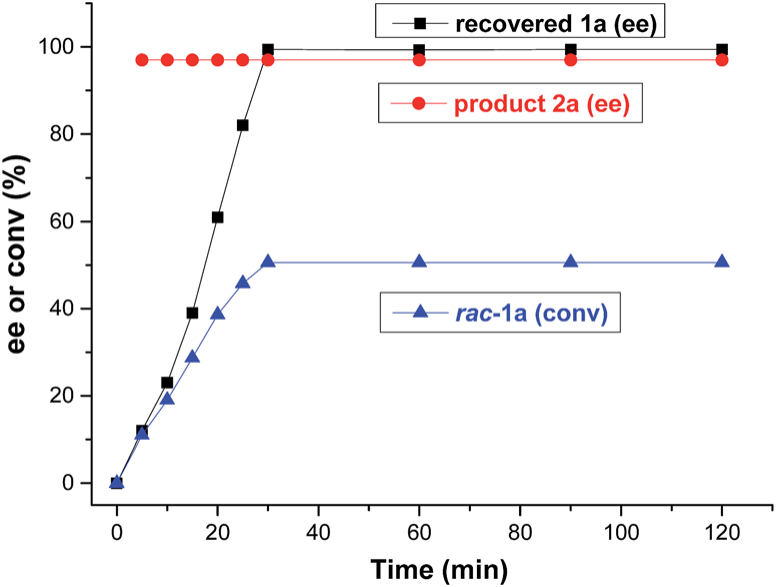
Plot of the enantioselectivity of product **2a** and recovered **1a**, and the conversion of *rac*-**1a** against reaction time.

In the ideal kinetic resolution of a racemate, only one of the two enantiomers reacts, and the other one is recovered (*i.e.*, *C* = 50%). To explore the kinetic resolution of *rac*-**1a** in more detail, the ee values of product **2a** and recovered **1a**, and the conversion of *rac*-**1a** were monitored from the start of the reaction (Fig. 3). Remarkably, the enantiomeric excess of recovered **1a** increased from 0 to 99.4% after 30 minutes and remained constant thereafter. Moreover, the borylation product **2a** was afforded with an almost unaltered excellent enantioselectivity of 97% ee, even if the kinetic resolution lasted for 2 hours. These results revealed that the kinetic resolution of *rac*-**1a** *via* Cu-catalyzed borylation was highly effective and almost perfect. In order to confirm that only one enantiomer reacts, we performed the borylation of recovered **1a** (99.4% ee) under identical reaction conditions and, as expected, almost no borylated product was observed.

Under the optimized reaction conditions, the kinetic resolution of various racemic 2-substituted 1,2-dihydroquinolines (*rac*-**1**) *via* asymmetric Cu-catalyzed borylation was investigated (Table 2). All of the racemic 1,2-dihydroquinolines **1** were resolved smoothly with selectivity factors of up to 569 to afford the corresponding chiral borylated tetrahydroquinolines **2** as the single diastereomer with up to 99% ee and the recovered 2-substituted 1,2-dihydroquinolines **1** in high yields with excellent enantioselectivities (98–99.9% ee). It is notable that various carbamate moieties of 1,2-dihydroquinolines have no obvious effect on the kinetic resolution, affording the borylation products **2a–2e** with 91–97% ee and the recovered products **1a–1e** with 98–99.9% ee, corresponding to selectivity factors (*s*) of 111 to 251. The racemic 1,2-dihydroquinolines bearing a Me or MeO group at the 6- or 7-position were also tolerated, furnishing the chiral products **2f–2h** in high yields, with excellent enantioselectivities (95–96% ee) and selectivity factors (*s* = 229–251). In addition, replacing 1-naphthyl with 2-naphthyl led to a similar result, producing the borylation product **2i** with 94% ee and recovered product **1i** with 98% ee, with a selectivity factor (*s*) of 211. Gratifyingly, substituted phenyl groups at the 2-position of 1,2-dihydroquinoline proved to have no effect on kinetic resolution. Thus, excellent ee values for both borylation products and recovered materials were achieved with high selectivity factors (*s* = 99–569) irrespective of the electronic properties or position of the substituents in the 2-phenyl group, affording **2j–2p** with 90–99% ee and recovered product **1j–1p** with over 99% ee. Remarkably, the *o*-tolyl substituted substrate *rac*-**1k** provided the product **2k** with both the highest enantioselectivity (99% ee) and a selectivity factor of 569. It is noteworthy that the 2-alkyl substituted substrates **1q** and **1r** could also be resolved with high selectivity factors (99 and 145, respectively), giving the corresponding borylation products **2q** and **2r** with comparable enantioselectivities (90% ee and 93% ee), and recovered products **1q** and **1r** with an identical ee of 99%.

**Table 2:** Substrate scope^*a*^ ^,^ ^*b*^

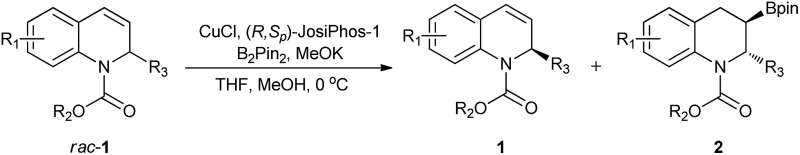
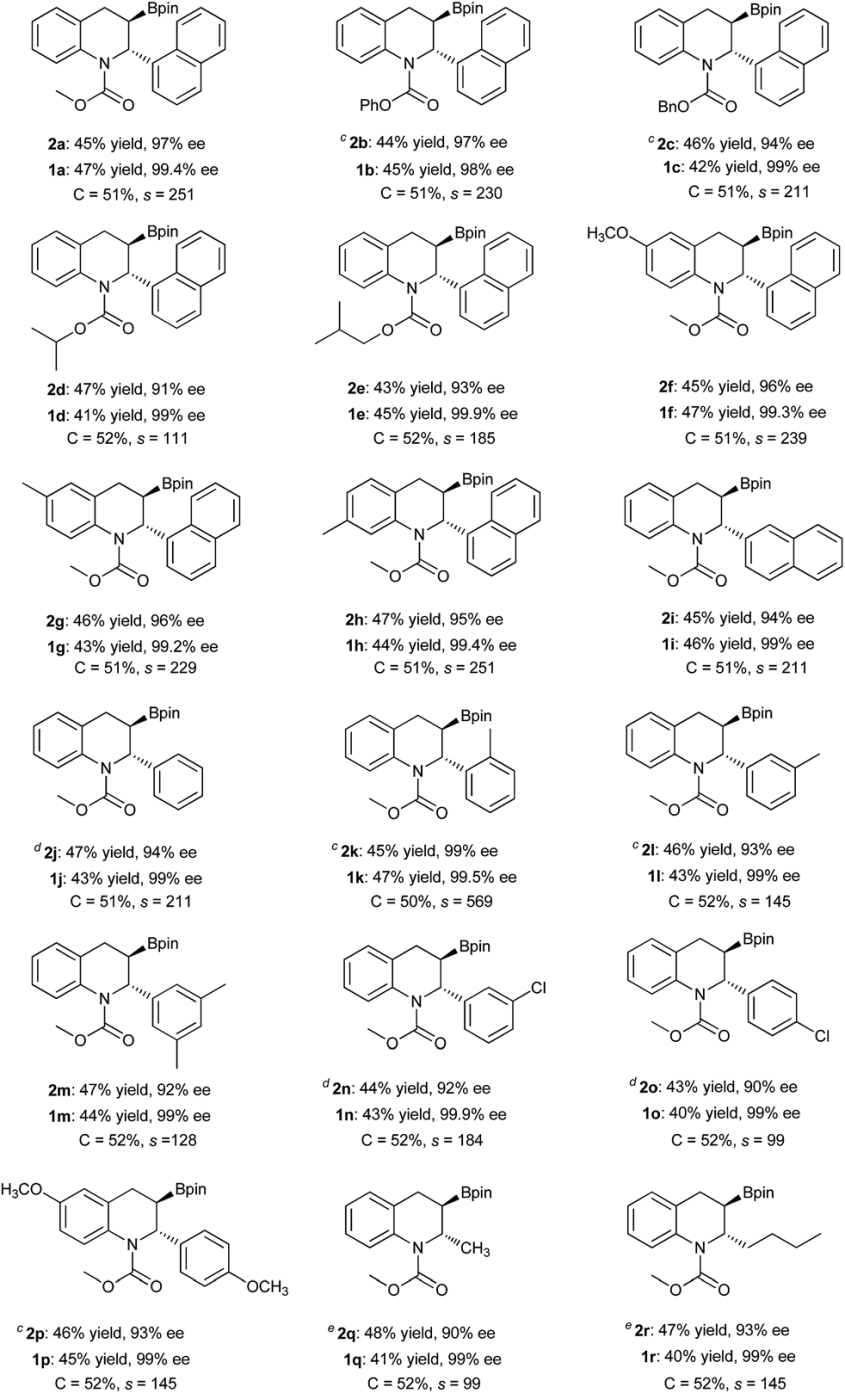

^*a*^Unless otherwise mentioned, all reactions were performed with CuCl (0.025 mmol), (*R*,*S*_p_)-JosiPhos-1 (0.025 mmol), *rac*-**1** (0.5 mmol), B_2_Pin_2_ (0.6 mmol), MeOK (0.1 mmol), THF (1.5 mL), MeOH (1.0 mmol), 0 °C, 30 min.

^*b*^Isolated yield; calculated conversion, *C* = ee**_1_**/(ee**_1_** + ee**_2_**); enantiomeric excess (ee) was determined by chiral HPLC or SFC analysis using a chiral stationary phase; diastereomeric ratio (dr) > 99 : 1 (determined by ^1^H NMR). Selectivity factor (*s*) = ln[(1 – *C*) (1 – ee**_1_**)]/ln[(1 – *C*) (1 + ee**_1_**)].

^*c*^THF/toluene/DME = 2 : 1 : 1 (1.5 mL), 2 h.

^*d*^ ^*t*^BuOK (0.1 mmol), THF/toluene/DME = 1 : 1 : 1 (1.5 mL), 2 h.

^*e*^THF/toluene/DME = 1 : 1 : 1 (1.5 mL), 2 h.

**Fig. 4:**
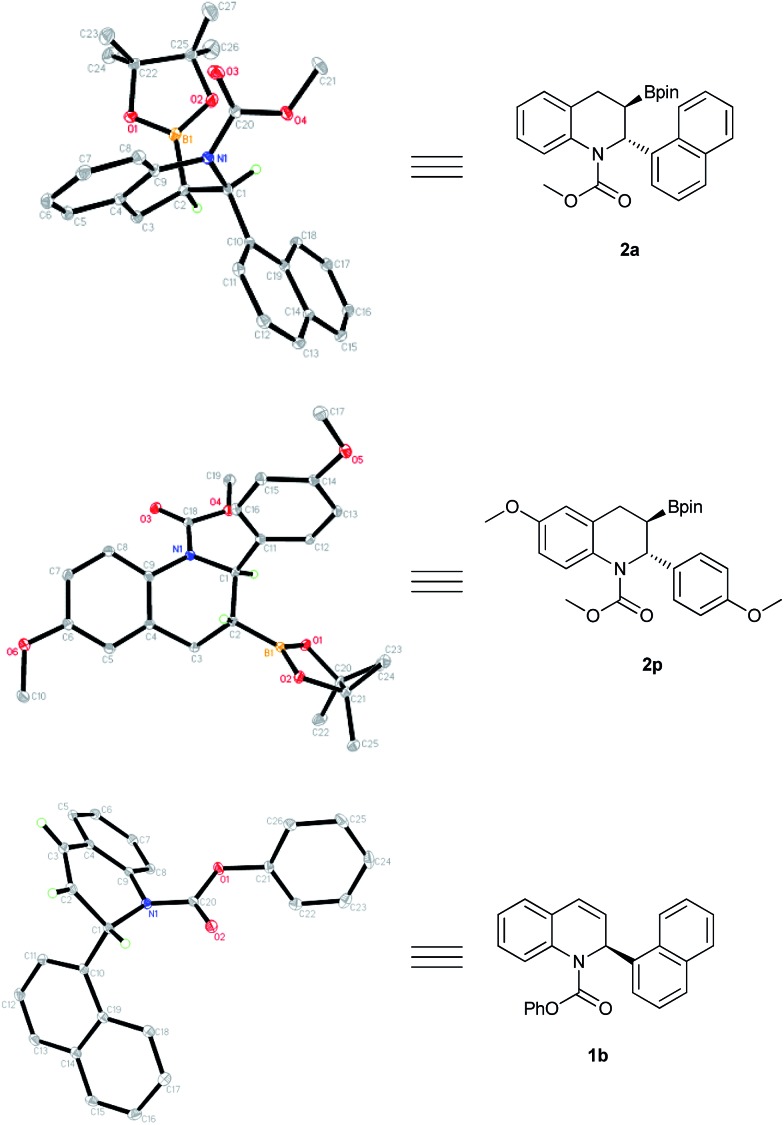
X-ray crystallographic analysis of products **2a**, **2p** and recovered **1b**.

The generated borylation products **2a** and **2p** were the trans isomers, with an absolute configuration of (2*R*,3*R*), determined by X-ray crystallography. Likewise, recovered product **1b** was assigned the (*S*) configuration according to the corresponding single-crystal structure (Fig. 4).

However, this strategy was not effective for the kinetic resolution of simple olefins. Thus, under the optimized reaction conditions, the borylation of substrates **1s** and **1t** did not proceed at all. The *rac*-**1s** and **1t** were completely recovered.
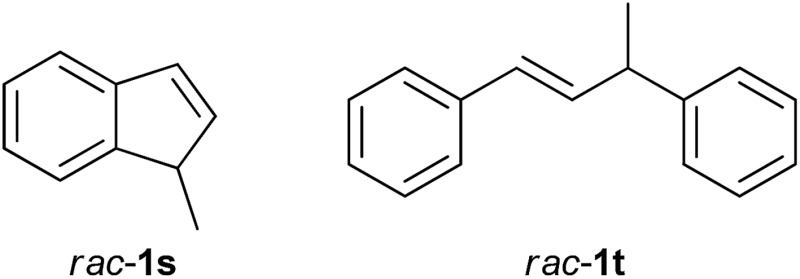


**Scheme 2:**
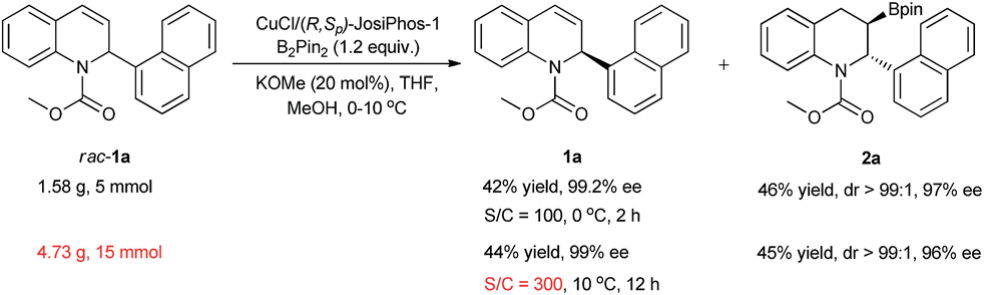
Kinetic resolution of *rac*-**1a** on a gram scale with a lower catalyst loading.

To demonstrate the potential of the kinetic resolution, turnover number (TON) experiments were conducted on a gram-scale. With a lower catalyst loading of 1.0 mol% (TON = 100), the kinetic resolution of *rac*-**1a** was completed, providing the desired product **2a** in 46% yield with 97% ee and the recovered product **1a** in 42% yield with 99.2% ee. With a much lower catalyst loading of 0.33 mol% (TON = 300), *rac*-**1a** (15 mmol, 4.73 g) was smoothly resolved at 10 °C to produce the corresponding product **2a** in 45% yield with 96% ee and the recovered product **1a** in 44% yield with 99% ee (Scheme 2), which represents the highest activity achieved in Cu-catalyzed borylation reactions to date.

**Scheme 3:**
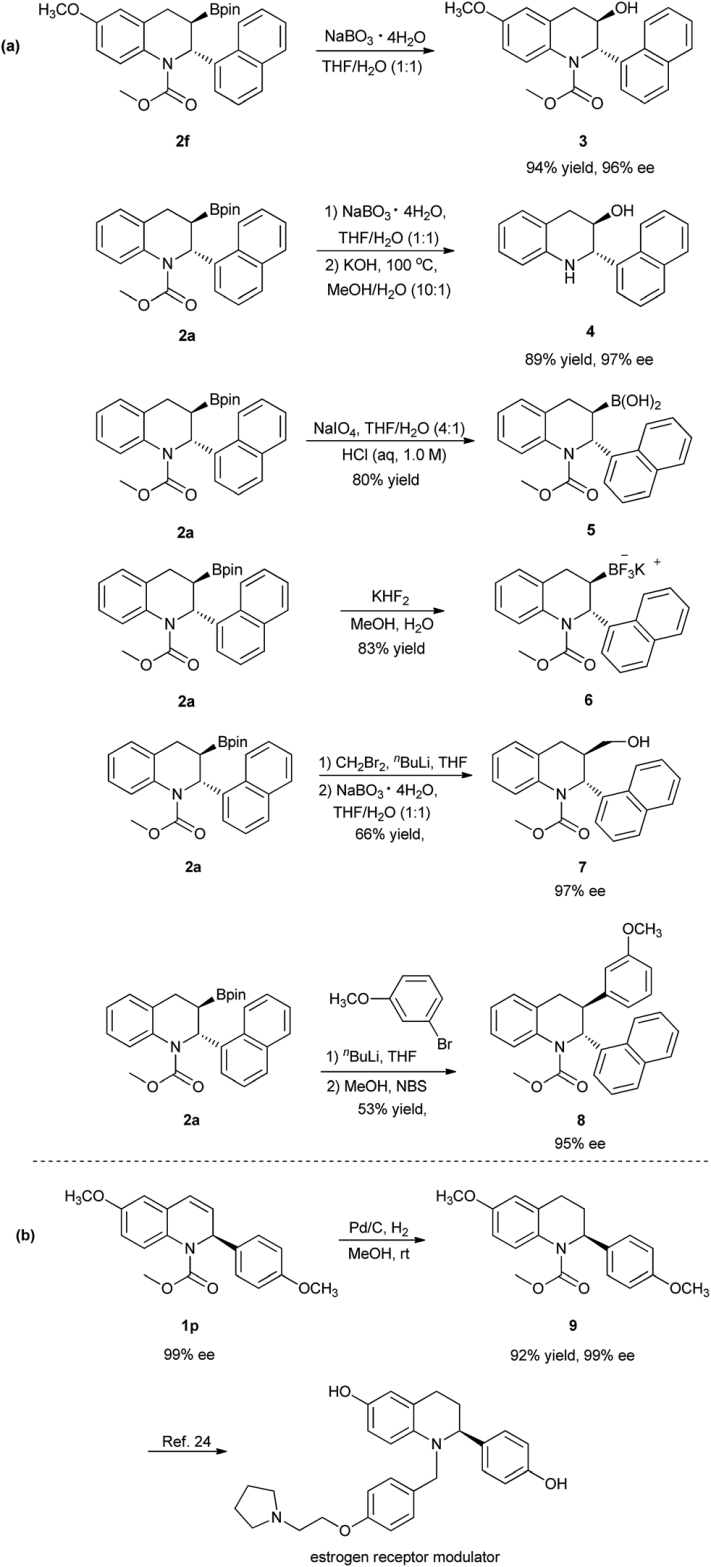
Representative transformations of products **2** and enantiomerically enriched recovered starting material **1**.

The chiral 3-boryl-tetrahydroquinolines **2** are versatile synthetic intermediates that can be easily converted to various derivatives (Scheme 3(a)).^18e,24^ For example, product **2f** could be converted to the chiral 3-hydroxyl tetrahydroquinoline **3** bearing two vicinal stereogenic centers by oxidation with NaBO_3_, in high yields and without any loss of enantioselectivity. The oxidation of **2a**, followed by the hydrolysis of the carbamate afforded the chiral 3-hydroxyl tetrahydroquinoline **4** with maintained enantioselectivity, 97% ee. Compound **2a** could also be successfully transformed into both the corresponding boronic acid **5** and the trifluoroborate salt **6** in good yields. Moreover, **2a** could also be converted to the primary alcohol **7** and compound **8** with high enantioselectivities by homologation and Suzuki–Miyaura coupling, respectively.^12b,25^ In addition, a simple Pd/C-catalyzed hydrogenation of the chiral recovered compound **1p** with 99% ee afforded the tetrahydroquinoline **9** with retention of configuration, which was subsequently applied to the enantioselective synthesis of selective estrogen receptor modulators (Scheme 3(b)).^26^

**Scheme 4:**
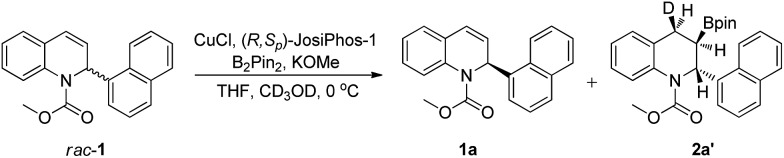
Deuterium labeling experiment.

In order to elucidate the reaction mechanism, a labeling experiment with deuterium was performed (see the ESI†). The borylation of *rac*-**1a** under the optimized conditions using CD_3_OD instead of CH_3_OH gave the product **2a** labelled with deuterium at the 4-position (>95% D), with excellent enantioselectivity, 97% ee (Scheme 4). The *syn* configuration between the deuterium atom at the 4-position and the boryl group at the 3-position indicated that the addition of the Cu–B complex to the C–C double bond of *rac*-**1a** occurred in a *syn* fashion.

**Fig. 5:**
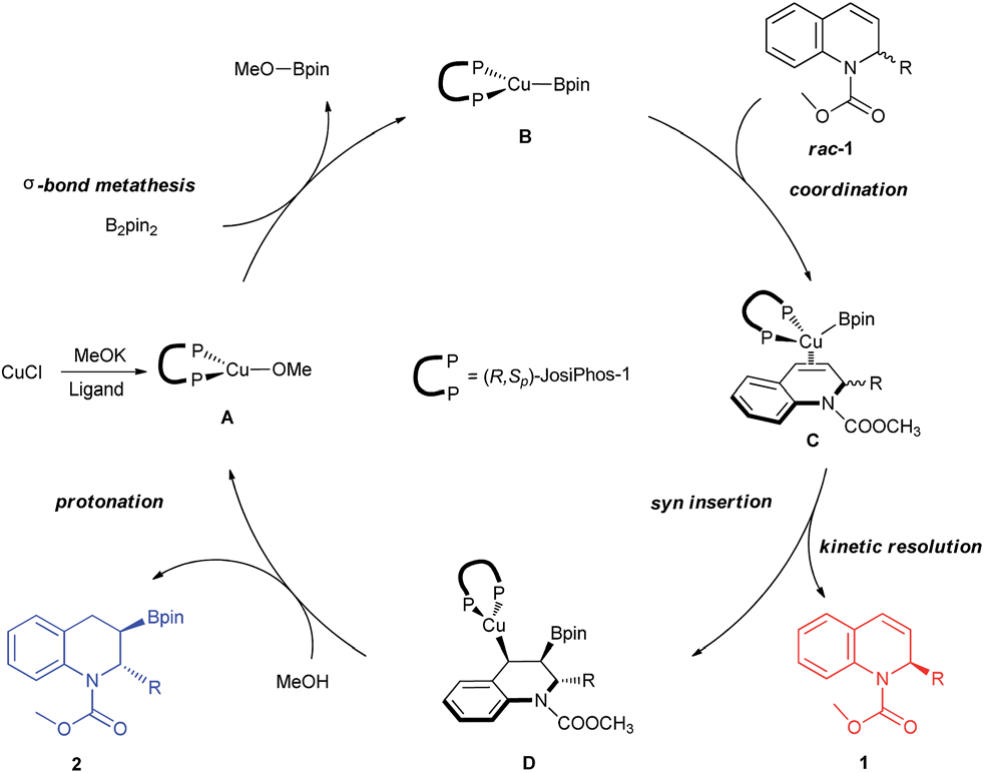
Proposed catalytic cycle for the kinetic resolution *via* Cu-catalyzed borylation.

Based on previous reports and our experiment,^18c,27^ a feasible mechanism for the kinetic resolution of 2-substituted 1,2-dihydroquinolines *via* Cu-catalyzed borylation was proposed, as shown in Fig. 5. The initial exchange of CuCl with the ligand and MeOK resulted in the formation of the Cu–OMe complex **A**, followed by a σ-bond metathesis with B_2_pin_2_ to generate the borylcopper species **B**. Subsequently, the coordination of the racemic 2-substituted 1,2-dihydroquinoline **1** to copper gave complex **C**. The *syn*-addition furnished the borylated alkylcopper intermediate **D** along with the chiral recovered starting material **1**. Finally, the protonation of **D** by MeOH formed the corresponding borylation product **2** and regenerated the Cu–OMe complex **A**.

## Conclusions

In conclusion, we have presented the first kinetic resolution of 2-substituted 1,2-dihydroquinolines by asymmetric Cu-catalyzed borylation under mild reaction conditions, achieving excellent enantiodiscrimination and kinetic resolution. A wide range of chiral 3-boryl-1,2,3,4-tetrahydroquinolines containing two vicinal stereogenic centers and recovered 2-substituted 1,2-dihydroquinolines were obtained after 30 minutes in high yields with 90–99% ee (dr > 99 : 1) and over 98% ee, respectively, corresponding to kinetic selectivity factors of up to 569. Finally, this protocol was successfully applied to the asymmetric synthesis of selective estrogen receptor modulators.
