# Supplementary material for: Kinetic resolution of racemic 2-substituted 1,2-dihydroquinolines via asymmetric Cu-catalyzed borylation
Source: Chem Sci. 2017 Apr 19;8(6):4558–64. doi: 10.1039/c7sc01556a (PMC5590099; doi:10.1039/c7sc01556a)
Supplement: Supplementary file 1 [file SC-008-C7SC01556A-s001.pdf]

Electronic Supplementary Material (ESI) for Chemical Science.
This journal is © The Royal Society of Chemistry 2017

Supplementary Information for:

Kinetic Resolution of 2-Substituted 1,2-Dihydroquinolines *via* Asymmetric Cu-Catalyzed Borylation Reaction

Duanyang Kong, Suna Han, Rui Wang, Meina Li, Guofu Zi, and Guohua Hou*

Key Laboratory of Radiopharmaceuticals, College of Chemistry, Beijing Normal University, Beijing 100875, China

Table of contents

1. General Information ……………………………………………………..…..…S2

2. Experimental Procedure and Characterization…………….…………….……..…S2

3. Procedure for the synthesis of **3-9**………………………………..………..…......S17

4. Deuterium Labeling Experiment………………...………….….……….....………S22

5. X-ray Crystallography………………...…………….…………………....………S23

6. NMR spectra of all compounds..……………………………..…...….………….S26

7. SFC and HPLC spectra of all compounds..………..……….……......…………S71

8. Figures of single-crystals…..………….…..……........................……………S112

1. General Information

All the air or moisture sensitive reactions and manipulations were performed by using standard Schlenk techniques and in a nitrogen-filled glovebox. DME, THF, dioxane and toluene were distilled from sodium benzophenone ketyl. CH_2Cl_2 was distilled from calcium hydride. Anhydrous MeOH was distilled from magnesium. ^{1}H NMR and ^{13}C NMR spectra were recorded on Bruker AV (400 MHz) spectrometers and JEOL JNM-ECX600P and JNM-ECS600 (600 MHz) spectrometers ($CDC1_3$ was the solvent used for the NMR analysis, with TMS as the internal standard. Chemical shifts were reported upfield to TMS (0.00 ppm) for ^{1}H NMR. Data is represented as follows: chemical shift, integration, multiplicity (s = singlet, d = doublet, dd = double of doublets, t = triplet, q = quartet, m = multiplet) and coupling constants (*J*) in Hertz (Hz). Optical rotation was determined using Autopol III Automatic polarimeter (Rudolph research Analyical). HPLC analysis was conducted on Agilent 1260 series instrument. SFC analysis was conducted on Agilent 1260 series instrument. HRMS were recorded on a Waters LCT Premier XE mass spectrometer with APCI or ESI.

2. Experimental Procedure and Characterization

General procedure for the preparation of substrates [1]

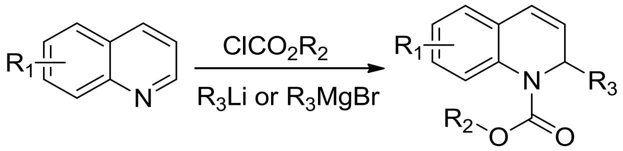

To a solution of quinoline or substituted quinoline (10 mmol) in dry THF or Et_2O (20.0 mL) was added dropwise RMgBr (12 mmol) or *n*-BuLi (12 mmol, 2.4 M solution in hexane) at -78°C under a nitrogen atmosphere. After 2 h at -78 ^{o}C, $ClCO_2R$ (12 mmol) was added and the solution became yellow. After 10-30 min, the solution was quenched with a solution of NH_4Cl, extracted with EtOAc. The organic layers were dried over $MgSO_4$, filtered and evaporated. The crude oil was purified by chromatography on silica gel.

General procedure of the kinetic resolution

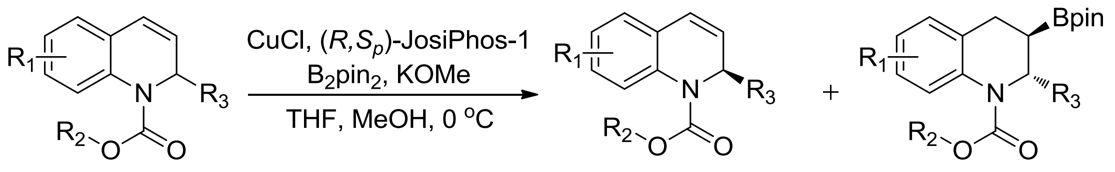

rac-**1** **1** **2**

In a nitrogen-filled glovebox, CuCl (0.025 mmol), (R,S_p)-JosiPhos-1 (0.025 mmol), B_2pin_2 (0.6 mmol) and KOMe (0.1 mmol) were placed in an oven-dried Schleck reaction vial, which was sealed with a rubber plug. The Schleck reaction vial was then removed from glovebox. THF (1.5 mL) was added to the Schleck vial through the rubber plug. After *rac*-**1** (0.50 mmol) was added to the mixture at 0 ℃, MeOH (1.0 mmol) was added dropwise. Upon completion of the reaction, the reaction mixture was passed through a short silica gel column eluting with Et_2O. The crude mixture was purified by chromatography on silica gel to give the corresponding borylation product **2** and the recovered starting material **1**. The ee values of **1** and **2** were determined by HPLC or SFC analysis on a chiral stationary phase. Diastereomeric ratio was determined by ^{1}H NMR spectroscopy of the crude mixture.

Characterization of the products

(*S*)-methyl 2-(naphthalen-1-yl)quinoline-1(2*H*)-carboxylate (1a): Yield: 47%; ^{1}H

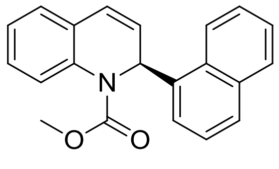

NMR (CDCl$_3$, 400 MHz) δ: 8.55 (d, J = 7.8 Hz, 1H), 7.85 (d, J = 8.2 Hz, 1H), 7.72 (d, J = 8.1 Hz, 1H), 7.64 (t, J = 7.1 Hz, 1H), 7.52 (t, J = 7.3 Hz, 2H), 7.31 (d, J = 7.1 Hz, 1H), 7.26-7.10 (m, 4H), 7.01 (d, J = 5.7 Hz, 1H), 6.67 (d, J = 9.6 Hz, 1H), 6.32 (dd, J = 9.5 Hz, 6.0 Hz, 1H), 3.80 (s, 3H). ^{13}C NMR (CDCl$_3$, 100 MHz) δ: 155.9, 135.8, 134.6, 130.9, 129.5, 129.4, 129.1, 128.3, 127.8, 127.0, 126.8, 126.2, 125.9, 125.8, 125.3, 124.2, 53.9, 53.3. TOF-HRMS Calcd. for $C_{21}H_{17}NO_2Na$ [M+Na$^+$]: 338.1151, found 338.1153. 99.4% ee; $[\alpha]_D^{25}$ = -866 (c = 1.0, CHCl$_3$); SFC condition: Lux 5u Cellulose-1 (250 × 4.60 mm), CO_2 : MeOH = 80:20, 2.5 mL/min, 254 nm; t_A = 6.5 min (minor), t_B = 7.6 min (major).

(*S*)-phenyl 2-(naphthalen-1-yl)quinoline-1(2*H*)-carboxylate (1b): Yield: 45%; ^{1}H NMR (CDCl$_3$, 400 MHz) δ: 8.76 (s, 1H), 7.73-7.69 (m, 2H), 7.61-7.58 (m, 3H), 7.55-7.38 (m, 3H), 7.39-7.14 (m, 8H), 6.81 (d, J = 9.2 Hz, 1H), 6.42 (dd, J = 9.5 Hz,

6.0 Hz, 1H). ^{13}C NMR ($CDCl_3$, 100 MHz) δ: 153.5, 151.3, 134.9, 134.3, 131.2, 130.7, 129.6, 129.3, 129.1, 128.2, 127.9, 126.9, 126.6, 126.0, 125.9, 125.6, 125.5, 123.9, 121.9, 53.2. TOF-HRMS Calcd. for $C_{26}H_{19}NO_2Na$ [M+Na$^+$]: 400.1308, found 400.1307. 98% ee; $[\alpha]_D^{25}$ = -774 (c = 1.0, $CHCl_3$); SFC condition: Lux 5u Cellulose-3 (250 × 4.60 mm), CO_2 : MeOH = 75:25, 3.0 mL/min, 230 nm; t_A = 1.7 min (major), t_B = 2.2 min (minor).

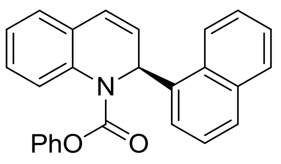

(*S*)-benzyl 2-(naphthalen-1-yl)quinoline-1(2*H*)-carboxylate (1c): Yield: 42%; ^{1}H NMR ($CDCl_3$, 400 MHz) δ: 8.61 (s, 1H), 7.93 (d, *J* = 8.0 Hz, 1H), 7.80-7.58 (m, 4H), 7.49 (s, 1H), 7.37-7.17 (m, 10H), 6.71 (d, *J* = 9.5Hz, 1H), 6.36 (dd, *J* = 9.2 Hz, 6.0 Hz, 1H), 5.44 (d, *J* = 12.5 Hz, 1H), 5.31 (d, *J* = 12.4 Hz, 1H). ^{13}C NMR ($CDCl_3$, 100 MHz) δ: 155.3, 136.7, 135.9, 134.7, 131.0, 129.5, 129.4, 129.3, 129.2, 128.7, 128.6, 128.5, 127.9, 127.2, 127.1, 126.4, 126.1, 125.8, 125.4, 124.3, 68.7, 53.6. TOF-HRMS Calcd. for $C_{27}H_{22}NO_2$ [M+H$^+$]: 392.1645, found 392.1646. 99% ee; $[\alpha]_D^{25}$ = -828 (c = 1.0, $CHCl_3$); SFC condition: Lux 5u Cellulose-3 (250 × 4.60 mm), CO_2 : MeOH = 70:30, 3.0 mL/min; t_A = 2.1 min (major), t_B = 2.3 min (minor).

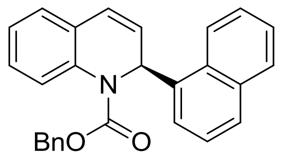

(*S*)-isopropyl 2-(naphthalen-1-yl)quinoline-1(2*H*)-carboxylate (1d): Yield: 41%; ^{1}H NMR ($CDCl_3$, 400 MHz) δ: 8.55 (d, *J* = 8.5 Hz, 1H), 7.84 (d, *J* = 8.2 Hz, 1H), 7.71 (d, *J* = 8.1 Hz, 1H), 7.61 (t, *J* = 7.3 Hz, 1H), 7.50 (t, *J* = 7.2 Hz, 2H) 7.31 (d, *J* = 7.5 Hz, 1H), 7.26-7.07 (m, 4H), 6.99 (d, *J* = 5.9 Hz, 1H), 6.65 (d, *J* = 9.6 Hz, 1H), 6.28 (dd, *J* = 15.5 Hz, 6.0 Hz, 1H), 5.07-5.04 (m, 1H), 1.57 (s, 1H), 1.32 (d, *J* = 6.2 Hz, 3H), 1.18 (d, *J* = 6.2 Hz, 3H). ^{13}C NMR ($CDCl_3$, 100 MHz) δ: 154.9, 136.0, 134.5, 130.8, 129.4, 129.3, 128.9, 128.2, 127.7, 126.9, 126.8, 126.1, 126.0, 125.7, 125.3, 124.9, 124.2, 53.1, 22.5. TOF-HRMS Calcd. for $C_{23}H_{22}NO_2$ [M+H$^+$]: 344.1645, found 344.1644. 99% ee; $[\alpha]_D^{25}$ = -628 (c = 1.0, $CHCl_3$); SFC condition: Lux 5u Cellulose-3 (250 × 4.60 mm), CO_2 : MeOH = 90:10, 3.0 mL/min, 210 nm; t_A = 3.9 min (major), t_B = 5.7 min (minor).

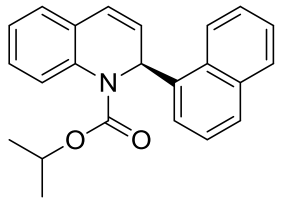

(*S*)-isobutyl 2-(naphthalen-1-yl)quinoline-1(2*H*)-carboxylate (1e): Yield: 45%; ^{1}H

NMR (CDCl$_3$, 400 MHz) δ: 8.64 (s, 1H), 7.89 (d, J = 8.2 Hz, 1H), 7.76 (d, J = 8.1

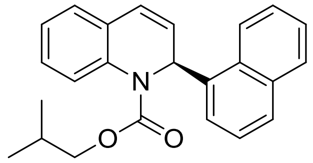

H$_Z$, 1H), 7.70 (t, J = 7.1 Hz, 2H), 7.56 (t, J = 7.2 Hz, 1H) 7.44 (d, J = 7.1 Hz, 1H), 7.28 (d, J = 7.9 Hz, 2H), 7.20-7.13 (m, 3H), 6.68 (d, J = 9.5 Hz, 1H), 6.35 (dd, J = 9.5, 6.0 Hz, 1H), 4.16-4.03 (m, 2H), 2.01-1.94 (m, 1H), 0.92 (t, J = 6.5 Hz, 6H). ^{13}C NMR (CDCl$_3$, 100 MHz) δ: 155.6, 136.1, 134.7, 131.0, 129.5, 129.1, 128.3, 127.8, 127.1, 127.0, 126.3, 126.0, 125.8, 125.2, 124.3, 73.2, 53.5, 28.5, 19.7. TOF-HRMS Calcd. for $C_{24}H_{24}NO_2$ [M+H$^+$]: 358.1801, found 358.1800. 99.9% ee; $[\alpha]_D^{25}$ = -688 (c = 1.0, CHCl$_3$); SFC condition: Lux 5u Cellulose-3 (250 × 4.60 mm), CO$_2$: MeOH = 90:10, 3.0 mL/min, 210 nm; t_A = 2.1 min (major), t_B = 2.3 min (minor).

(*S*)-methyl 6-methoxy-2-(naphthalen-1-yl)quinoline-1(2*H*)-carboxylate (1f): Yield:

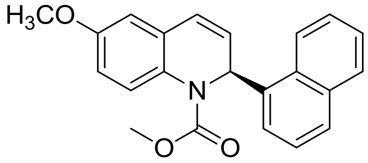

47%; ^{1}H NMR (CDCl$_3$, 400 MHz) δ: 8.62 (s, 1H), 7.86 (d, J = 8.1 Hz, 1H), 7.74 (d, J = 8.1 Hz, 1H), 7.66 (t, J = 7.2 Hz, 1H), 7.53 (t, J = 7.2 Hz, 1H), 7.35-7.23 (m, 3H), 7.03 (s, 1H), 6.78-6.65 (m, 3H), 6.35 (dd, J = 9.5 Hz, 6.0 H$_Z$, 1H), 3.81 (d, J = 3.1 H$_Z$, 6H). ^{13}C NMR (CDCl$_3$, 100 MHz) δ: 156.7, 155.6, 134.2, 130.7, 129.9, 128.9, 128.7, 128.3, 126.7, 125.8, 125.6, 125.5, 123.9, 113.3, 111.2, 55.5, 53.5, 52.7. TOF-HRMS Calcd. for $C_{22}H_{20}NO_3$ [M+H$^+$]: 346.1437, found 346.1441. 99.3% ee; $[\alpha]_D^{25}$ = -765 (c = 1.0, CHCl$_3$); SFC condition: Lux 5u Cellulose-3 (250 × 4.60 mm), CO$_2$: MeOH = 90:10, 3.0 mL/min, 210 nm; t_A = 2.6 min (major), t_B = 3.5 min (minor).

(*S*)-methyl 6-methyl-2-(naphthalen-1-yl)quinoline-1(2*H*)-carboxylate (1g): Yield:

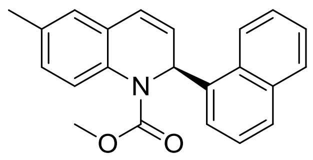

43%; ^{1}H NMR (CDCl$_3$, 400 MHz) δ: 8.69 (s, 1H), 7.92-7.02 (m, 10H), 6.67 (d, J = 7.8 Hz, 1H), 6.37-6.33 (m, 1H), 3.86 (s, 3H), 2.39 (s, 3H). ^{13}C NMR (CDCl$_3$, 100 MHz) δ: 156.1, 135.7, 135.0, 134.7, 133.3, 131.1, 129.5, 129.2, 129.1, 127.8, 127.5, 127.2, 126.3, 126.0, 125.6, 124.4, 54.0, 53.4, 21.6. TOF-HRMS Calcd. for $C_{22}H_{19}NO_2Na$ [M+Na$^+$]: 352.1308, found 352.1305. 99.2% ee; $[\alpha]_D^{25}$ = -837 (c = 1.0, CHCl$_3$); SFC condition: Lux 5u Cellulose-3 (250 × 4.60 mm), CO$_2$: MeOH = 75:25, 3.0 mL/min, 210 nm; t_A = 2.7 min (major), t_B = 4.2 min (minor).

(*S*)-methyl 7-methyl-2-(naphthalen-1-yl)quinoline-1(2*H*)-carboxylate (1h): Yield:

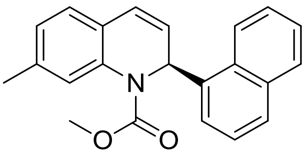

44%; ^{1}H NMR (CDCl$_3$, 400 MHz) δ: 8.62 (s, 1H), 7.89 (d, J = 8.0 Hz, 1H), 7.77-7.68 (m, 2H), 7.58-7.27 (m, 4H), 7.09-6.96 (m, 3H), 6.67 (d, J = 8.7 Hz, 1H), 6.29 (t, J = 6.5 Hz, 1H), 3.85 (s, 3H), 2.37 (s, 3H). ^{13}C NMR (CDCl$_3$, 100 MHz) δ: 156.0, 138.4, 135.7, 134.6, 131.0, 129.5, 129.1, 128.3, 127.1, 126.7, 126.3, 126.2, 126.1, 125.8, 125.4, 124.3, 54.0, 53.4, 22.3. TOF-HRMS Calcd. for $C_{22}H_{19}NO_2Na$ [M+Na$^+$]: 352.1308, found 352.1305. 99.4% ee; $[\alpha]_D^{25}$ = -817 (c = 1.0, CHCl$_3$); SFC condition: Lux 5u Cellulose-3 (250 × 4.60 mm), CO_2 : MeOH = 75:25, 3.0 mL/min, 210 nm; t_A = 2.5 min (major), t_B = 3.5 min (minor).

(*S*)-methyl 2-(naphthalen-2-yl)quinoline-1(2*H*)-carboxylate (1i): Yield: 46%; ^{1}H NMR (CDCl$_3$, 600 MHz) δ: 7.73-7.68 (m, 4H), 7.41-7.38 (m, 4H), 7.19-7.01 (m, 3H),

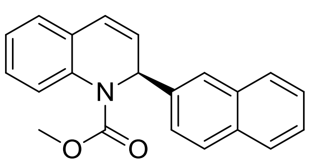

6.69 (d, J = 9.5 Hz, 1H), 6.34-6.24 (m, 2H), 3.83 (s, 3H). ^{13}C NMR (CDCl$_3$, 150 MHz) δ: 155.4, 136.9, 134.7, 133.3, 133.0, 128.6, 128.2, 127.9, 127.7, 127.3, 126.5, 126.2, 126.1, 125.9, 125.3, 124.8, 124.6, 55.8, 53.4. TOF-HRMS Calcd. for $C_{21}H_{17}NO_2Na$ [M+Na$^+$]: 338.1151, found 338.1153. 99% ee; $[\alpha]_D^{25}$ = -685 (c = 1.0, CHCl$_3$); SFC condition: Lux 5u Cellulose-3 (250 × 4.60 mm), CO_2 : MeOH = 75:25, 3.0 mL/min, 210 nm; t_A = 4.3 min (major), t_B = 5.4 min (minor). [1]

(*S*)-methyl 2-phenylquinoline-1(2*H*)-carboxylate (1j): Yield: 43%; ^{1}H NMR

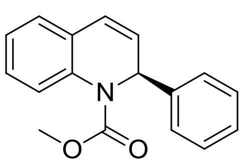

(CDCl$_3$, 400 MHz) δ: 7.50 (s, 1H), 7.28-7.18 (m, 6H), 7.12-7.03 (m, 2H), 6.65 (d, J = 8.6 Hz, 1H), 6.22-6.17 (m, 2H), 3.84 (s, 3H). ^{13}C NMR (CDCl$_3$, 100 MHz) δ: 155.3, 139.7, 134.8, 128.7, 128.4, 127.9, 127.3, 127.2, 126.5, 125.5, 124.7, 124.5, 55.8, 53.3. TOF-HRMS Calcd. for $C_{17}H_{15}NO_2Na$ [M+Na$^+$]: 288.0995, found 288.0995. 99% ee; $[\alpha]_D^{25}$ = -694 (c = 1.0, CHCl$_3$); SFC condition: Lux 5u Cellulose-3 (250 × 4.60 mm), CO_2 : MeOH = 75:25, 3.0 mL/min, 230 nm; t_A = 2.1 min (major), t_B = 2.3 min (minor). [1]

(*S*)-methyl 2-(o-tolyl)quinoline-1(2*H*)-carboxylate (1k): Yield: 47%; ^{1}H NMR (CDCl$_3$, 400 MHz) δ: 7.61 (d, J = 5.7 Hz, 1H), 7.27-7.23 (m, 1H), 7.17-7.07 (m, 5H),

6.97 (t, J = 7.7 Hz, 1H), 6.55 (d, J = 9.6 Hz, 1H), 6.37 (d, J = 5.8 Hz, 1H), 6.12 (dd, J = 9.6Hz, 6.0 Hz, 1H), 3.81 (s, 3H), 2.57 (s, 3H). ^{13}C NMR ($CDCl_3$, 100 MHz) δ: 155.8, 139.4, 136.1, 134.8, 131.2, 129.0, 128.5, 128.3, 127.6, 127.2, 126.9, 125.1, 125.0, 124.8, 54.4, 53.8, 20.1. TOF-HRMS Calcd. for $C_{18}H_{17}NO_2Na$ [M+Na$^+$]: 302.1151, found 302.1153. 99.5% ee; $[\alpha]_D^{25}$ = -842 (c = 1.0, $CHCl_3$); SFC condition: Lux 5u Cellulose-3 (250 × 4.60 mm), CO_2 : MeOH = 75:25, 3.0 mL/min, 230 nm; t_A = 1.7 min (major), t_B = 2.1 min (minor).

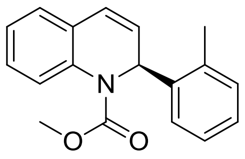

(*S*)-methyl 2-(m-tolyl)quinoline-1(2*H*)-carboxylate (1l): Yield: 43%; ^{1}H NMR ($CDCl_3$, 400 MHz) δ: 7.52 (s, 1H), 7.21-7.17 (m, 1H), 7.14-7.02 (m, 6H), 6.64 (d, J = 9.2 Hz, 1H), 6.22-6.15 (m, 2H), 3.85 (s, 3H), 2.28 (s, 3H). ^{13}C NMR ($CDCl_3$, 100 MHz) δ: 155.8, 140.1, 138.7, 135.2, 129.1, 129.0, 128.3, 127.7, 126.8, 125.7, 125.2, 124.8, 124.5, 56.2, 53.7, 22.0. TOF-HRMS Calcd. for $C_{18}H_{17}NO_2Na$ [M+Na$^+$]: 302.1151, found 302.1153. 99% ee; $[\alpha]_D^{25}$ = -784 (c = 1.0, $CHCl_3$); SFC condition: Lux 5u Cellulose-3 (250 × 4.60 mm), CO_2 : MeOH = 90:10, 3.0 mL/min, 210 nm; t_A = 3.4 min (major), t_B = 3.8 min (minor).

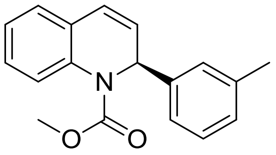

(*S*)-methyl 2-(3,5-dimethylphenyl)quinoline-1(2*H*)-carboxylate (1m): Yield: 44%; ^{1}H NMR ($CDCl_3$, 400 MHz) δ: 7.55 (s, 1H), 7.26-7.05 (m, 3H), 6.89 (d, J = 14.4 Hz, 3H), 6.64 (d, J = 9.3 Hz, 1H), 6.23-6.15 (m, 2H), 3.86 (s, 3H), 2.25 (s, 6H). ^{13}C NMR ($CDCl_3$, 100 MHz) δ: 155.9, 140.2, 138.6, 135.3, 130.0, 129.0, 128.2, 127.7, 126.9, 125.6, 125.3, 125.2, 124.8, 56.2, 53.7, 21.9. TOF-HRMS Calcd. for $C_{19}H_{20}NO_2$ [M+H$^+$]: 294.1488, found 294.1488. 99% ee; $[\alpha]_D^{25}$ = -810 (c = 1.0, $CHCl_3$); SFC condition: Lux 5u Cellulose-3 (250 × 4.60 mm), CO_2 : MeOH = 90:10, 2.5 mL/min, 230 nm; t_A = 2.0 min (major), t_B = 2.6 min (minor).

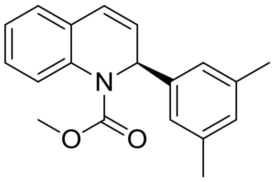

(*S*)-methyl 2-(3-chlorophenyl)quinoline-1(2*H*)-carboxylate (1n): Yield: 43%; ^{1}H NMR ($CDCl_3$, 400 MHz) δ: 7.51 (s, 1H), 7.28-7.05 (m, 7H), 6.70-6.66 (m, 1H), 6.18 (d, J = 5.6 Hz, 2H), 3.85 (s,

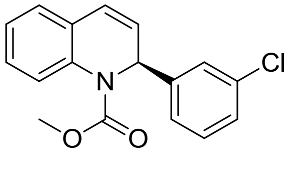

3H). ^{13}C NMR ($CDCl_3$, 100 MHz) δ: 155.7, 142.2, 134.9, 130.4, 128.5, 127.8, 127.4, 127.0, 126.5, 125.8, 125.1, 55.4, 53.9. TOF-HRMS Calcd. for $C_{17}H_{14}NO_2NaCl$ [$M+Na^+$]: 322.0605, found 322.0610. 99.9% ee; $[\alpha]_D^{25}$ = -836 (c = 1.0, $CHCl_3$); SFC condition: Lux 5u Cellulose-3 (250 × 4.60 mm), CO_2 : MeOH = 90:10, 3.0 mL/min, 210 nm; t_A = 2.8 min (major), t_B = 3.2 min (minor).

(*S*)-methyl 2-(4-chlorophenyl)quinoline-1(2*H*)-carboxylate (1o): Yield: 40%; ^{1}H

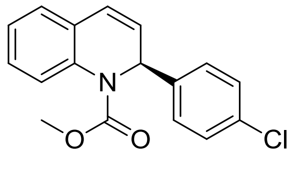

NMR (CDCl$_3$, 600 MHz) δ: 7.45-7.42 (m, 1H), 7.19-7.16 (m, 7H), 6.66 (d, J = 8.3 Hz, 1H), 6.17-6.15 (m, 2H), 3.83 (s, 3H). ^{13}C NMR ($CDCl_3$, 150 MHz) δ: 155.2, 138.0, 134.4, 133.7, 129.3, 128.8, 128.6, 128.0, 127.7, 127.0, 126.5, 125.9, 124.6, 54.9, 53.4. TOF-HRMS Calcd. for $C_{17}H_{14}NO_2NaCl$ [$M+Na^+$]: 322.0605, found 322.0610. 99% ee; $[\alpha]_D^{25}$ = -745 (c = 1.0, $CHCl_3$); SFC condition: Lux 5u Cellulose-3 (250 × 4.60 mm), CO_2 : MeOH = 90:10, 3.0 mL/min, 230 nm; t_A = 4.7 min (major), t_B = 8.4 min (minor).

(*S*)-methyl 6-methoxy-2-(4-methoxyphenyl)quinoline-1(2*H*)-carboxylate (1p):

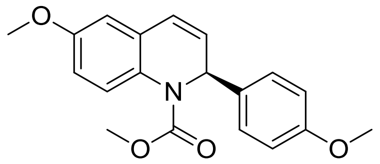

Yield: 45%; ^{1}H NMR ($CDCl_3$, 400 MHz) δ: 7.20 (d, J = 8.5 Hz, 3H), 6.78-6.60 (m, 5H), 6.21-6.17 (m, 2H), 3.83 (s, 3H), 3.77 (s, 3H), 3.71 (s, 3H). ^{13}C NMR ($CDCl_3$, 100 MHz) δ: 159.8, 156.8, 131.9, 129.8, 129.1, 128.8, 128.1, 126.4, 125.8, 114.7, 114.3, 113.7, 111.6, 55.9, 55.7, 55.6, 53.7. TOF-HRMS Calcd. for $C_{19}H_{20}NO_4$ [$M+H^+$]: 326.1386, found 326.1393. 99% ee; $[\alpha]_D^{25}$ = -695 (c = 1.0, $CHCl_3$); SFC condition: Lux 5u Cellulose-3 (250 × 4.60 mm), CO_2 : MeOH = 75:25, 3.0 mL/min, 210 nm; t_A = 2.2 min (major), t_B = 2.9 min (minor).

(*R*)-methyl 2-methylquinoline-1(2*H*)-carboxylate (1q): Yield: 41%; ^{1}H NMR

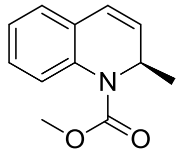

($CDCl_3$, 400 MHz) δ: 7.56 (s, 1H), 7.22-7.18 (m, 1H), 7.07-7.05 (m, 2H), 6.42 (d, J = 9.6 Hz, 1H), 6.02-5.98 (m, 1H), 5.14-5.07 (m, 1H), 3.80 (s, 3H), 1.11 (d, J = 6.8 H_Z, 3H). ^{13}C NMR ($CDCl_3$, 100 MHz) δ: 155.2, 134.7, 131.2, 128.0, 127.5, 126.7, 125.0, 124.7, 124.6, 53.5, 49.5, 19.1. TOF-HRMS Calcd. for $C_{12}H_{14}NO_2$ [$M+H^+$]: 204.1019, found 204.1025. 99% ee; $[\alpha]_D^{25}$ = -365 (c = 1.0, $CHCl_3$); SFC condition: Lux 5u Cellulose-3 (250 × 4.60 mm),

CO_2 : MeOH = 90:10, 2.5 mL/min, 210 nm; t_A = 2.6 min (major), t_B = 3.0 min (minor). [1]

(*R*)-methyl 2-butylquinoline-1(2H)-carboxylate (1r): Yield: 40%; ^{1}H NMR

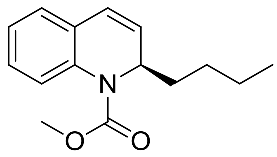

($CDCl_3$, 400 MHz) δ: 7.54 (s, 1H), 7.22-7.18 (m, 1H), 7.06 (d, *J* = 4.4 Hz, 2H), 6.44 (d, *J* = 9.6 Hz, 1H), 6.07-6.03 (m, 1H), 4.99 (d, *J* = 5.0 Hz, 1H), 3.78 (s, 3H), 1.43-1.25 (m, 6H), 0.85 (t, *J* = 7.2 Hz, 3H). ^{13}C NMR ($CDCl_3$, 100 MHz) δ: 155.6, 135.0, 130.8, 128.0, 127.8, 126.6, 125.4, 125.1, 124.8, 53.4, 53.3, 33.2, 27.9, 23.0, 14.5. TOF-HRMS Calcd. for $C_{15}H_{19}NO_2Na$ [M+Na$^+$]: 268.1308, found 268.1304. 99% ee; $[\alpha]_D^{25}$ = -427 (c = 1.0, $CHCl_3$); SFC condition: Lux 5u Cellulose-3 (250 × 4.60 mm), CO_2 : MeOH = 80:20, 3.0 mL/min, 230 nm; t_A = 2.9 min (major), t_B = 3.2 min (minor). [1]

1-methyl-1H-indene (1s): *n*-Butyllithium (41.3 mmol) was added dropwise to a

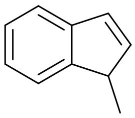

solution of indene (34.4 mmol) in 80 mL diethyl ether at -78 ℃. This was slowly allowed to warm to room temperature and stirred for 4 h in total. CH_3I (68.8 mmol) was then added dropwise to this orange-yellow solution at -78 ℃. This was slowly allowed to warm to room temperature and stirred for 4 h, and the reaction was terminated by the addition of 50 mL of a saturated aqueous NH_4Cl solution. The organic material was extracted into Et_2O, separated, dried ($MgSO_4$), filtered and the solvent removed at a rotary-evaporator. The residue was distilled at reduced pressure to afford a colorless oil **1s.** Yield: 68%; ^{1}H NMR ($CDCl_3$, 400 MHz) δ: 7.46-7.22 (m, 4H), 6.81 (dd, *J* = 5.5 Hz, 1.6 Hz, 1H), 6.51 (dd, *J* = 5.5 Hz, 1.6 Hz, 1H), 3.55-3.43 (m, 1H), 1.35 (d, *J* = 7.6 Hz, 3H). ^{13}C NMR ($CDCl_3$, 100 MHz) δ: 149.7, 144.5, 141.9, 130.7, 126.9, 125.3, 123.2, 121.6, 45.7, 16.7.[2]

(*E*)-but-1-ene-1,3-diyldibenzene (1t): A mixture of styrene (2 mmol),

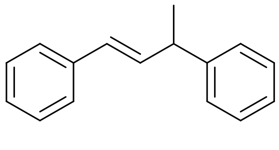

$Pd(OAc)_2$ (5.0 mol %) in CH_2Cl_2 (4 mL) in a round-bottomed flask was stirred at room temperature. Then, TFA (2 mmol) was added. The mixture was magnetically stirred at room temperature for 10 min until the complete consumption of styrene (observed by TLC). After the evaporation of the solvent under vacuum, the residue was purified by silica

gel chromatography to afford **1t.** Yield: 72%; ^{1}H NMR (CDCl$_3$, 400 MHz) δ: 7.58-7.44 (m, 8H), 7.43-7.38 (m, 2H), 6.67-6.58 (m, 2H), 3.88-3.82 (m, 1H), 1.69 (d, J = 7.0 Hz, 3H). ^{13}C NMR (CDCl$_3$, 100 MHz) δ: 146.4, 138.3, 135.9, 129.3, 129.2, 128.1, 127.8, 127.0, 126.9, 43.4, 22.0. [3]

(*2R,3R*)-methyl 2-(naphthalen-1-yl)-3-(4,4,5,5-tetramethyl-1,3,2-dioxaborolan-2-yl)-3,4-dihydroquinoline-1(2*H*)-carboxylate (2a): Yield: 45%; ^{1}H NMR (CDCl$_3$,

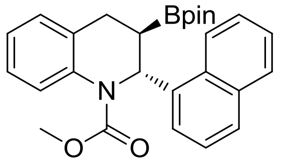

400 MHz) δ: 8.37 (d, J = 8.2 Hz, 1H), 7.85-7.70 (m, 3H), 7.53-7.45 (m, 2H), 7.35-7.09 (m, 5H), 6.37 (d, J = 9.1 Hz, 1H), 3.60 (s, 3H), 2.81-2.64 (m, 2H), 1.83-1.78 (m, 1H), 1.12 (s, 6H), 1.06 (s, 6H). ^{13}C NMR (CDCl$_3$, 100 MHz) δ: 155.8, 140.9, 138.4, 133.8, 133.7, 130.4, 128.7, 127.4, 127.3, 126.7, 125.6, 125.4, 125.3, 124.8, 124.5, 124.2, 123.9, 83.8, 57.5, 52.8, 28.9, 24.7, 24.6. TOF-HRMS Calcd. for $C_{27}H_{31}BNO_4$ [M+H$^+$]: 444.2346, found 444.2348. 97% ee; $[\alpha]_D^{25}$ = +74.2 (c = 1.0, CHCl$_3$); HPLC condition: Lux 5u Cellulose-1 (250 × 4.60 mm), ipa : hex = 10:90, 1.0 mL/min, 254 nm; t_A = 4.6 min (minor), t_B = 5.7 min (major).

(*2R,3R*)-phenyl 2-(naphthalen-1-yl)-3-(4,4,5,5-tetramethyl-1,3,2-dioxaborolan-2-yl)-3,4-dihydroquinoline-1(2*H*)-carboxylate (2b): Yield: 44%; ^{1}H NMR (CDCl$_3$,

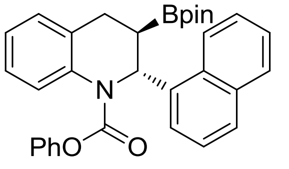

400 MHz) δ: 8.35 (s, 1H), 7.96-7.75 (m, 3H), 7.49-7.32 (m, 5H), 7.26-7.08 (m, 5H), 6.83 (d, J = 6.4 Hz, 2H), 6.56 (d, J = 8.3 Hz, 1H), 2.91-2.73 (m, 2H), 1.91 (t, J = 8.6 Hz, 1H), 1.15 (s, 6H), 1.09 (s, 6H). ^{13}C NMR (CDCl$_3$, 100 MHz) δ: 153.6, 151.2, 140.7, 138.1, 133.9, 130.4, 129.1, 128.8, 127.7, 127.5, 126.9, 125.8, 125.5, 125.4, 125.3, 124.6, 124.2, 121.8, 83.9, 28.9, 24.7, 22.8. TOF-HRMS Calcd. for $C_{32}H_{33}BNO_4$ [M+H$^+$]: 506.2503, found 506.2506. 97% ee; $[\alpha]_D^{25}$ = +85.4 (c = 1.0, CHCl$_3$); HPLC condition: Lux 5u Cellulose-1 (250 × 4.60 mm), ipa : hex = 10:90, 1.0 mL/min, 254 nm; t_A = 5.2 min (minor), t_B = 9.8 min (major).

(*2R,3R*)-benzyl 2-(naphthalen-1-yl)-3-(4,4,5,5-tetramethyl-1,3,2-dioxaborolan-2-yl)-3,4-dihydroquinoline-1(2*H*)-carboxylate (2c): Yield: 46%; ^{1}H NMR (CDCl$_3$, 400 MHz) δ: 8.29 (d, J = 8.1 Hz, 1H), 7.95-7.85 (m, 2H), 7.73 (d, J = 7.9 Hz, 1H), 7.49-7.42 (m, 2H), 7.35-7.26 (m, 3H), 7.20-7.09 (m, 5H), 6.91 (d, J = 7.2 Hz, 2H),

6.42 (d, J = 8.4 Hz, 1H), 5.04 (q, J = 12.5 Hz, 2H), 2.82-2.67 (m, 2H), 1.85-1.79 (m, 1H), 1.11 (s, 6H), 1.05 (s, 6H). ^{13}C NMR ($CDCl_3$, 100 MHz) δ: 155.5, 141.6, 138.8,

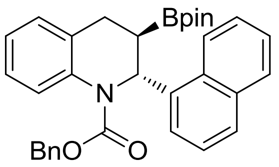

136.6, 134.3, 130.7, 129.2, 128.6, 128.2, 128.1, 127.9, 127.8, 127.2, 126.1, 125.9, 125.8, 124.8, 124.6, 124.5, 124.1, 84.3, 67.8, 58.0, 29.2, 25.1, 25.0. TOF-HRMS Calcd. for $C_{33}H_{35}BNO_4$ [M+H$^+$]: 520.2660, found 520.2664. 94% ee; $[\alpha]_D^{25}$ = +84.1 (c = 1.0, $CHCl_3$); HPLC condition: Lux 5u Cellulose-1 (250 × 4.60 mm), ipa : hex = 1:99, 1.0 mL/min, 254 nm; t_A = 11.4 min (minor), t_B = 13.0 min (major).

(2*R*,3*R*)-isopropyl 2-(naphthalen-1-yl)-3-(4,4,5,5-tetramethyl-1,3,2-dioxaborolan-2-yl)-3,4-dihydroquinoline-1(2*H*)-carboxylate (2d): Yield: 47%; ^{1}H NMR ($CDCl_3$,

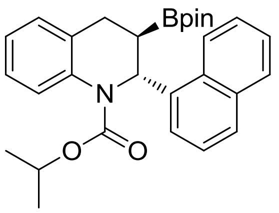

400 MHz) δ: 8.34 (d, J = 8.3 Hz, 1H), 7.87-7.81 (m, 2H), 7.68 (d, J = 8.0 Hz, 1H), 7.50-7.43 (m, 2H), 7.34-7.25 (m, 3H), 7.16 (d, J = 6.5 Hz, 1H), 7.08-7.05 (m, 1H), 6.26 (d, J = 9.3 Hz, 1H), 4.85-4.75 (m, 1H), 2.80-2.64 (m, 2H), 1.77-1.71 (m, 1H), 1.13 (d, J = 6.2 Hz, 3H), 1.10 (s, 6H), 1.02 (s, 6H), 0.63 (d, J = 5.9 Hz, 3H). ^{13}C NMR ($CDCl_3$, 100 MHz) δ: 155.2, 142.2, 139.1, 134.2, 133.9, 130.8, 129.1, 127.7, 127.6, 127.1, 125.9, 125.8, 124.9, 124.7, 124.6, 123.9, 84.3, 69.6, 57.8, 29.6, 25.2, 25.0, 22.5, 21.9. TOF-HRMS Calcd. for $C_{29}H_{35}BNO_4$ [M+H$^+$]: 472.2659, found 472.2663. 91% ee; $[\alpha]_D^{25}$ = +64.4 (c = 1.0, $CHCl_3$); HPLC condition: Lux 5u Cellulose-1 (250 × 4.60 mm), ipa : hex = 10:90, 1.0 mL/min, 254 nm; t_A = 3.9 min (minor), t_B = 4.6 min (major).

(2*R*,3*R*)-isobutyl 2-(naphthalen-1-yl)-3-(4,4,5,5-tetramethyl-1,3,2-dioxaborolan-2-yl)-3,4-dihydroquinoline-1(2*H*)-carboxylate (2e): Yield: 43%; ^{1}H NMR ($CDCl_3$,

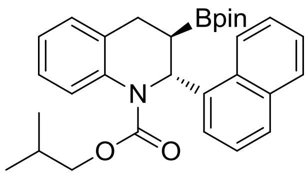

400 MHz) δ: 8.30 (d, J = 8.2 Hz, 1H), 7.84-7.79 (m, 2H), 7.68 (d, J = 8.0 Hz, 1H), 7.49-7.41 (m, 2H), 7.32-7.24 (m, 3H), 7.15-7.13 (m, 1H), 7.07-7.04 (m, 1H), 6.33 (d, J = 8.8 Hz, 1H), 3.79-3.71 (m, 2H), 2.77-2.60 (m, 2H), 1.78-1.73 (m, 1H), 1.66-1.59 (m, 1H), 1.09 (s, 6H), 1.03 (s, 6H), 0.64 (d, J = 6.7 Hz, 3H), 0.56 (d, J = 6.6 Hz, 3H). ^{13}C NMR ($CDCl_3$, 100 MHz) δ: 155.4, 141.3, 138.6, 133.8, 133.3, 130.3, 128.7, 127.3, 126.6, 125.4, 125.3, 124.6, 124.3, 124.1, 123.6,

83.8, 72.1, 57.3, 28.9, 27.7, 24.7, 24.6, 19.0, 18.9. TOF-HRMS Calcd. for $C_{30}H_{37}BNO_4$ [M+H$^+$]: 486.2816, found 486.2819. 93% ee; $[\alpha]_D^{25}$ = +71.1 (c = 1.0, $CHCl_3$); HPLC condition: Lux 5u Cellulose-1 (250 × 4.60 mm), ipa : hex = 10:90, 1.0 mL/min, 254 nm; t_A = 4.0 min (minor), t_B = 4.5 min (major).

(*2R*,*3R*)-methyl 6-methoxy-2-(naphthalen-1-yl)-3-(4,4,5,5-tetramethyl-1,3,2-dioxaborolan-2-yl)-3,4-dihydroquinoline-1(2*H*)-carboxylate (2f): Yield: 45%; ^{1}H NMR ($CDCl_3$, 400 MHz) δ: 8.35 (d, *J* = 7.8 Hz, 1H), 7.82 (d, *J* = 7.8 Hz, 1H), 7.70-7.63 (m, 2H), 7.51-7.43 (m, 2H), 7.34-7.25 (m, 2H), 6.86 (dd, *J* = 8.8 Hz, 2.6 Hz, 1H), 6.74 (d, *J* = 2.3 Hz, 1H), 6.33 (d, *J* = 9.3 Hz, 1H), 3.82 (s, 3H), 3.59 (s, 3H), 2.78-2.59 (m, 2H), 1.81-1.75 (m, 1H), 1.12 (s, 6H), 1.06 (s, 6H). ^{13}C NMR ($CDCl_3$, 100 MHz) δ: 155.8, 136.2, 133.8, 131.5, 130.4, 128.7, 128.5, 127.4, 126.0, 125.6, 125.4, 125.3, 124.7, 124.3, 121.8, 112.3, 112.1, 83.8, 55.5, 52.7, 29.7, 29.5, 26.9, 24.7. TOF-HRMS Calcd. for $C_{28}H_{33}BNO_5$ [M+H$^+$]: 474.2451, found 474.2454. 96% ee; $[\alpha]_D^{25}$ = +58.5 (c = 1.0, $CHCl_3$); Enantiomeric excess of the corresponding hydroxyl compound obtained by oxidation with $NaBO_3$ in THF/H_2O (1:1); HPLC condition: Lux 5u Cellulose-4 (250 × 4.60 mm), ipa : hex = 20:80, 1.0 mL/min, 254 nm; t_A = 16.5 min (minor), t_B = 17.8 min (major).

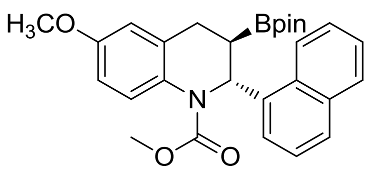

(*2R*,*3R*)-methyl 6-methyl-2-(naphthalen-1-yl)-3-(4,4,5,5-tetramethyl-1,3,2-dioxaborolan-2-yl)-3,4-dihydroquinoline-1(2*H*)-carboxylate (2g): Yield: 46%; ^{1}H NMR ($CDCl_3$, 400 MHz) δ: 8.35 (d, *J* = 7.7 Hz, 1H), 7.82 (d, *J* = 7.8 Hz, 1H), 7.69 (d, *J* = 8.0 Hz, 1H), 7.60 (s, 1H), 7.51-7.43 (m, 2H), 7.34-7.25 (m, 2H), 7.11 (d, *J* = 8.2 Hz, 1H), 7.00 (s, 1H), 6.32 (d, *J* = 9.2 Hz, 1H), 3.59 (s, 3H), 2.75-2.57 (m, 2H), 2.36 (s, 3H), 1.78-1.73 (m, 1H), 1.11 (s, 6H), 1.05 (s, 6H). ^{13}C NMR ($CDCl_3$, 100 MHz) δ: 156.3, 141.5, 136.3, 134.3, 133.9, 130.9, 129.2, 128.3, 127.9, 127.8, 126.1, 125.9, 125.8, 125.1, 124.8, 84.3, 53.3, 29.5, 27.5, 25.2, 25.1, 21.5. TOF-HRMS Calcd. for $C_{28}H_{33}BNO_4$ [M+H$^+$]: 458.2502, found 458.2507. 96% ee; $[\alpha]_D^{25}$ = +64.7 (c = 1.0, $CHCl_3$); Enantiomeric excess of the corresponding hydroxyl compound obtained by oxidation with $NaBO_3$ in THF/H_2O (1:1); HPLC condition: Lux 5u Cellulose-4 (250 ×

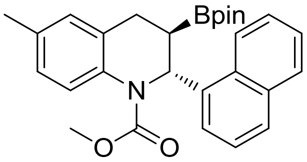

4.60 mm), ipa : hex = 30:70, 1.0 mL/min, 254 nm; t_A = 8.0 min (minor), t_B = 8.7 min (major).

(2*R*,3*R*)-methyl 7-methyl-2-(naphthalen-1-yl)-3-(4,4,5,5-tetramethyl-1,3,2-dioxaborolan-2-yl)-3,4-dihydroquinoline-1(2*H*)-carboxylate (2h): Yield: 47%; ^{1}H NMR

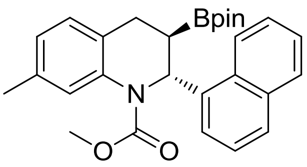

($CDCl_3$, 400 MHz) δ: 8.35 (d, J = 8.1 Hz, 1H), 7.82 (d, J = 7.9 Hz, 1H), 7.69 (d, J = 7.9 Hz, 1H), 7.58 (s, 1H), 7.51-7.43 (m, 2H), 7.32 (t, J = 7.4 Hz, 1H), 7.25 (t, J = 4.2 Hz, 1H), 7.04 (d, J = 7.6 Hz, 1H), 6.91 (d, J = 7.6 Hz, 1H), 6.32 (d, J = 9.2 Hz, 1H), 3.58 (s, 3H), 2.73-2.56 (m, 2H), 2.40 (s, 3H), 1.76-1.71 (m, 1H), 1.10 (s, 6H), 1.03 (s, 6H). ^{13}C NMR ($CDCl_3$, 100 MHz) δ: 156.3, 141.6, 138.6, 136.7, 134.2, 130.8, 129.2, 127.8, 127.5, 126.0, 125.9, 125.8, 125.7, 125.2, 124.9, 124.7, 84.3, 53.3, 29.0, 27.5, 25.2, 25.1, 22.1. TOF-HRMS Calcd. for $C_{28}H_{33}BNO_4$ [M+H$^+$]: 458.2502, found 458.2507. 95% ee; $[\alpha]_D^{25}$ = +80.2 (c = 1.0, $CHCl_3$); Enantiomeric excess of the corresponding hydroxyl compound obtained by oxidation with $NaBO_3$ in THF/H_2O (1:1); HPLC condition: Lux 5u Cellulose-4 (250 × 4.60 mm), ipa : hex = 30:70, 1.0 mL/min, 254 nm; t_A = 6.4 min (minor), t_B = 11.1 min (major).

(2*R*,3*R*)-methyl 2-(naphthalen-2-yl)-3-(4,4,5,5-tetramethyl-1,3-dioxolan-2-yl)-3,4-dihydroquinoline-1(2*H*)-carboxylate (2i): Yield: 45%; ^{1}H NMR ($CDCl_3$, 600

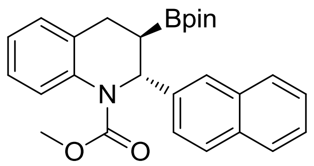

MHz) δ: 7.76-7.67 (m, 5H), 7.42-7.38 (m, 2H), 7.32-7.27 (m, 2H), 7.13 (d, J = 6.5 Hz, 1H), 7.09-7.07 (m, 1H), 5.67 (d, J = 10.3 Hz, 1H), 3.66 (s, 3H), 2.70-2.63 (m, 2H), 1.62-1.58 (m, 1H), 1.20 (d, J = 14.0 Hz, 12H). ^{13}C NMR ($CDCl_3$, 150 MHz) δ: 155.7, 142.3, 138.0, 134.9, 133.3, 132.6, 128.2, 127.9, 127.7, 126.9, 126.6, 125.9, 125.5, 125.2, 124.6, 124.2, 84.0, 61.1, 52.9, 32.2, 29.0, 25.1, 24.6. TOF-HRMS Calcd. for $C_{27}H_{31}BNO_4$ [M+H$^+$]: 444.2346, found 444.2348. 94% ee; $[\alpha]_D^{25}$ = +59.4 (c = 1.0, $CHCl_3$); HPLC condition: Enantiomeric excess of the corresponding hydroxyl compound obtained by oxidation with $NaBO_3$ in THF/H_2O (1:1); Lux 5u Cellulose-4 (250 × 4.60 mm), ipa : hex = 40:60, 1.0 mL/min, 254 nm; t_A = 5.3 min (minor), t_B = 8.4 min (major).

(2*R*,3*R*)-methyl 2-phenyl-3-(4,4,5,5-tetramethyl-1,3,2-dioxaborolan-2-yl)-3,4-dihydroquinoline-1(2*H*)-carboxylate (2j): Yield: 47%; ^{1}H NMR ($CDCl_3$, 400 MHz) δ:

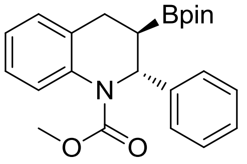

7.66 (d, J = 7.9 Hz, 1H), 7.28-7.05 (m, 8H), 5.56 (d, J = 9.9 Hz, 1H), 3.69 (s, 3H), 2.67-2.63 (m, 2H), 1.60-1.54 (m, 1H), 1.23 (d, J = 4.4 Hz, 12H). ^{13}C NMR ($CDCl_3$, 100 MHz) δ: 156.1, 145.2, 138.5, 135.2, 129.4, 128.7, 127.3, 127.2, 127.0, 126.9, 125.6, 124.5, 84.3, 61.3, 53.3, 29.3, 25.4, 25.1. TOF-HRMS Calcd. for $C_{23}H_{29}BNO_4$ [M+H$^+$]: 394.2188, found 394.2191. 94% ee; $[\alpha]_D^{25}$ = +54.9 (c = 1.0, $CHCl_3$); HPLC condition: Lux 5u Cellulose-1 (250 × 4.60 mm), ipa : hex = 10:90, 1.0 mL/min, 254 nm; t_A = 4.8 min (minor), t_B = 5.7 min (major).

(2*R*,3*R*)-methyl 3-(4,4,5,5-tetramethyl-1,3,2-dioxaborolan-2-yl)-2-(*o*-tolyl)-3,4-dihydroquinoline-1(2*H*)-carboxylate (2k): Yield: 45%; ^{1}H NMR ($CDCl_3$, 400 MHz) δ: 7.56 (d, J = 8.0 Hz, 1H), 7.28-7.24 (m, 1H), 7.16 (d, J = 6.6 Hz, 1H), 7.11-7.00 (m,

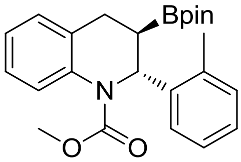

4H), 6.90 (d, J = 7.6 Hz, 1H), 5.69 (d, J = 10.3 Hz, 1H), 3.67 (s, 3H), 2.66-2.64 (m, 2H), 2.60 (s, 3H), 1.59-1.53 (m, 1H), 1.19 (d, J = 7.9 Hz, 12H). ^{13}C NMR ($CDCl_3$, 100 MHz) δ: 156.1, 143.5, 138.8, 135.9, 135.6, 130.8, 127.4, 127.2, 127.1, 127.0, 126.5, 125.8, 124.6, 84.3, 58.0, 53.2, 29.9, 27.5, 25.2, 19.9. TOF-HRMS Calcd. for $C_{24}H_{31}BNO_4$ [M+H$^+$]: 408.2345, found 408.2345. 99% ee; $[\alpha]_D^{25}$ = +78.4 (c = 1.0, $CHCl_3$); HPLC condition: Lux 5u Cellulose-1 (250 × 4.60 mm), ipa : hex = 10:90, 1.0 mL/min, 254 nm; t_A = 4.3 min (minor), t_B = 5.3 min (major).

(2*R*,3*R*)-methyl 3-(4,4,5,5-tetramethyl-1,3,2-dioxaborolan-2-yl)-2-(*m*-tolyl)-3,4-dihydroquinoline-1(2*H*)-carboxylate (2l): Yield: 46%; ^{1}H NMR ($CDCl_3$, 400 MHz)

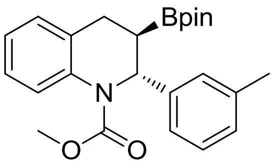

δ: 7.62 (d, J = 8.0 Hz, 1H), 7.27-7.22 (m, 1H), 7.12-6.96 (m, 6H), 5.47 (d, J = 10.1 Hz, 1H), 3.68 (s, 3H), 2.67-2.58 (m, 2H), 2.27 (s, 3H), 1.53-1.47 (m, 1H), 1.22 (d, J = 5.1 Hz, 12H). ^{13}C NMR ($CDCl_3$, 100 MHz) δ: 156.1, 145.2, 138.5, 138.1, 135.3, 128.7, 127.9, 127.6, 127.3, 126.9, 125.6, 124.5, 123.7, 84.3, 61.3, 53.3, 29.3, 25.4, 25.1, 22.0. TOF-HRMS Calcd. for $C_{24}H_{31}BNO_4$ [M+H$^+$]: 408.2345, found 408.2345. 93% ee; $[\alpha]_D^{25}$ = +65.8 (c = 1.0, $CHCl_3$); HPLC condition: Lux 5u

Cellulose-1 (250 × 4.60 mm), ipa : hex = 10:90, 1.0 mL/min, 254 nm; t_A = 4.3 min (minor), t_B = 5.1 min (major).

(2*R*,3*R*)-methyl 3-(4,4,5,5-tetramethyl-1,3,2-dioxaborolan-2-yl)-2-(*m*-tolyl)-3,4-dihydroquinoline-1(2*H*)-carboxylate (2m): Yield: 47%; ^{1}H NMR (CDCl$_3$, 400 MHz)

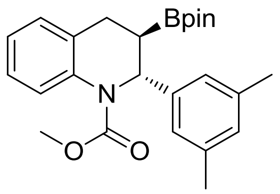

δ: 7.63 (d, *J* = 8.0 Hz, 1H), 7.27-7.23 (m, 1H), 7.12-7.04 (m, 2H), 6.81 (d, *J* = 16.2 Hz, 3H), 5.44 (d, *J* = 10.2 Hz, 1H), 3.69 (s, 3H), 2.63-2.60 (m, 2H), 2.22 (s, 6H), 1.51-1.44 (m, 1H), 1.23 (d, *J* = 6.0 Hz, 12H). ^{13}C NMR (CDCl$_3$, 100 MHz) *δ*: 156.1, 145.3, 138.6, 138.0, 135.4, 128.8, 127.2, 126.9, 125.6, 124.4, 84.3, 61.4, 53.3, 30.2, 29.4, 25.4, 25.0, 21.9. TOF-HRMS Calcd. for $C_{25}H_{33}BNO_4$ [M+H$^+$]: 422.2502, found 422.2500. 92% ee; $[\alpha]_D^{25}$ = +58.6 (c = 1.0, CHCl$_3$); HPLC condition: Lux 5u Cellulose-1 (250 × 4.60 mm), ipa : hex = 10:90, 1.0 mL/min, 254 nm; t_A = 4.1 min (minor), t_B = 4.8 min (major).

(2*R*,3*R*)-methyl 2-(3-chlorophenyl)-3-(4,4,5,5-tetramethyl-1,3,2-dioxaborolan-2-yl)-3,4-dihydroquinoline-1(2*H*)-carboxylate (2n): Yield: 44%; ^{1}H NMR (CDCl$_3$,

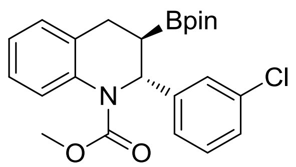

600 MHz) *δ*: 7.60 (d, *J* = 6.2 Hz, 1H), 7.26-7.25 (m, 2H), 7.17-7.06 (m, 5H), 5.44 (d, *J* = 10.5 Hz, 1H), 3.69 (s, 3H), 2.62 (d, *J* = 7.5 Hz, 2H), 1.45-1.41 (m, 1H), 1.23 (d, *J* = 7.0 Hz, 12H). ^{13}C NMR (CDCl$_3$, 150 MHz) *δ*: 155.6, 147.1, 137.7, 135.0, 134.0, 129.7, 127.0, 126.9, 126.8, 126.7, 125.2, 124.4, 84.1, 60.7, 53.0, 32.4, 29.0, 25.1, 24.6. TOF-HRMS Calcd. for $C_{23}H_{27}BClNO_4Na$ [M+Na$^+$]: 450.1618, found 450.1620. 92% ee; $[\alpha]_D^{25}$ = +69.2 (c = 1.0, CHCl$_3$); SFC condition: Lux 5u Cellulose-1 (250 × 4.60 mm), CO_2 : MeOH = 90:10, 3.0 mL/min, 254 nm; t_A = 3.5 min (minor), t_B = 4.0 min (major).

(2*R*,3*R*)-methyl 2-(4-chlorophenyl)-3-(4,4,5,5-tetramethyl-1,3,2-dioxaborolan-2-yl)-3,4-dihydroquinoline-1(2*H*)-carboxylate (2o): Yield: 43%; ^{1}H NMR (CDCl$_3$,

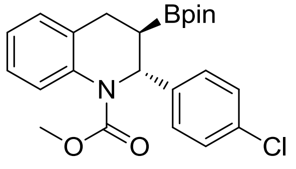

400 MHz) *δ*: 7.58 (d, *J* = 7.6 Hz, 1H), 7.27-7.05 (m, 7H), 5.46 (d, *J* = 10.3 Hz, 1H), 3.68 (s, 3H), 2.63 (d, *J* = 7.5 Hz, 2H), 1.50-1.45 (m, 1H), 1.22 (s, 12H). ^{13}C NMR (CDCl$_3$, 100 MHz) *δ*: 156.0, 143.9, 138.1, 135.4, 132.9, 128.9, 128.4, 127.3, 127.1, 125.6,

124.8, 84.5, 60.9, 53.4, 29.4, 27.5, 25.5, 25.1. TOF-HRMS Calcd. for $C_{23}H_{27}BClNO_4Na$ [M+Na$^+$]: 450.1618, found 450.1620. 90% ee; $[\alpha]_D^{25}$ = +55.8 (c = 1.0, $CHCl_3$); SFC condition: Lux 5u Cellulose-1 (250 × 4.60 mm), CO_2 : MeOH = 90:10, 2.5 mL/min, 210 nm; t_A = 4.5 min (minor), t_B = 5.2 min (major).

(*2R,3R*)-methyl 6-methoxy-2-(4-methoxyphenyl)-3-(4,4,5,5-tetramethyl-1,3,2-dioxaborolan-2-yl)-3,4-dihydroquinoline-1(2*H*)-carboxylate (2p): Yield: 46%; ^{1}H NMR (CDCl$_3$, 600 MHz) δ: 7.45 (d, J = 0.9 Hz, 1H), 7.13 (d, J = 8.7 Hz, 2H), 6.77-6.67 (m, 4H), 5.43 (d, J = 10.2 Hz, 1H), 3.79 (s, 3H), 3.74 (s, 3H), 3.67 (s, 3H), 2.62-2.55 (m, 2H), 1.50-1.46 (m, 1H), 1.21 (d, J = 3.6 Hz, 12H). ^{13}C NMR (CDCl$_3$, 150 MHz) δ: 158.3, 156.1, 155.7, 136.8, 136.6, 131.0, 127.7, 126.2, 113.6, 112.0, 111.8, 83.8, 60.1, 55.5, 55.2, 52.8, 32.1, 29.2, 25.0, 24.6. TOF-HRMS Calcd. for $C_{25}H_{32}BNO_6Na$ [M+Na$^+$]: 476.2220, found 476.2221. 93% ee; $[\alpha]_D^{25}$ = +60.8 (c = 1.0, $CHCl_3$); Enantiomeric excess of the corresponding hydroxyl compound obtained by oxidation with $NaBO_3$ in THF/H_2O (1:1); HPLC condition: Lux 5u Cellulose-4 (250 × 4.60 mm), ipa : hex = 20:80, 1.0 mL/min, 254 nm; t_A = 6.6 min (minor), t_B = 7.6 min (major).

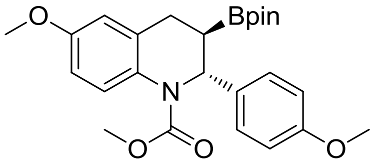

(*2S,3R*)-methyl 2-methyl-3-(4,4,5,5-tetramethyl-1,3,2-dioxaborolan-2-yl)-3,4-dihydroquinoline-1(2*H*)-carboxylate (2q): Yield: 48%; ^{1}H NMR (CDCl$_3$, 400 MHz) δ: 7.40 (d, J = 8.0 Hz, 1H), 7.16-6.98 (m, 3H), 4.57-4.53 (m, 1H), 3.74 (s, 3H), 2.65-2.48 (m, 2H), 1.23 (s, 12H), 1.21 (s, 3H), 1.19-1.05 (m, 1H). ^{13}C NMR (CDCl$_3$, 100 MHz) δ: 155.9, 137.2, 135.6, 127.1, 126.5, 126.1, 124.6, 84.1, 53.1, 28.7, 25.6, 25.3, 25.2, 22.0. TOF-HRMS Calcd. for $C_{18}H_{27}BNO_4$ [M+H$^+$]: 332.2031, found 332.2034. 90% ee; $[\alpha]_D^{25}$ = +59.2 (c = 1.0, $CHCl_3$); HPLC condition: Lux 5u Cellulose-1 (250 × 4.60 mm), ipa : hex = 3:97, 1.0 mL/min, 254 nm; t_A = 5.9 min (minor), t_B = 6.4 min (major).

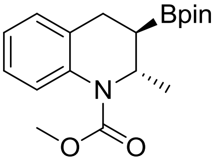

(*2S,3R*)-methyl 2-butyl-3-(4,4,5,5-tetramethyl-1,3,2-dioxaborolan-2-yl)-3,4-dihydroquinoline-1(2*H*)-carboxylate (2r): Yield: 47%; ^{1}H NMR (CDCl$_3$, 600 MHz) δ: 7.35 (s, 1H), 7.07-6.93 (m, 3H), 4.55 (q, J = 6.83 Hz, 1H), 3.67 (s, 3H), 2.63-2.55 (m, 2H), 1.49-1.43 (m, 1H), 1.18-1.13 (m, 18H), 0.76 (t, J = 6.9 Hz, 3H). ^{13}C NMR

(CDCl$_3$, 150 MHz) δ: 155.6, 137.1, 127.3, 126.0, 125.5, 124.1, 83.6, 55.2, 52.6, 34.8, 28.1, 27.2, 24.8, 24.7, 22.8, 14.2. TOF-HRMS Calcd. for $C_{21}H_{33}BNO_4$ [M+H$^+$]: 374.2501, found 374.2504. 93% ee; $[\alpha]_D^{25}$ = +61.5 (c = 1.0, CHCl$_3$); Enantiomeric excess of the corresponding hydroxyl compound obtained by oxidation with NaBO$_3$ in THF/H$_2$O (1:1); HPLC condition: Lux 5u Cellulose-4 (250 × 4.60 mm), ipa : hex = 30:70, 1.0 mL/min, 254 nm; t_A = 6.7 min (minor), t_B = 8.0 min (major).

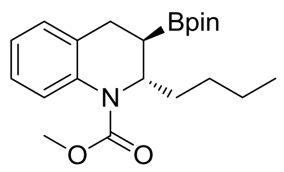

3. Procedure for the synthesis of 3-7

Procedure for the synthesis of 3 [4]

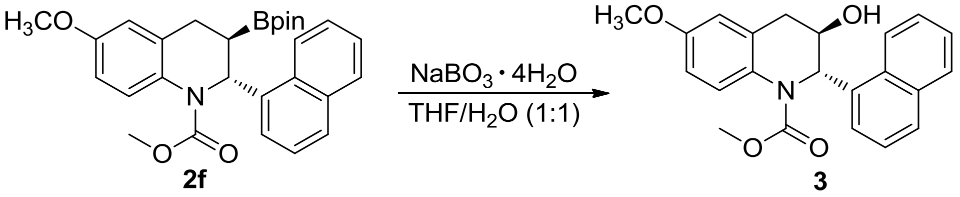

In a round bottom flask, **2f** (0.2 mmol) was dissolved in THF/H$_2$O (1:1, 4 mL). NaBO$_3$•4H$_2$O (1.0 mmol) was added at room temperature. After stirred for 2 h, the reaction mixture was extracted with EtOAc, dried over Na$_2$SO$_4$, and filtered. The residue was purified by silica gel chromatography to afford **3**.

(*2S,3R*)- methyl 3-hydroxy-6-methoxy-2-(naphthalen-1-yl)-3,4-dihydroquinoline-1(2*H*)-carboxylate (3): Yield: 94%; ^{1}H NMR (CDCl$_3$, 600 MHz) δ: 8.20 (d, J = 7.9 Hz, 1H), 7.82-7.81 (m, 2H), 7.70 (d, J = 8.1 Hz, 1H), 7.47-7.43 (m, 2H), 7.29-7.20 (m, 2H), 6.85 (dd, J = 9.0 Hz, 2.9 Hz, 1H), 6.63 (d, J = 2.7 Hz, 1H), 6.09 (d, J = 5.4 Hz, 1H), 4.19 (s, 1H), 3.78 (s, 3H), 3.58 (m, 3H), 2.76-2.67 (m, 2H), 2.57 (s, 1H),. ^{13}C NMR (CDCl$_3$, 150 MHz) δ: 156.3, 156.1, 137.3, 133.8, 130.9, 130.8, 129.0, 128.1, 127.8, 126.4, 125.8, 125.6, 124.9, 123.7, 123.4, 114.0, 113.0, 71.5, 61.5, 55.6, 53.2, 34.4. TOF-HRMS Calcd. for $C_{22}H_{22}NO_4$ [M+H$^+$]: 364.1543, found 362.1551. 96% ee; $[\alpha]_D^{25}$ = -21.6 (c = 1.0, CHCl$_3$); HPLC condition: Lux 5u Cellulose-4 (250 × 4.60 mm), ipa : hex = 20:80, 1.0 mL/min, 254 nm; t_A = 16.5 min (minor), t_B = 17.8 min (major).

Procedure for the synthesis of 4 [4, 5]

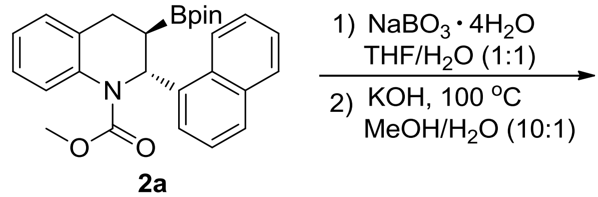

2a **4**

In a round bottom flask, **2a** (0.2 mmol) was dissolved in THF/H_2O (1:1, 4 mL). $NaBO_3$•$4H_2O$ (1.0 mmol) was added at room temperature. After stirred for 2 h, the reaction mixture was extracted with EtOAc, dried over Na_2SO_4, and filtered. The resulting crude material was used in the next reaction without further purification. To a solution of this intermediate in a mixture of MeOH and H_2O (1:1, 5.5 mL) was added KOH (9.0 mmol) at rt. The resulting mixture was heated to 100 ^{o}C. After the reaction was completed, and the reaction mixture was extracted with DCM, dried over Na_2SO_4 and evaporated under reduced pressure. The residue was purified by silica gel chromatography to afford **4**.

(*2S,3R*)-2-(naphthalen-1-yl)-1,2,3,4-tetrahydroquinolin-3-ol (4): Yield: 89%; ^{1}H NMR ($CDCl_3$, 400 MHz) δ: 8.24 (d, J = 8.2 Hz, 1H), 7.91-7.79 (m, 2H), 7.62-7.41 (m, 4H), 7.13-7.04 (m, 2H), 6.75-6.65 (m, 2H), 5.21 (d, J = 4.7 Hz, 1H), 4.45 (t, J = 5.2 Hz, 1H), 4.27 (s, 1H), 2.91-2.79 (m, 2H), 2.03 (d, J = 7.3 Hz, 1H), 1.57 (s, 1H). ^{13}C NMR ($CDCl_3$, 100 MHz) δ: 143.9, 137.9, 134.4, 131.3, 131.1, 129.6, 129.0, 128.1, 127.0, 126.4, 126.2, 125.0, 123.5, 118.3, 117.7, 113.8, 68.3, 59.0, 33.3. TOF-HRMS Calcd. for $C_{19}H_{18}NO$ [M+H^{+}]: 276.1382, found 276.1385. 97% ee; $[\alpha]_D^{25}$ = +18.5 (c = 1.0, $CHCl_3$); SFC condition: Lux 5u Cellulose-1 (250 × 4.60 mm), CO_2 : MeOH = 90:10, 2.5 mL/min, 230 nm; t_A = 3.5 min (minor), t_B = 3.7 min (major).

Procedure for the synthesis of 5 [6]

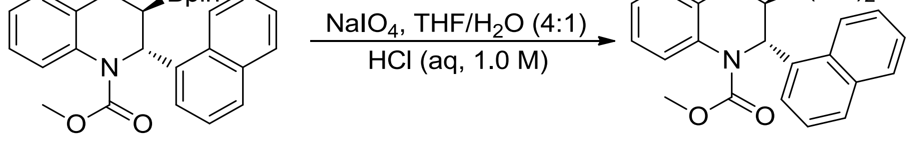

2a **5**

In a round bottom flask, **2a** (0.5 mmol) was dissolved in a THF and H_2O (4:1, 2 mL). $NaIO_4$ (0.75 mmol) was then added at rt, and the suspension was stirred for 15 min.

HCl (aq, 1.0 M, 0.50 mL) was added. After the completion of the reaction, the reaction mixture was extracted with EtOAc. The combined organic layers were washed with H_2O and brine, dried with Na_2SO_4, filtered, and concentrated under vacuum to give a white solid **5**.

(*2R,3R*) -(1-(methoxycarbonyl)-2-(naphthalen-1-yl)-1,2,3,4-tetrahydroquinolin-3-yl) boronic acid (5): Yield: 80%; $[\alpha]_D^{25}$ = +43.2 (c = 1.0, $CHCl_3$). ^{1}H NMR ($CDCl_3$, 400 MHz) δ: 8.24 (d, J = 8.2 Hz, 1H), 8.01-7.74 (m, 3H), 7.57-7.49 (m, 2H), 7.36-7.23 (m, 4H), 7.16-7.13 (m, 2H), 6.15 (d, J = 5.3 Hz, 1H), 4.30 (s, 1H), 3.64 (s, 3H), 2.83 (d, J = 5.1 Hz, 2H), 2.07 (s, 1H). ^{13}C NMR ($CDCl_3$, 100 MHz) δ: 155.9, 137.8, 137.3, 133.9, 130.7, 129.4, 129.1, 128.2, 127.4, 126.5, 125.8, 125.6, 124.3, 123.6, 123.6, 123.5, 123.2, 71.2, 61.6, 53.3, 34.1. TOF-HRMS Calcd. for $C_{21}H_{21}BNO_4$ [M+H$^+$]: 362.1562, found 362.1559.

Procedure for the synthesis of 6 [7]

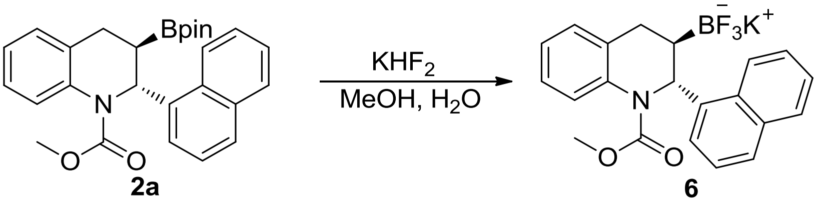

2a **6**

In a round bottom flask, **2a** (0.5 mmol) was dissolved in MeOH (2 mL), An aqueous solution of KHF_2 (4.5M, 2.5 mmol) was added to the flask. The solution was stirred at rt for 4 h. After the evaporation of the solvent under vacuum, the residual pinacol was removed by adding three portions of Et_2O, retiring the resulting solution. The solid that was obtained was triturated with acetone and filtered through a plug of Celite. The acetone solution was evaporated to yield a white solid **6**.

(*2R,3R*)-potassium (1-(methoxycarbonyl)-2-(naphthalen-1-yl)-1,2,3,4-tetrahydro-quinolin-3-yl) trifluoroborate (6) Yield: 83%; $[\alpha]_D^{25}$ = -21.6 (c = 1.0, CH_3COCH_3). ^{1}H NMR (CD_3CN, 600 MHz) δ: 8.42 (d, J = 8.2 Hz, 1H), 7.78 (d, J = 7.9 Hz, 1H), 7.62 (d, J = 8.0 Hz, 1H), 7.46-7.02 (m, 8H), 6.13 (d, J = 9.1 Hz, 1H), 3.44 (s, 3H), 2.61-2.41 (m, 2H), 0.95 (s, 1H). ^{13}C NMR (CD_3CN, 150 MHz) δ: 155.7, 144.9, 139.0, 138.4, 133.4, 131.6, 128.1, 126.5, 126.2, 125.4, 125.3, 125.2, 125.1, 124.8, 124.2, 123.9, 58.2, 51.8, 38.4, 29.7. TOF-HRMS Calcd. for $C_{21}H_{18}BF_3NO_2$ [M-K$^+$]:

384.1392, found 384.1396.

Procedure for the synthesis of 7[8]

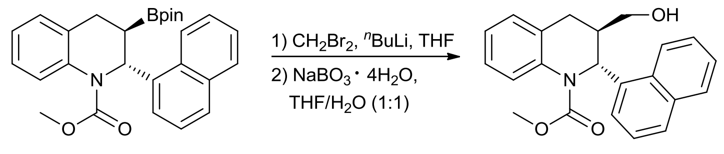

2a **7**

To a solution of **2a** (0.113 mmol) and dibromomethane (19.4 μL, 0.283 mmol) in THF (1.2 mL) was added dropwise *n*-BuLi (0.25 mmol, 2.4 M solution in hexane) at -78 ℃ under an nitrogen atmosphere. The resulting mixture was stirred for 10 min at -78 ℃ and then warmed to rt and allowed to stir for 2 h. The reaction was quenched with a saturated aqueous solution of NH_4Cl, extracted with EtOAc. The resulting organic layer was dried over $MgSO_4$, filtered and concentrated in vacuo. The resulting intermediate was dissolved in THF/H_2O (1:1, 4 mL). $NaBO_3$•$4H_2O$ (0.565 mmol) was added at room temperature. After stirred for 2 h, the reaction mixture was extracted with EtOAc, dried over Na_2SO_4, and filtered. The residue was purified by silica gel chromatography to afford **7.**

(2*R*,3*R*)-methyl 3-(hydroxymethyl)-2-(naphthalen-1-yl)-3,4-dihydroquinoline-1(2*H*)-carboxylate (7) Yield: 66%; ^{1}H NMR ($CDCl_3$, 400 MHz) δ 8.26 (d, *J* = 8.4 Hz, 1H), 7.97-7.65 (m, 3H), 7.50-7.40 (m, 2H), 7.39-7.02 (m, 5H), 6.23 (d, *J* = 7.5 Hz, 1H), 3.72-3.50 (m, 5H), 2.73-2.66 (m, 2H), 2.45-2.19 (m, 1H), 1.93 (s, 1H). ^{13}C NMR ($CDCl_3$, 100 MHz) *δ*: 156.0, 139.5, 138.1, 133.8, 131.0, 130.9, 129.0, 128.2, 127.8, 126.9, 126.3, 125.7, 125.5, 124.4, 123.3, 63.7, 55.6, 53.2, 46.1, 29.3. 97% ee; TOF-HRMS Calcd. for $C_{22}H_{22}NO_3$ [M+H$^+$]: 348.1594, found 348.1598. $[\alpha]_D^{25}$ = +36.4 (c = 1.0, $CHCl_3$); HPLC condition: Lux 5u Cellulose-4 (250 × 4.60 mm), ipa : hex = 20:80, 1.0 mL/min, 254 nm; t_A = 6.3 min (minor), t_B = 7.5 min (major).

Procedure for the synthesis of 8[9]

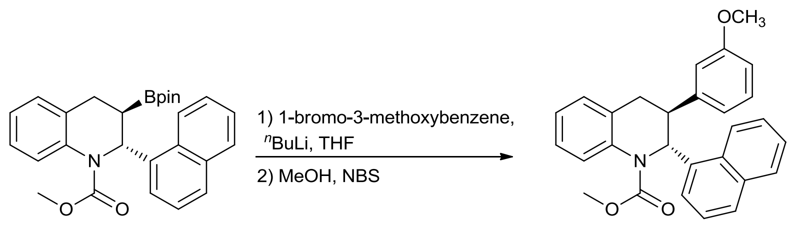

2a **8**

To a solution of 1-bromo-3-methoxybenzene (17.3 µL, 0.137 mmol) in THF (0.5 mL) was added dropwise ^{n}BuLi (0.137 mmol, 2.4 M solution in hexane) at -78 ℃ under an nitrogen atmosphere. After stirred for 1 h at -78 ℃, a THF solution (0.5 mL) of **2a** (0.113 mmol) was added. After stirred for 1 h at -78 ℃, the solvent was removed under reduced pressure and MeOH (1.2 ml) was added to the mixture. A MeOH solution (1.8 mL) of NBS (0.208 mmol) was then added at -78 ℃ and the reaction stirred for 1 h at -78 ℃. Aqueous $Na_2S_2O_3$ was added and the reaction mixture was allowed to warm to rt. The mixture was extracted with CH_2Cl_2, dried over $MgSO_4$, and filtered. The residue was purified by silica gel chromatography to afford **8.**

(*2R,3S*)-methyl 3-(3-methoxyphenyl)-2-(naphthalen-1-yl)-3,4-dihydroquinoline-1 (2*H*)-carboxylate (8) Yield: 53%; ^{1}H NMR ($CDCl_3$, 400 MHz) δ 7.76-7.57 (m, 4H), 7.40-7.01 (m, 10H), 6.75-6.53 (m, 2H), 6.39 (d, *J* = 1.7 Hz, 1H), 6.14-5.96 (m, 1H), 3.56 (s, 3H), 3.50 (s, 3H), 3.29-3.06 (m, 2H), 2.97-2.77 (m, 1H). ^{13}C NMR ($CDCl_3$, 100 MHz) *δ*: 159.6, 155.6, 143.3, 140.1, 138.3, 133.4, 133.0, 131.1, 129.5, 128.5, 127.7, 127.5, 127.1, 125.7, 125.3, 125.2, 125.1, 124.7, 124.3, 123.2, 119.9, 113.6, 112.7, 62.3, 55.1, 53.0, 52.3, 34.0. TOF-HRMS Calcd. for $C_{28}H_{26}NO_3$ [M+H$^+$]: 424.1907, found 424.1911. 95% ee; $[\alpha]_D^{25}$ = +29.4 (c = 1.0, $CHCl_3$); HPLC condition: Lux 5u Cellulose-1 (250 × 4.60 mm), ipa : hex = 10:90, 1.0 mL/min, 254 nm; t_A = 11.7 min (minor), t_B = 13.1 min (major).

Procedure for the synthesis of 9

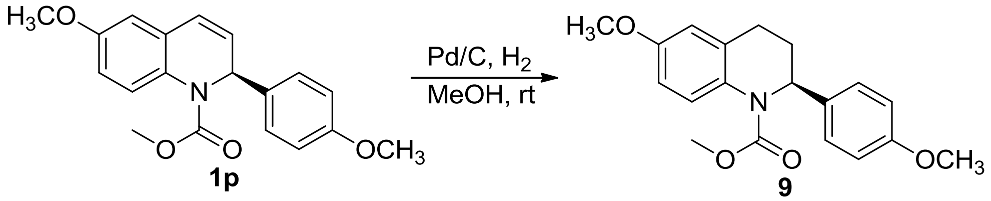

A 25 mL round bottom flask was charged with compound **1p** (0.5 mmol), MeOH (5 mL), and 10% Pd/C (10 mg). The reaction vessel was purged with hydrogen three times and then the mixture was stirred under atmospheric hydrogen (using a hydrogen balloon) at room temperature until no starting material was detected by TLC. The catalyst was filtered and the filtrate was concentrated under reduced pressure. The residue was purified via flash chromatography to afford **9** as yellow oil**.**

(*S*)-methyl 6-methoxy-2-(4-methoxyphenyl)-3,4-dihydroquinoline-1(2*H*)-carboxylate (9): Yield: 92%; ^{1}H NMR ($CDCl_3$, 400 MHz) δ: 7.55 (s, 1H), 7.14 (d, *J* = 8.3 Hz, 2H), 6.81 (d, *J* = 8.3 Hz, 3H), 6.66 (m, 1H), 5.42 (t, *J* = 7.4 Hz, 1H), 3.79 (s, 3H), 3.75 (s, 3H), 3.71 (s, 3H), 2.69-2.55 (m, 2H), 2.51-2.45 (m, 1H), 1.91-1.82 (m, 21H). ^{13}C NMR ($CDCl_3$, 100 MHz) δ: 158.9, 156.6, 156.2, 135.9, 135.2, 131.2, 127.8, 126.7, 114.3, 113.1, 112.4, 58.1, 55.9, 55.8, 53.5, 33.7, 27.0. TOF-HRMS Calcd. for $C_{19}H_{22}NO_4$ [M+H$^+$]: 328.1543, found 328.1546. 99% ee; $[\alpha]_D^{25}$ = -544 (c = 1.0, $CHCl_3$); SFC condition: Lux 5u Cellulose-3 (250 × 4.60 mm), CO_2 : MeOH = 75:25, 3.0 mL/min, 210 nm; t_A = 4.5 min (minor), t_B = 5.2 min (major).

4. Deuterium Labeling Experiment

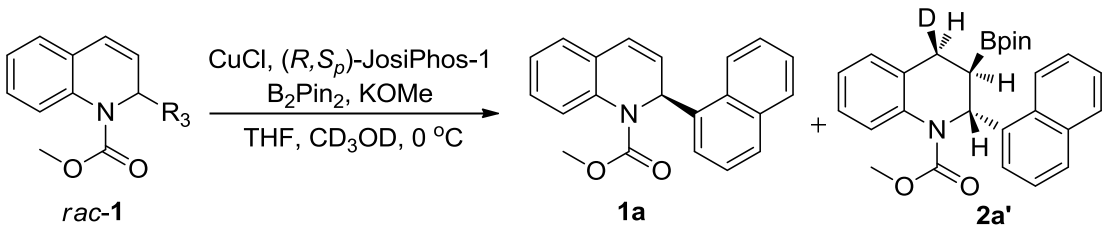

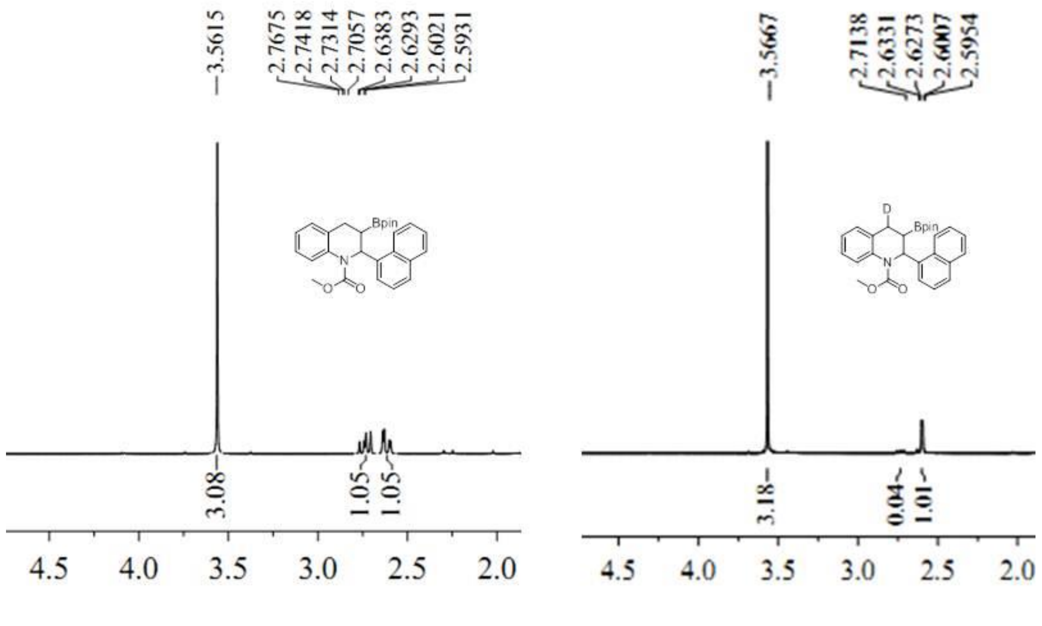

The borylation of *rac*-**1a** under the optimized conditions using CD_3OD instead of MeOH gave product **2a′**, bearing a deuterium label at its 4-position (> 95% D), with high enantioselectivity (97% ee).

Deuterium Labeling **2a′**: ^{1}H NMR ($CDCl_3$, 400 MHz) δ: 8.32 (d, *J* = 7.6 Hz, 1H), 7.80 (d, *J* = 8.2 Hz, 1H), 7.74 (d, *J* = 5.8 Hz, 1H), 7.67 (d, *J* = 8.1 Hz, 1H), 7.48-7.42 (m, 2H), 7.31-7.27 (m, 2H), 7.22 (d, *J* = 6.7 Hz, 1H), 7.16-7.14 (m, 1H), 7.09-7.07 (m, 1H), 6.33 (d, *J* = 8.9 Hz, 1H), 3.57 (s, 3H), 2.63-2.59 (m, 1H), 1.76 (dd, *J* = 9.0 Hz, 3.1 Hz, 1H), 1.09 (s, 6H), 1.03 (s, 6H).

5. X-ray Crystallography

Single-crystal X-ray diffraction measurements were carried out on a Rigaku Saturn CCD diffractometer at 100(2) K using graphite monochromated Cu Kα radiation (λ = 1.54184 Å). An empirical absorption correction was applied using the SADABS program.[10] All structures were solved by direct methods and refined by full-matrix least squares on F^2 using the SHELXL program package.[11] All the hydrogen atoms were geometrically fixed using the riding model. The crystal data and experimental data for **1b, 2a and 2p** are summarized in **Table S1**.

References

[1] F. Amiot, L. Cointeaux, E. J. Silve and A. Alexakis, *Tetrahedron* 2004, **60**, 8221.

[2] N. E. Grimmer, N. J. Coville, C. B. de Koning, J. M. Smith, L. M. Cook, *J. Organomet. Chem.* 2000, **616**, 112.

[3] H. Ma, Q. Sun, W. Li, J. Wang, Z. Zhang, Y. Yang, Z. Lei, *Tetrahedron lett.* 2011, **52**, 1569.

[4] K. Kubota, K. Hayama, H. Iwamoto and H. Ito, *Angew. Chem., Int. Ed.* 2015, **54**, 8809.

[5] J. Yang, H. Wu, L. Shen and Y. Qin, *J. Am. Chem. Soc.* 2007, **129**, 13794.

[6] C. W. Liskey and J. F. Hartwig, *J. Am. Chem. Soc.* 2013, **135**, 3375.

[7] M. Guisan-Ceinos, A. Parra, V. Martin-Heras and M. Tortosa, *Angew. Chem., Int. Ed.* 2016, **55**, 6969.

[8] M. V. Joannou, B. S. Moyer, S. J. Meek, *J. Am. Chem. Soc.* 2015, **137**, 6176.

[9] A. Bonet, M. Odachowski, D. Leonori, S. Essafi and V. K. Aggarwal, *Nat. Chem.* 2014, **6**, 584.

[10] G. M. Sheldrick, SADABS, Program for Empirical Absorption Correction of Area Detector Data, University of Göttingen, Göttingen, Germany, 1996.

[11] G. M. Sheldrick, *Acta Cryst.*, 2008, **A64**, 112-122.

Crystal parameters

Table S1. Crystal Data and Experimental Parameters for Compounds 1b, 2a and 2p

Compound	**1b**	**2a**	**2p**
Formula	$C_{26}H_{19}NO_2$	$C_{27}H_{30}NBO_4$	$C_{25}H_{32}NBO_6$
Fw	377.42	443.33	453.32
crystal system	orthorhombic	orthorhombic	orthorhombic
space group	$P2_12_12_1$	$P2_12_12_1$	$P2_12_12_1$
a (Å)	7.657(1)	8.521(1)	11.094(1)
b (Å)	9.515(1)	15.612(2)	14.666(1)
c (Å)	26.125(1)	17.520(1)	14.750(1)
α (deg)	90	90	90
β (deg)	90	90	90
γ (deg)	90	90	90
V (Å^3)	1903.42(5)	2330.58(5)	2399.83(8)
Z	4	4	4
D_{calc} (g/cm^3)	1.317	1.263	1.255
μ(Mo/Kα)$_{calc}$ (cm^{-1})	0.658	0.665	0.718
size (mm)	0.30 × 0.25 × 0.15	0.30 × 0.25 × 0.20	0.30 × 0.10 × 0.05
F(000)	792	944	968
2θ range (deg)	9.89 to 146.69	7.58 to 146.73	8.50 to 146.56
no. of reflns, collected	5060	9699	7673
no of obsd reflns	3309	4390	4326
no of variables	262	303	305
abscorr (T_{max}, T_{min})	1.00, 0.84	1.00, 0.68	1.00, 0.70
R	0.029	0.032	0.035
R_w	0.07	0.080	0.083
R_{all}	0.030	0.032	0.039
Absolute structure parameter	0.13(11)	-0.03(7)	0.01(12)
Gof	1.09	1.06	1.06
CCDC	1542782	1542784	1542783

5. NMR spectra of all compounds

(*S*)-methyl 2-(naphthalen-1-yl)quinoline-1(2*H*)-carboxylate (1a):

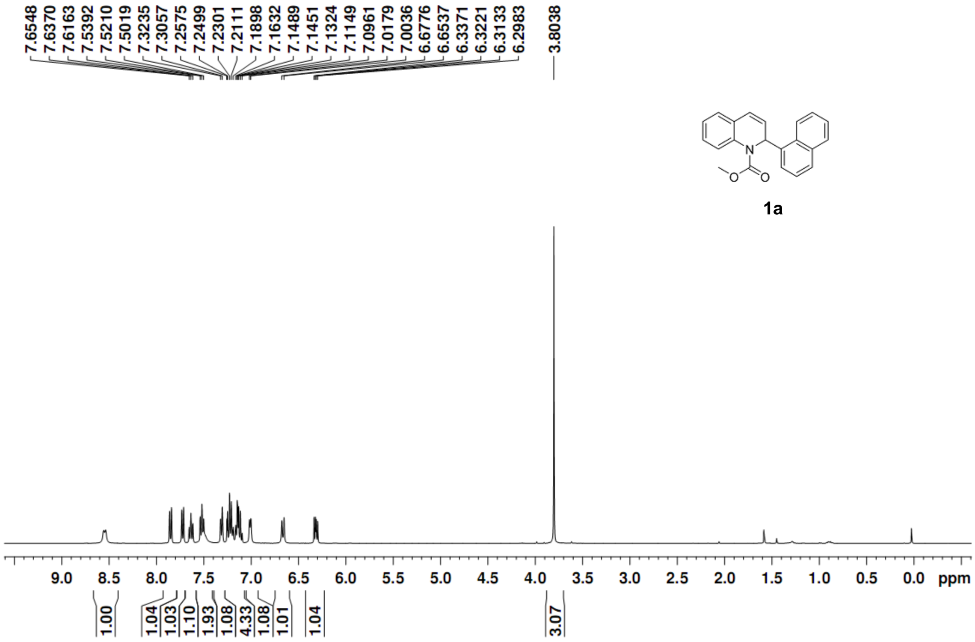

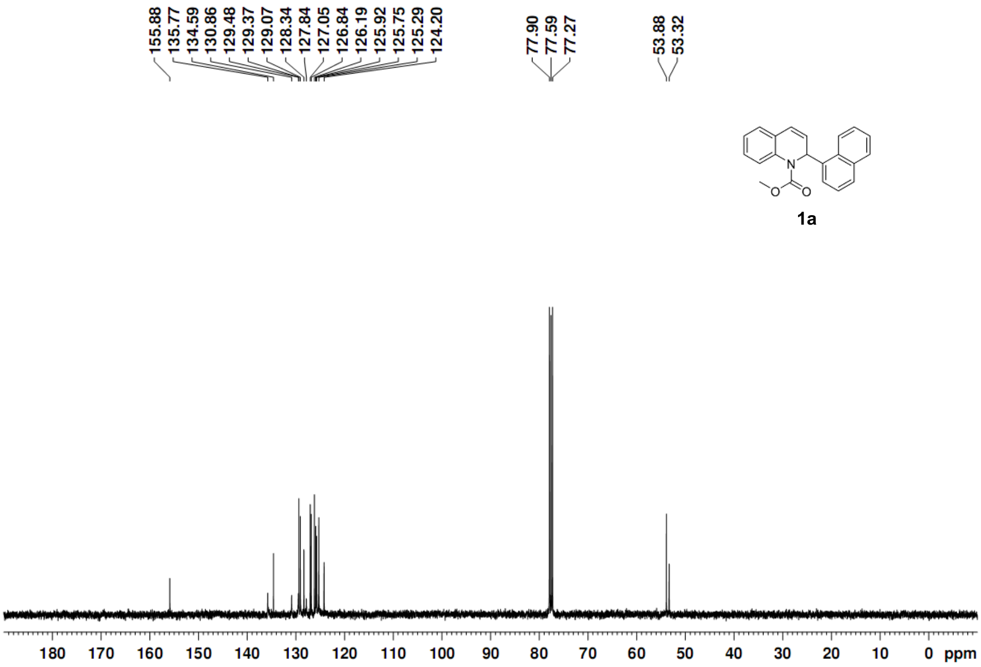

(*S*)-phenyl 2-(naphthalen-1-yl)quinoline-1(2*H*)-carboxylate (1b):

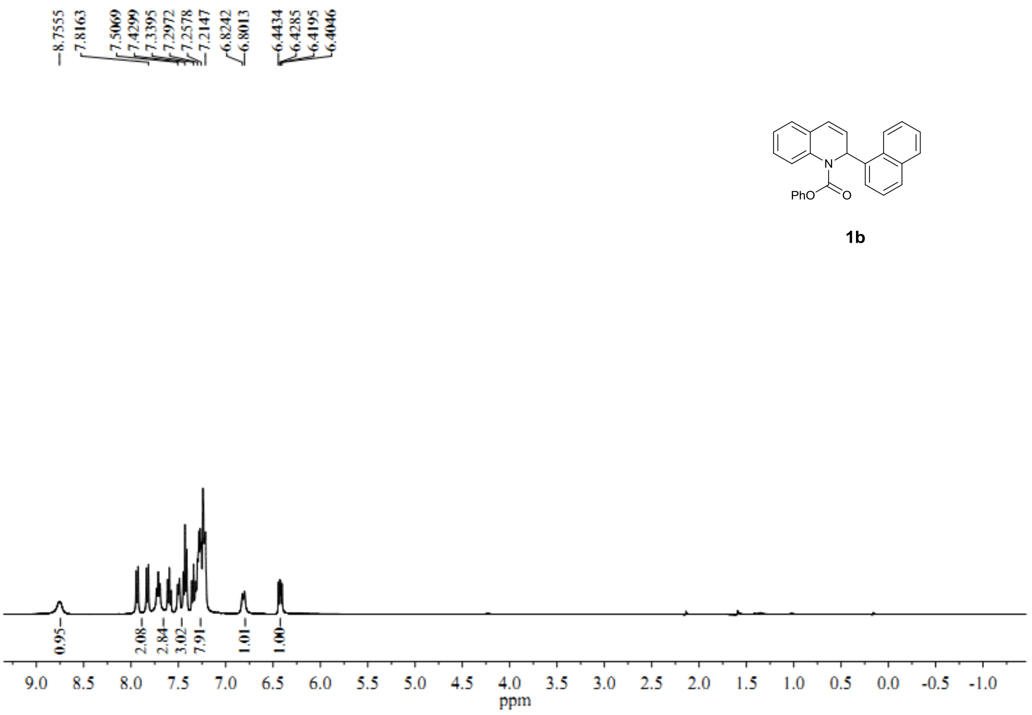

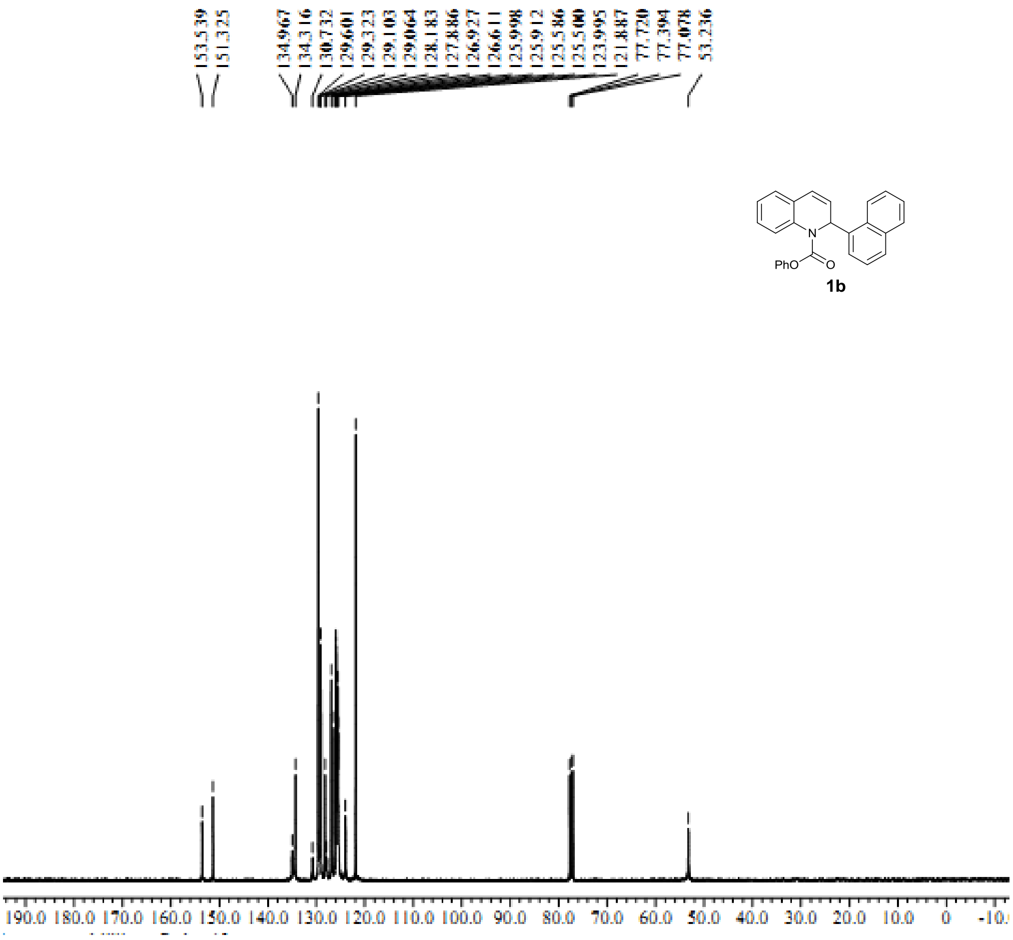

(*S*)-benzyl 2-(naphthalen-1-yl)quinoline-1(2*H*)-carboxylate (1c):

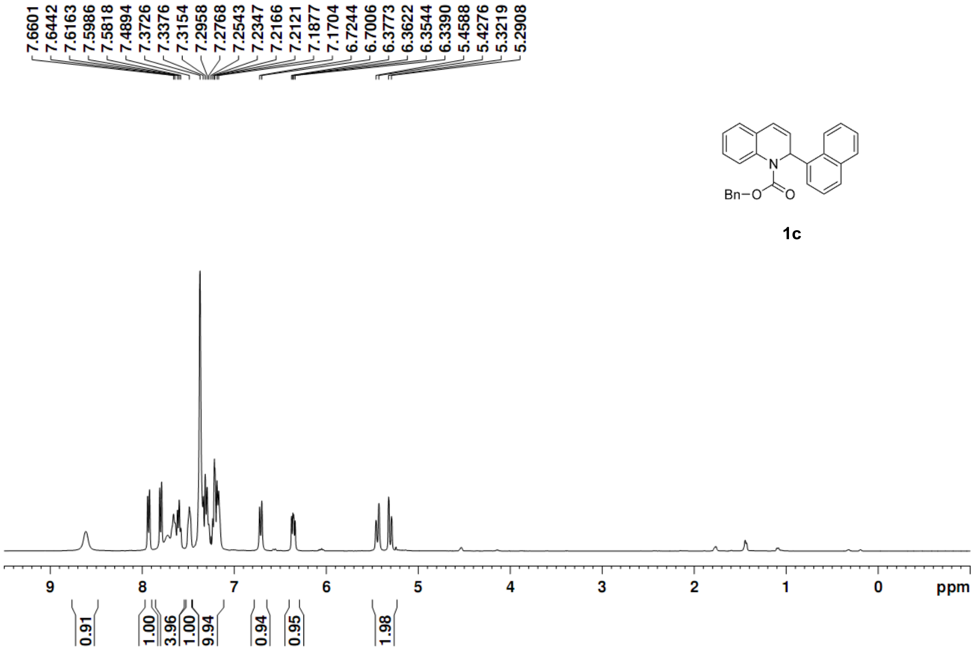

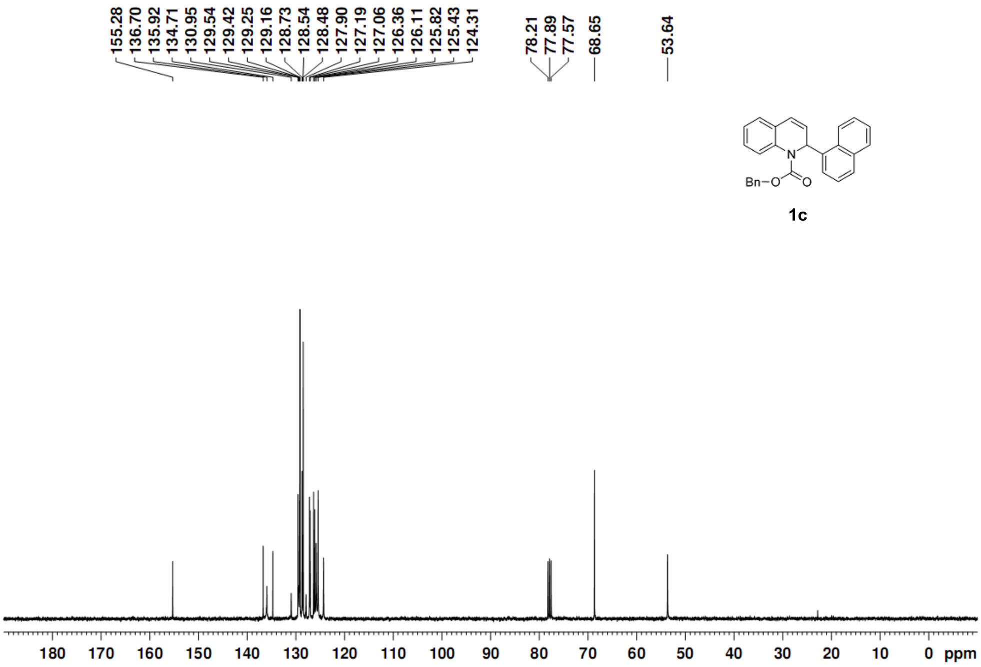

(*S*)-isopropyl 2-(naphthalen-1-yl)quinoline-1(2*H*)-carboxylate (1d):

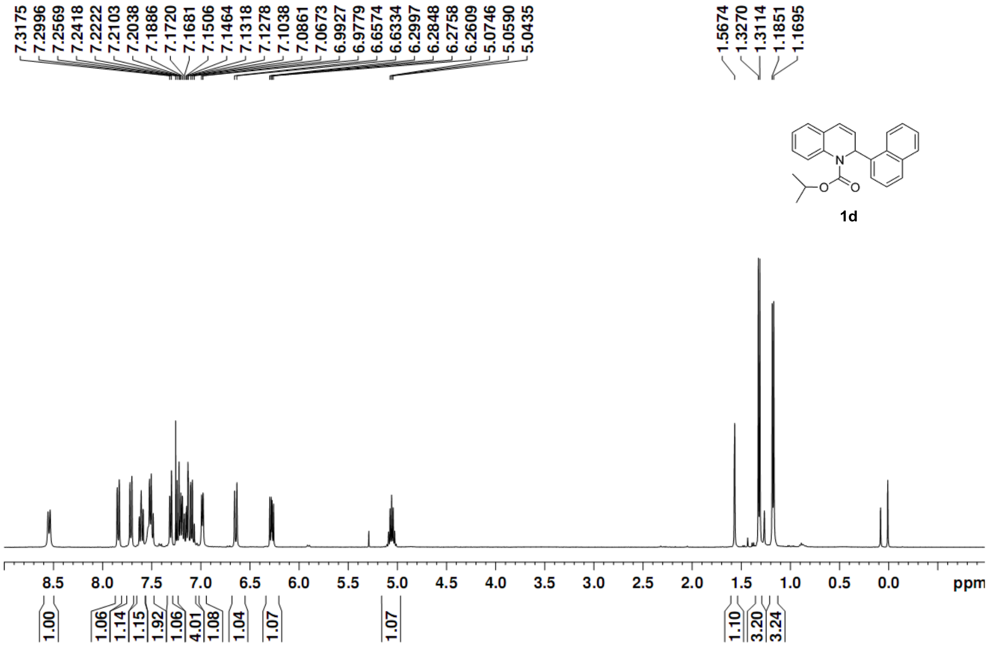

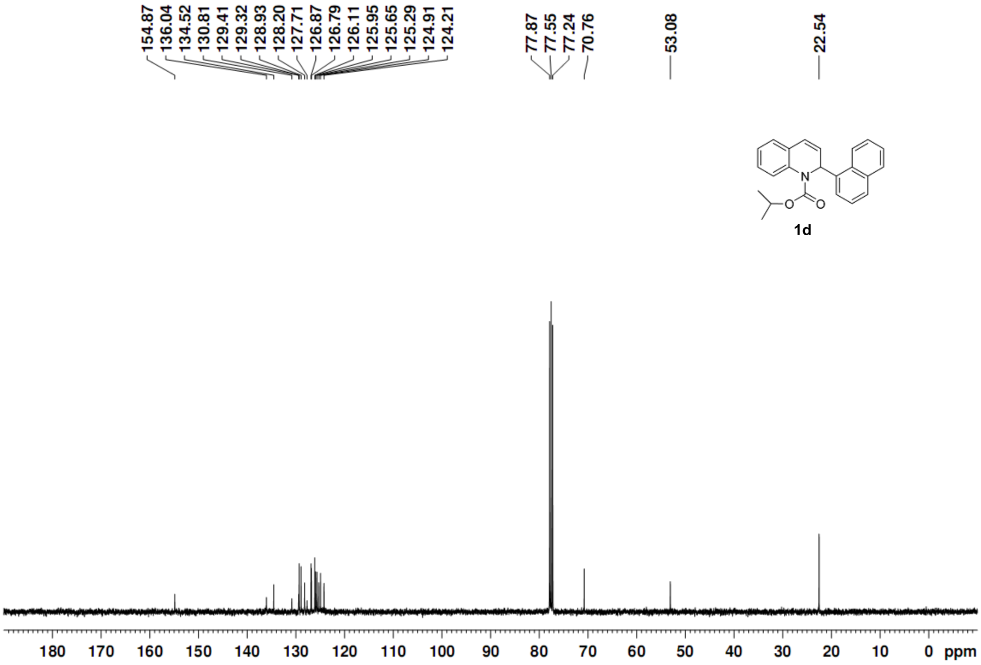

(*S*)-isobutyl 2-(naphthalen-1-yl)quinoline-1(2*H*)-carboxylate (1e):

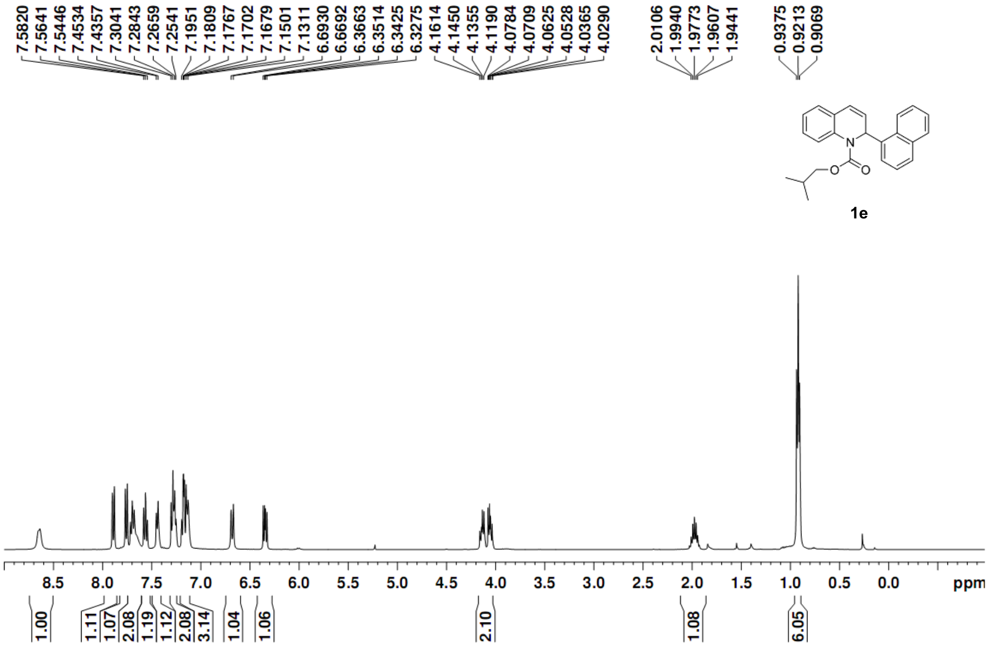

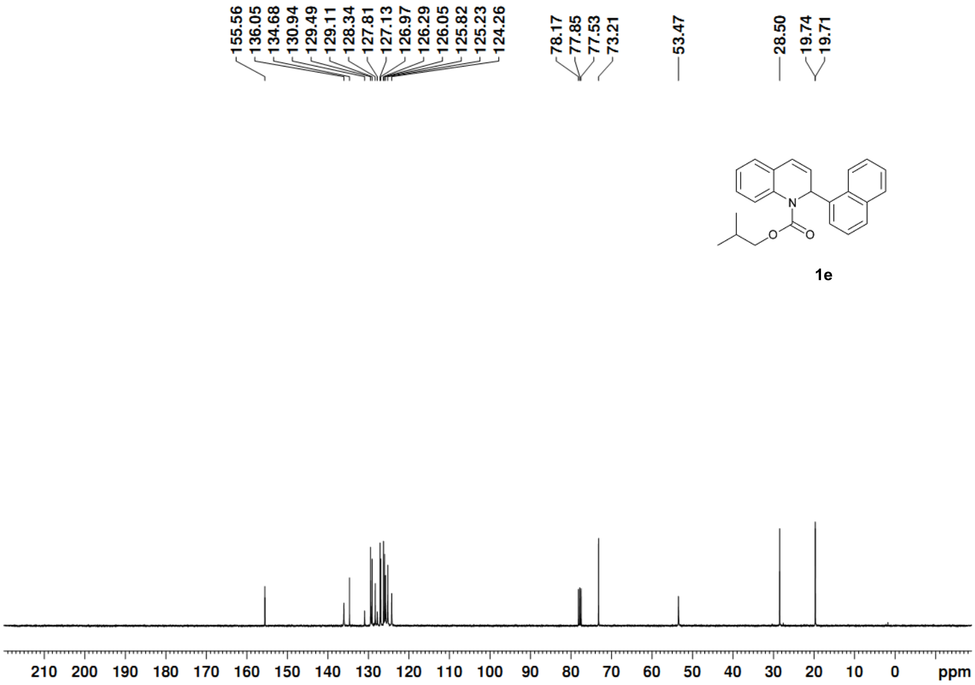

(*S*)-methyl 6-methoxy-2-(naphthalen-1-yl)quinoline-1(2*H*)-carboxylate (1f):

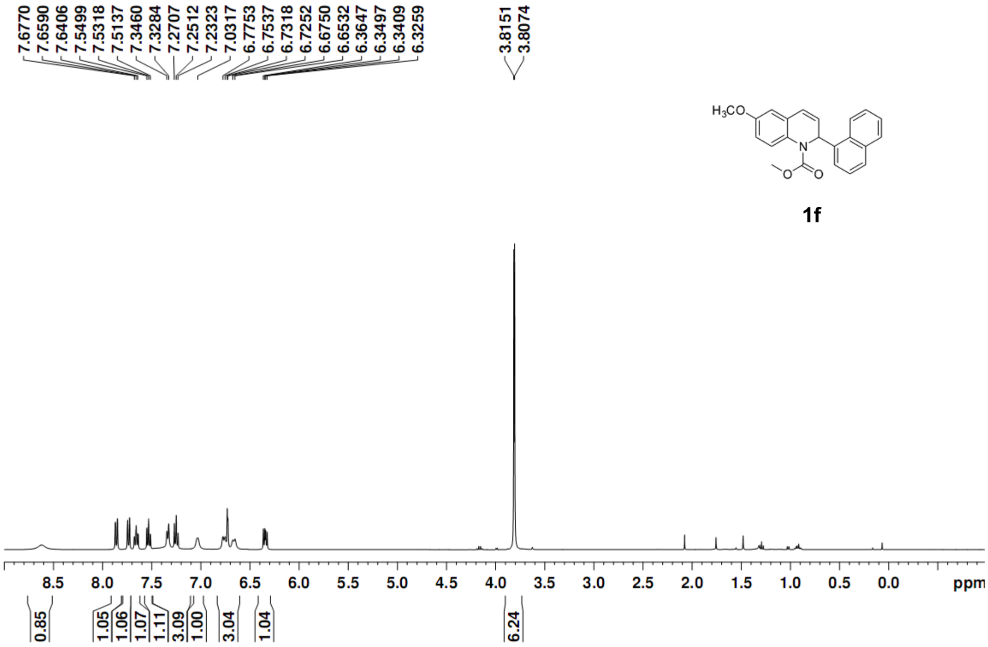

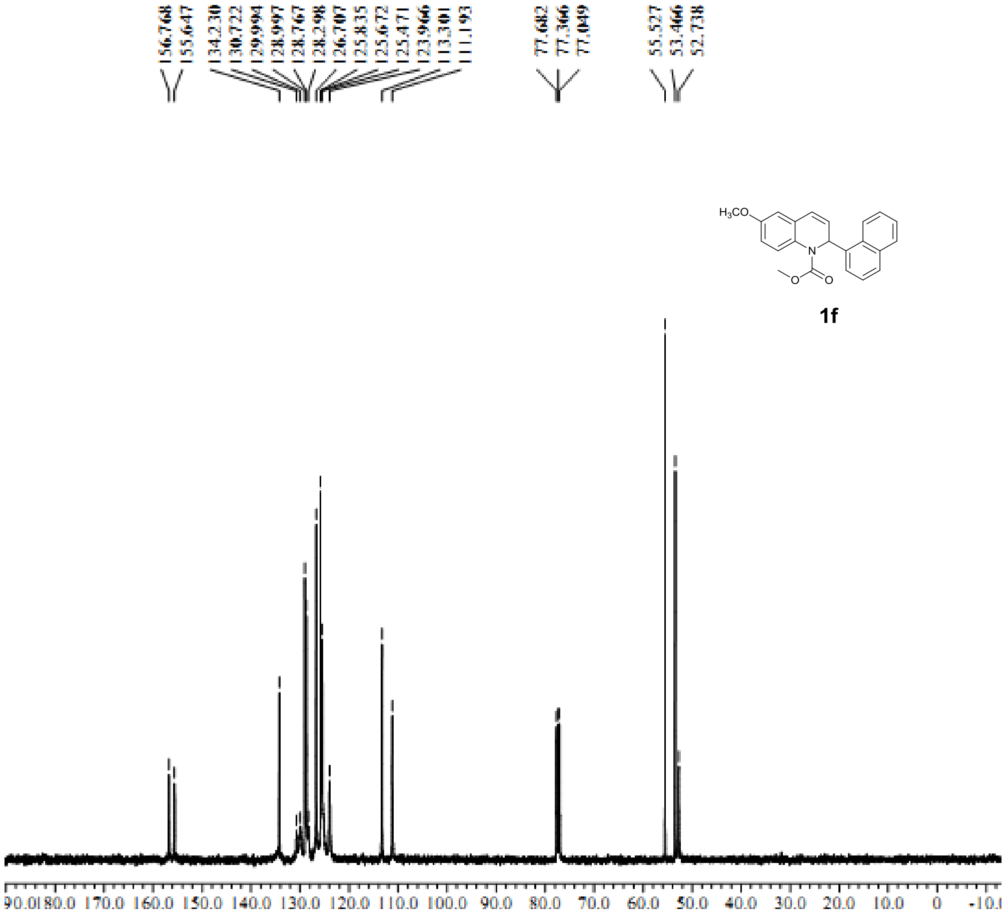

(*S*)-methyl 6-methyl-2-(naphthalen-1-yl)quinoline-1(2*H*)-carboxylate (1g):

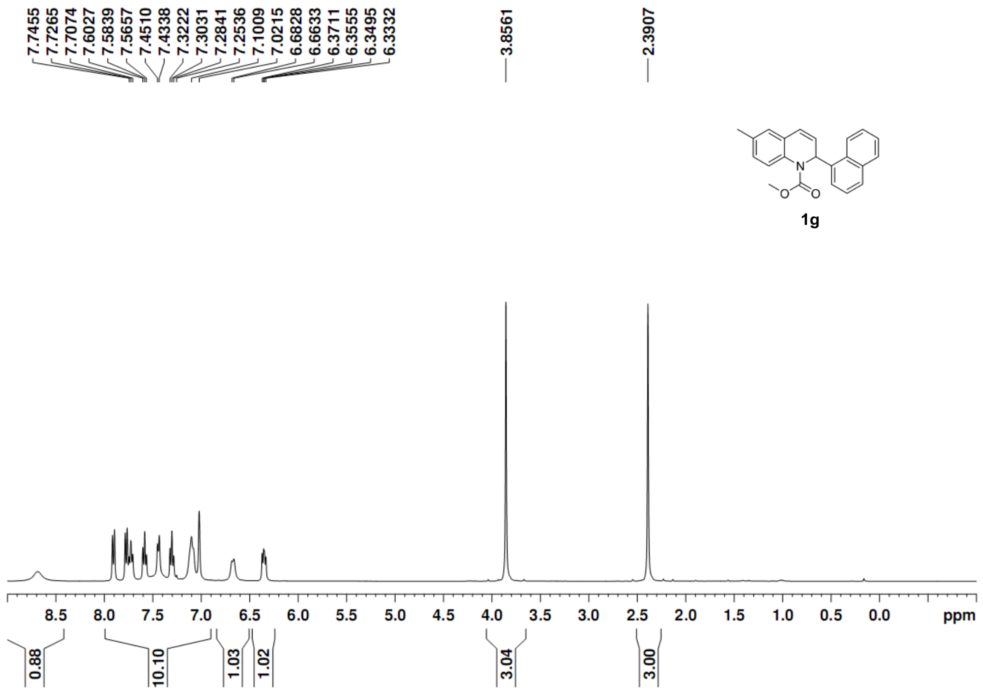

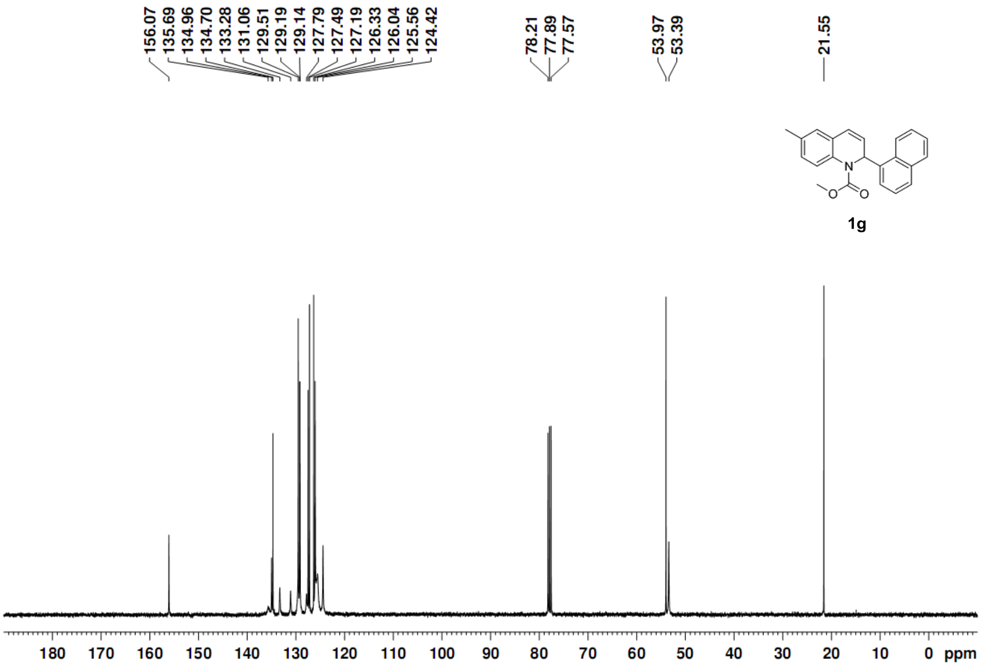

(*S*)-methyl 7-methyl-2-(naphthalen-1-yl)quinoline-1(2*H*)-carboxylate (1h):

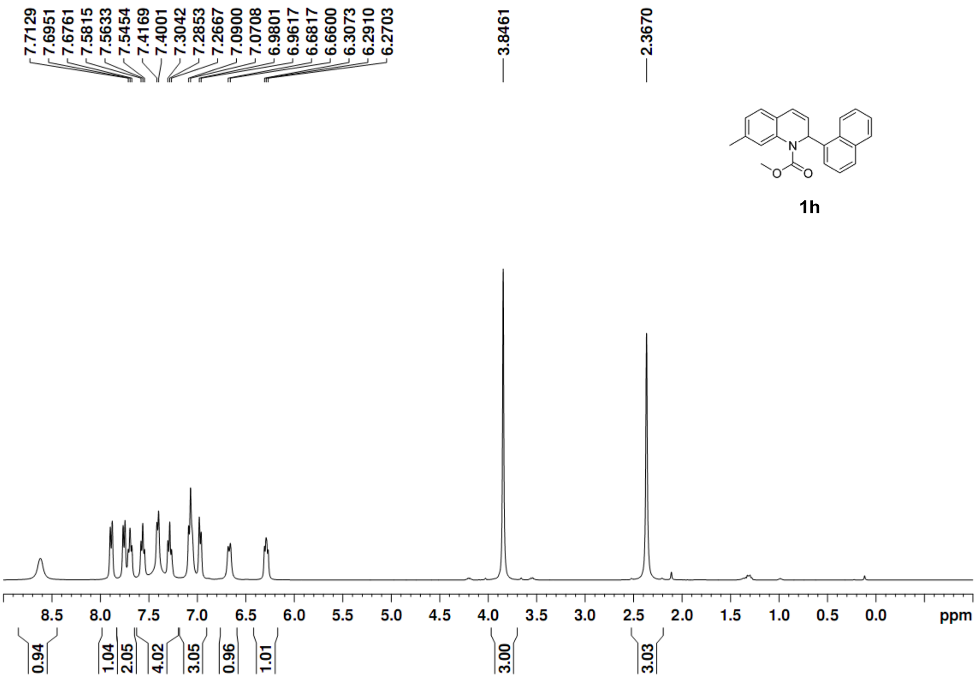

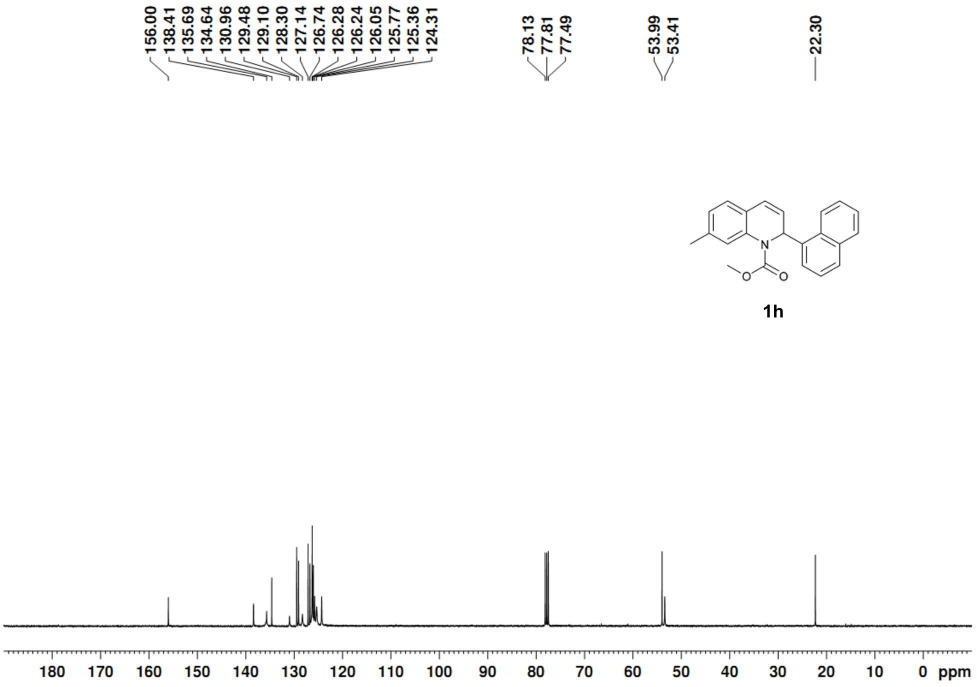

(*S*)-methyl 2-(naphthalen-2-yl)quinoline-1(2*H*)-carboxylate (1i):

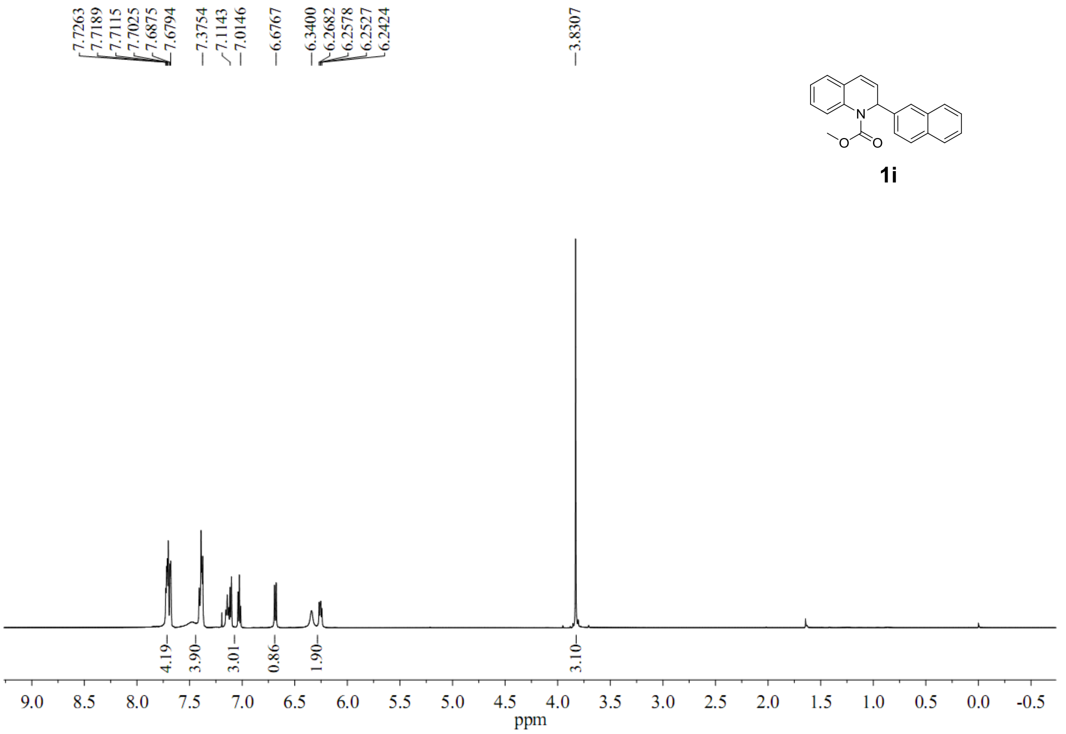

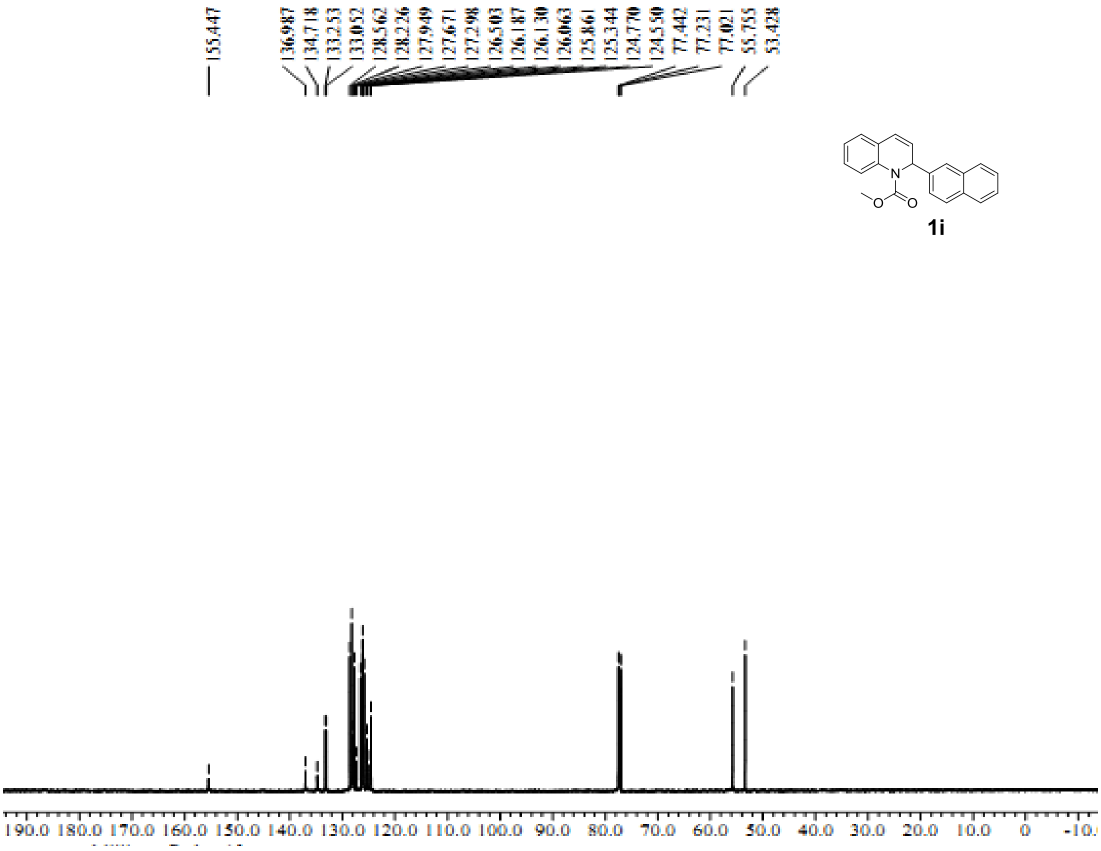

(*S*)-methyl 2-phenylquinoline-1(2*H*)-carboxylate (1j):

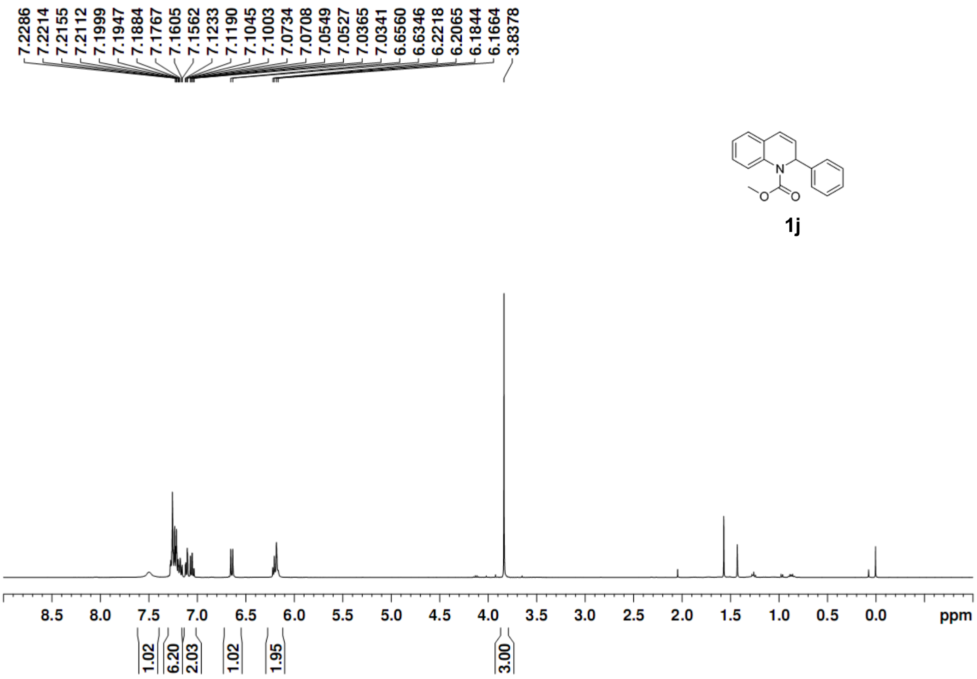

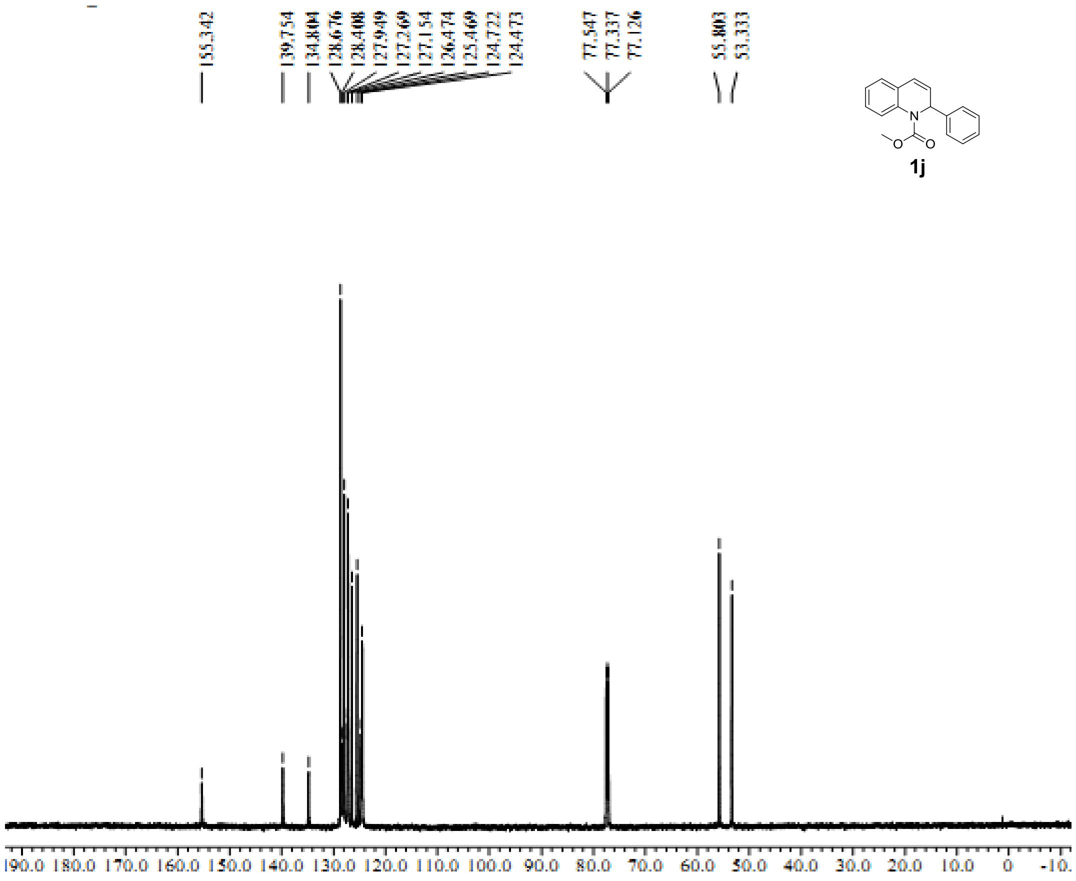

(*S*)-methyl 2-(o-tolyl)quinoline-1(2*H*)-carboxylate (1k):

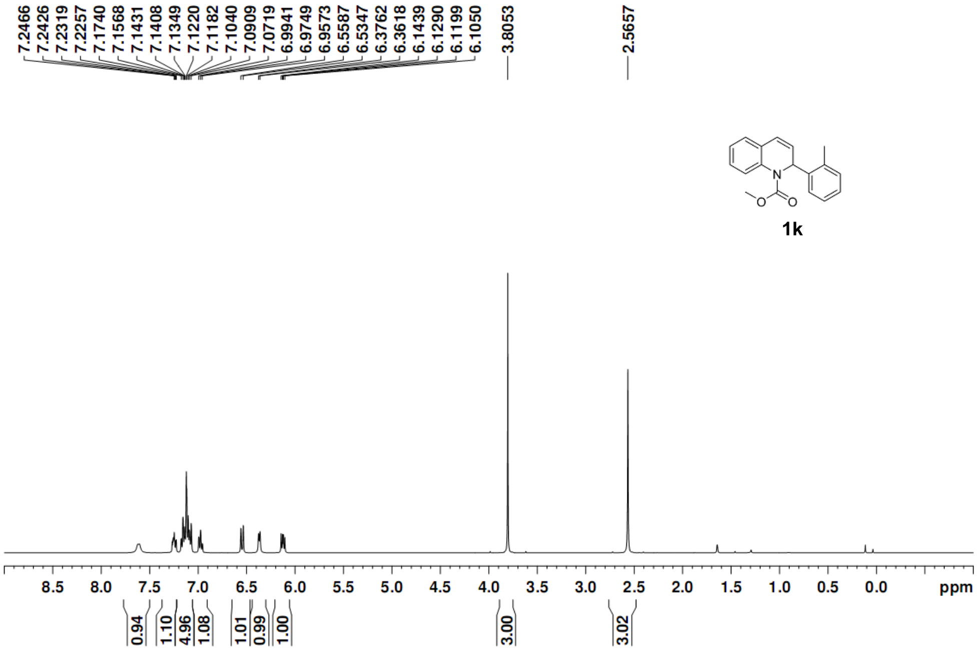

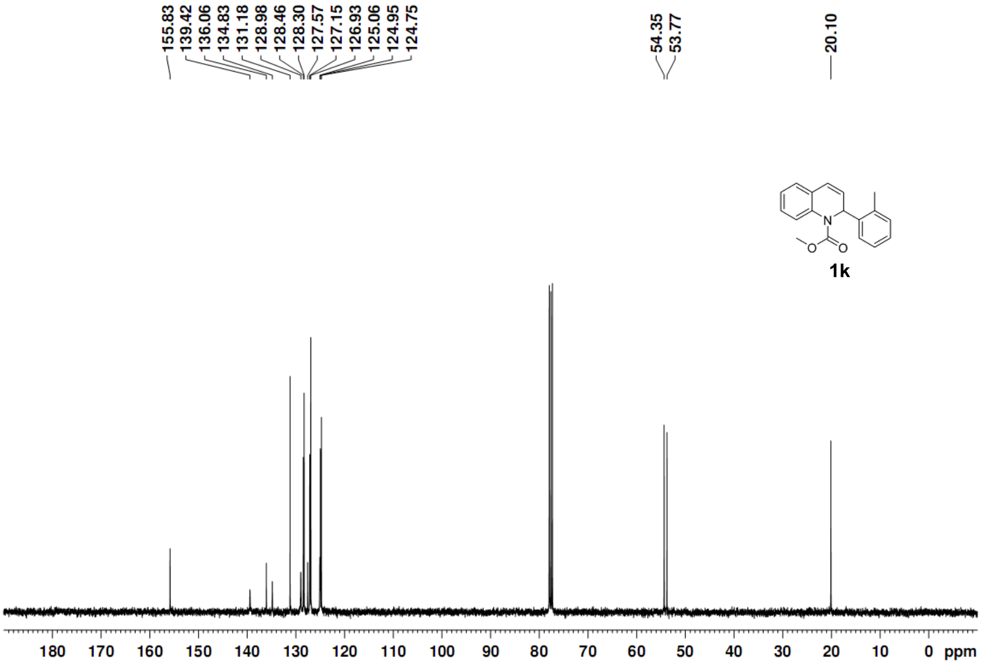

(*S*)-methyl 2-(m-tolyl)quinoline-1(2*H*)-carboxylate (1l):

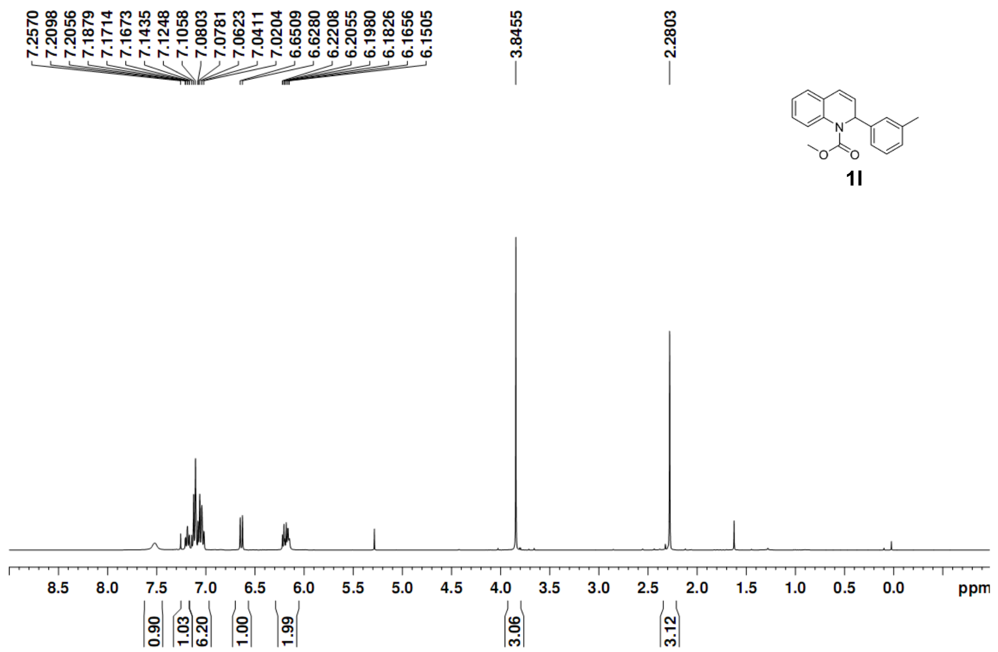

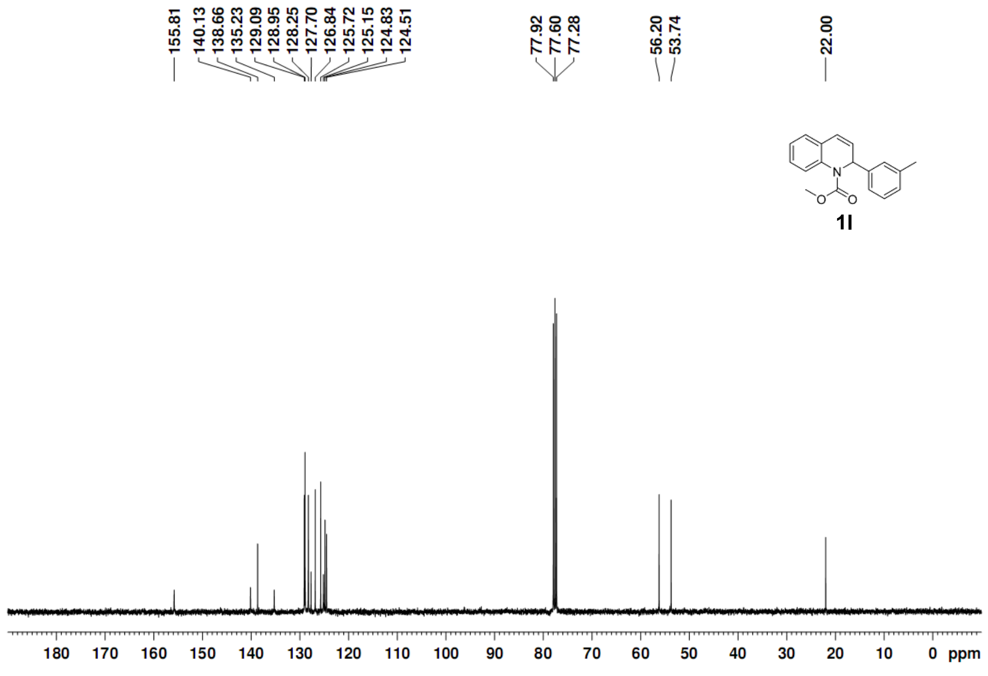

(*S*)-methyl 2-(3,5-dimethylphenyl)quinoline-1(2*H*)-carboxylate (1m):

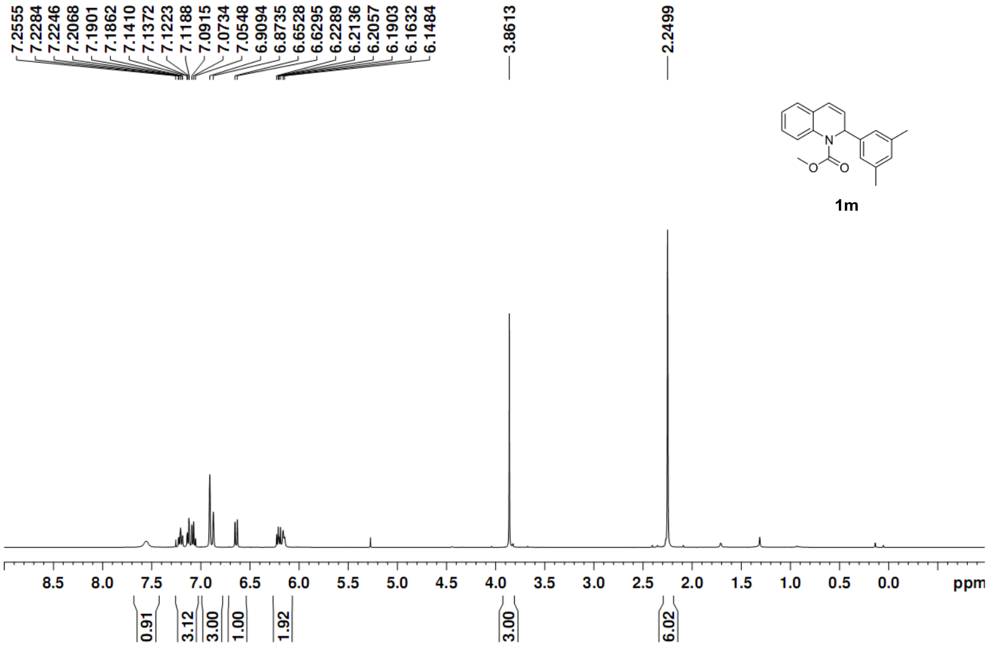

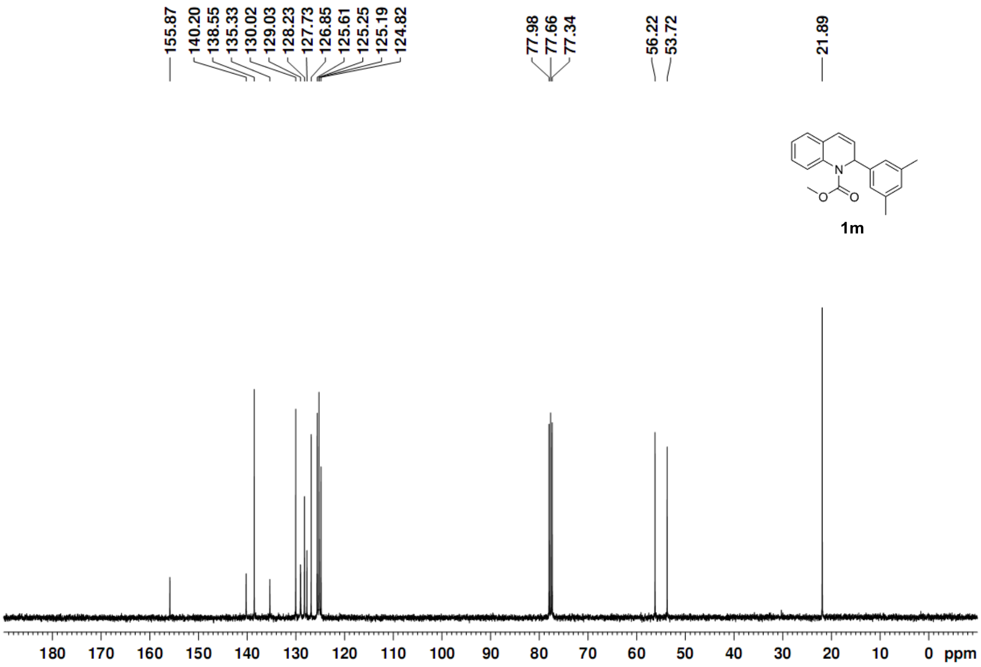

(*S*)-methyl 2-(3-chlorophenyl)quinoline-1(2*H*)-carboxylate (1n):

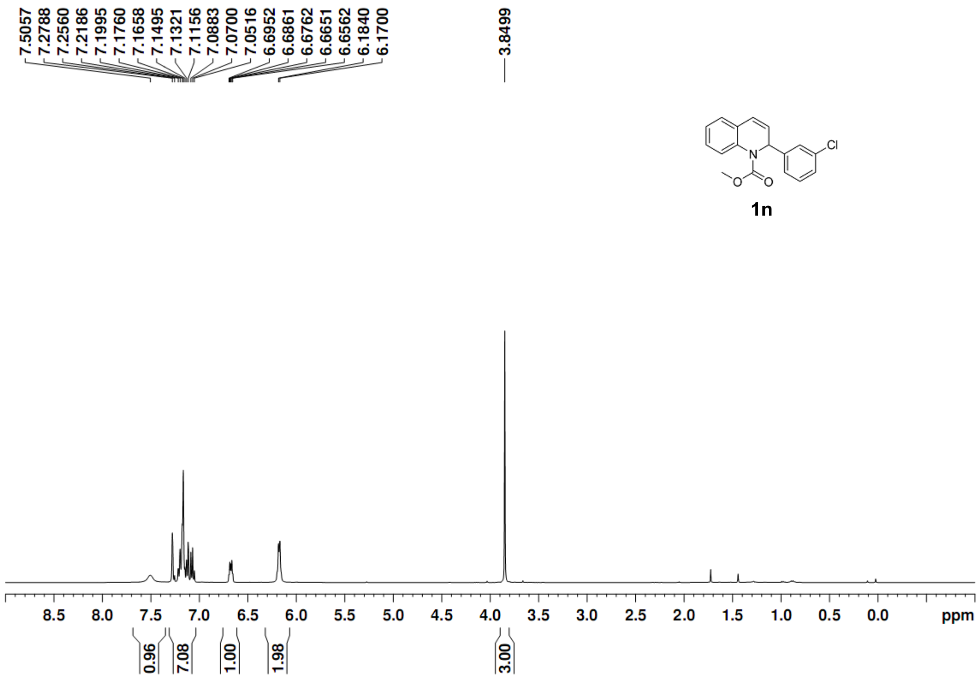

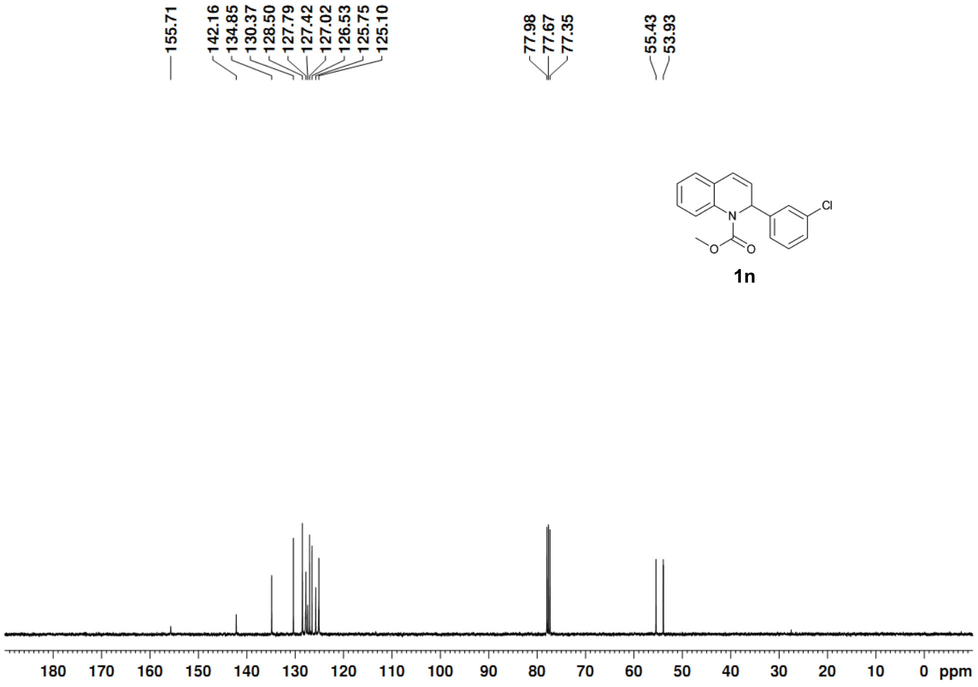

(*S*)-methyl 2-(4-chlorophenyl)quinoline-1(2*H*)-carboxylate (1o):

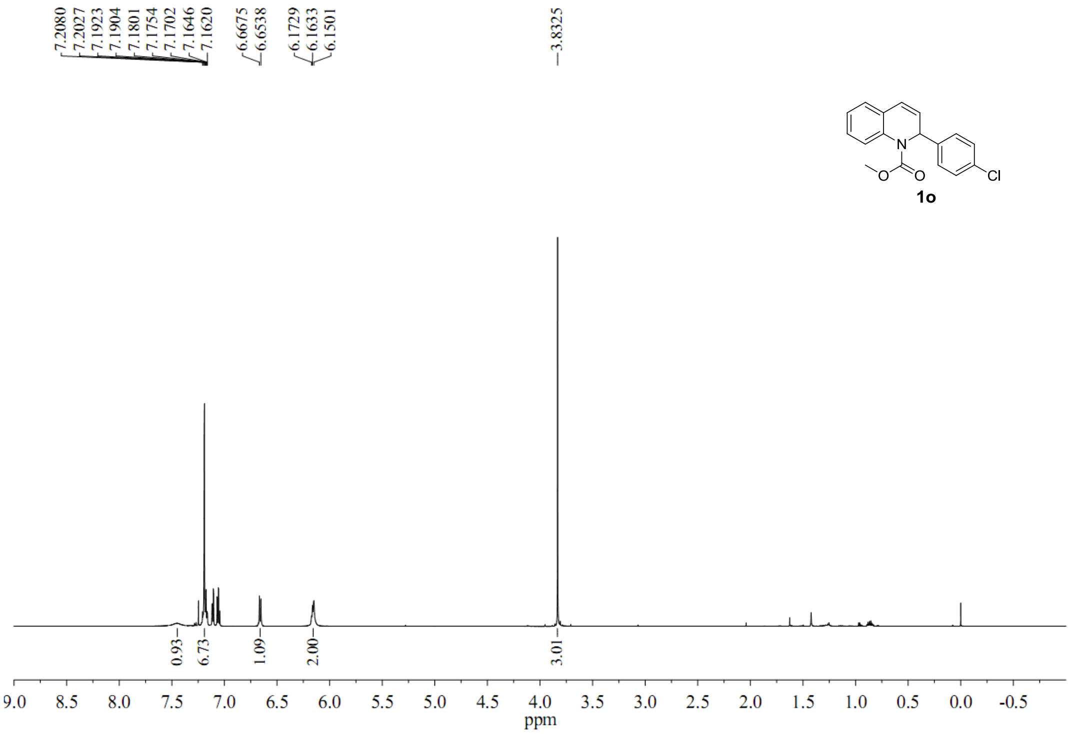

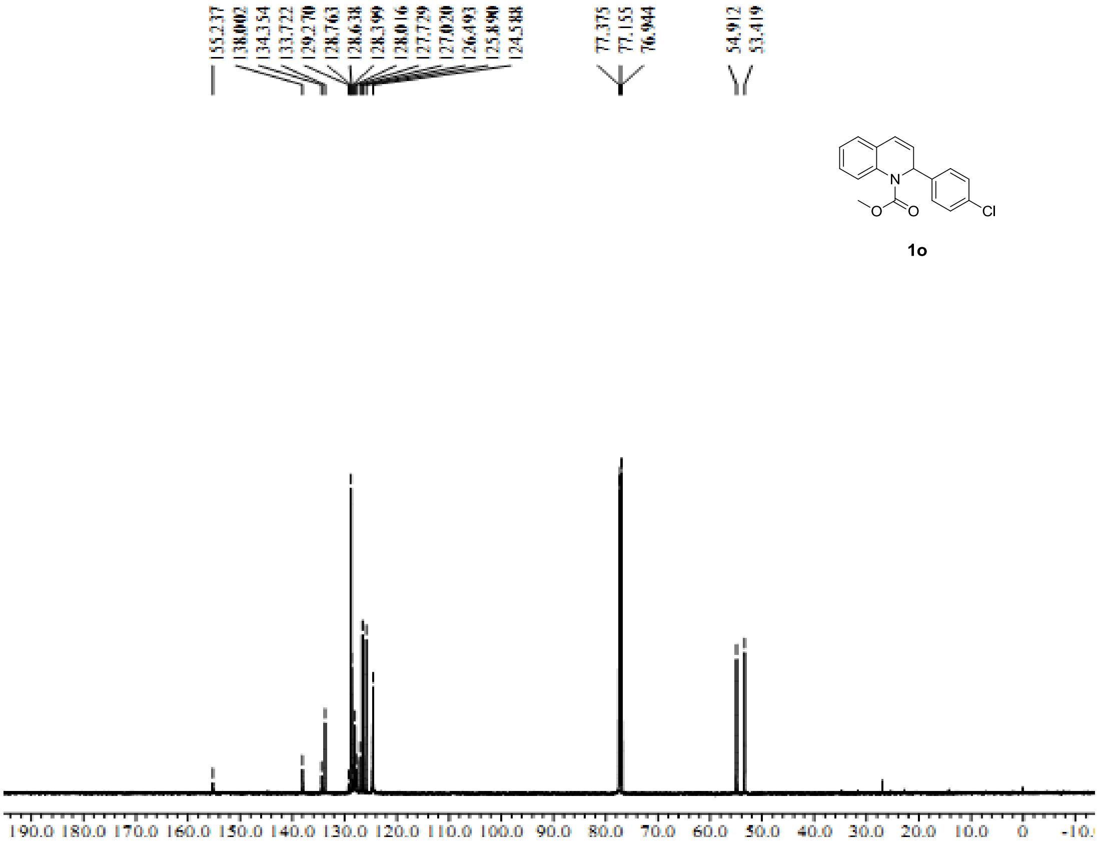

(*S*)-methyl 6-methoxy-2-(4-methoxyphenyl)quinoline-1(2*H*)-carboxylate (1p):

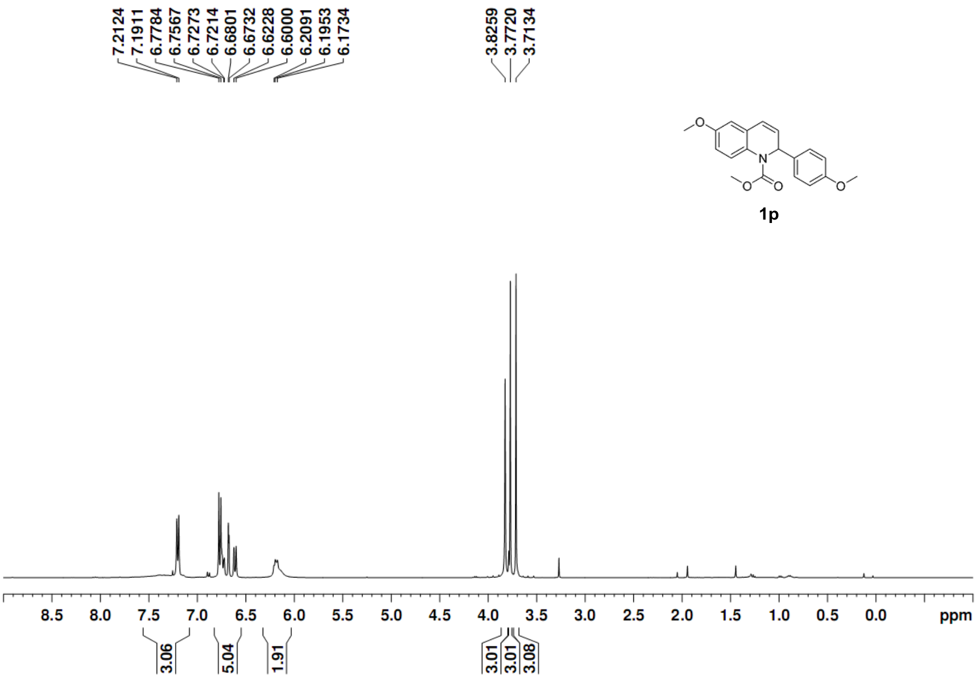

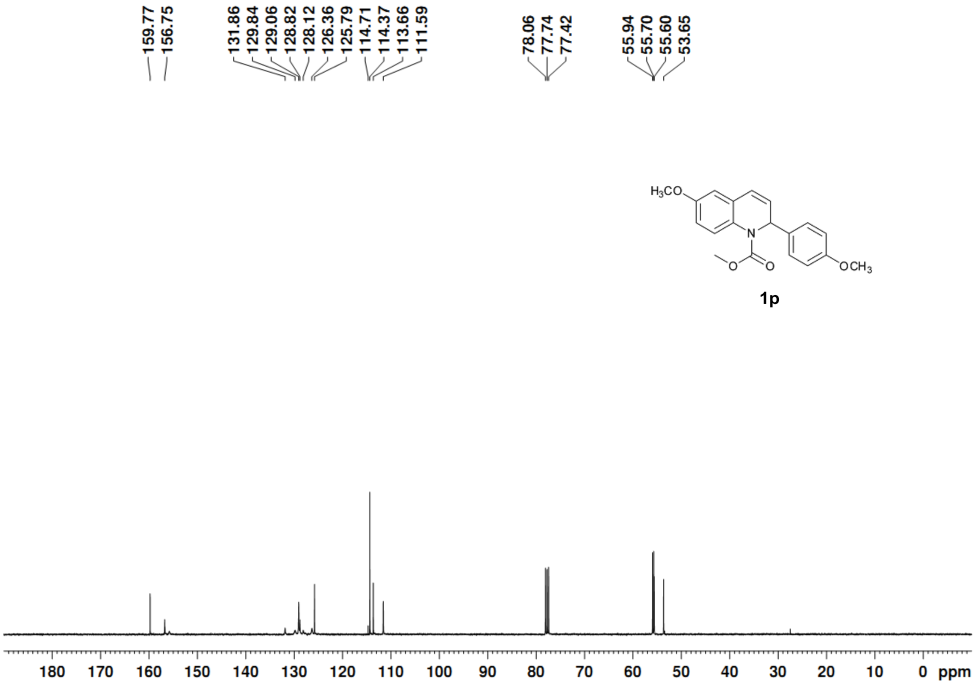

(*R*)-methyl 2-methylquinoline-1(2*H*)-carboxylate (1q):

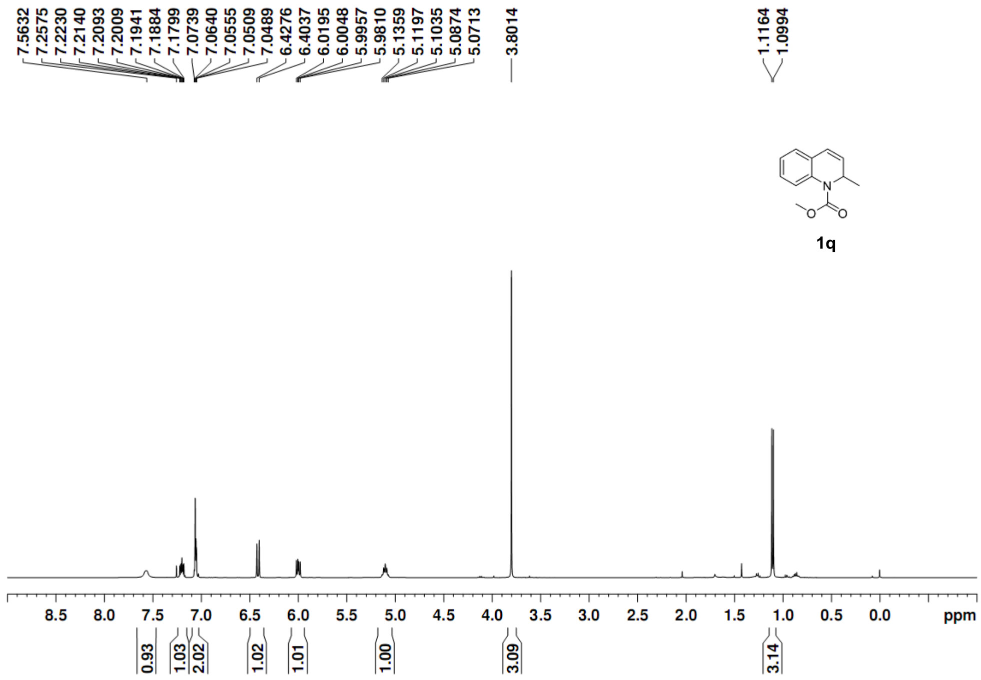

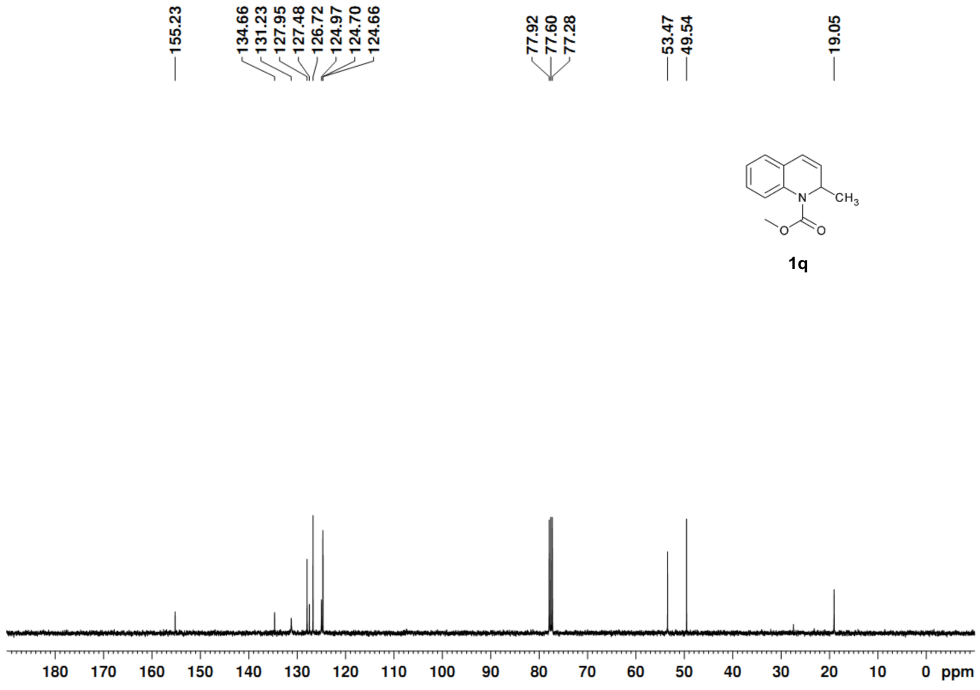

(*R*)-methyl 2-butylquinoline-1(2*H*)-carboxylate (1r):

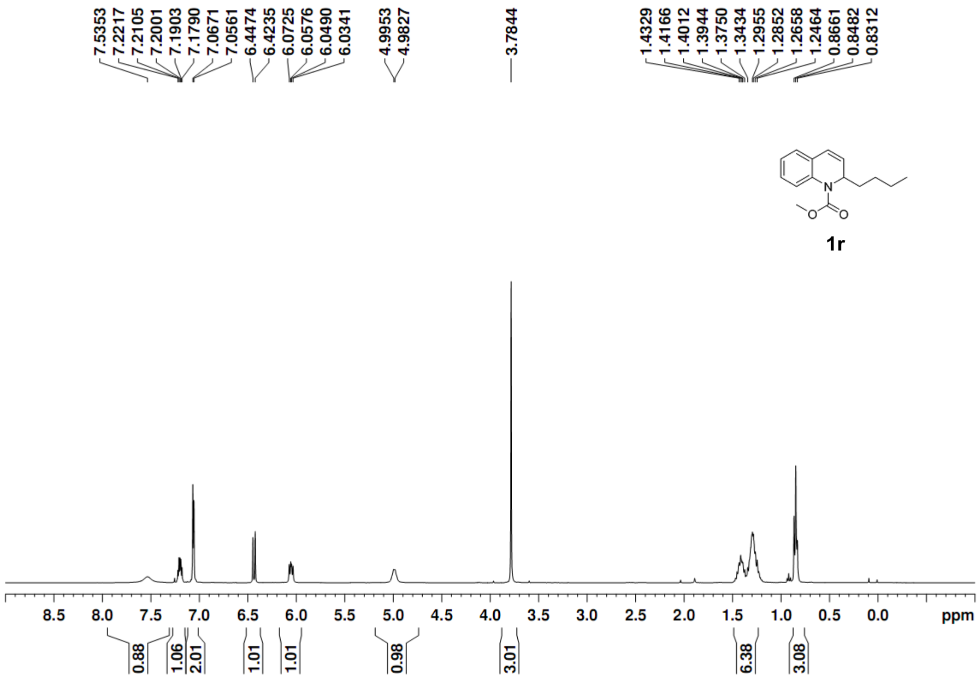

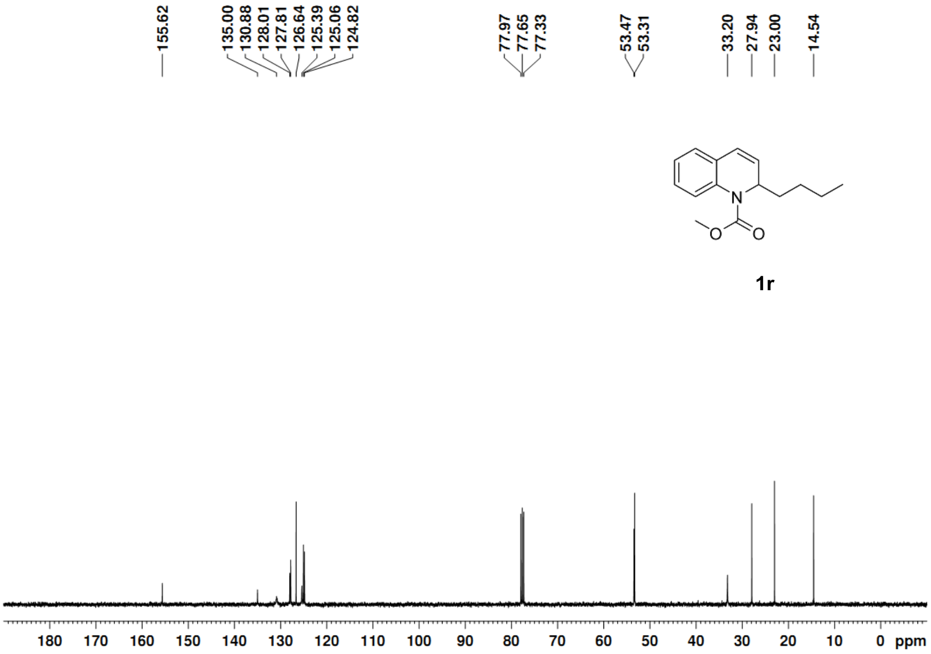

1-methyl-1H-indene (1s):

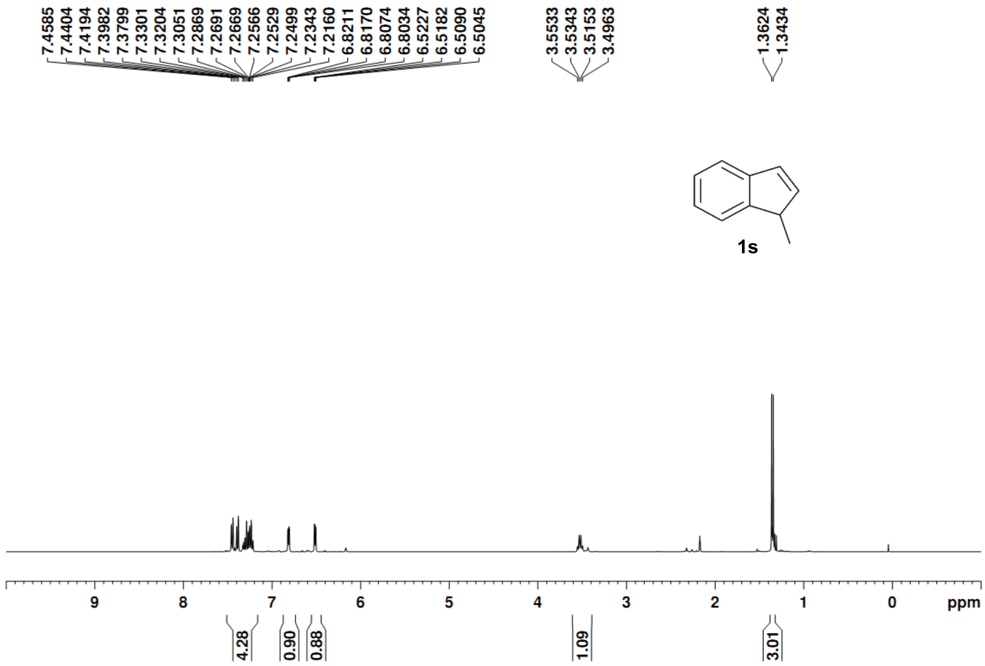

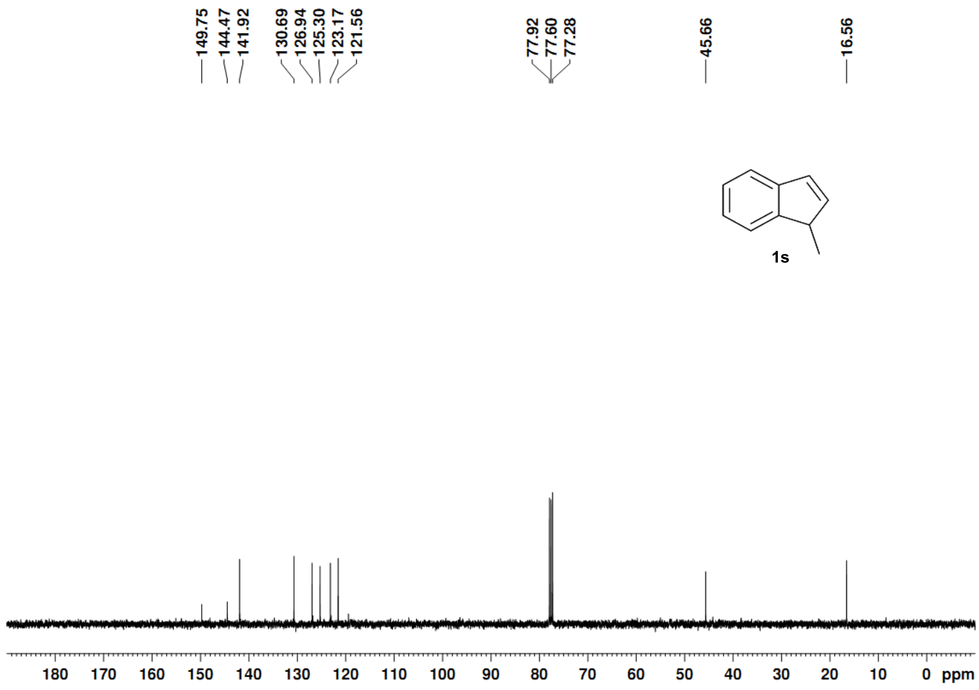

(*E*)-but-1-ene-1,3-diyldibenzene (1t):

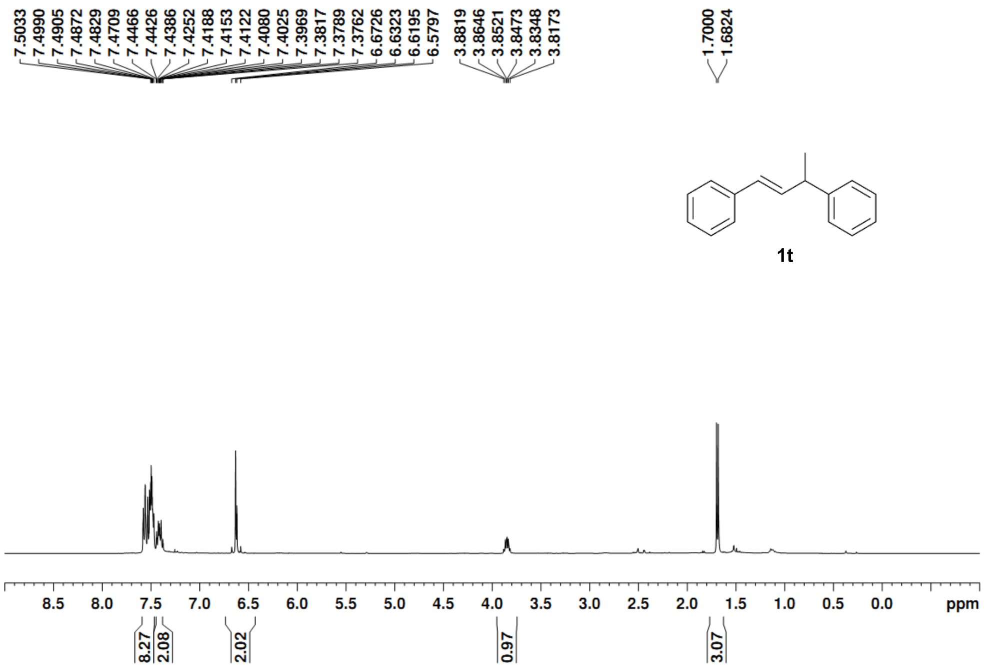

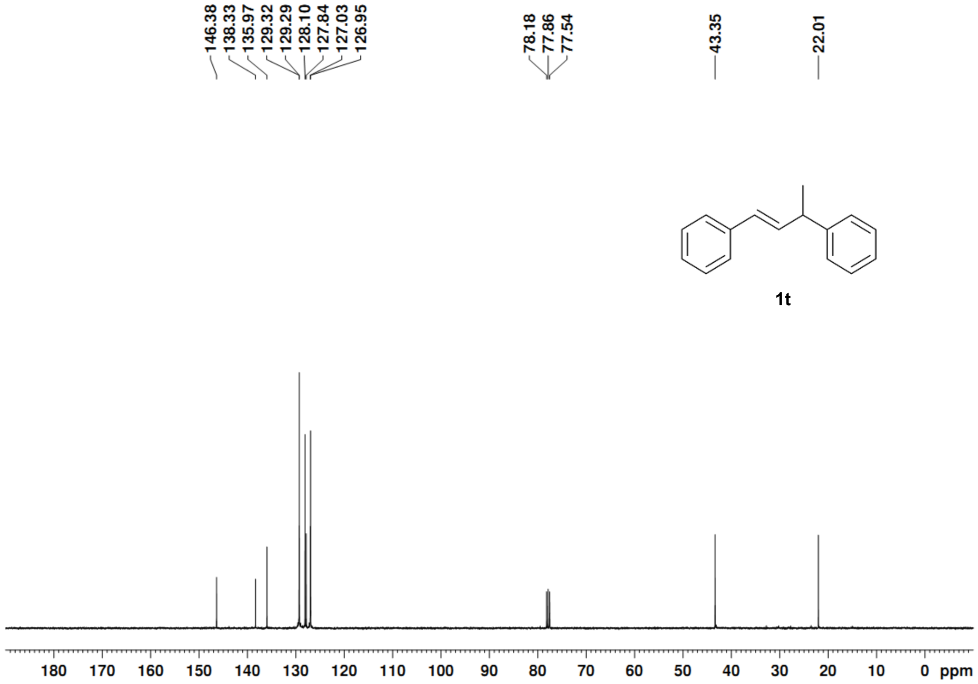

(*2R*,*3R*)-methyl 2-(naphthalen-1-yl)-3-(4,4,5,5-tetramethyl-1,3,2-dioxaborolan-2-yl)-3,4-dihydroquinoline-1(2*H*)-carboxylate (2a):

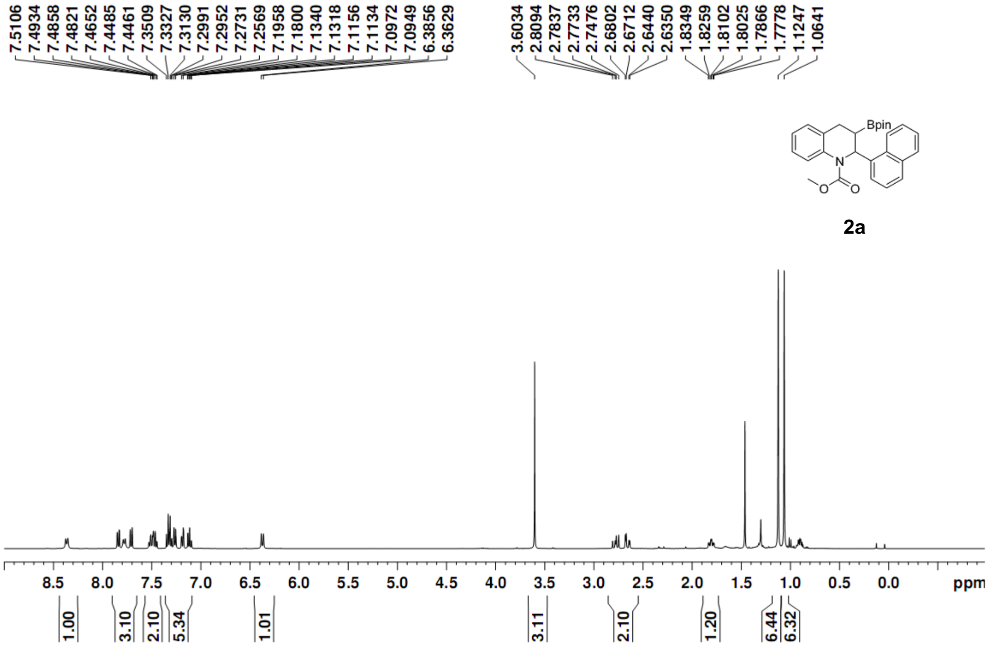

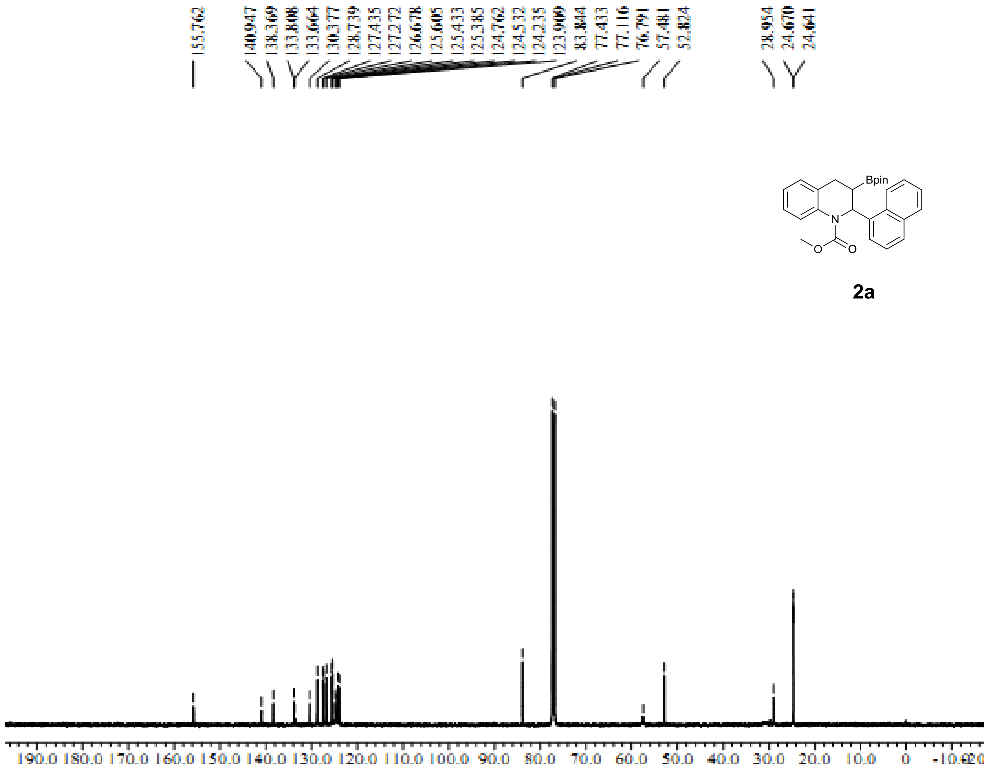

(2*R*,3*R*)-phenyl 2-(naphthalen-1-yl)-3-(4,4,5,5-tetramethyl-1,3,2-dioxaborolan-2-yl)-3,4-dihydroquinoline-1(2*H*)-carboxylate (2b):

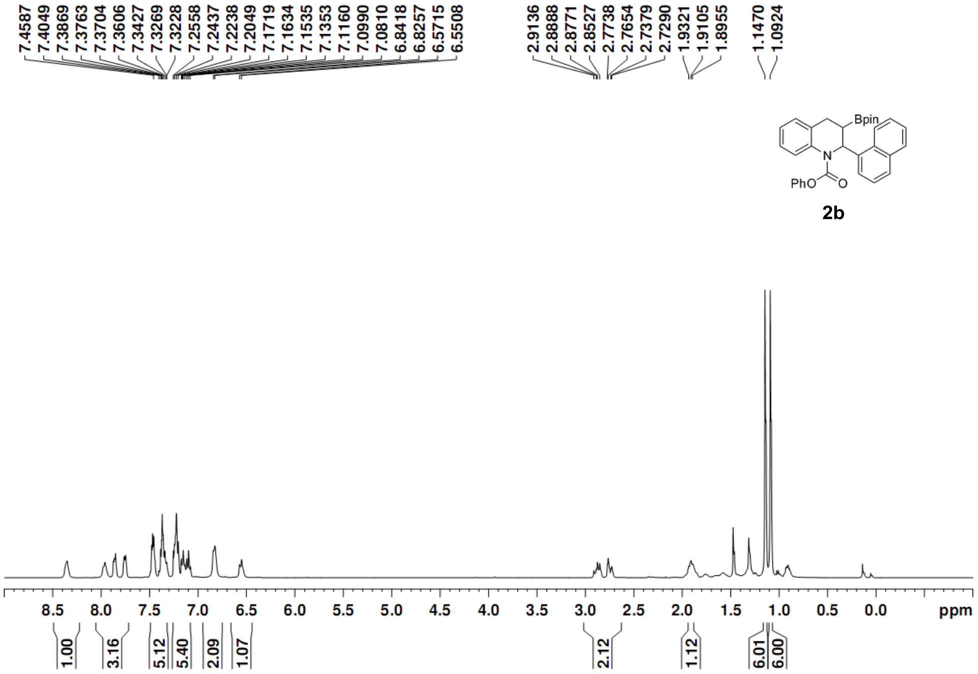

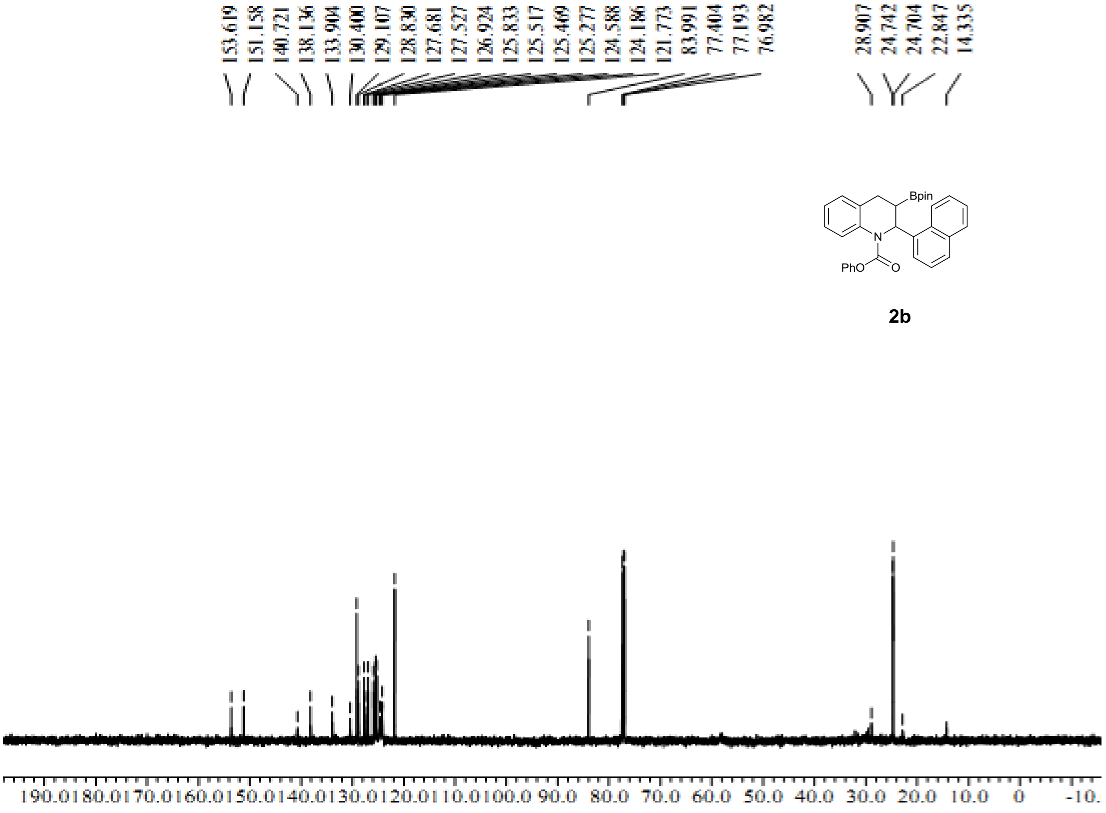

(2*R*,3*R*)-benzyl 2-(naphthalen-1-yl)-3-(4,4,5,5-tetramethyl-1,3,2-dioxaborolan-2-yl)-3,4-dihydroquinoline-1(2*H*)-carboxylate (2c):

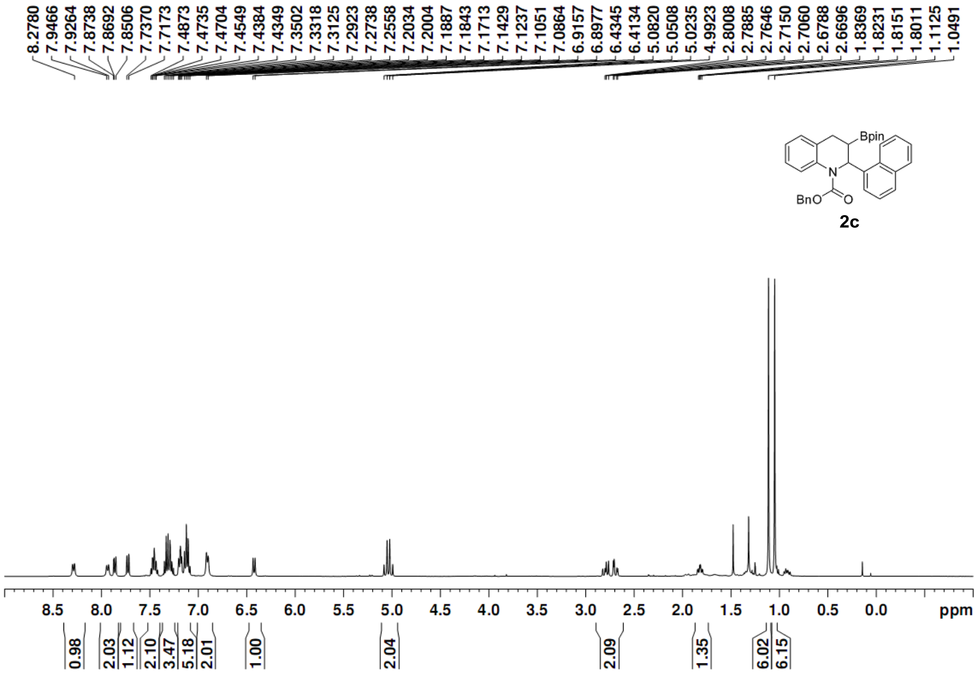

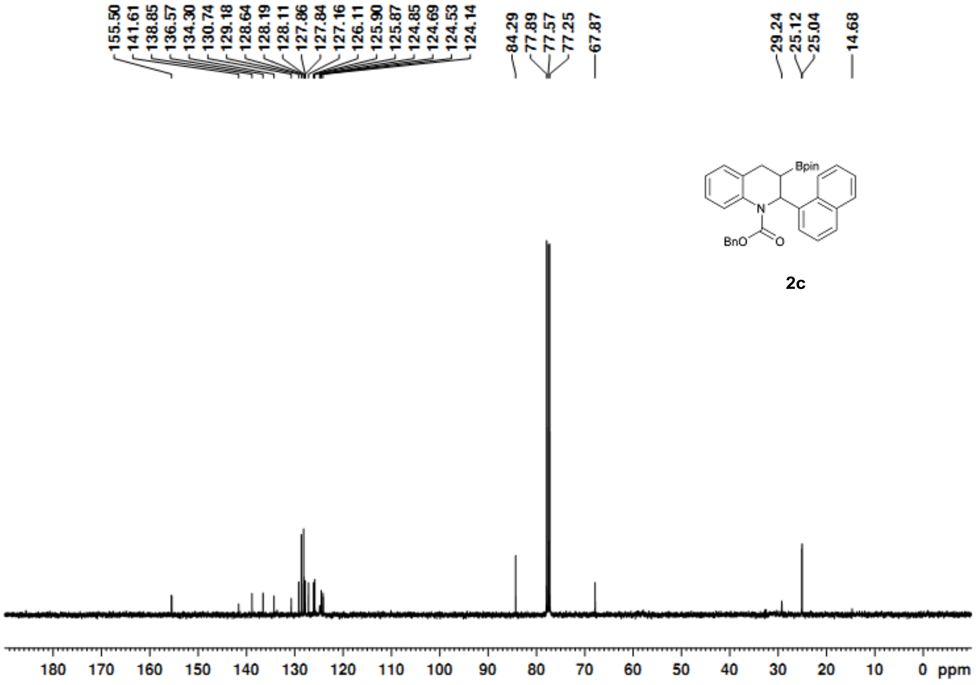

(*2R*,*3R*)-isopropyl 2-(naphthalen-1-yl)-3-(4,4,5,5-tetramethyl-1,3,2-dioxaborolan-2-yl)-3,4-dihydroquinoline-1(2*H*)-carboxylate (2d):

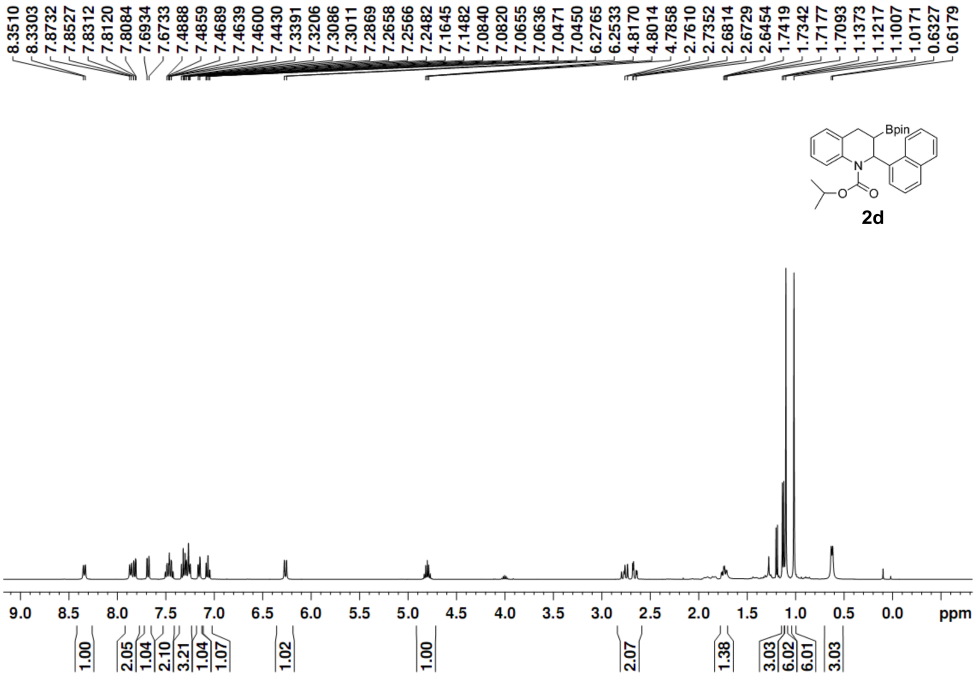

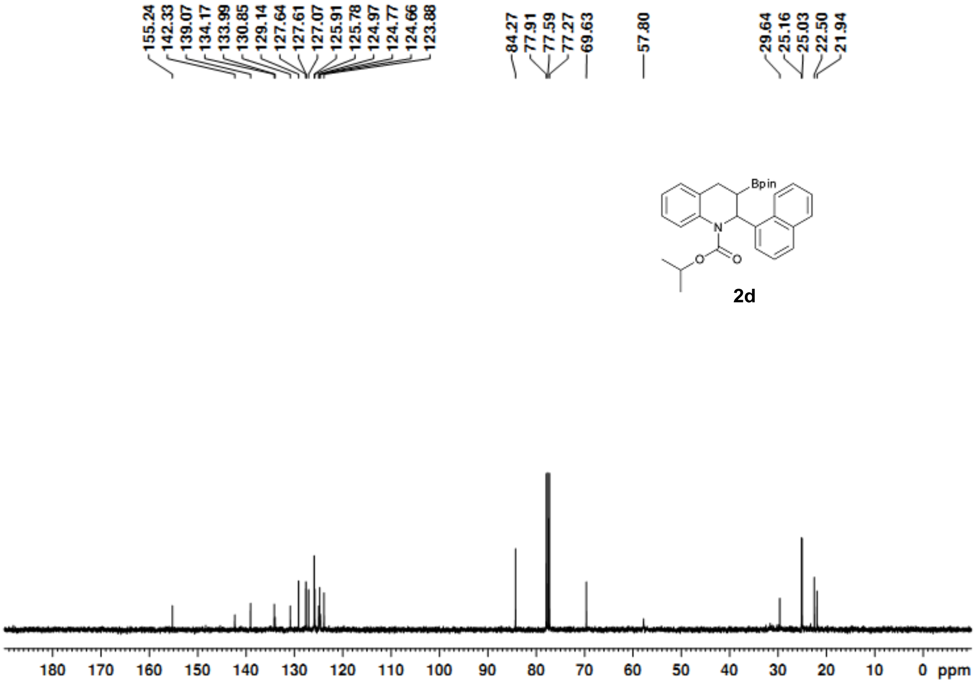

(*2R,3R*)-isobutyl 2-(naphthalen-1-yl)-3-(4,4,5,5-tetramethyl-1,3,2-dioxaborolan-2-yl)-3,4-dihydroquinoline-1(2*H*)-carboxylate (2e):

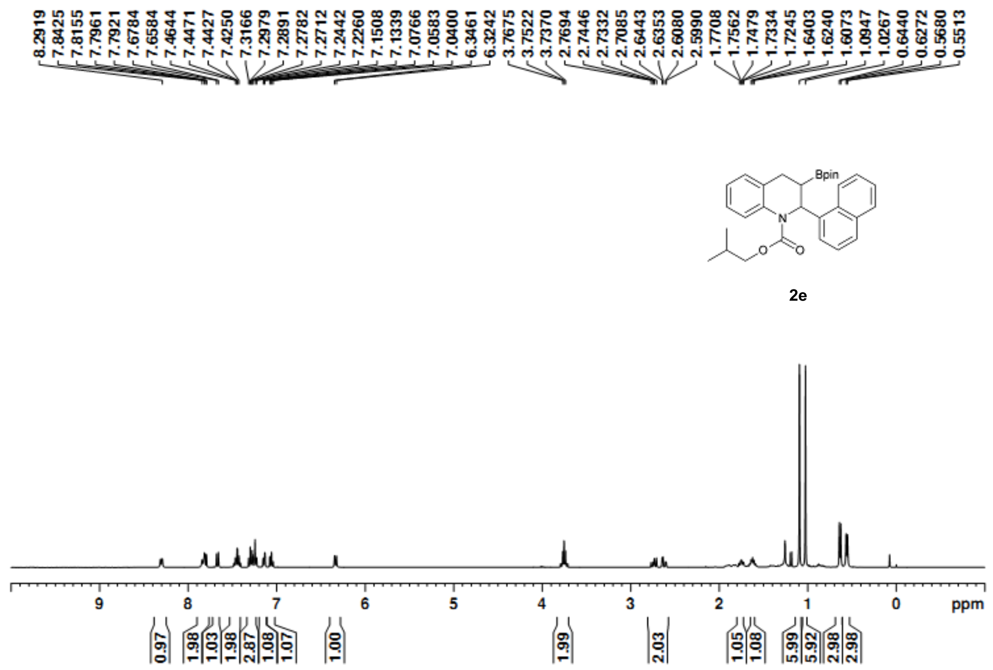

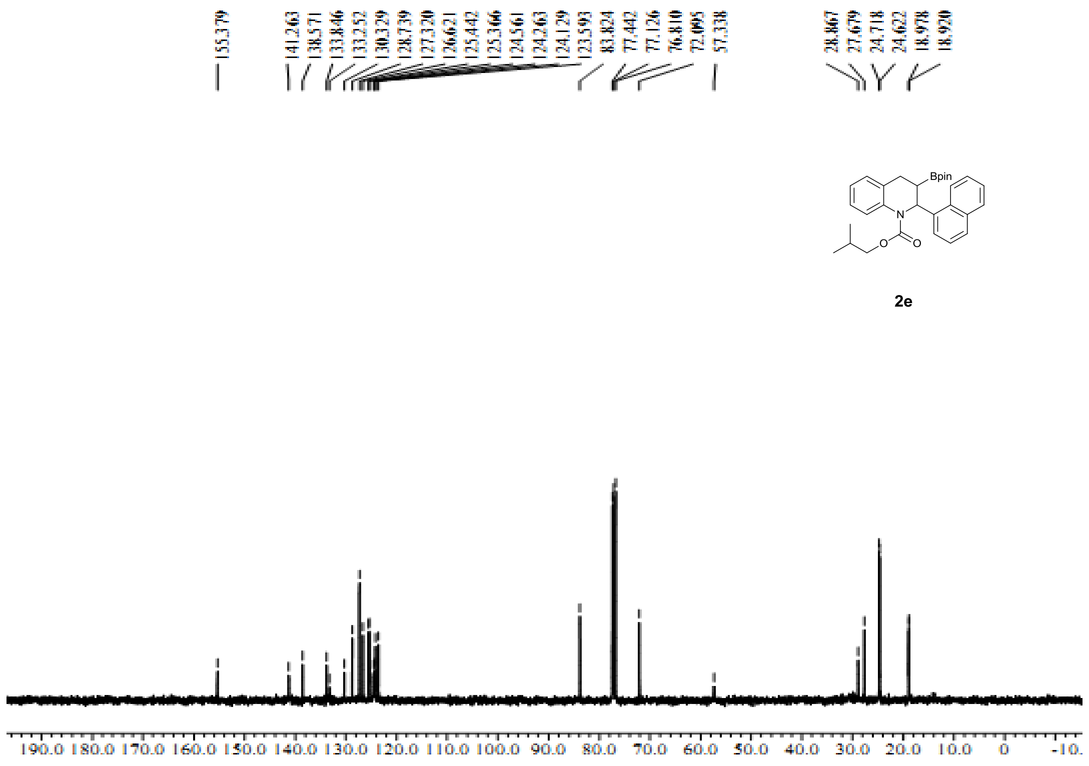

(*2R*,*3R*)-methyl 6-methoxy-2-(naphthalen-1-yl)-3-(4,4,5,5-tetramethyl-1,3,2-dioxaborolan-2-yl)-3,4-dihydroquinoline-1(2*H*)-carboxylate (2f):

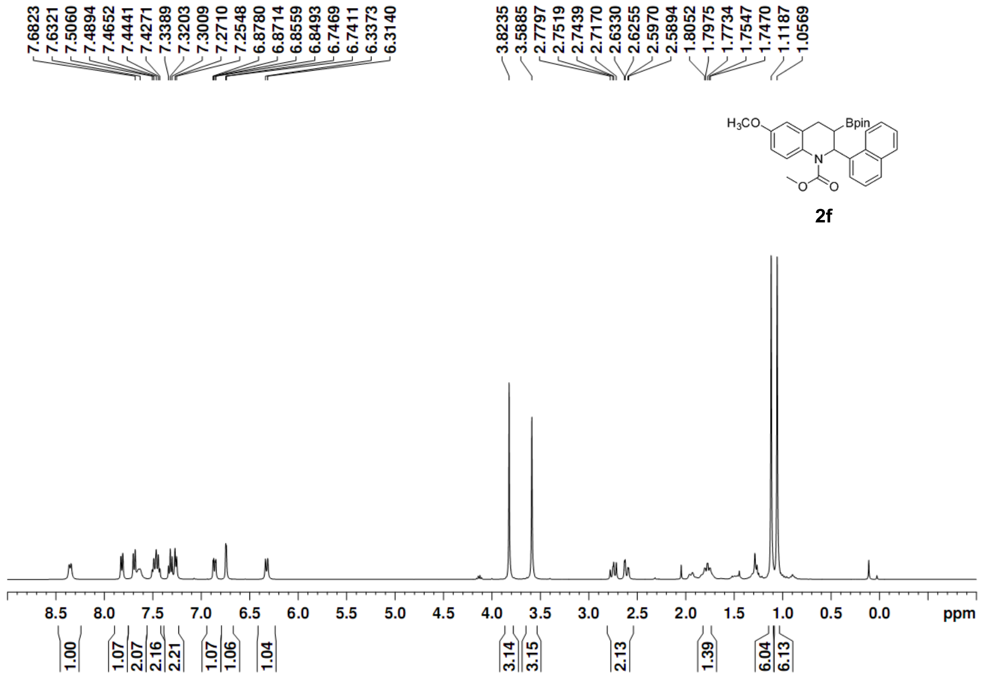

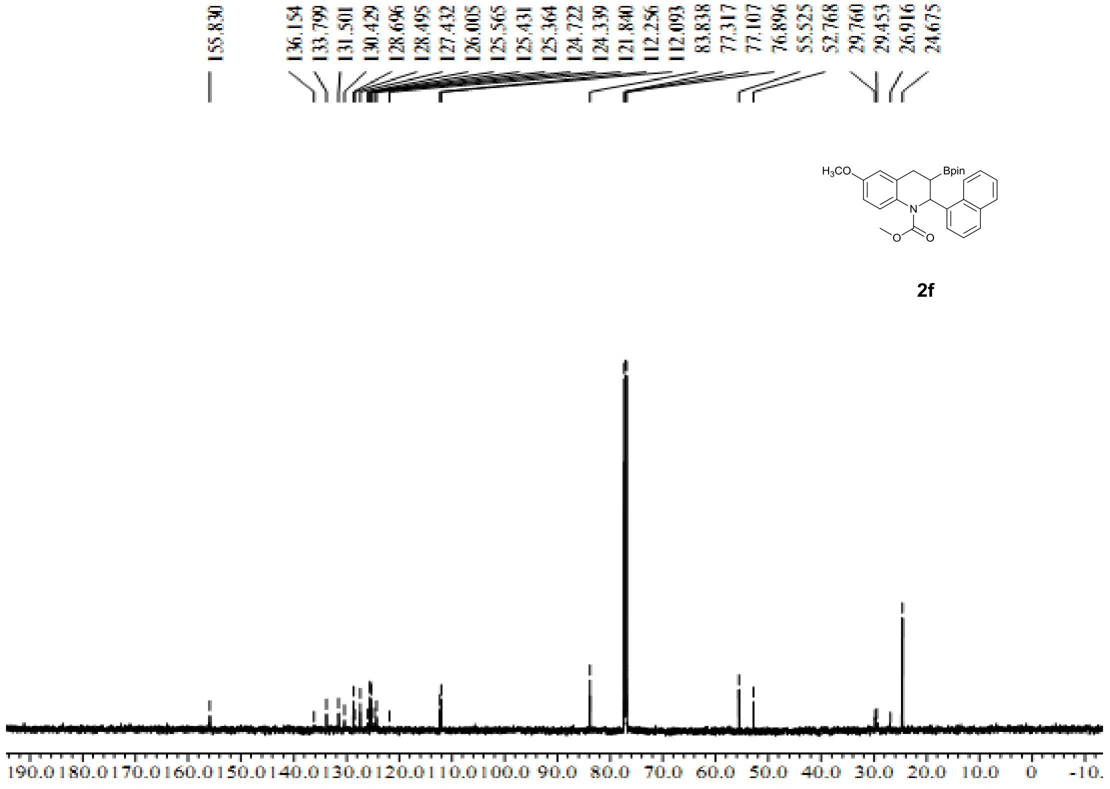

(*2R*,*3R*)-methyl 6-methyl-2-(naphthalen-1-yl)-3-(4,4,5,5-tetramethyl-1,3,2-dioxa-borolan-2-yl)-3,4-dihydroquinoline-1(2*H*)-carboxylate (2g):

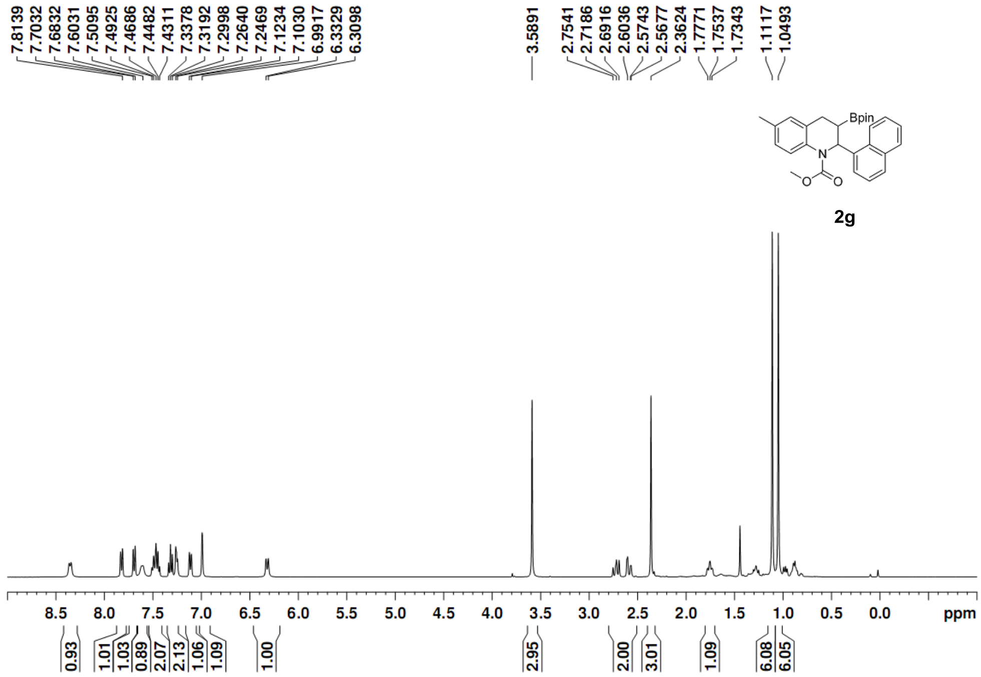

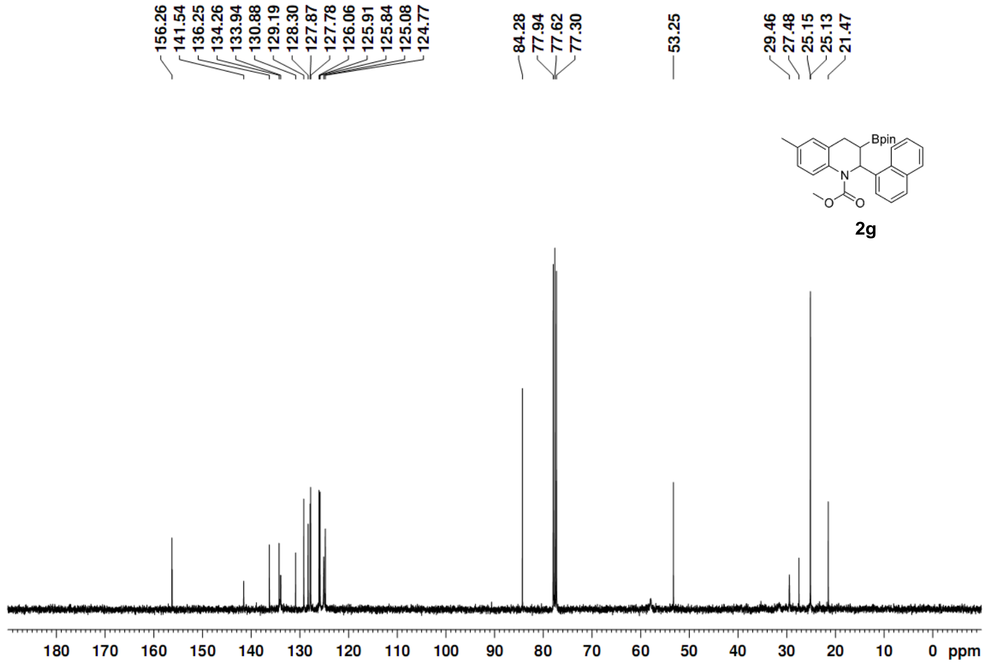

(*2R,3R*)-methyl 7-methyl-2-(naphthalen-1-yl)-3-(4,4,5,5-tetramethyl-1,3,2-dioxaborolan-2-yl)-3,4-dihydroquinoline-1(2*H*)-carboxylate (2h):

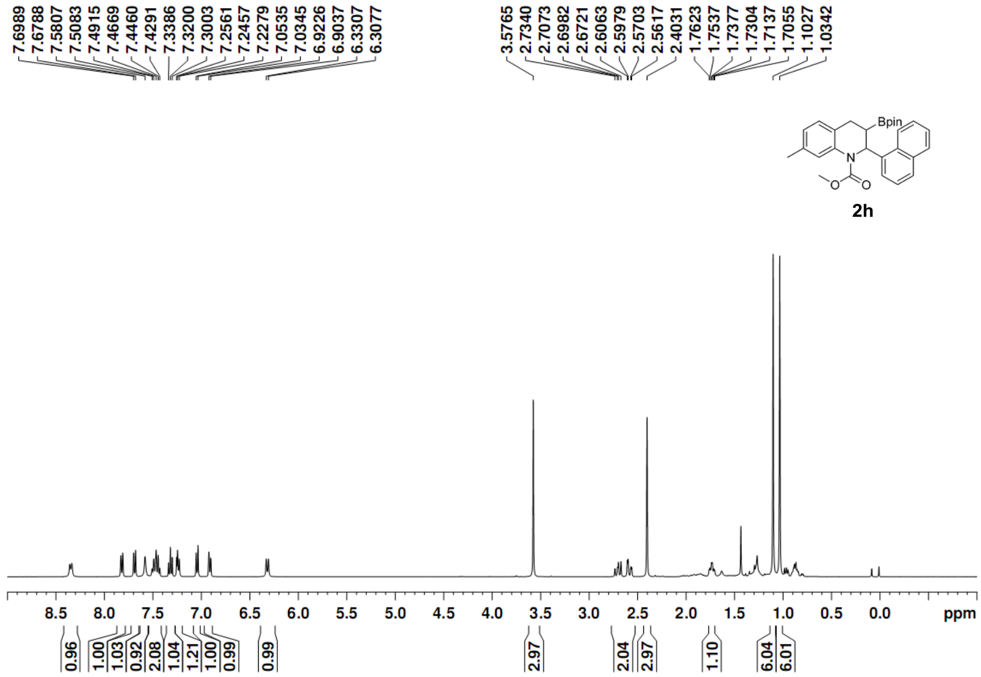

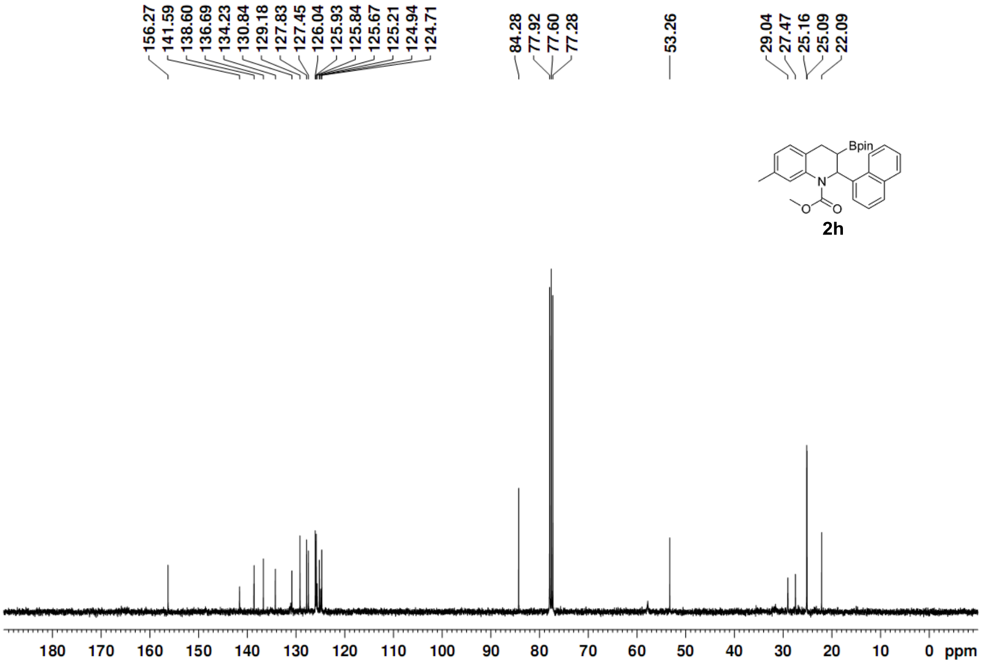

(2*R*,3*R*)-methyl 2-(naphthalen-2-yl)-3-(4,4,5,5-tetramethyl-1,3-dioxolan-2-yl)-3,4-dihydroquinoline-1(2*H*)-carboxylate (2i):

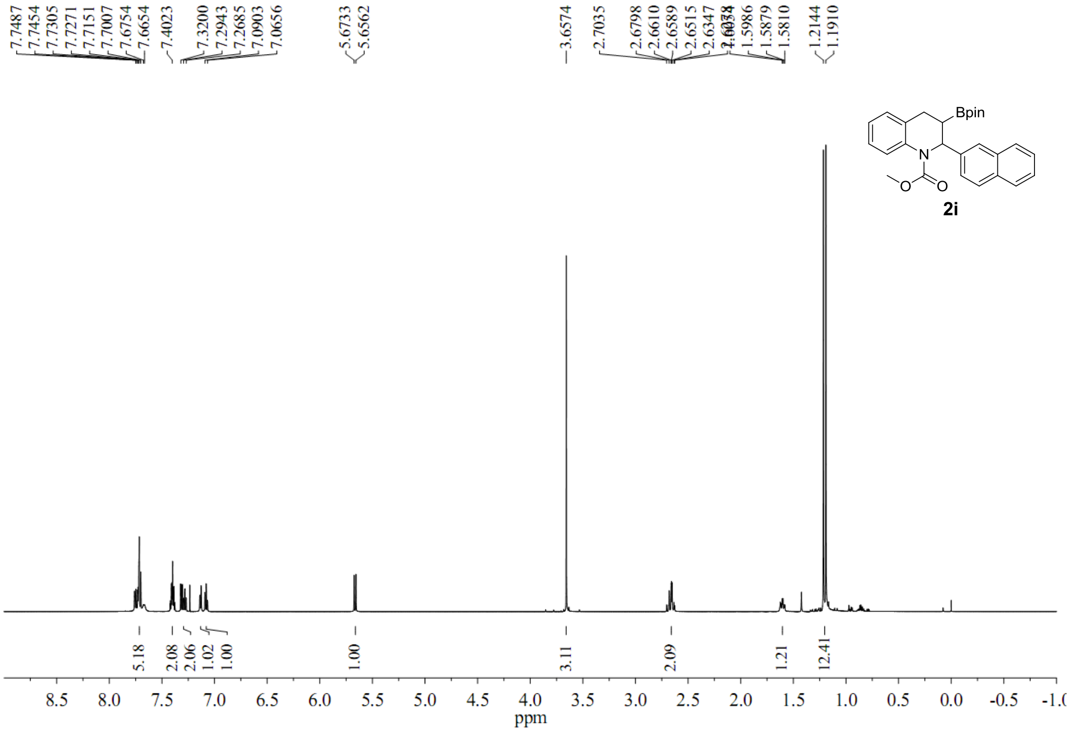

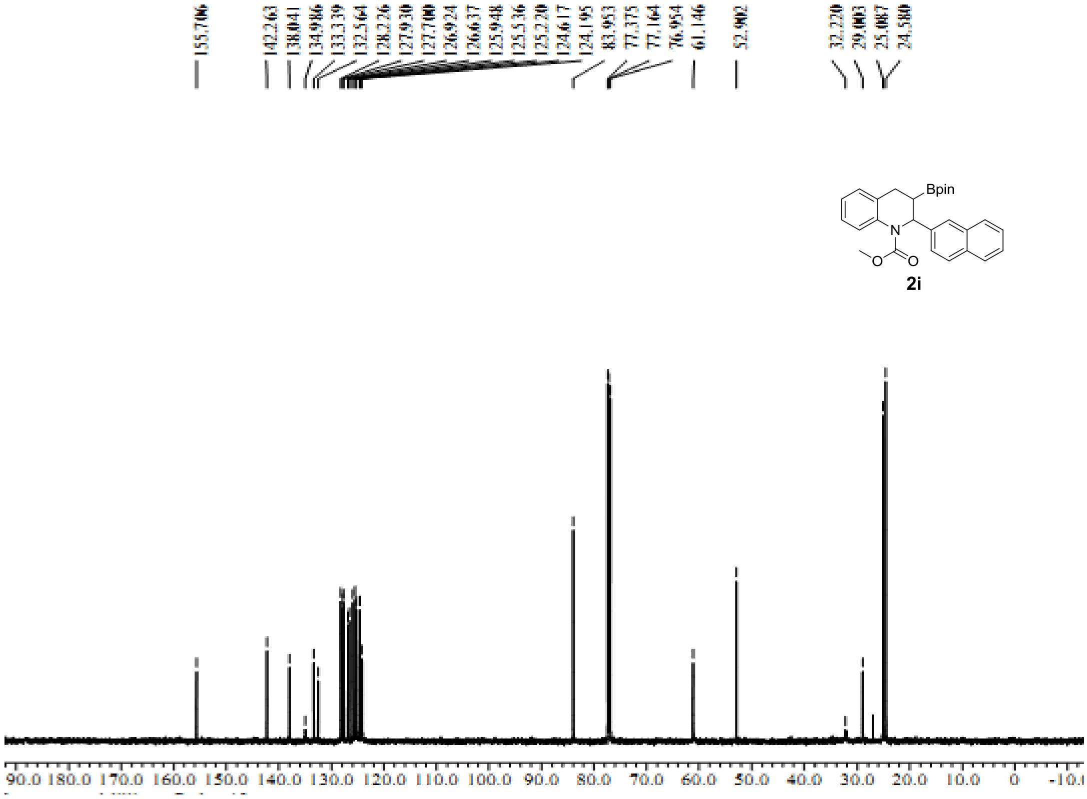

(*2R*,*3R*)-methyl 2-phenyl-3-(4,4,5,5-tetramethyl-1,3,2-dioxaborolan-2-yl)-3,4-dihydroquinoline-1(2*H*)-carboxylate (2j):

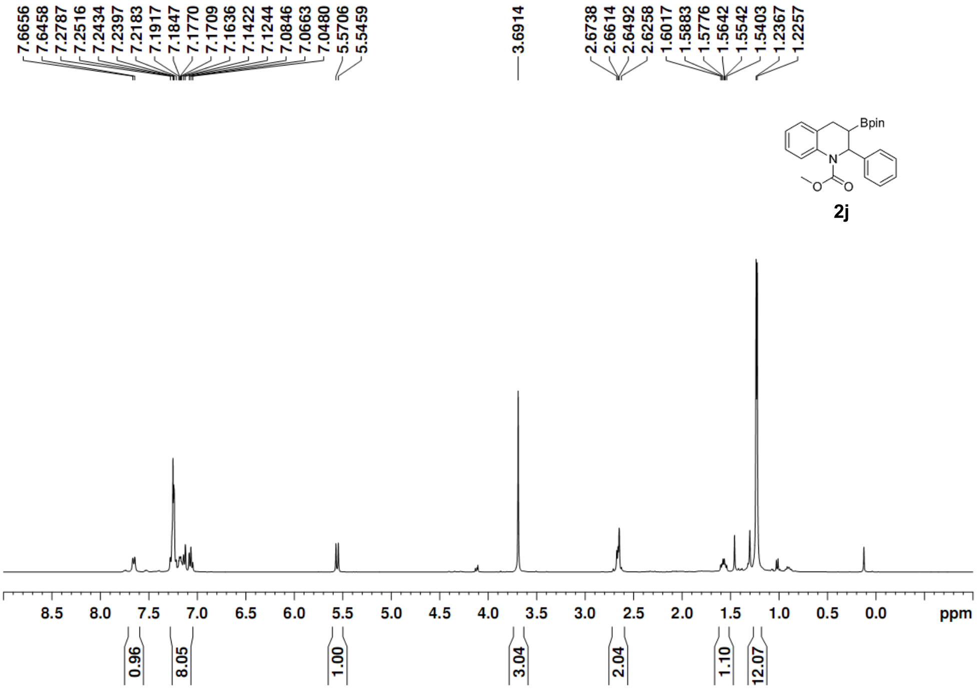

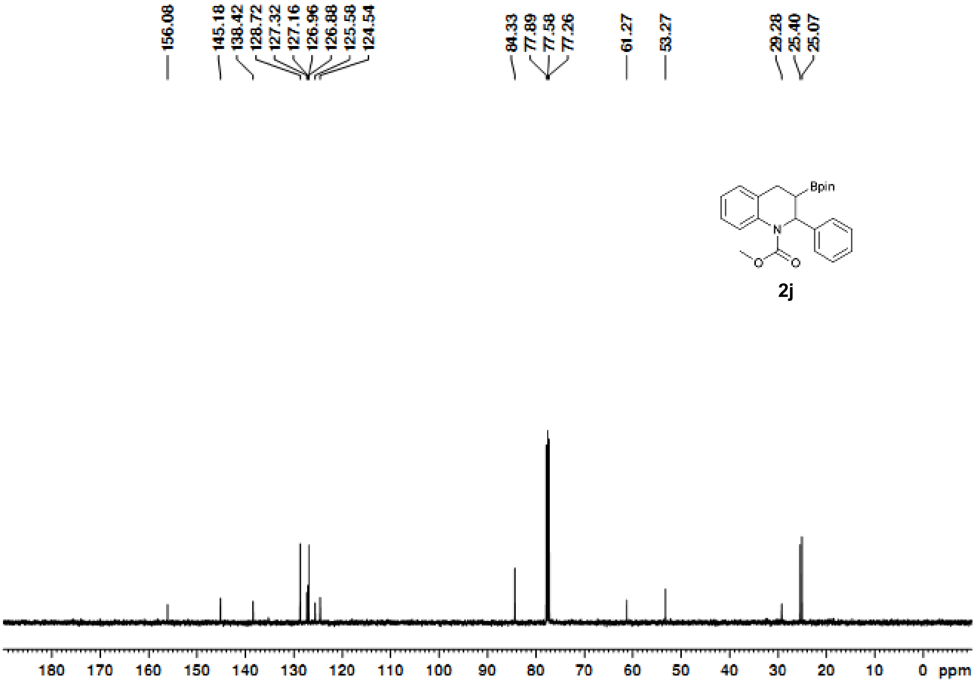

(*2R*,*3R*)-methyl 3-(4,4,5,5-tetramethyl-1,3,2-dioxaborolan-2-yl)-2-(*o*-tolyl)-3,4-dihydroquinoline-1(2*H*)-carboxylate (2k):

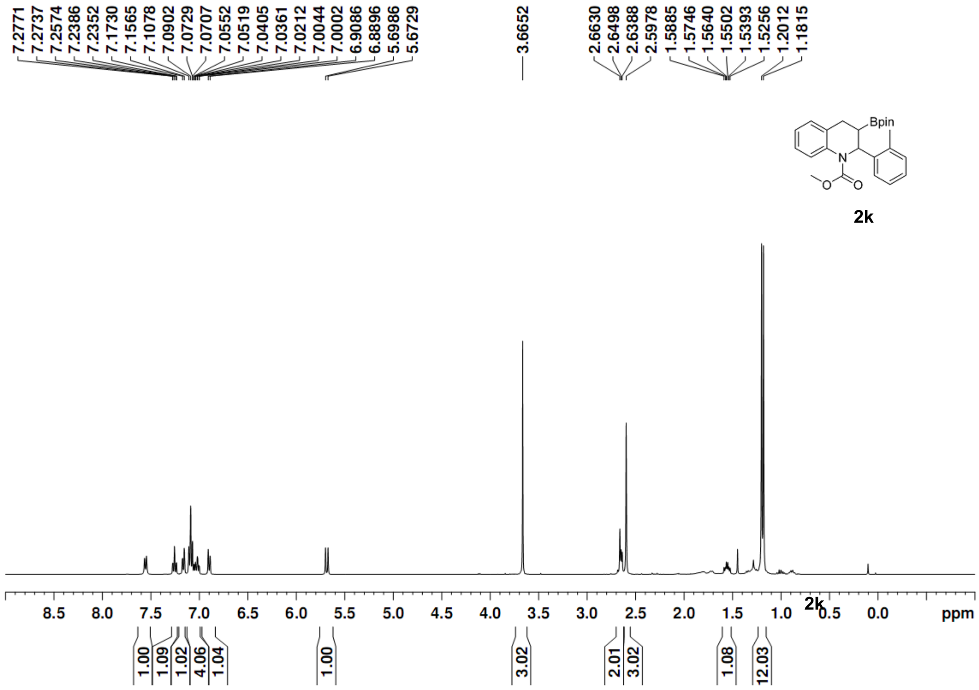

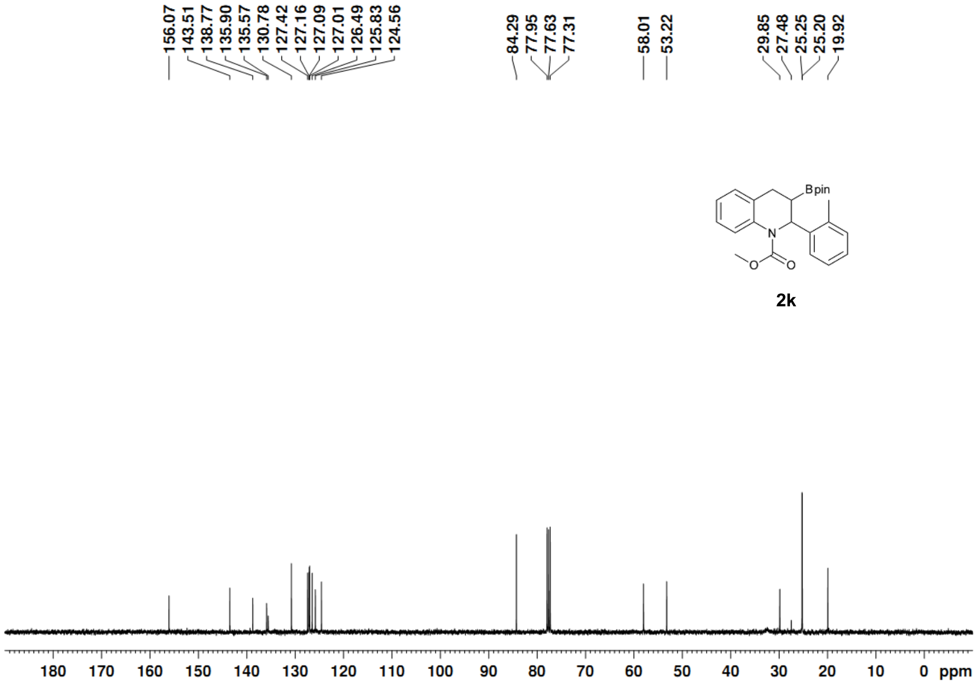

(2*R*,3*R*)-methyl 3-(4,4,5,5-tetramethyl-1,3,2-dioxaborolan-2-yl)-2-(*m*-tolyl)-3,4-dihydroquinoline-1(2*H*)-carboxylate (2l):

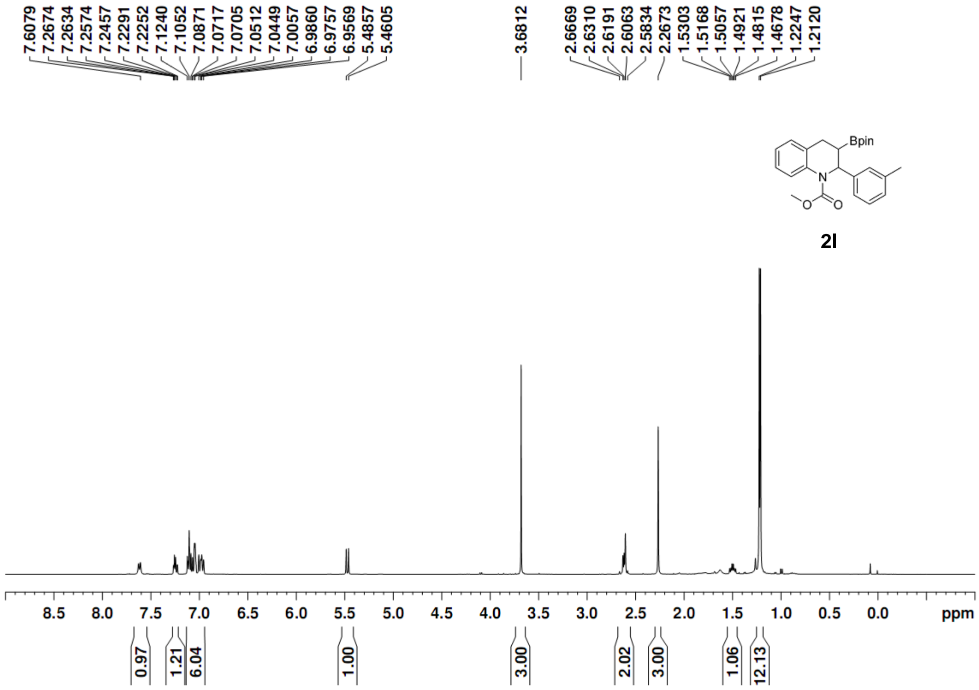

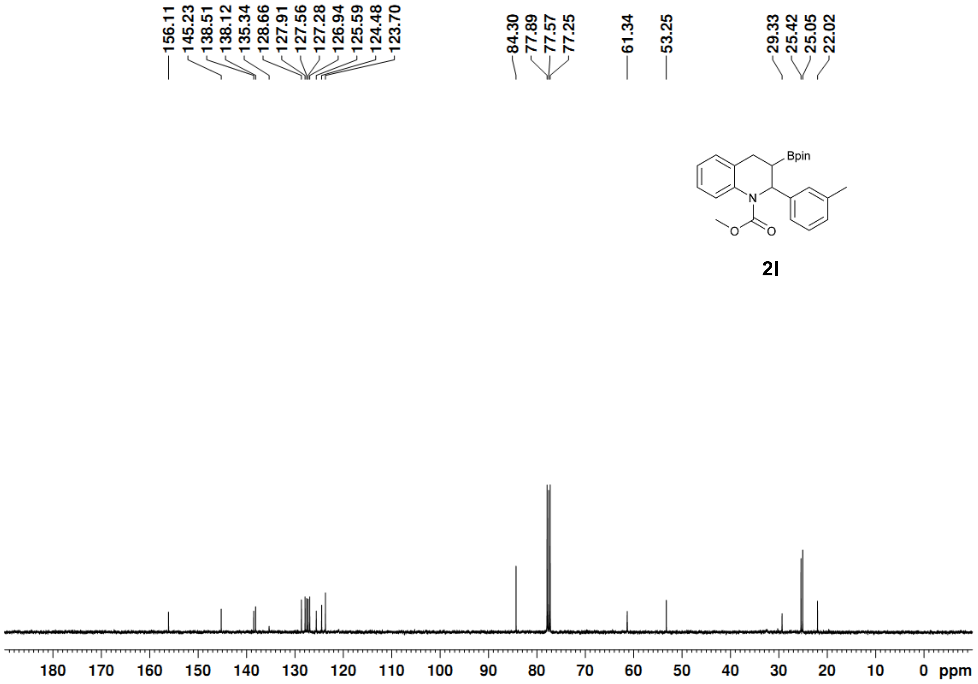

(*2R*,*3R*)-methyl 3-(4,4,5,5-tetramethyl-1,3,2-dioxaborolan-2-yl)-2-(*m*-tolyl)-3,4-dihydroquinoline-1(2*H*)-carboxylate (2m):

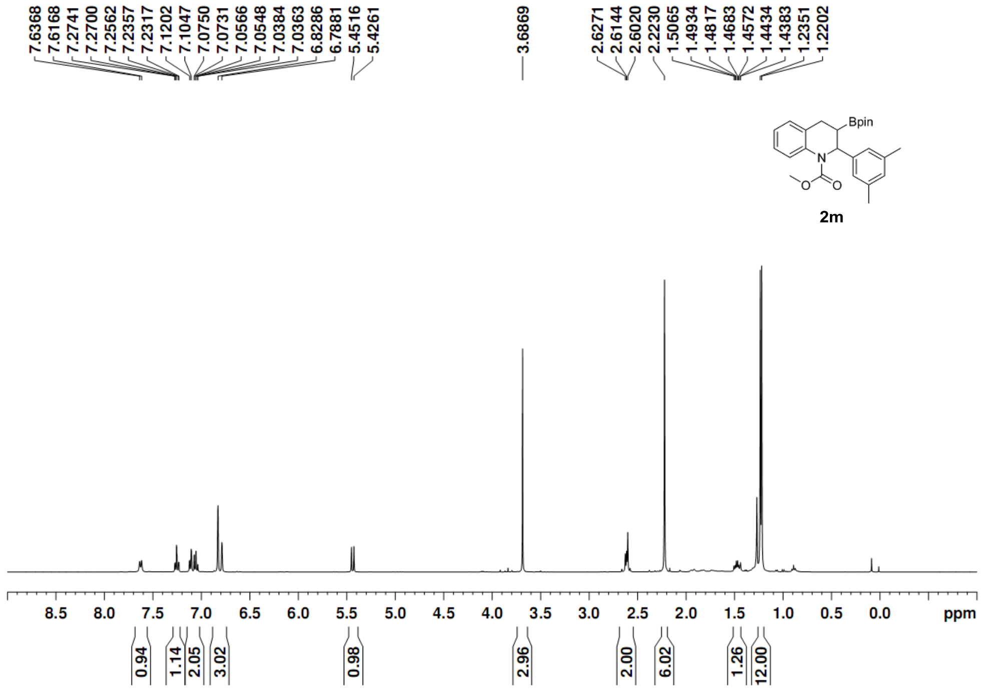

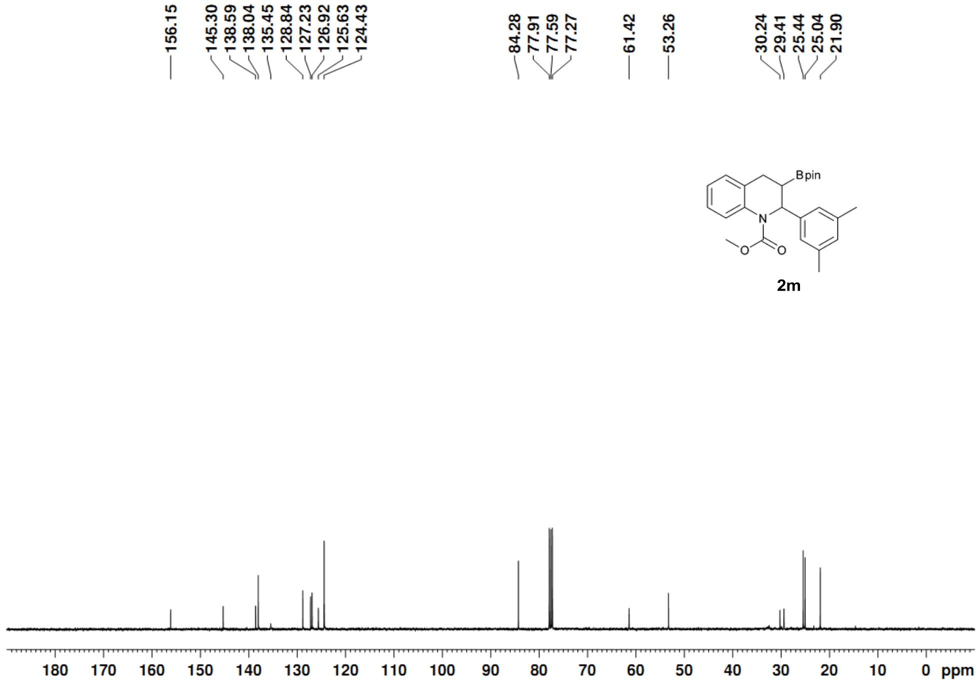

(2*R*,3*R*)-methyl 2-(3-chlorophenyl)-3-(4,4,5,5-tetramethyl-1,3,2-dioxaborolan-2-yl)-3,4-dihydroquinoline-1(2*H*)-carboxylate (2n):

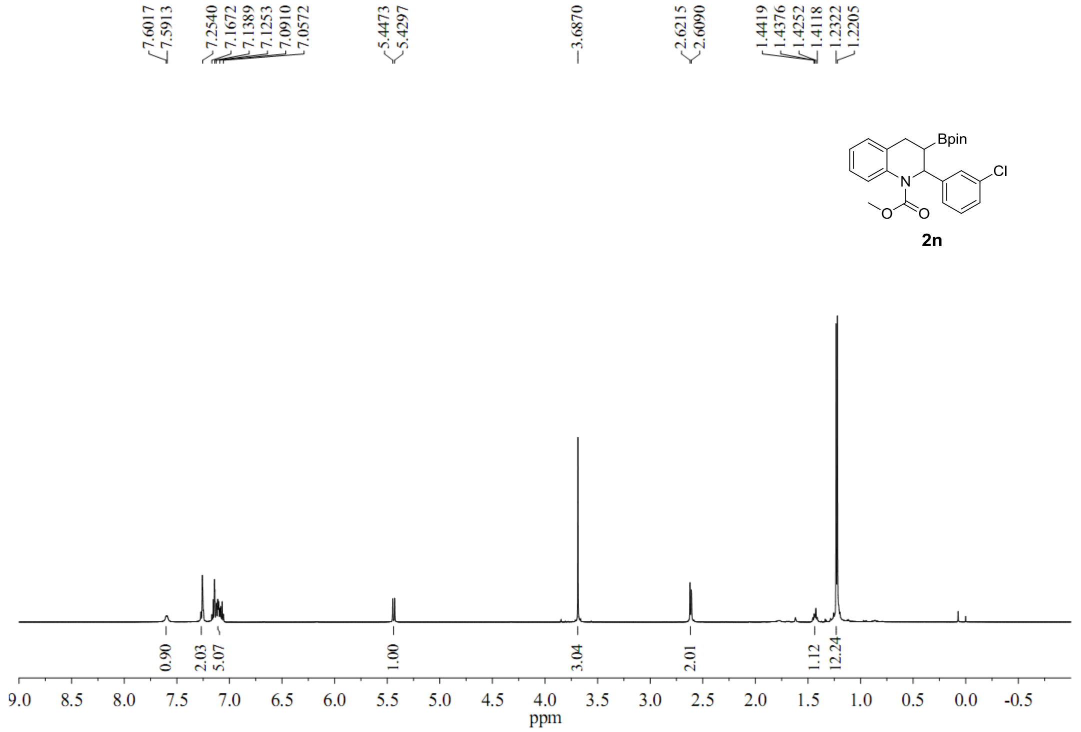

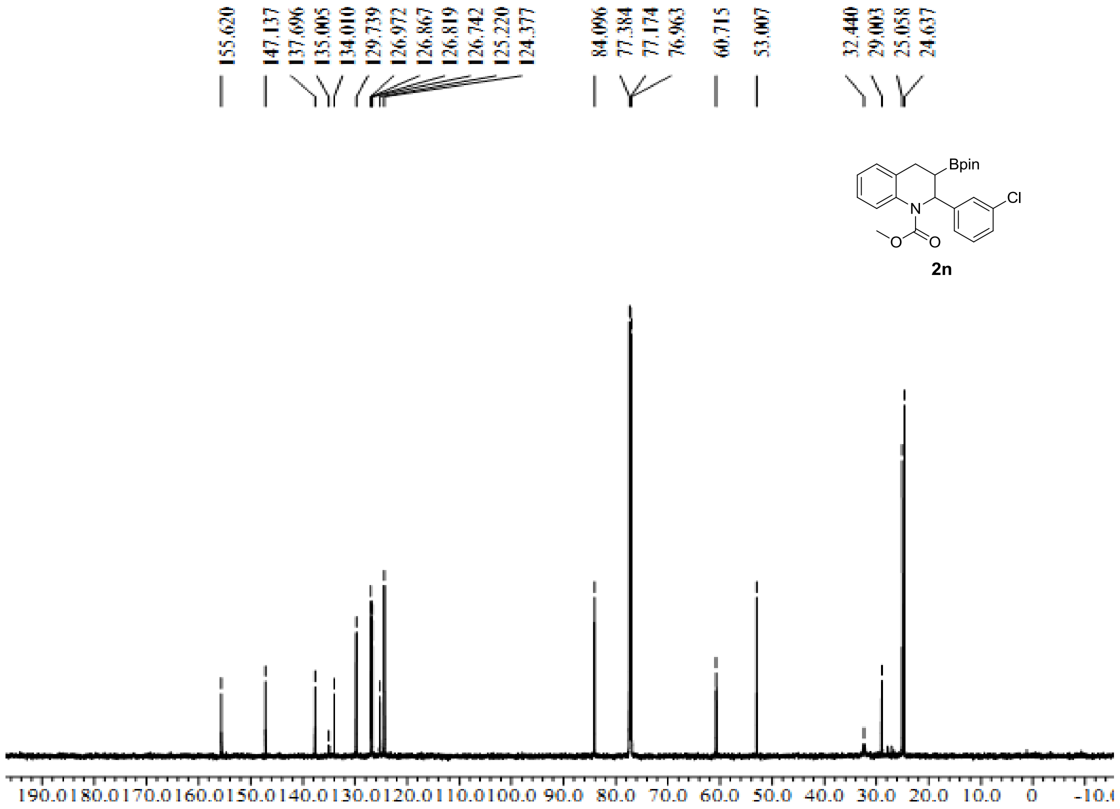

(2*R*,3*R*)-methyl 2-(4-chlorophenyl)-3-(4,4,5,5-tetramethyl-1,3,2-dioxaborolan-2-yl)-3,4-dihydroquinoline-1(2*H*)-carboxylate (2o):

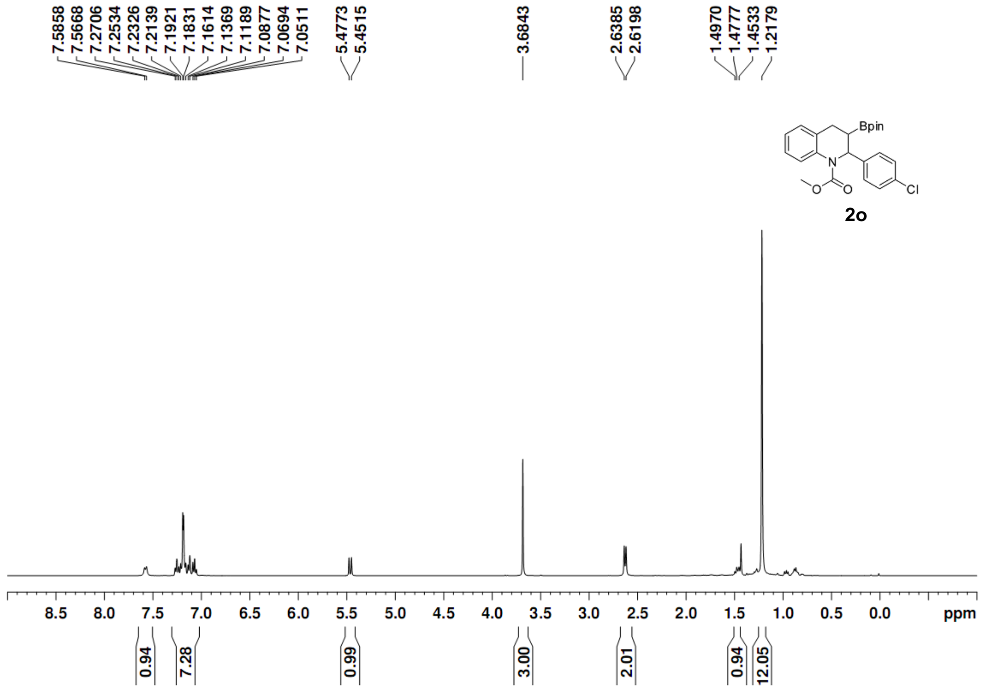

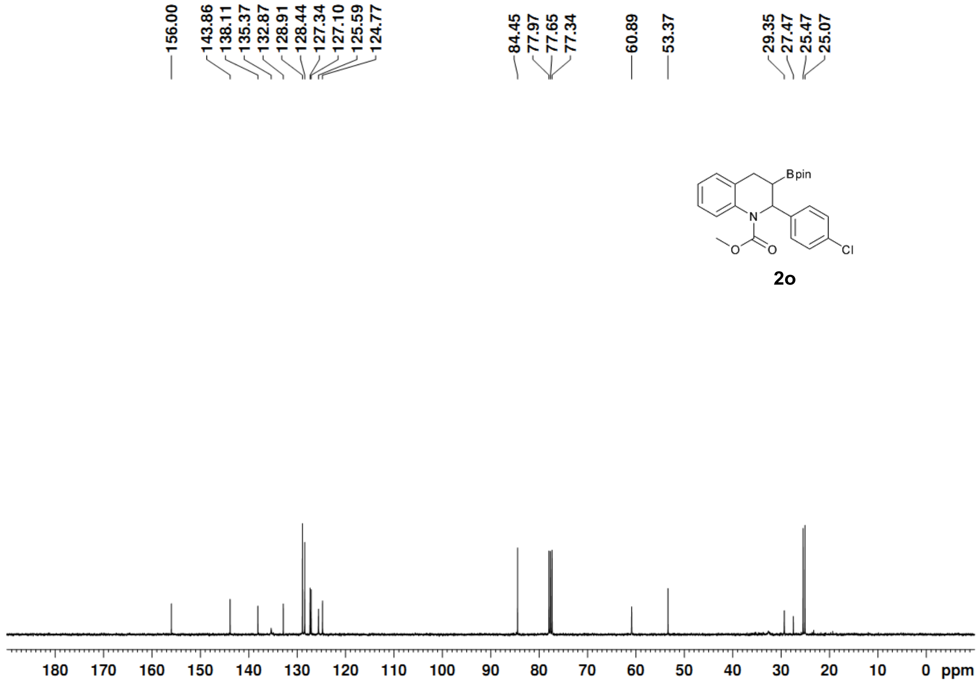

(2*R*,3*R*)-methyl 6-methoxy-2-(4-methoxyphenyl)-3-(4,4,5,5-tetramethyl-1,3,2-dioxaborolan-2-yl)-3,4-dihydroquinoline-1(2*H*)-carboxylate (2p):

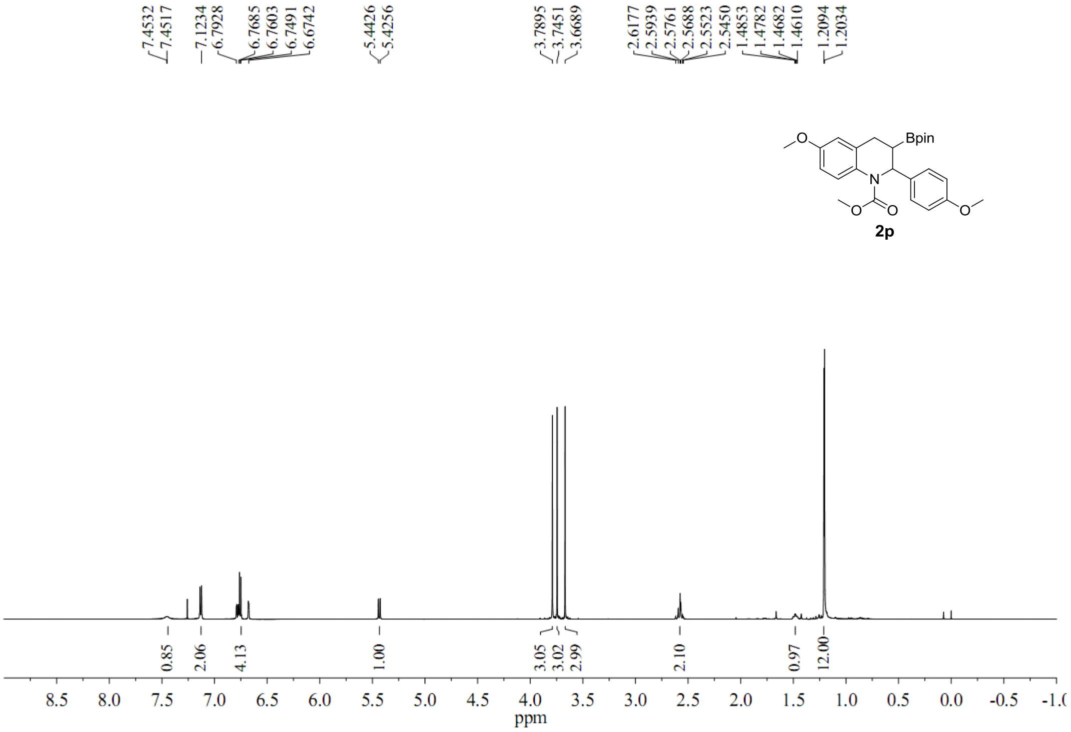

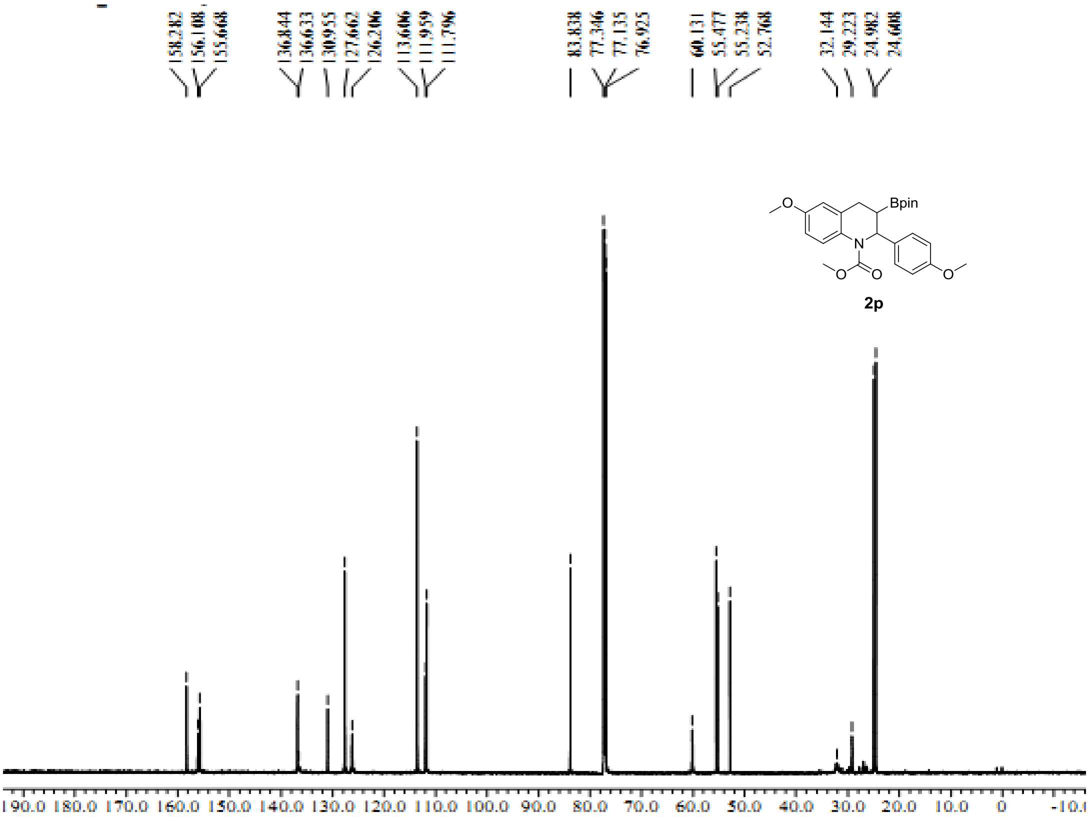

(*2S,3R*)-methyl 2-methyl-3-(4,4,5,5-tetramethyl-1,3,2-dioxaborolan-2-yl)-3,4-dihydroquinoline-1(2*H*)-carboxylate (2q):

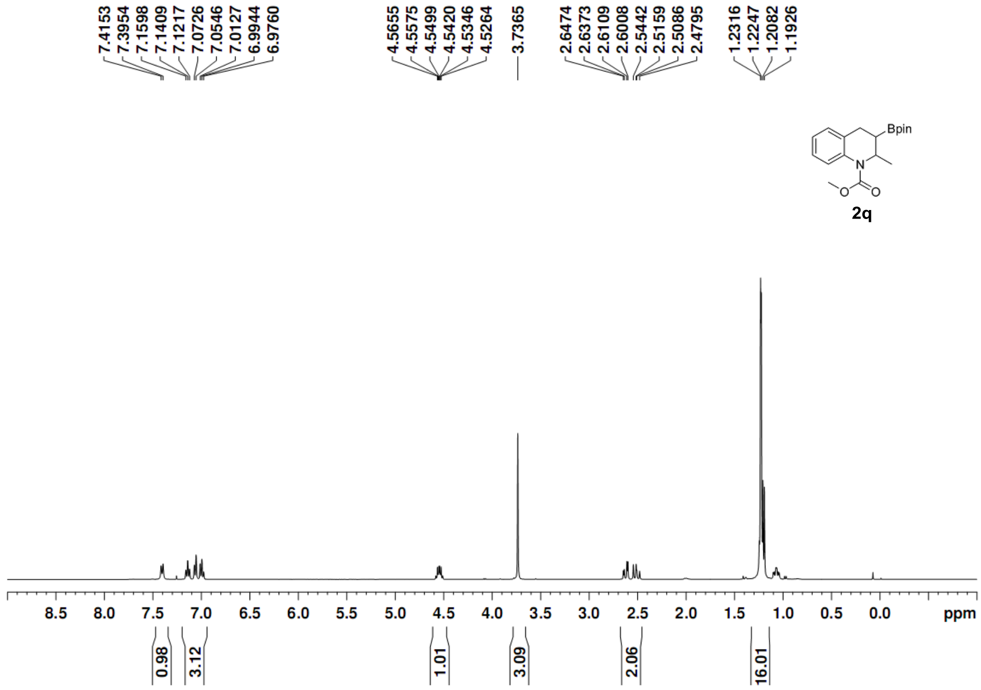

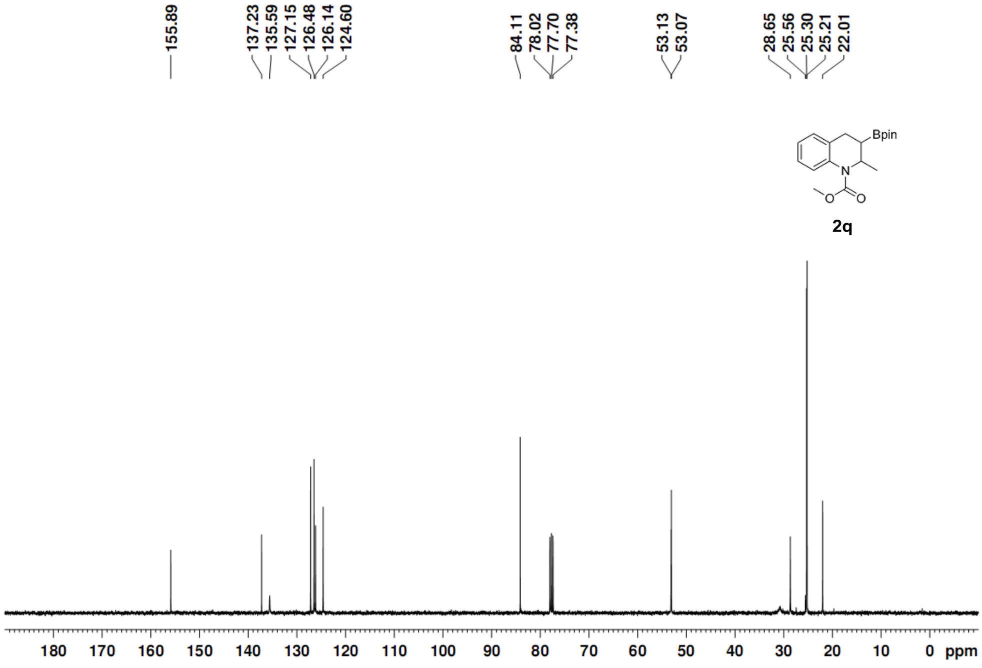

(2*S*,3*R*)-methyl 2-butyl-3-(4,4,5,5-tetramethyl-1,3,2-dioxaborolan-2-yl)-3,4-dihydroquinoline-1(2*H*)-carboxylate (2r):

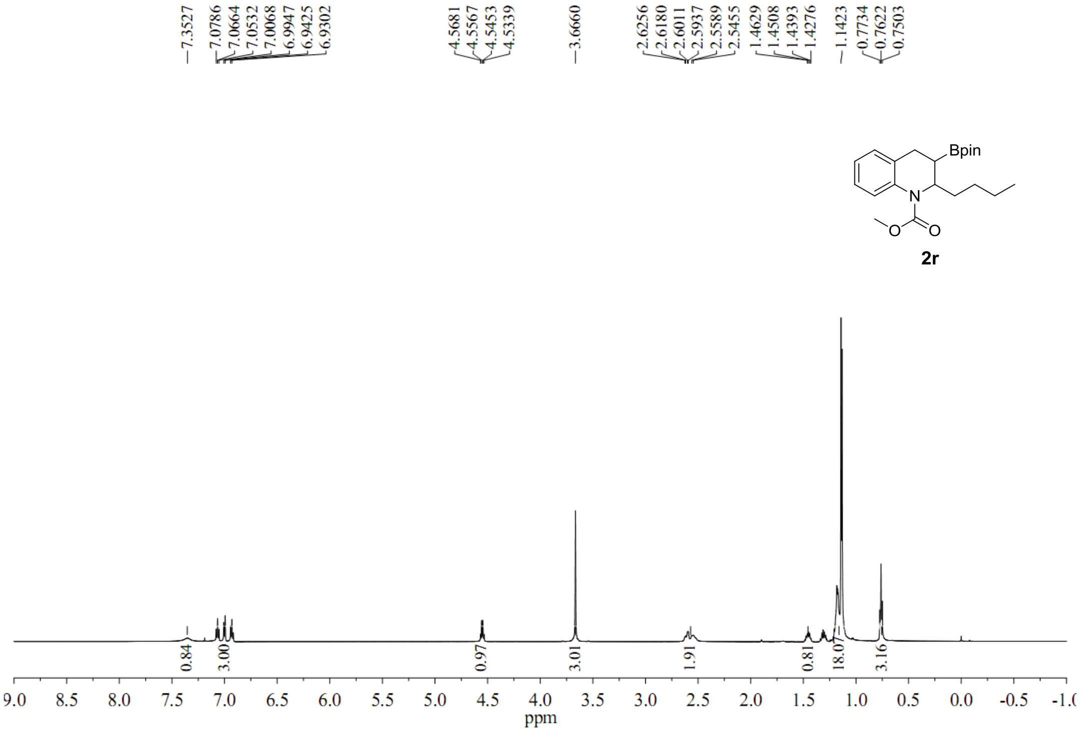

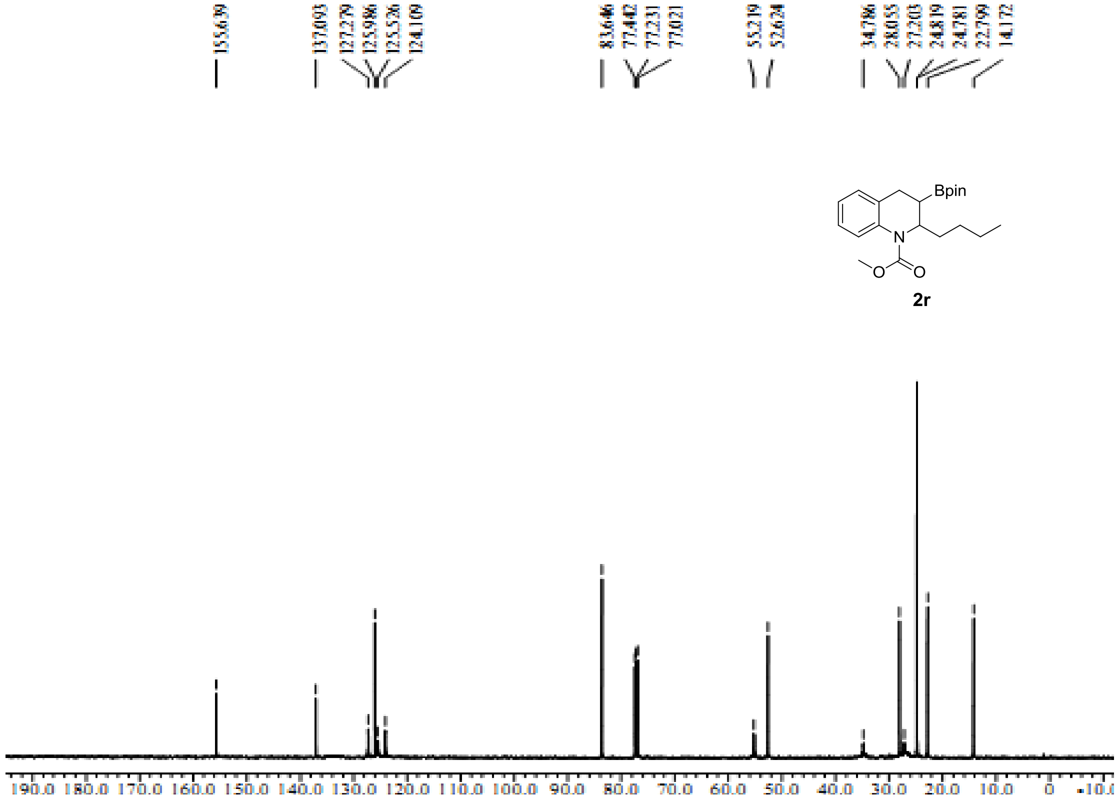

(2*S*,3*R*)- methyl 3-hydroxy-6-methoxy-2-(naphthalen-1-yl)-3,4-dihydroquinoline-1(2*H*)-carboxylate (3):

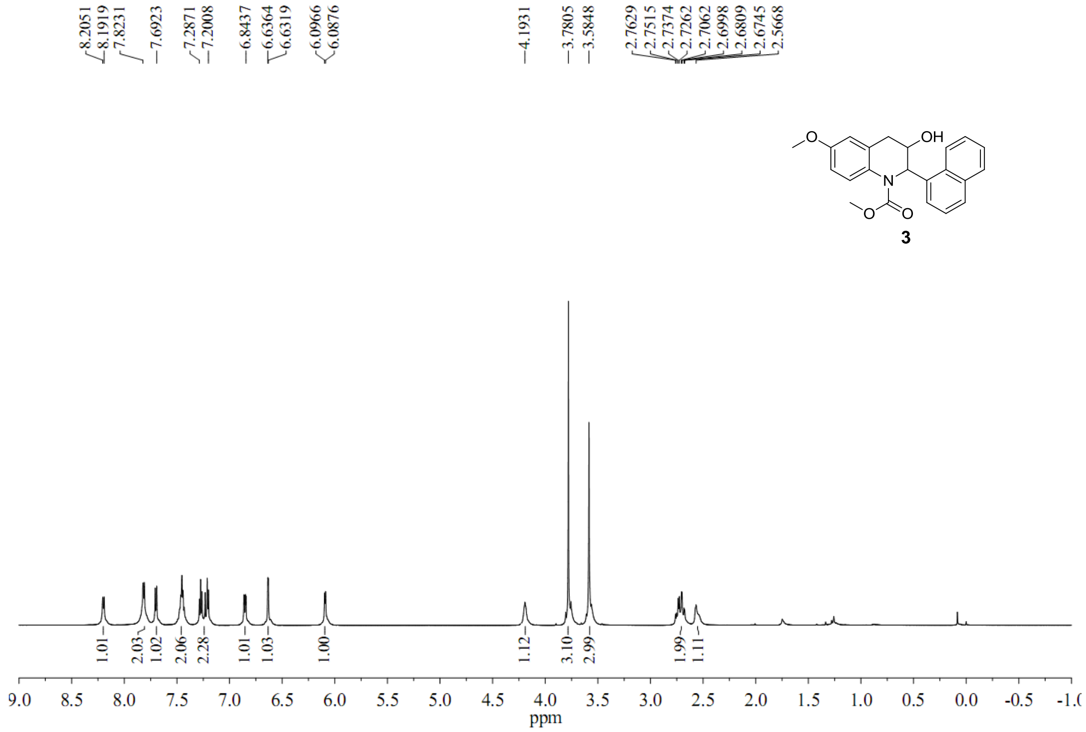

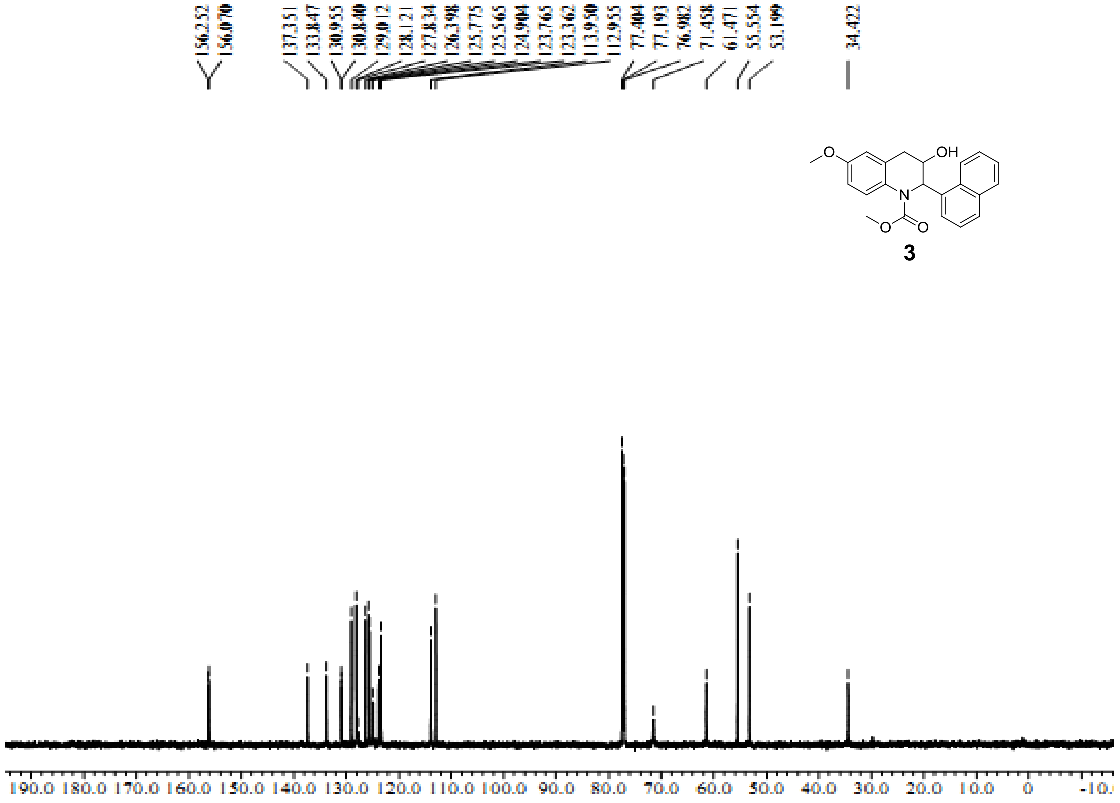

(*2S,3R*)-2-(naphthalen-1-yl)-1,2,3,4-tetrahydroquinolin-3-ol (4):

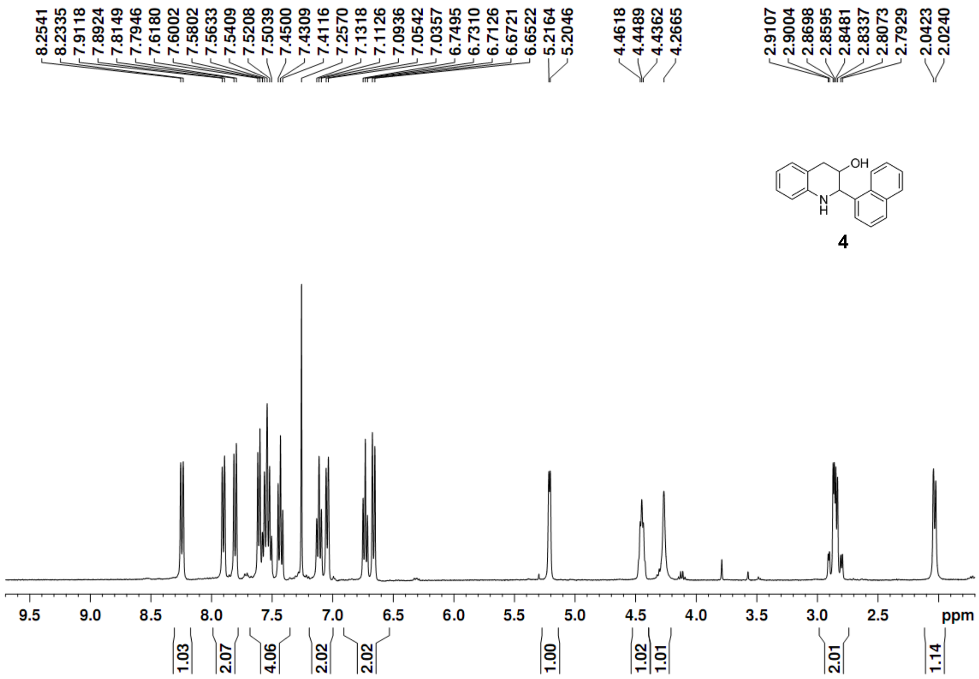

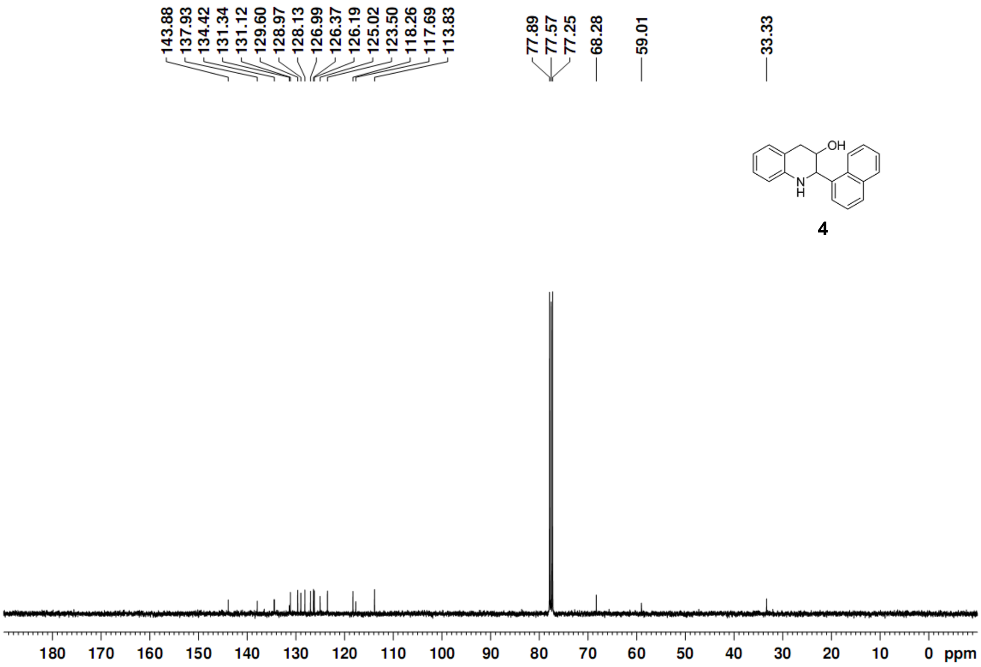

(*2R,3R*) -(1-(methoxycarbonyl)-2-(naphthalen-1-yl)-1,2,3,4-tetrahydroquinolin-3-yl) boronic acid (5):

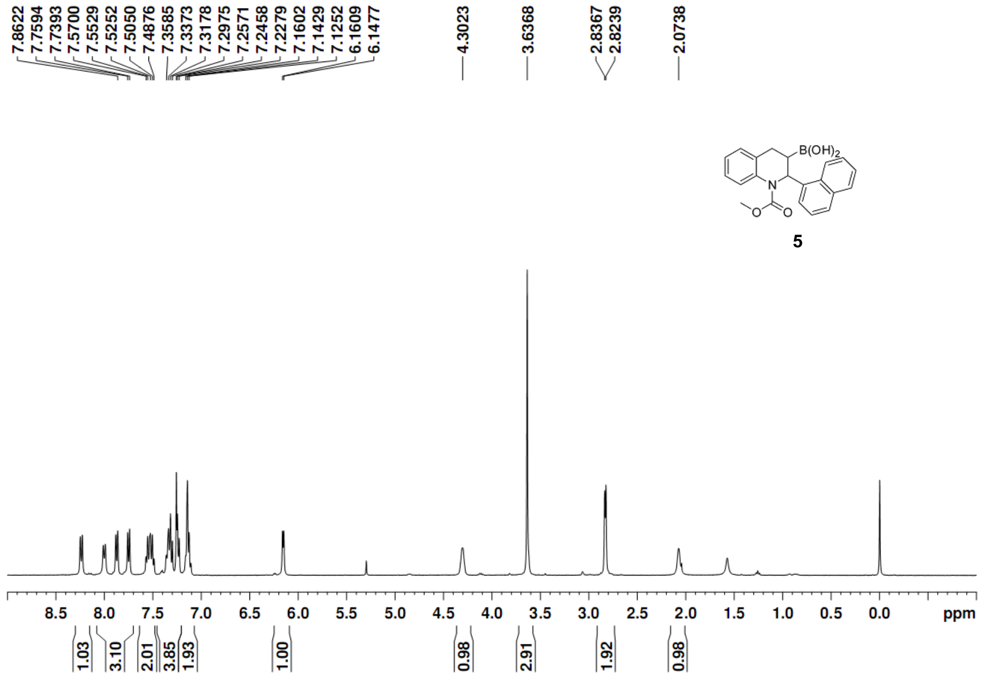

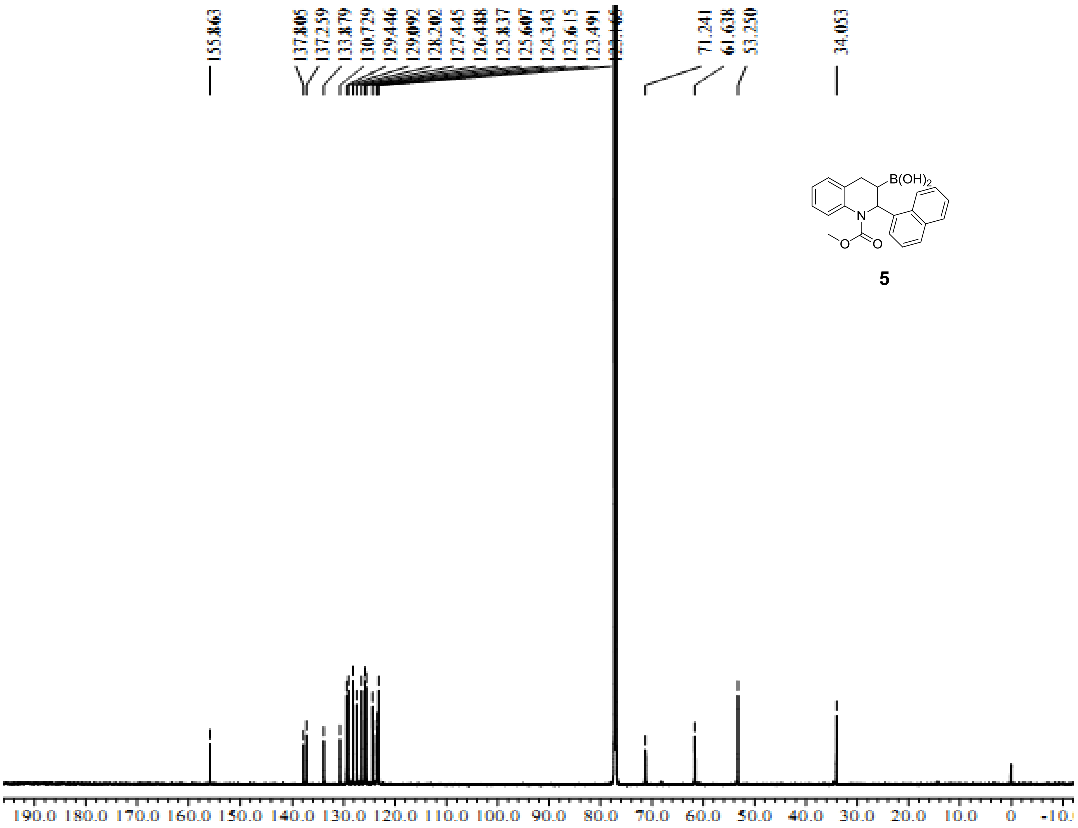

(2*R*,3*R*)-potassium (1-(methoxycarbonyl)-2-(naphthalen-1-yl)-1,2,3,4-tetrahydro-quinolin-3-yl) trifluoroborate (6):

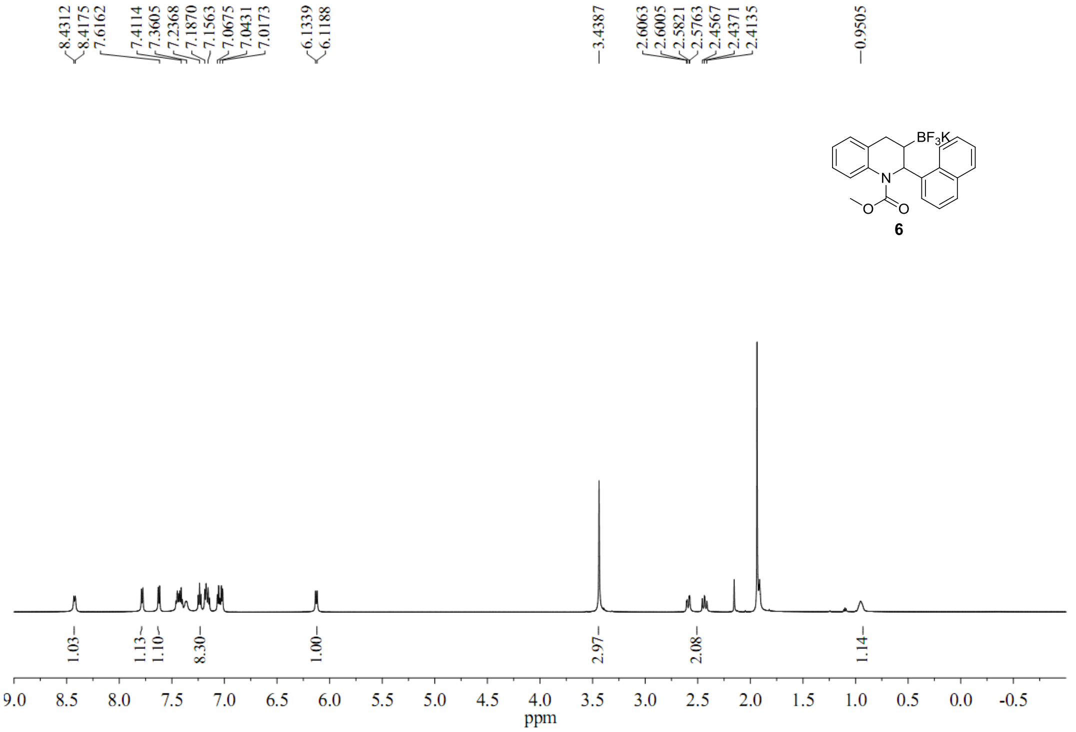

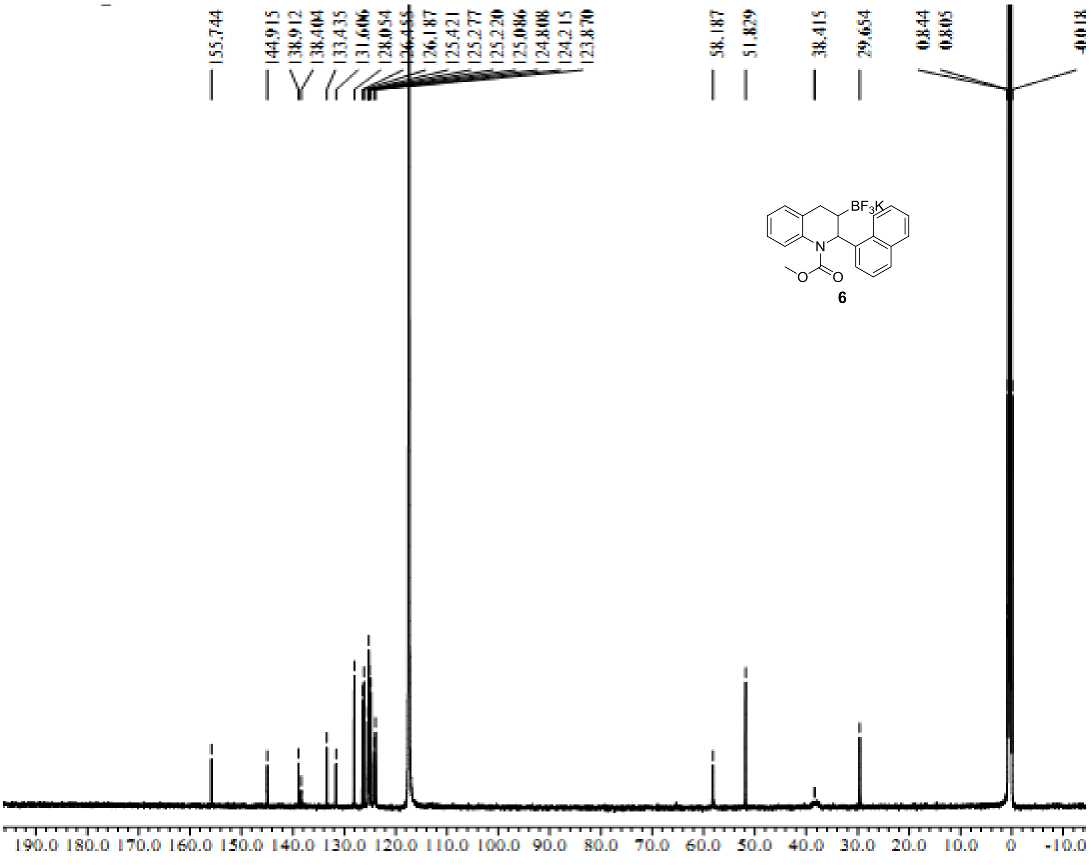

(2*R*,3*R*)-methyl 3-(hydroxymethyl)-2-(naphthalen-1-yl)-3,4-dihydroquinoline-1 (2*H*)-carboxylate (7):

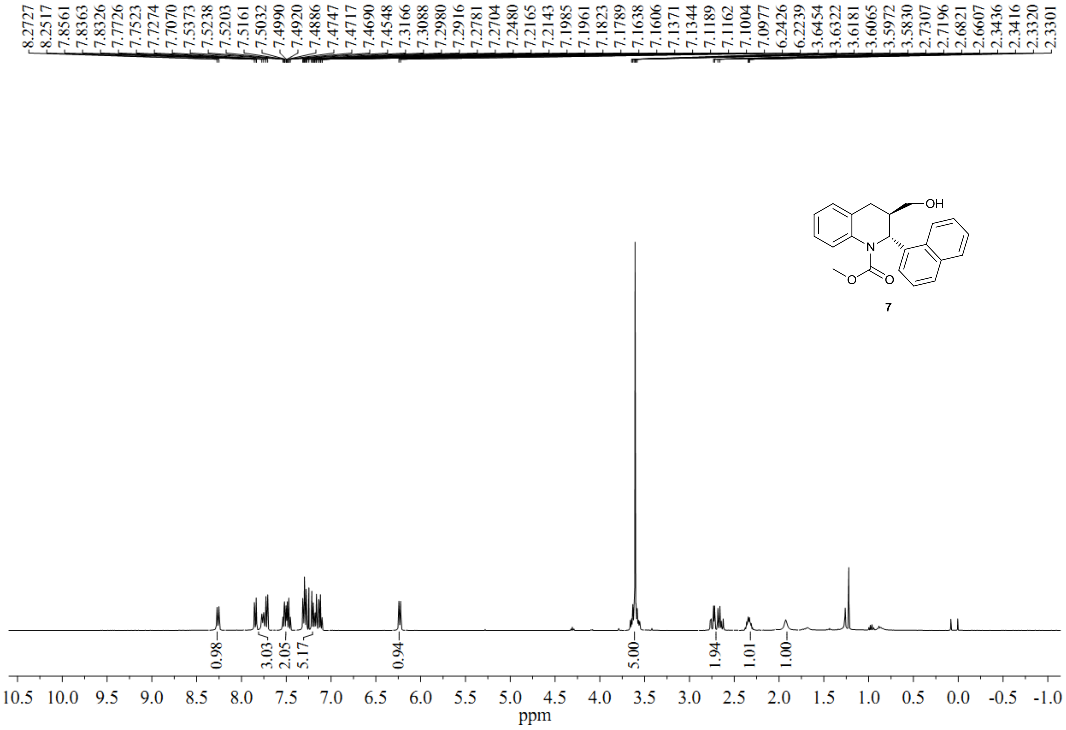

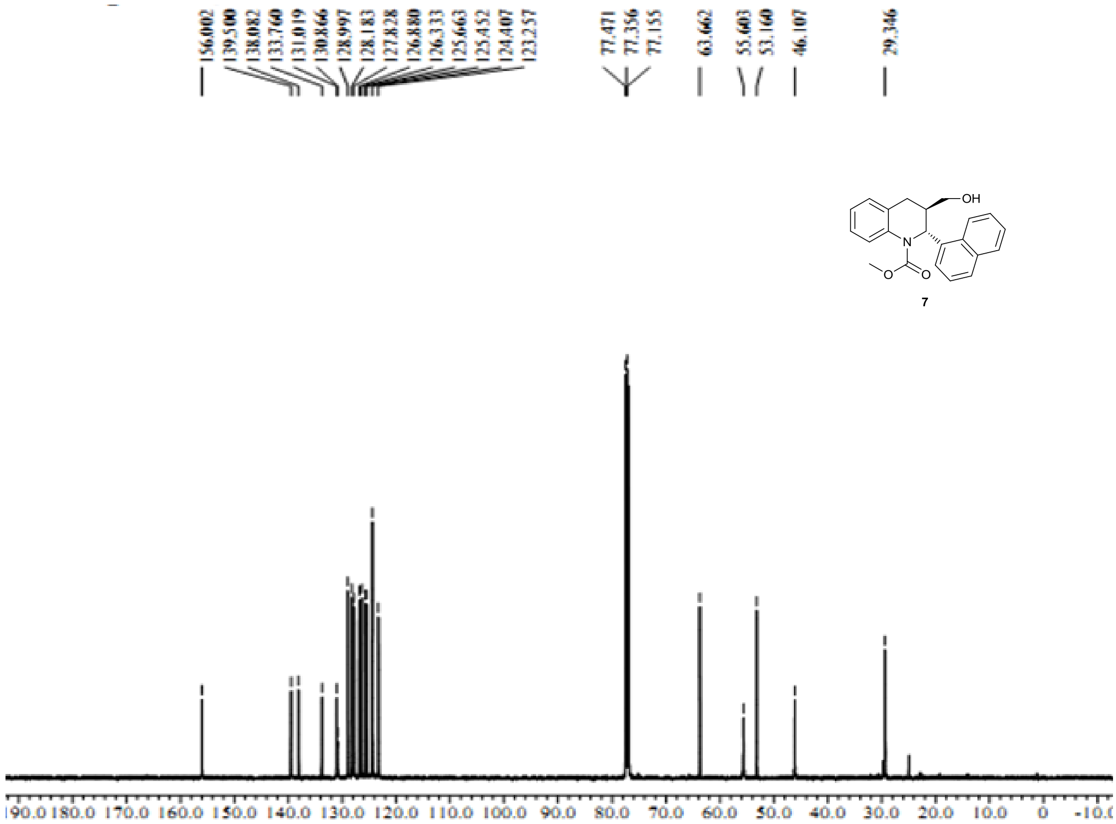

(2*R*,3*S*)-methyl 3-(3-methoxyphenyl)-2-(naphthalen-1-yl)-3,4-dihydroquinoline-1 (2*H*)-carboxylate (8):

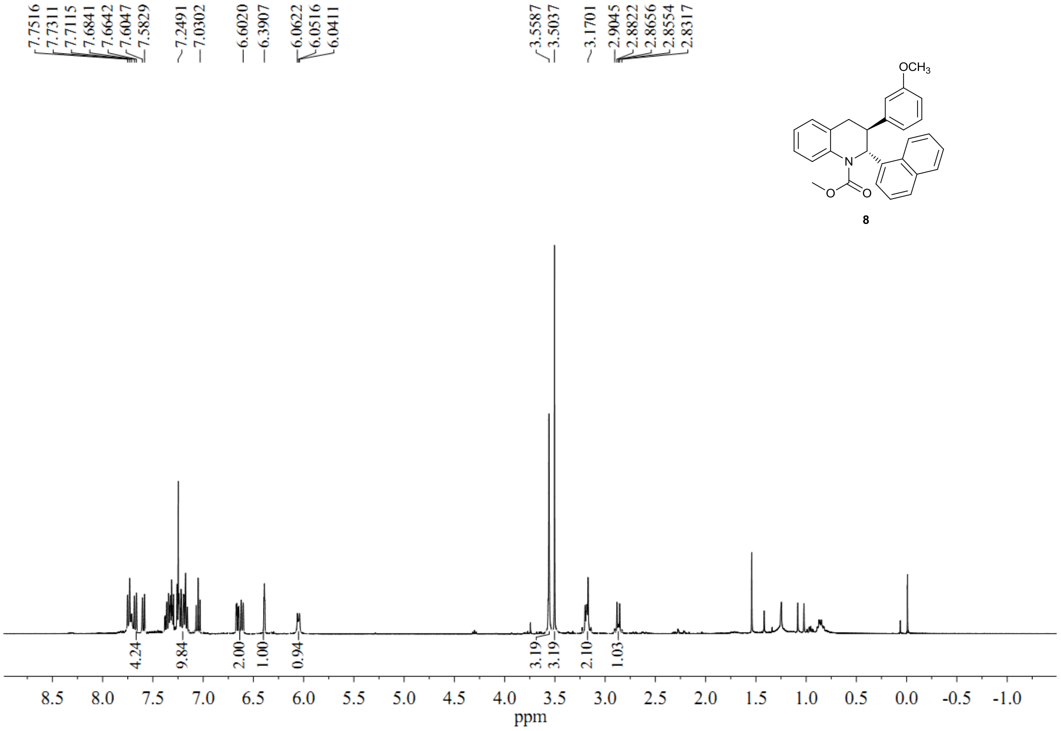

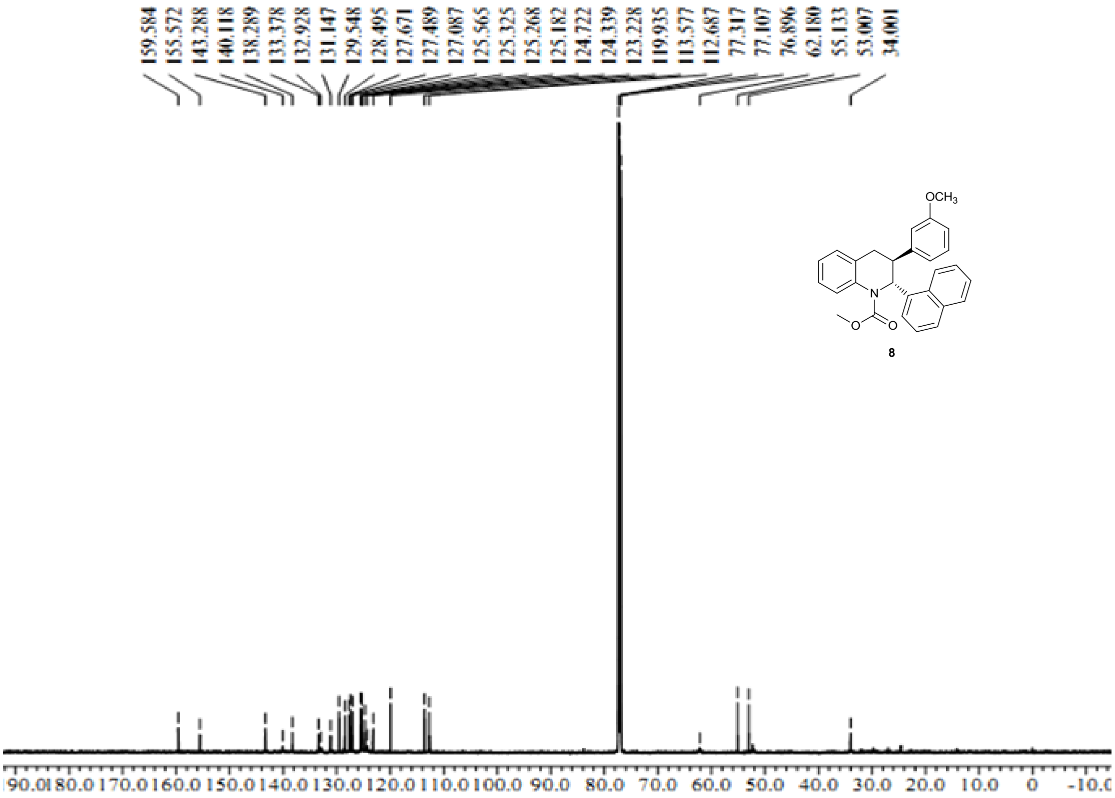

(*S*)-methyl 6-methoxy-2-(4-methoxyphenyl)-3,4-dihydroquinoline-1(2*H*)-carboxylate (9):

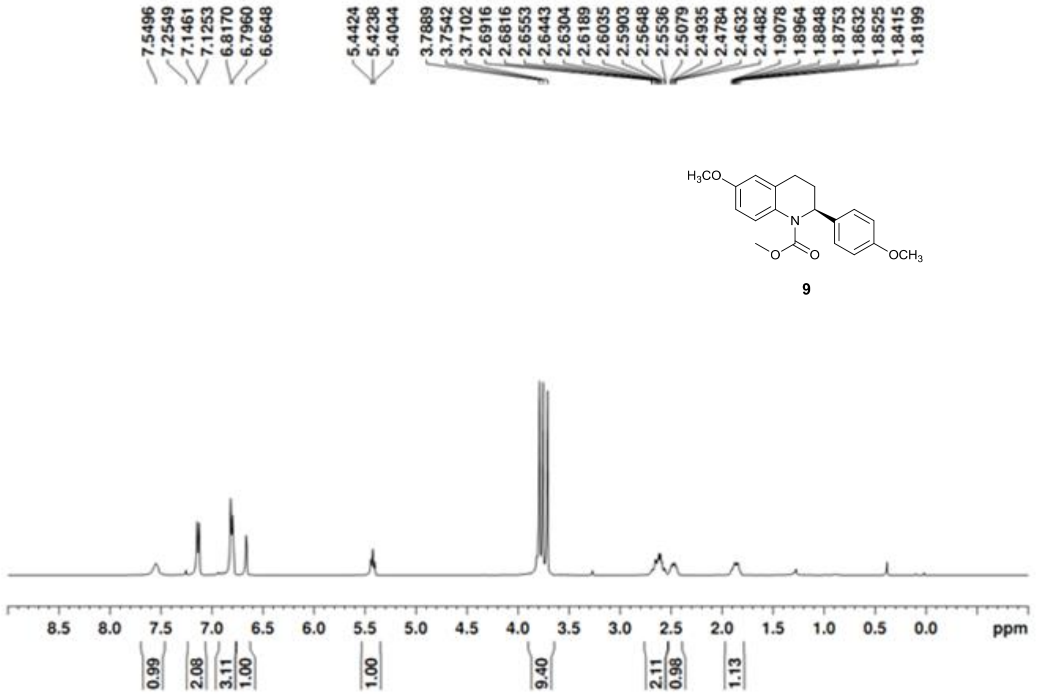

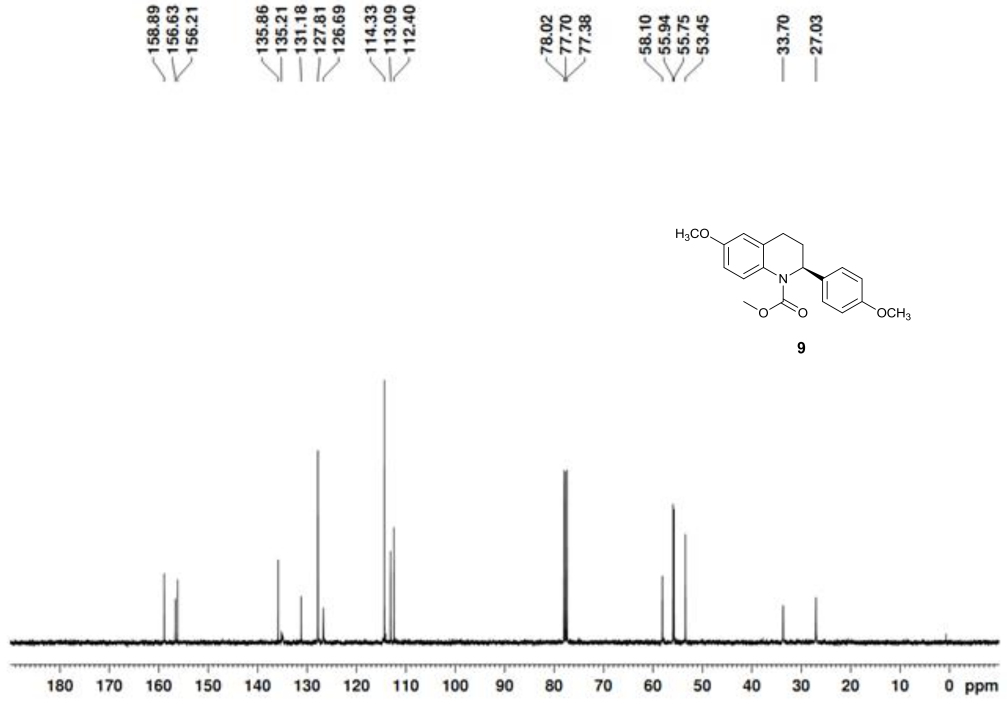

7. SFC and HPLC spectra of all compounds

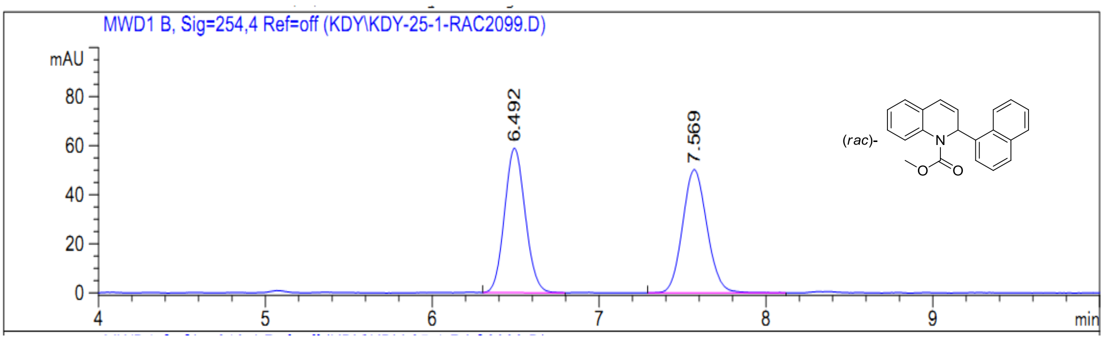

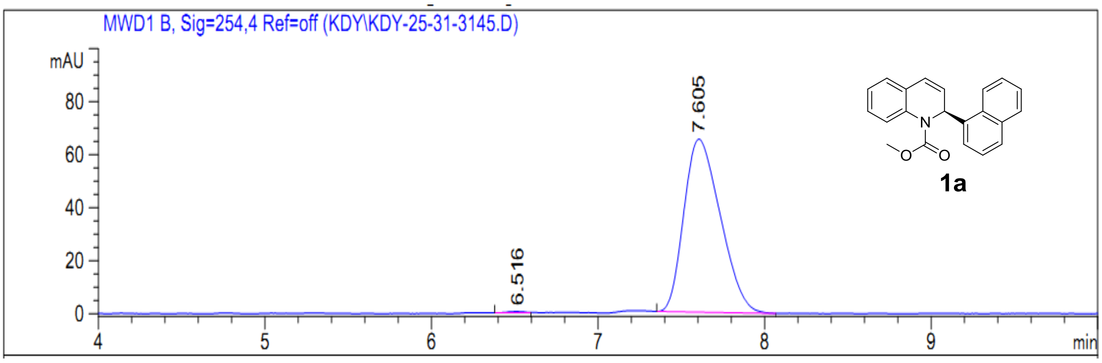

Peak #	RetTime [min]	Type	Width [min]	Area [mAU*s]	Height [mAU]	Area %
1	6.516	BV	0.0891	3.04038	4.09755e-1	0.3053
2	7.605	BB	0.2294	992.67297	65.27299	99.6947

Totals : 995.71335 65.68275

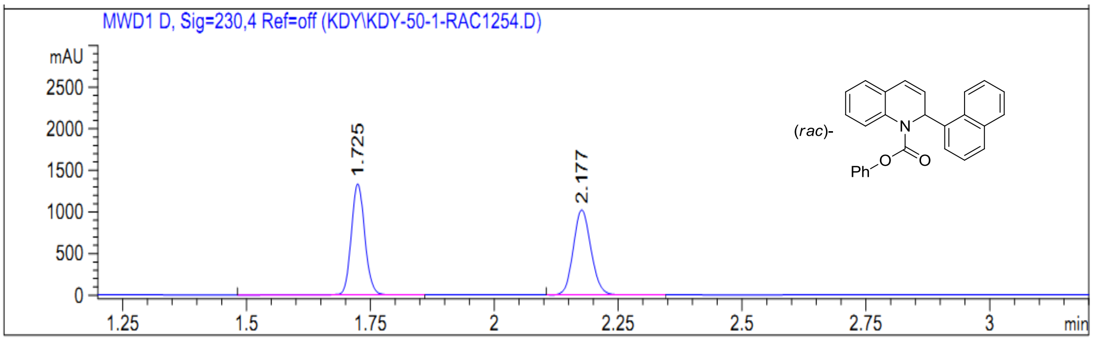

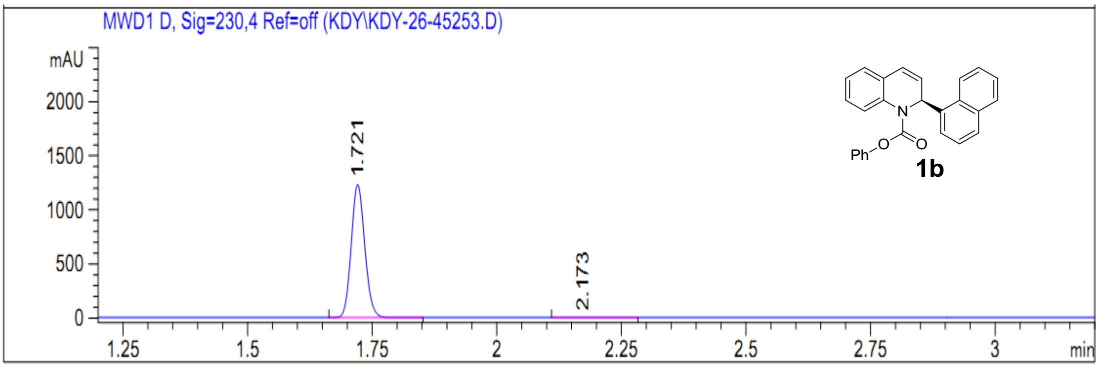

Peak #	RetTime [min]	Type	Width [min]	Area [mAU*s]	Height [mAU]	Area %
1	1.721	BV R	0.0290	2317.33032	1229.18518	99.7687
2	2.173	BB	0.0422	5.37300	1.97892	0.2313

Totals : 2322.70332 1231.16410

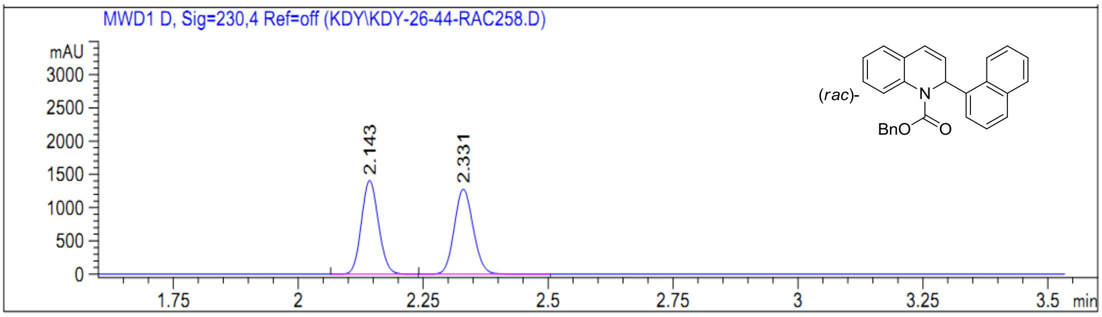

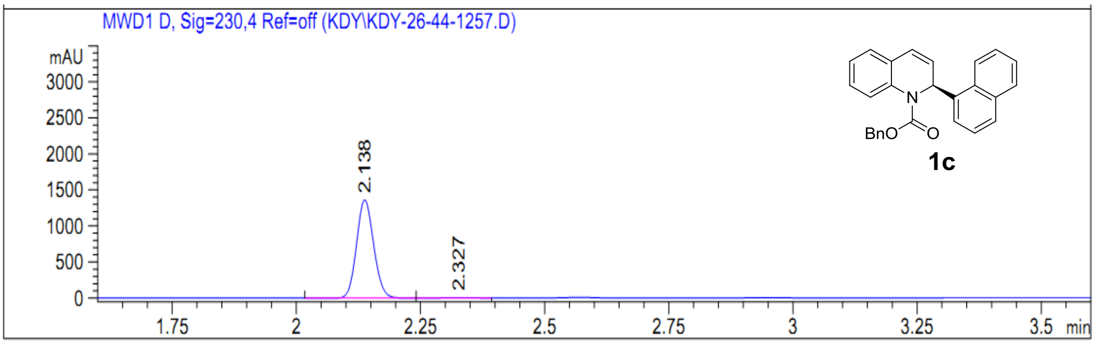

Signal 3: MWD1 D, Sig=230,4 Ref=off

Peak #	RetTime [min]	Type	Width [min]	Area [mAU*s]	Height [mAU]	Area %
1	2.138	VV R	0.0368	3241.92139	1361.82837	99.3613
2	2.327	VB	0.0409	20.84065	7.73643	0.6387

Totals : 3262.76204 1369.56480

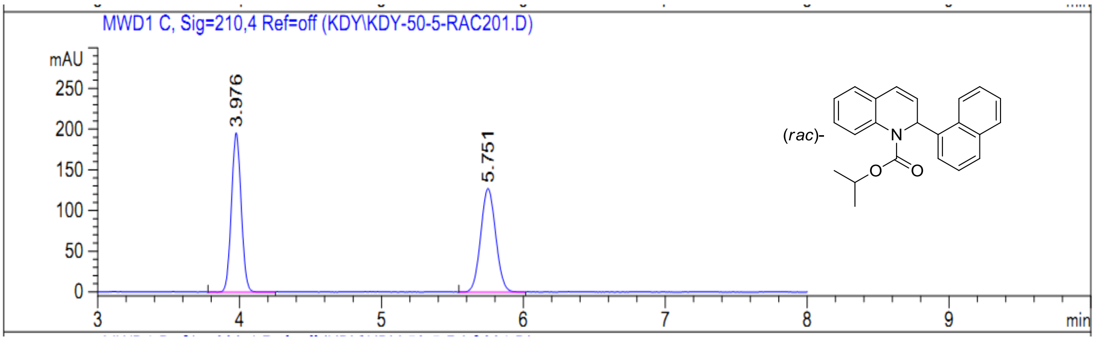

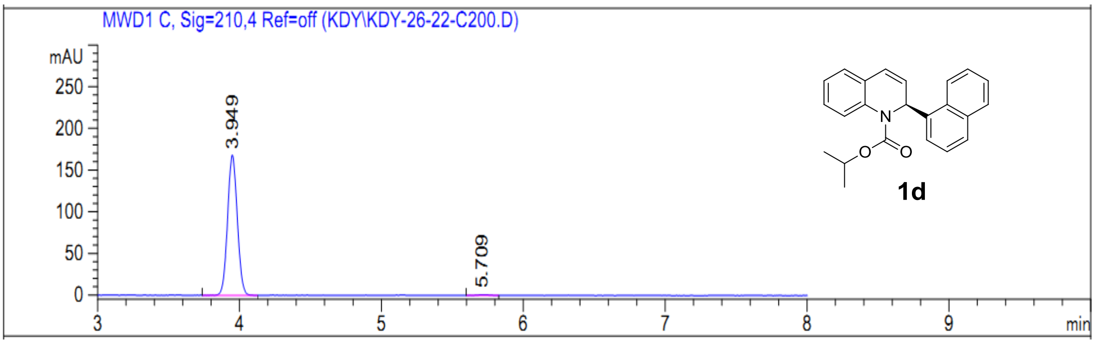

Signal 2: MWD1 C, Sig=210,4 Ref=off

Peak #	RetTime [min]	Type	Width [min]	Area [mAU*s]	Height [mAU]	Area %
1	3.949	BB	0.0740	807.14948	168.21577	99.2005
2	5.709	BB	0.0866	6.50504	9.19135e-1	0.7995

Totals : 813.65451 169.13491

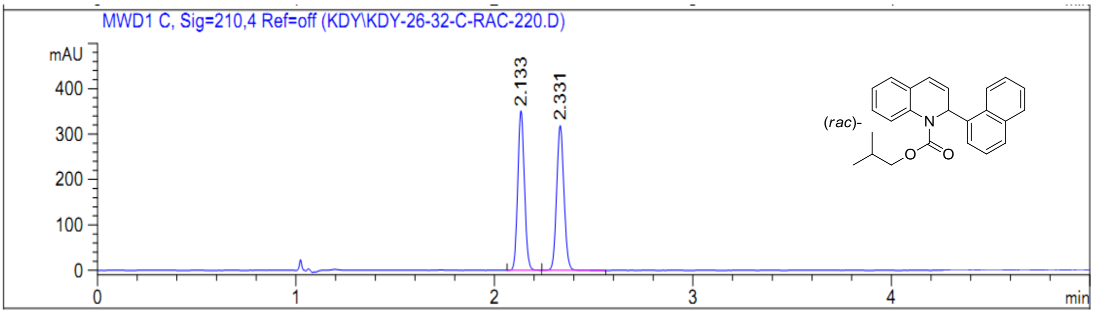

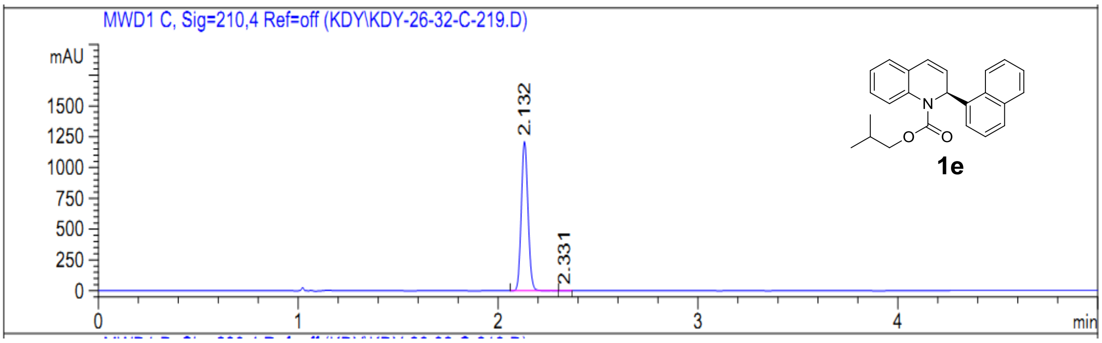

Peak #	RetTime [min]	Type	Width [min]	Area [mAU*s]	Height [mAU]	Area %
1	2.132	BV R	0.0374	2827.12524	1202.76563	99.9425
2	2.331	BB	0.0404	1.62646	6.68677e-1	0.0575

Totals : 2828.75170 1203.43430

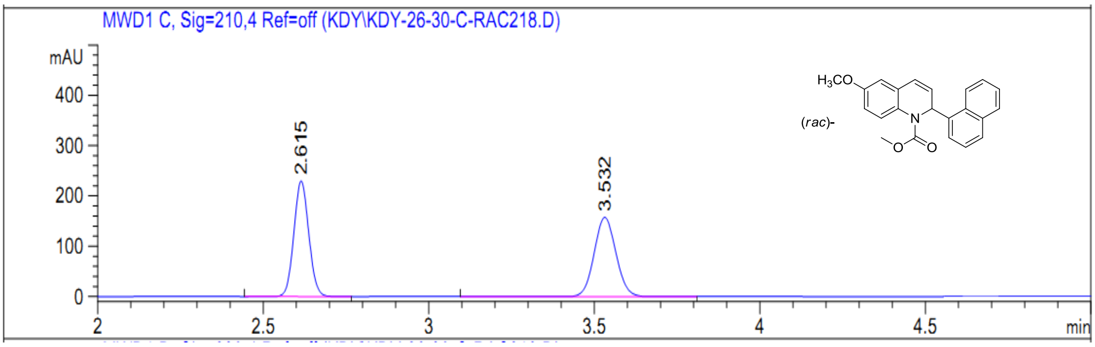

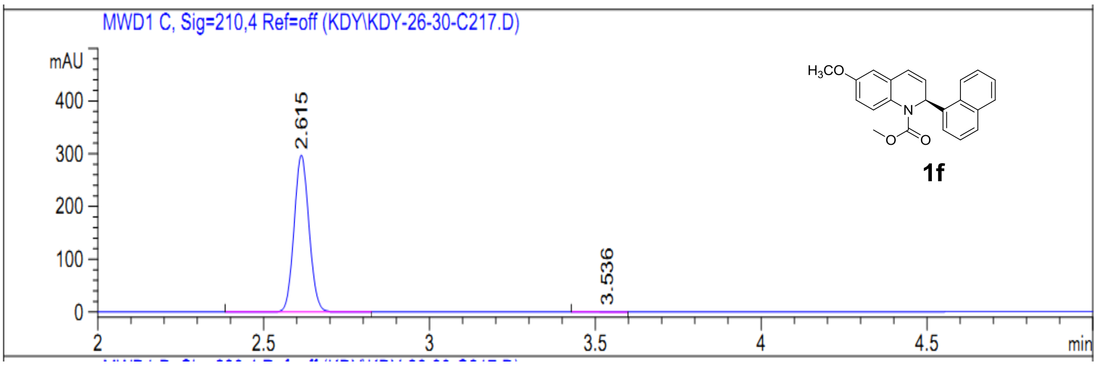

Peak #	RetTime [min]	Type	Width [min]	Area [mAU*s]	Height [mAU]	Area %
1	2.615	VV R	0.0487	933.03027	295.72791	99.6700
2	3.536	VV	0.0841	3.08945	4.86691e-1	0.3300

Totals : 936.11973 296.21460

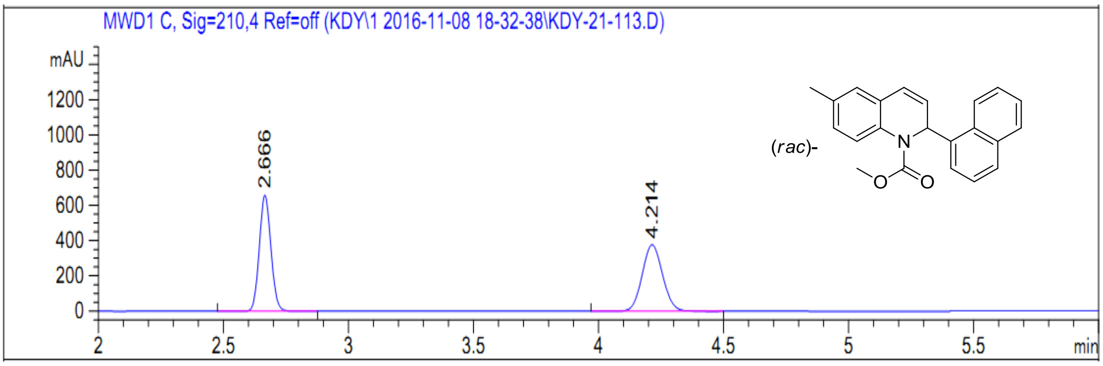

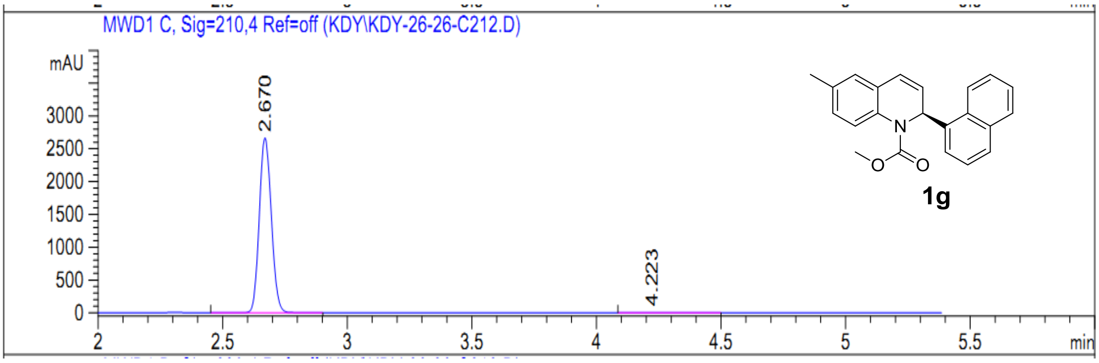

Peak #	RetTime [min]	Type	Width [min]	Area [mAU*s]	Height [mAU]	Area %
1	2.670	VB R	0.0526	8920.10449	2662.62915	99.6092
2	4.223	BV R	0.0898	34.99500	5.89195	0.3908

Totals : 8955.09949 2668.52110

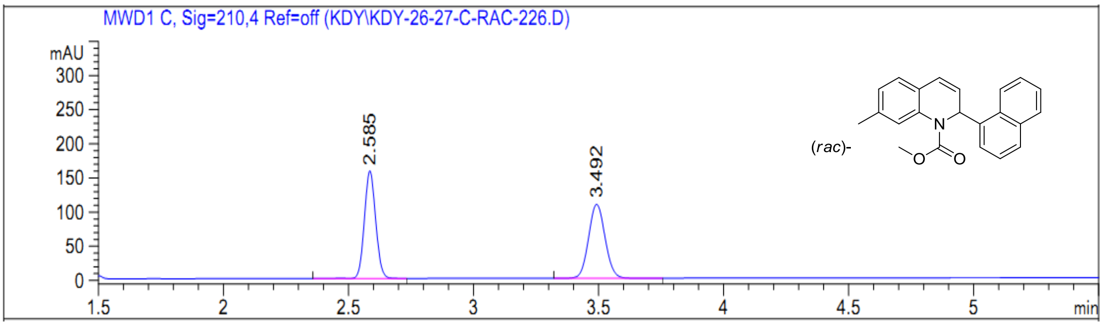

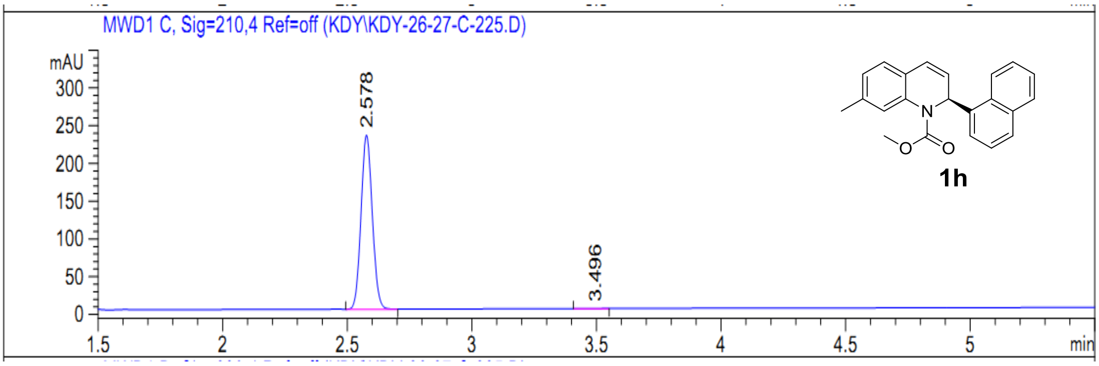

Peak #	RetTime [min]	Type	Width [min]	Area [mAU*s]	Height [mAU]	Area %
1	2.578	BB	0.0487	725.20166	230.80426	99.6500
2	3.496	BV	0.0618	2.54709	5.10098e-1	0.3500

Totals : 727.74875 231.31436

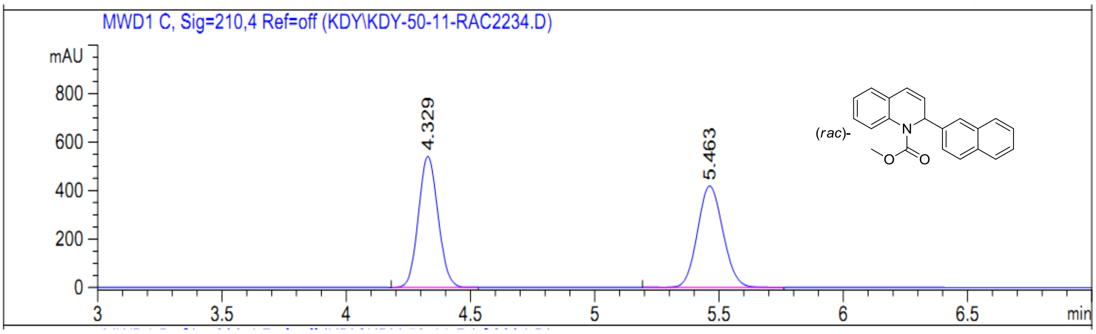

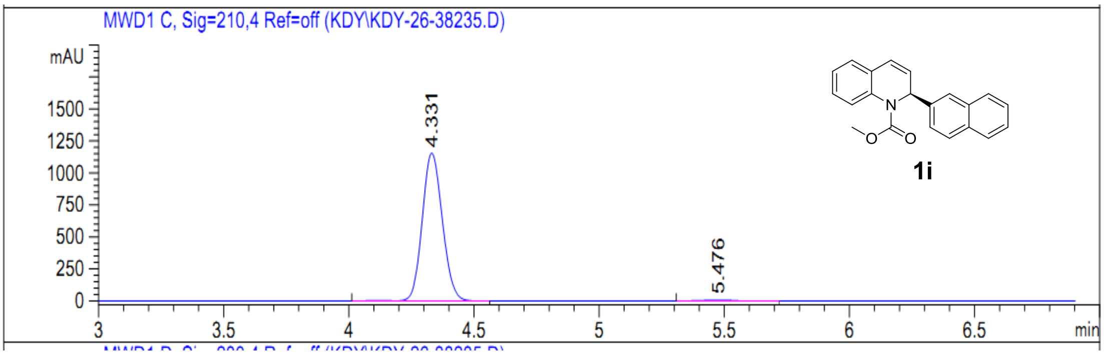

Peak #	RetTime [min]	Type	Width [min]	Area [mAU*s]	Height [mAU]	Area %
1	4.331	VB R	0.0865	6431.01563	1154.85425	99.3195
2	5.476	BV R	0.1060	44.06280	5.87289	0.6805

Totals : 6475.07842 1160.72714

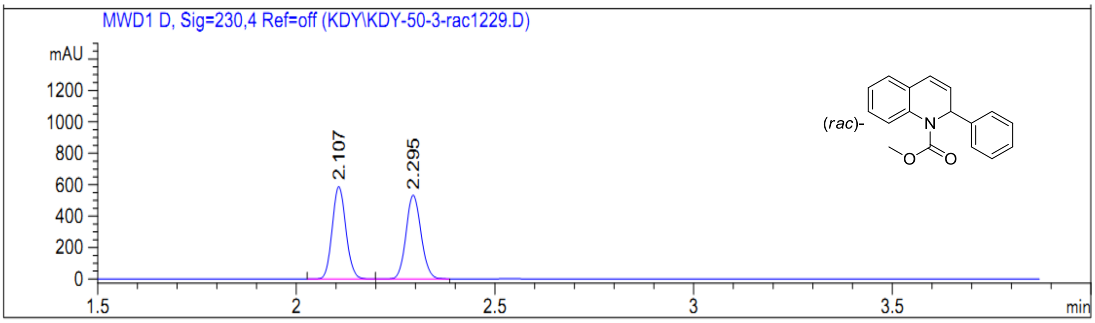

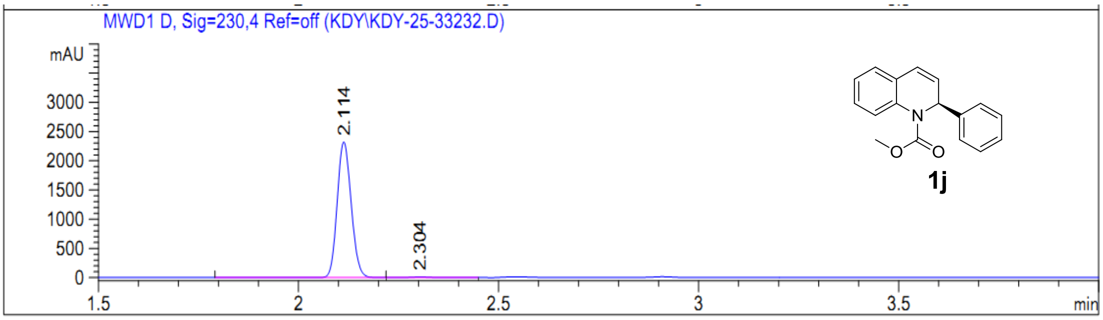

Peak #	RetTime [min]	Type	Width [min]	Area [mAU*s]	Height [mAU]	Area %
1	2.114	VV R	0.0378	5638.04639	2321.15942	99.3370
2	2.304	VV R	0.0424	37.62998	13.77411	0.6630

Totals : 5675.67636 2334.93353

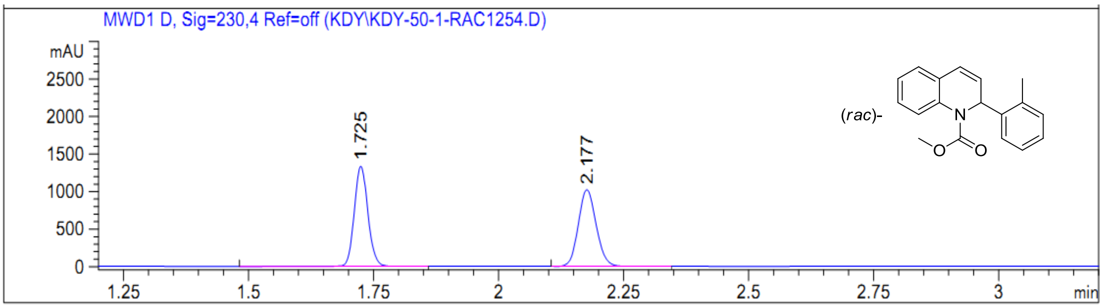

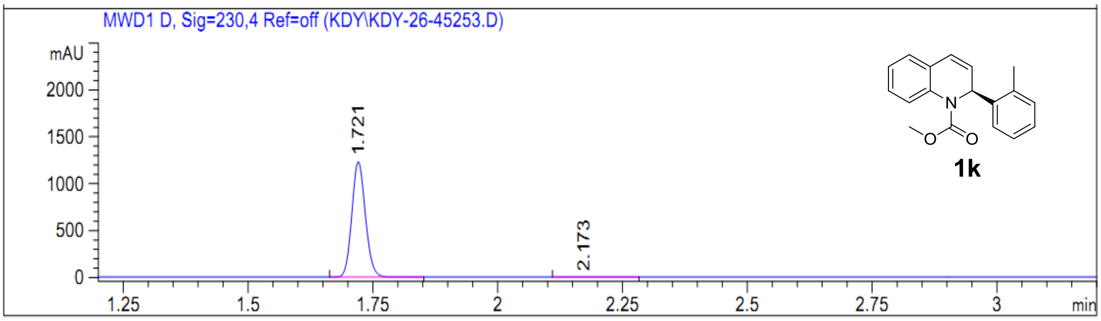

Peak #	RetTime [min]	Type	Width [min]	Area [mAU*s]	Height [mAU]	Area %
1	1.721	BV R	0.0290	2317.33032	1229.18518	99.7687
2	2.173	BB	0.0422	5.37300	1.97892	0.2313
Totals :				2322.70332	1231.16410	

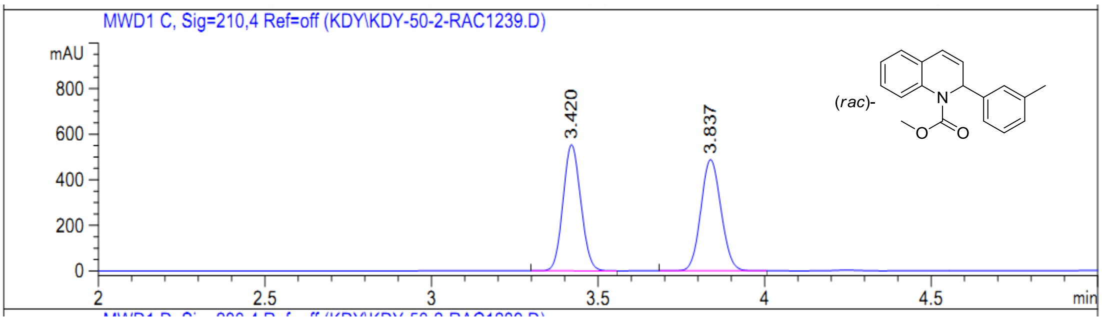

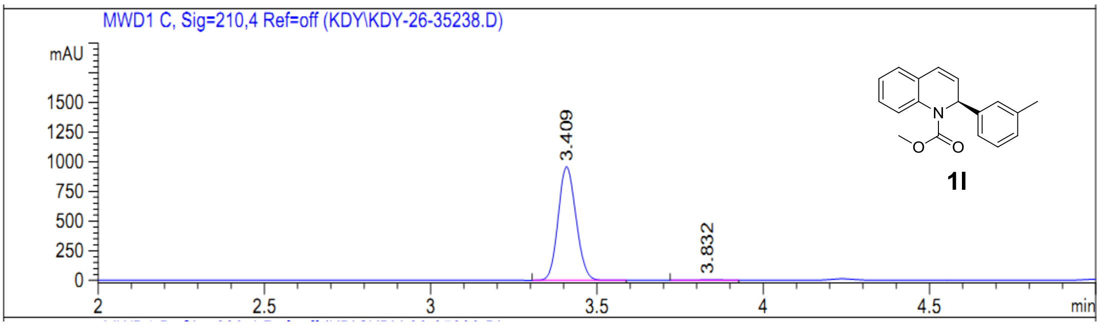

Peak #	RetTime [min]	Type	Width [min]	Area [mAU*s]	Height [mAU]	Area %
1	3.409	BV R	0.0585	3573.56274	958.04547	99.4033
2	3.832	VB R	0.0660	21.45264	5.00184	0.5967

Totals : 3595.01539 963.04731

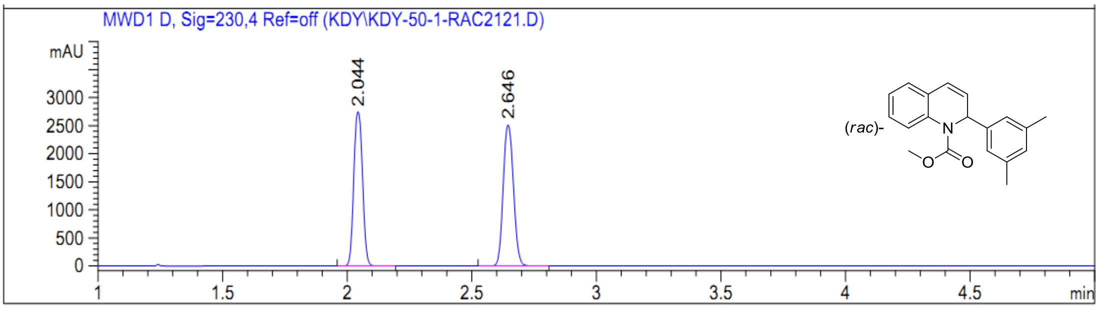

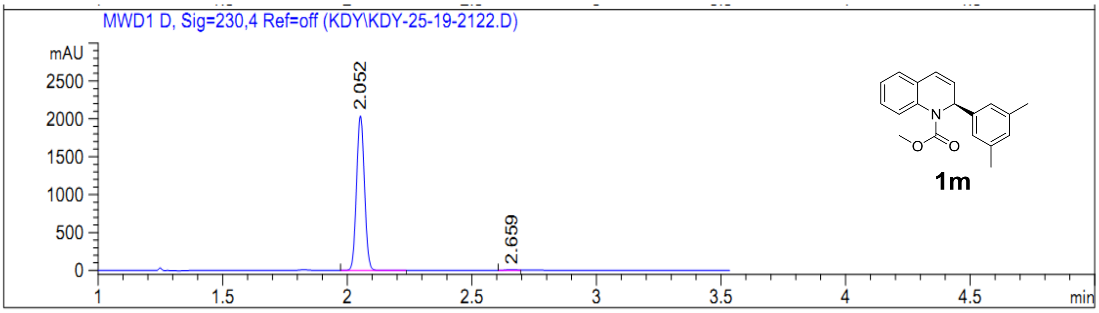

Peak #	RetTime [min]	Type	Width [min]	Area [mAU*s]	Height [mAU]	Area %
1	2.052	BV R	0.0347	4499.36133	2039.08789	99.3630
2	2.659	BV	0.0411	28.84402	10.80485	0.6370

Totals : 4528.20535 2049.89274

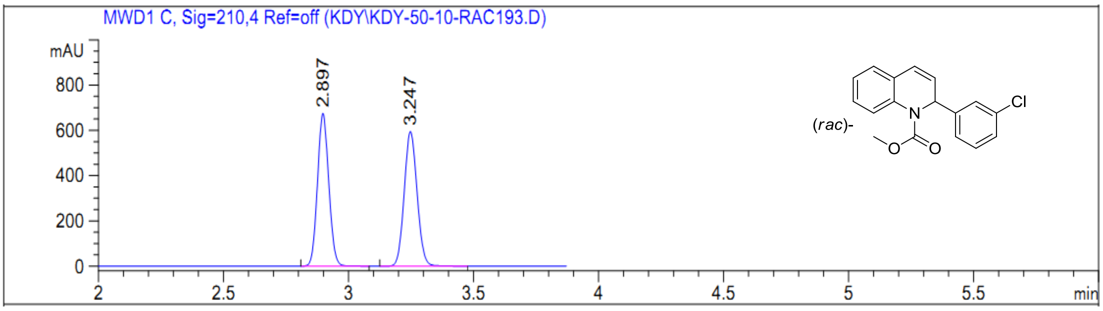

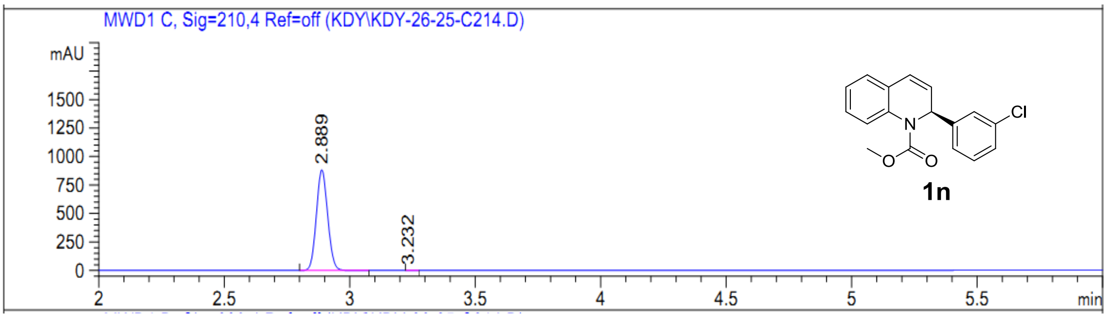

Peak #	RetTime [min]	Type	Width [min]	Area [mAU*s]	Height [mAU]	Area %
1	2.889	BV R	0.0486	2764.70215	882.46161	99.9575
2	3.232	VB	0.0290	1.17451	5.48428e-1	0.0425

Totals : 2765.87666 883.01004

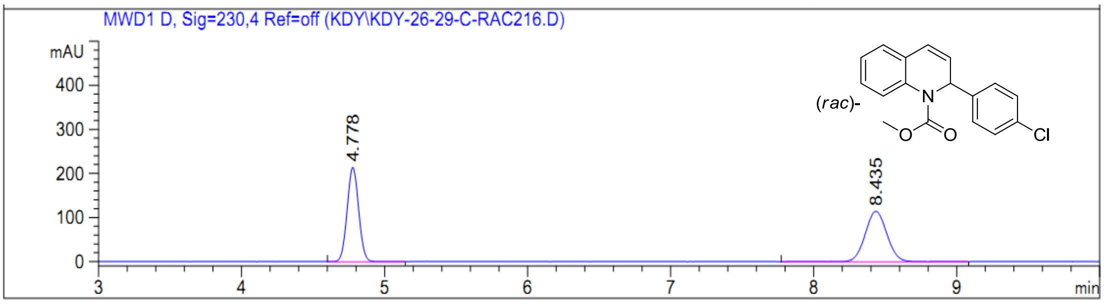

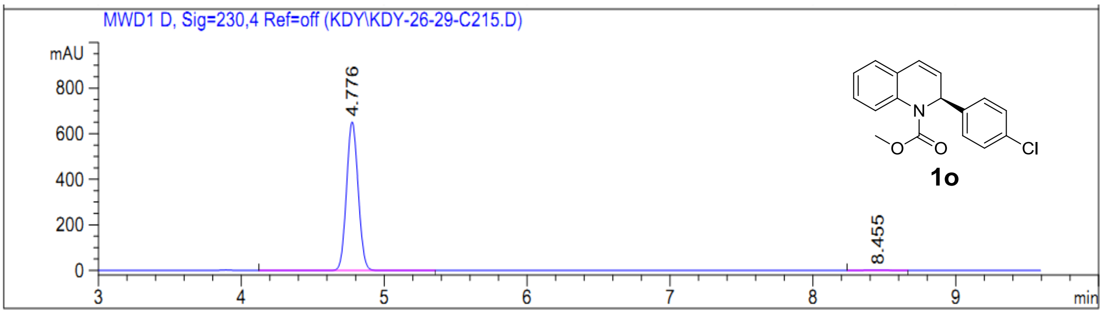

Peak #	RetTime [min]	Type	Width [min]	Area [mAU*s]	Height [mAU]	Area %
1	4.776	VV R	0.0879	3690.78418	651.10272	99.4280
2	8.455	VB	0.1668	21.23185	1.58006	0.5720

Totals : 3712.01603 652.68278

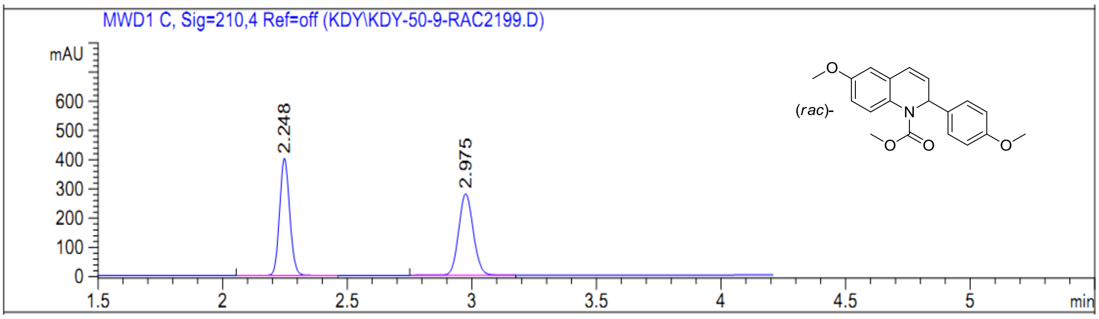

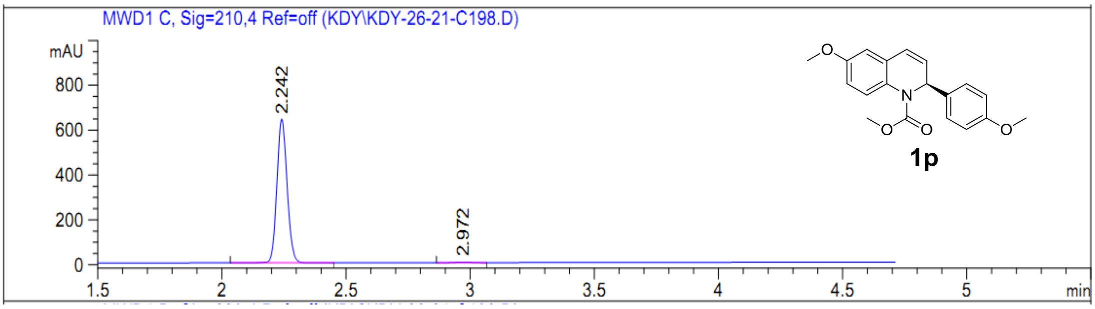

Peak #	RetTime [min]	Type	Width [min]	Area [mAU*s]	Height [mAU]	Area %
1	2.242	BB	0.0454	1826.28442	637.03552	99.4054
2	2.972	BB	0.0657	10.92443	2.67387	0.5946

Totals :				1837.20886	639.70939	

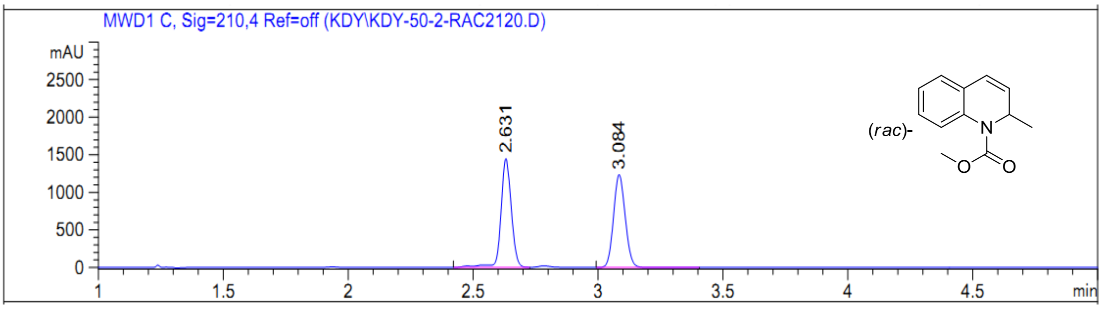

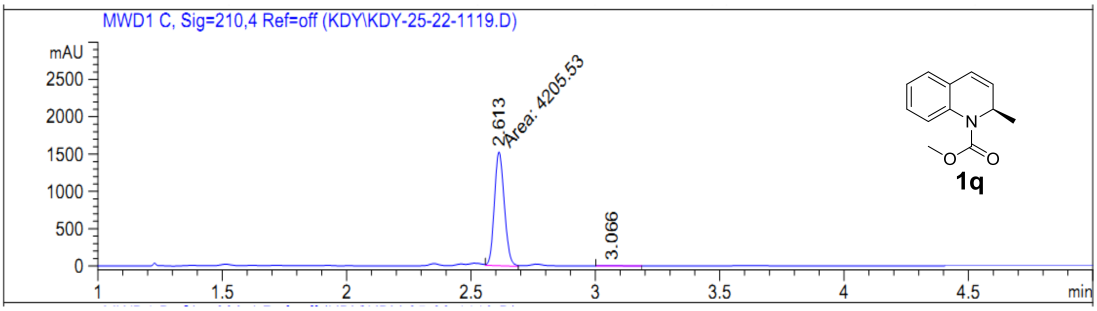

Peak #	RetTime [min]	Type	Width [min]	Area [mAU*s]	Height [mAU]	Area %
1	2.613	MM	0.0460	4205.53076	1524.93054	99.4676
2	3.066	BV R	0.0515	22.51037	6.65658	0.5324

Totals : 4228.04113 1531.58712

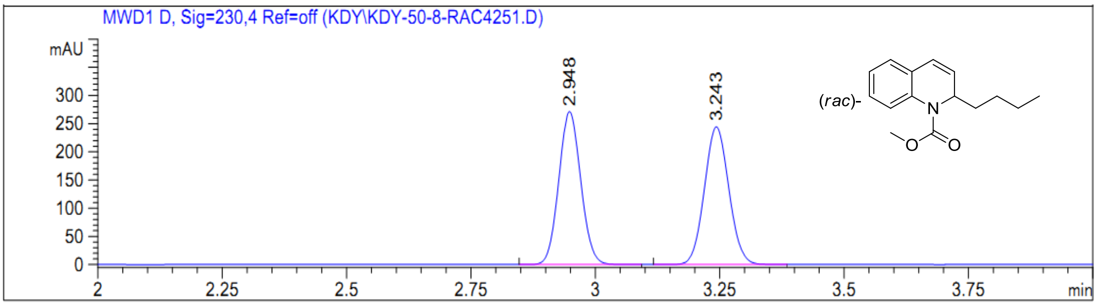

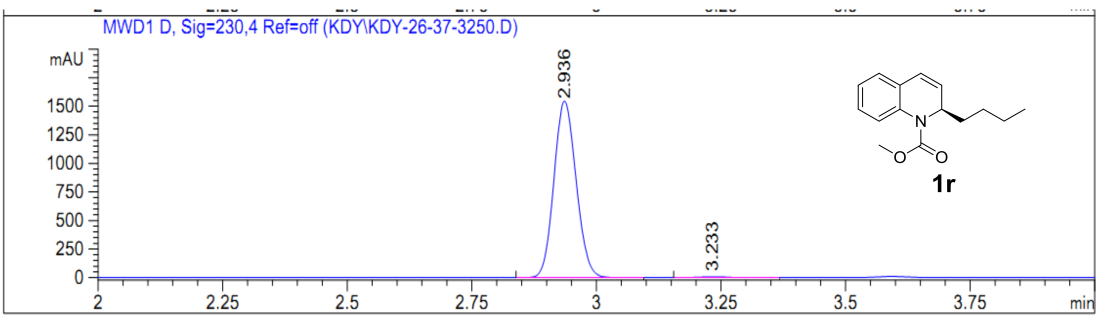

1r

Peak #	RetTime [min]	Type	Width [min]	Area [mAU*s]	Height [mAU]	Area %
1	2.936	BB	0.0487	4787.55713	1545.01685	99.3946
2	3.233	BV R	0.0530	29.16012	8.50900	0.6054

Totals : 4816.71725 1553.52585

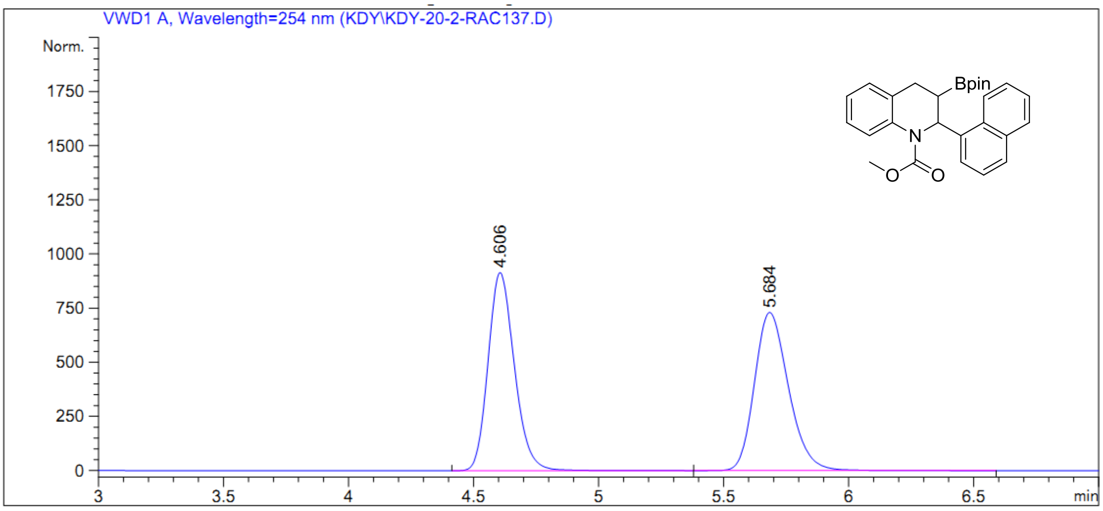

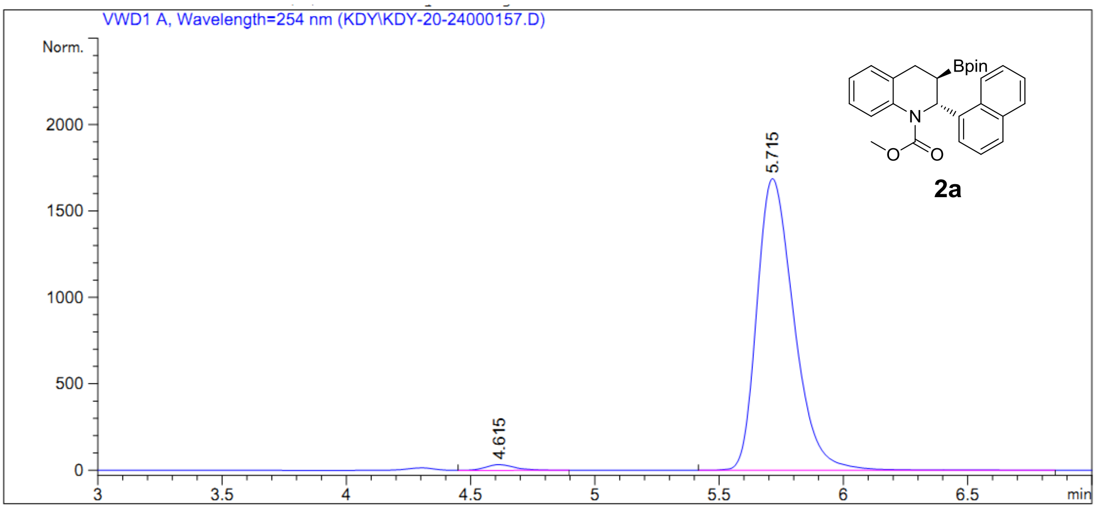

2a

Peak #	RetTime [min]	Type	Width [min]	Area [mAU*s]	Height [mAU]	Area %
1	4.615	VV	0.1263	283.21667	33.96539	1.5979
2	5.715	BB	0.1596	1.74411e4	1687.68201	98.4021

Totals : 1.77243e4 1721.64740

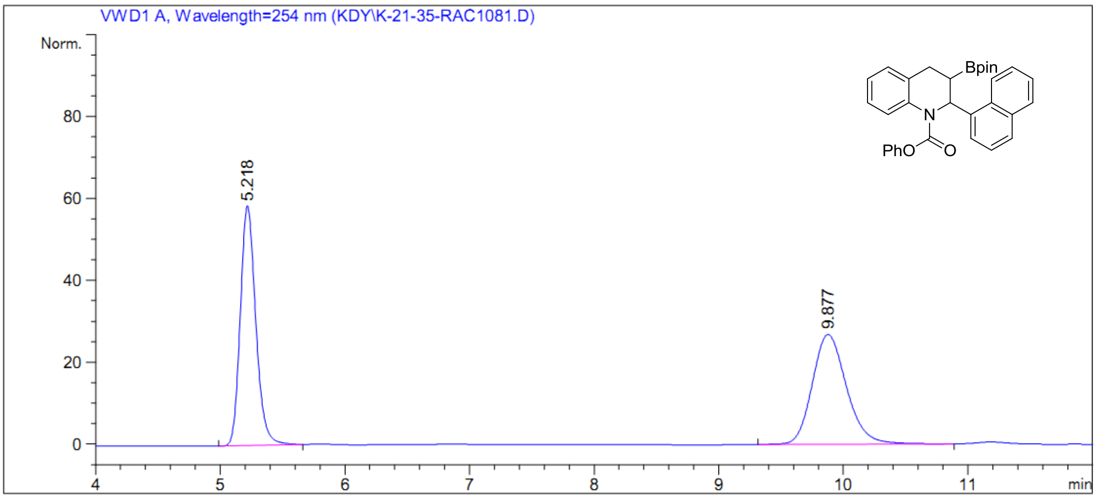

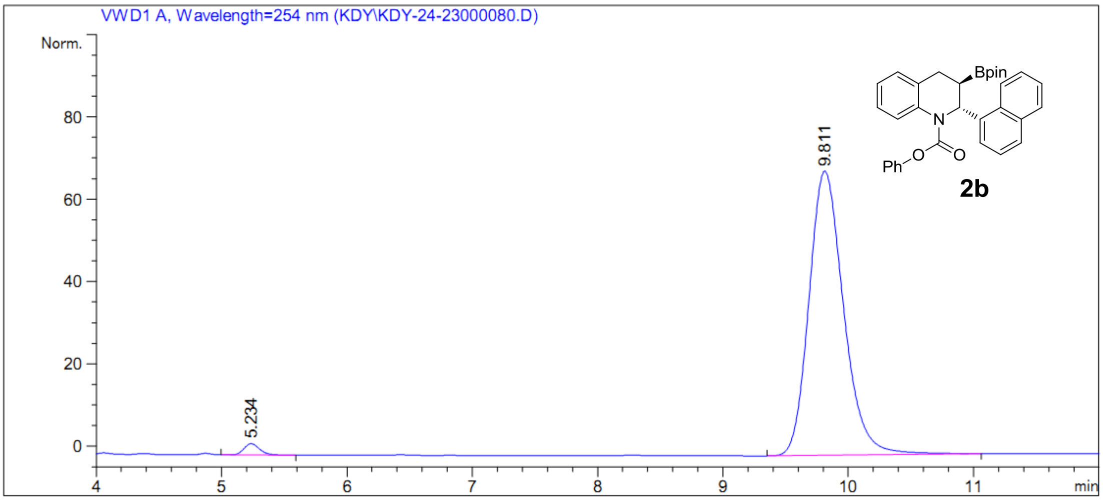

Peak #	RetTime [min]	Type	Width [min]	Area [mAU*s]	Height [mAU]	Area %
1	5.234	BB	0.1338	24.16607	2.77010	1.7898
2	9.811	BB	0.2961	1326.04944	69.03043	98.2102

Totals :	1350.21551	71.80053

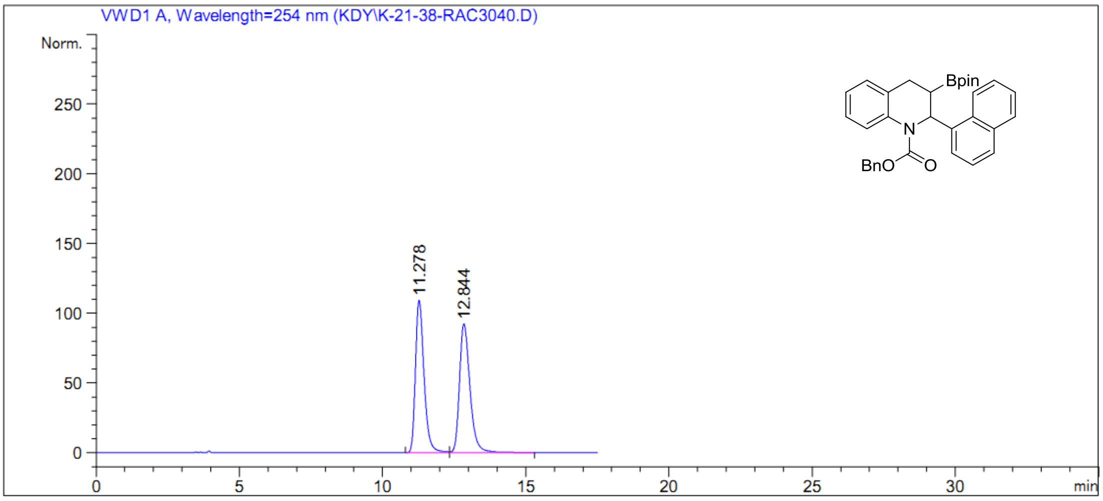

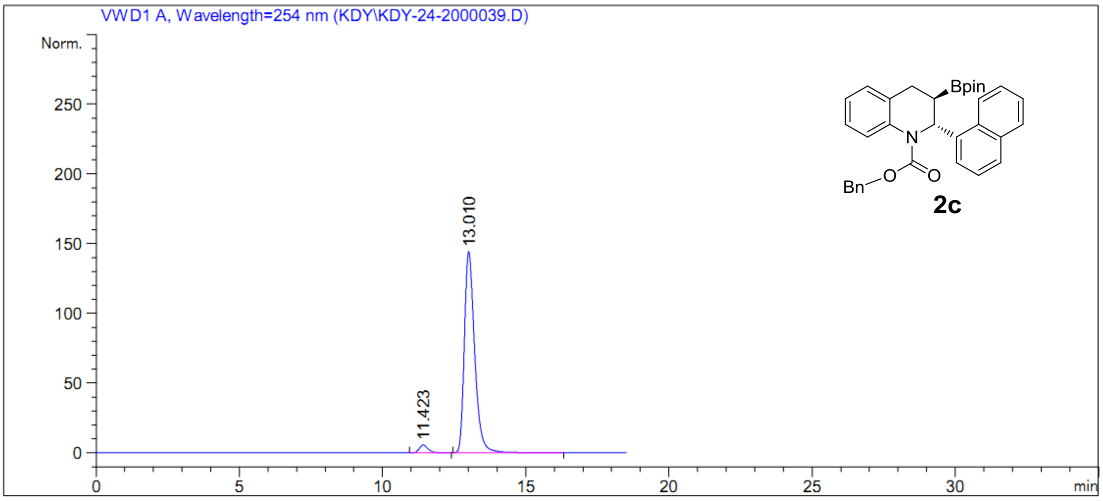

Peak #	RetTime [min]	Type	Width [min]	Area [mAU*s]	Height [mAU]	Area %
1	11.423	BB	0.3313	121.52647	5.59640	3.2625
2	13.010	BB	0.3818	3603.40820	144.08247	96.7375

Totals : 3724.93468 149.67887

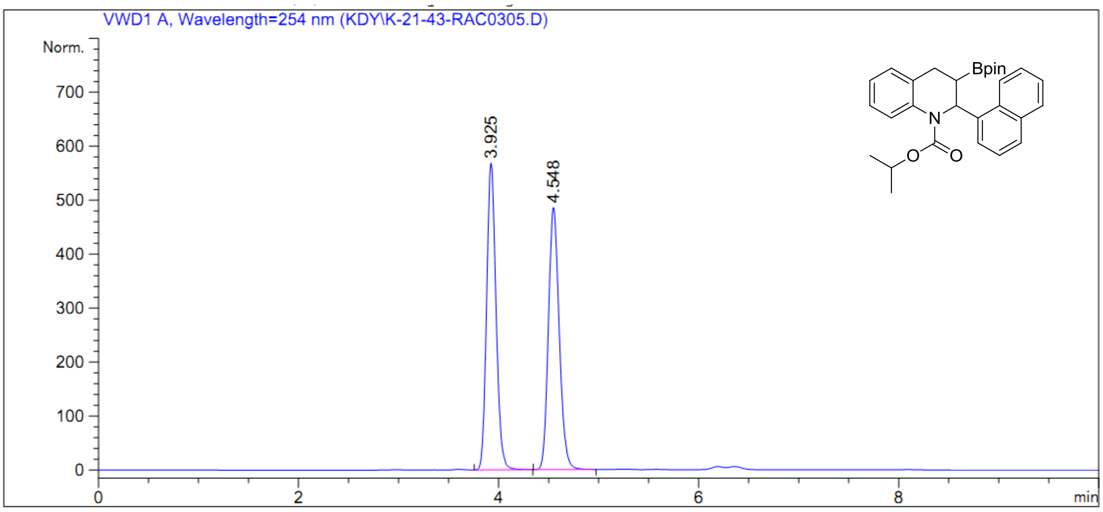

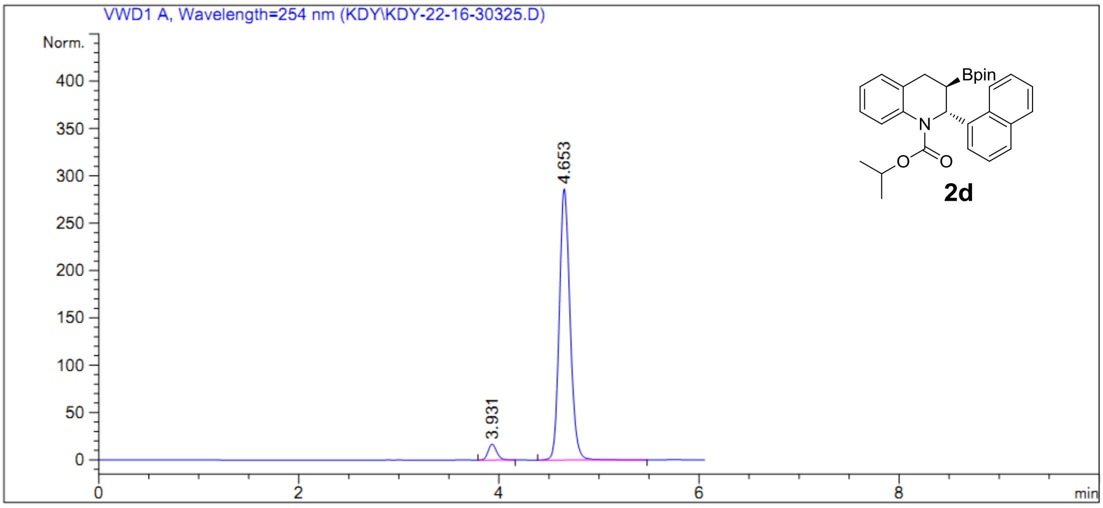

Peak #	RetTime [min]	Type	Width [min]	Area [mAU*s]	Height [mAU]	Area %
1	3.931	BB	0.0920	101.02320	16.83179	4.5177
2	4.653	BB	0.1139	2135.16553	286.55093	95.4823

Totals :				2236.18873	303.38272	

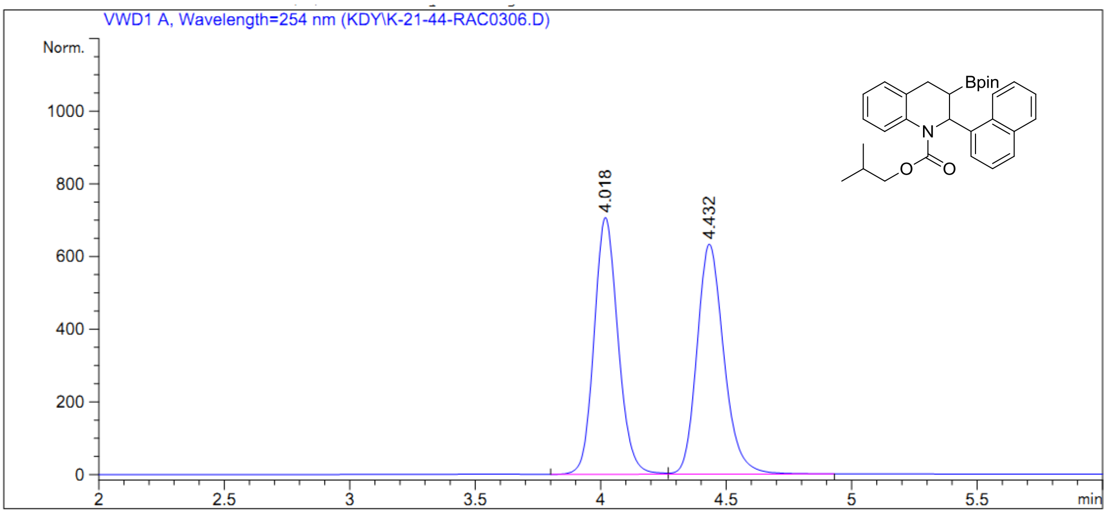

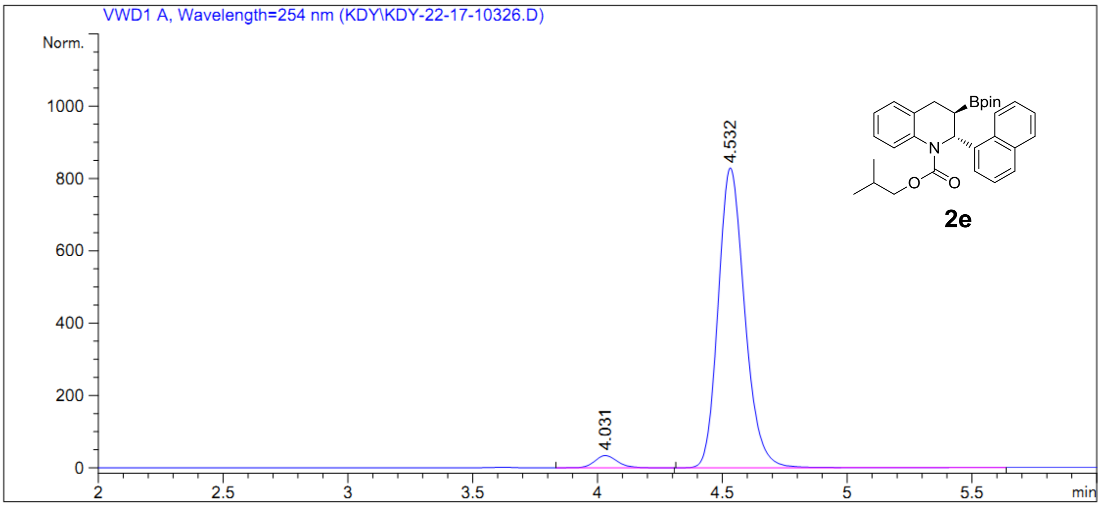

Peak #	RetTime [min]	Type	Width [min]	Area [mAU*s]	Height [mAU]	Area %
1	4.031	BB	0.0965	214.38449	34.00666	3.3817
2	4.532	BB	0.1142	6125.07959	829.24957	96.6183

Totals : 6339.46408 863.25624

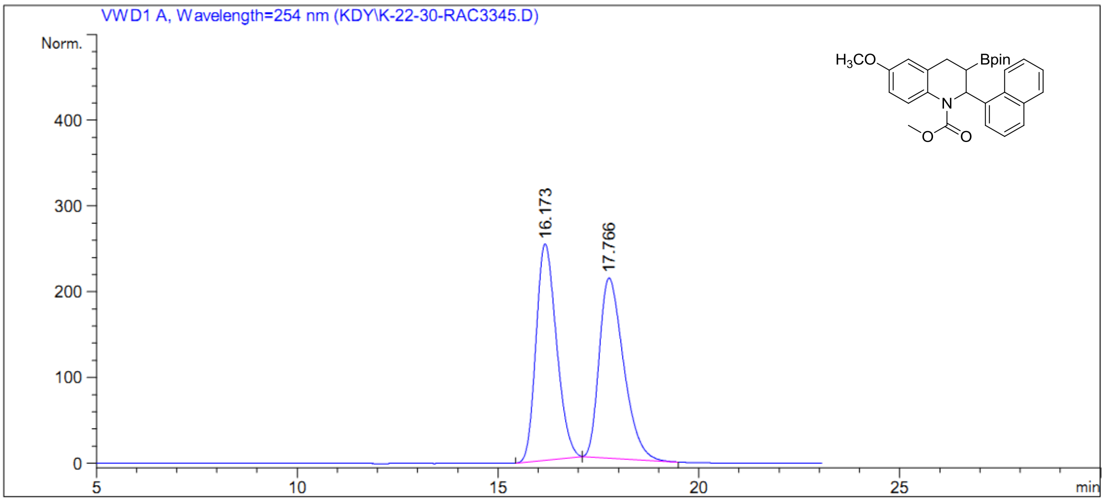

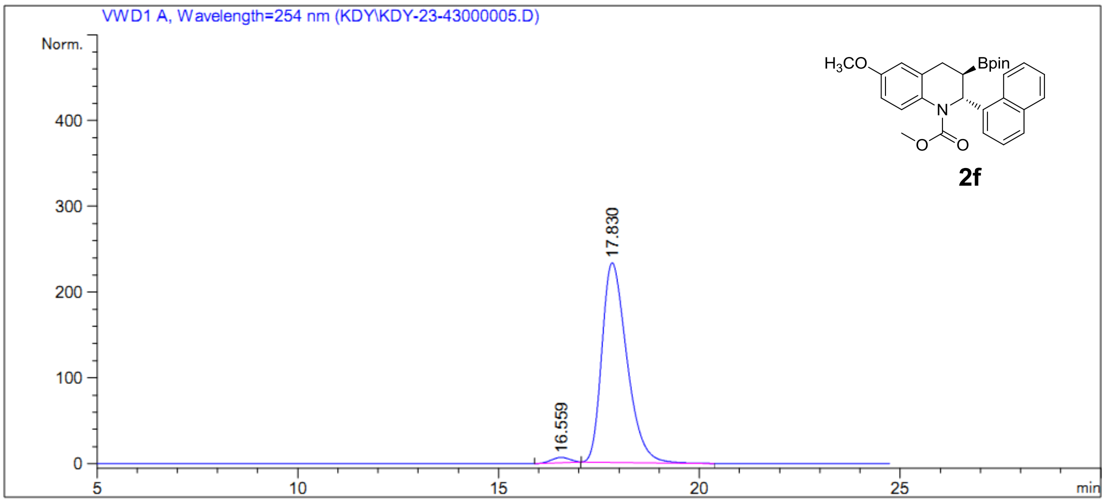

Peak #	RetTime [min]	Type	Width [min]	Area [mAU*s]	Height [mAU]	Area %
1	16.559	BB	0.5138	197.61757	6.26783	1.9247
2	17.830	BB	0.6649	1.00699e4	232.67824	98.0753

Totals : 1.02675e4 238.94607

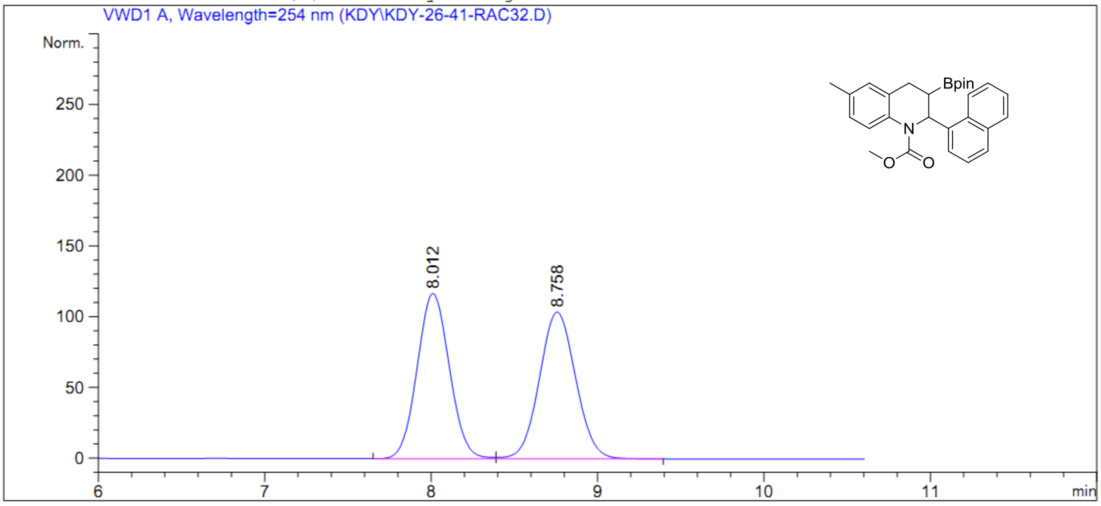

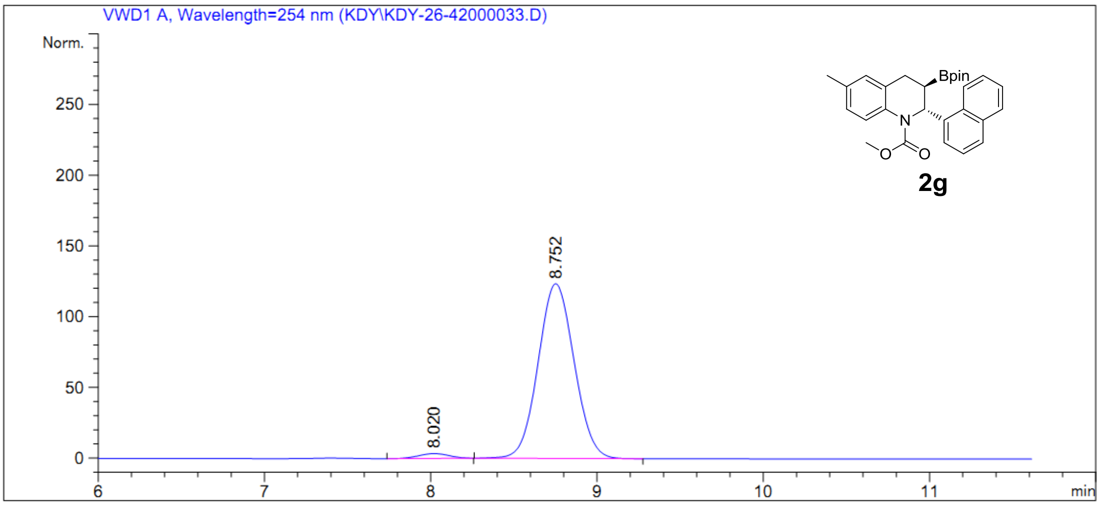

Peak #	RetTime [min]	Type	Width [min]	Area [mAU*s]	Height [mAU]	Area %
1	8.020	BB	0.2001	43.77079	3.44834	2.2967
2	8.752	BB	0.2358	1862.00842	123.58708	97.7033

Totals : 1905.77921 127.03543

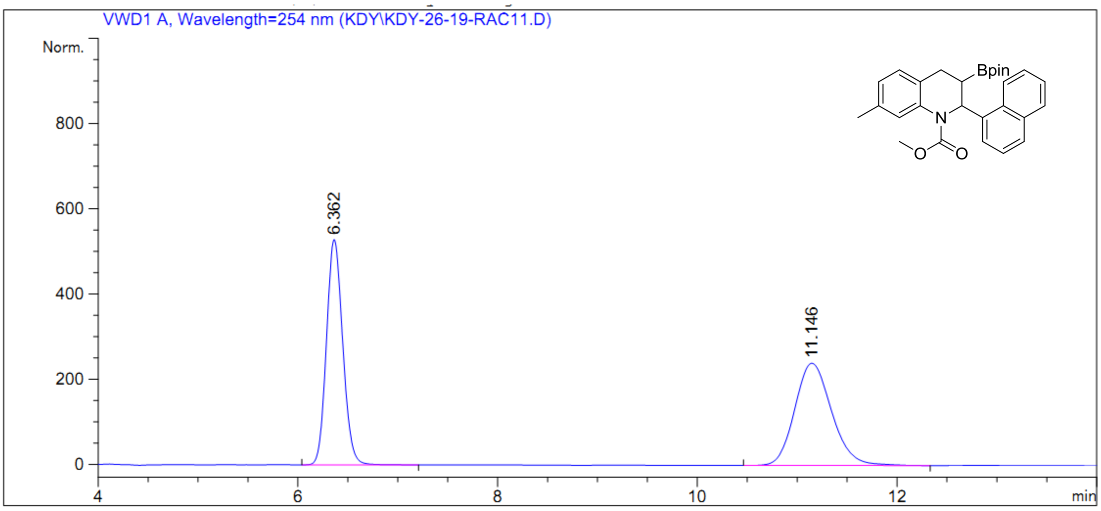

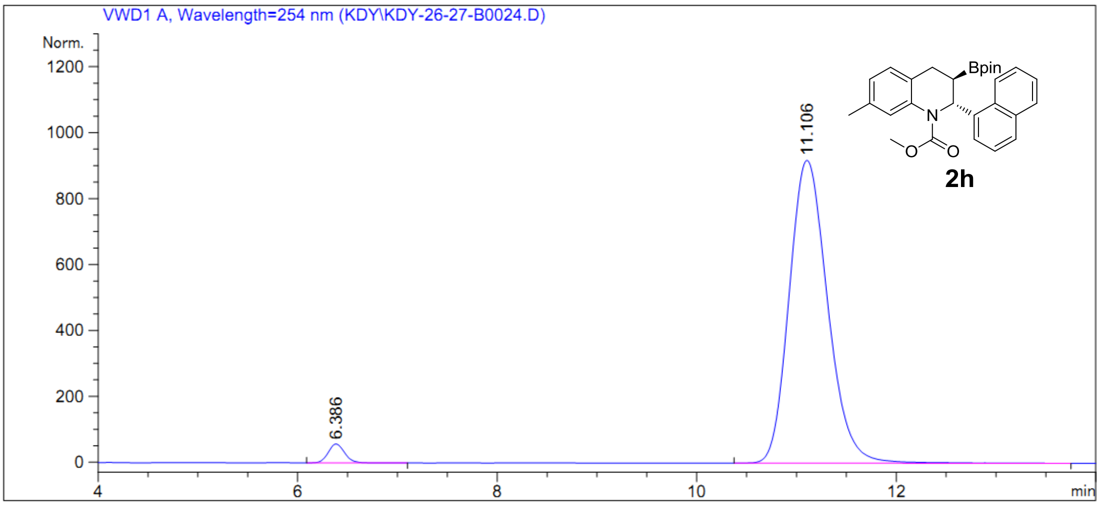

Peak #	RetTime [min]	Type	Width [min]	Area [mAU*s]	Height [mAU]	Area %
1	6.386	VB	0.1782	663.16229	57.63523	2.6284
2	11.106	BB	0.4179	2.45672e4	918.90814	97.3716

Totals : 2.52303e4 976.54337

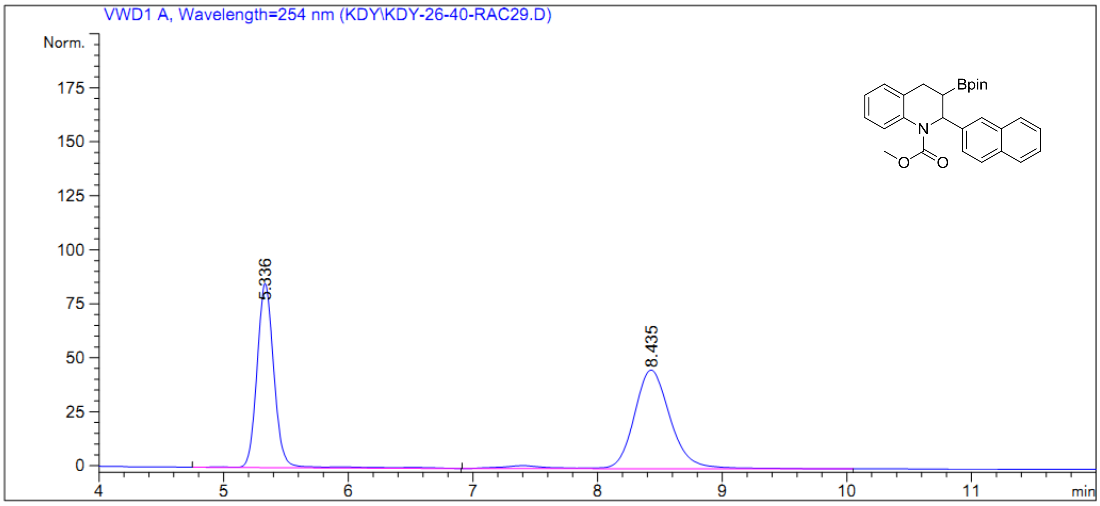

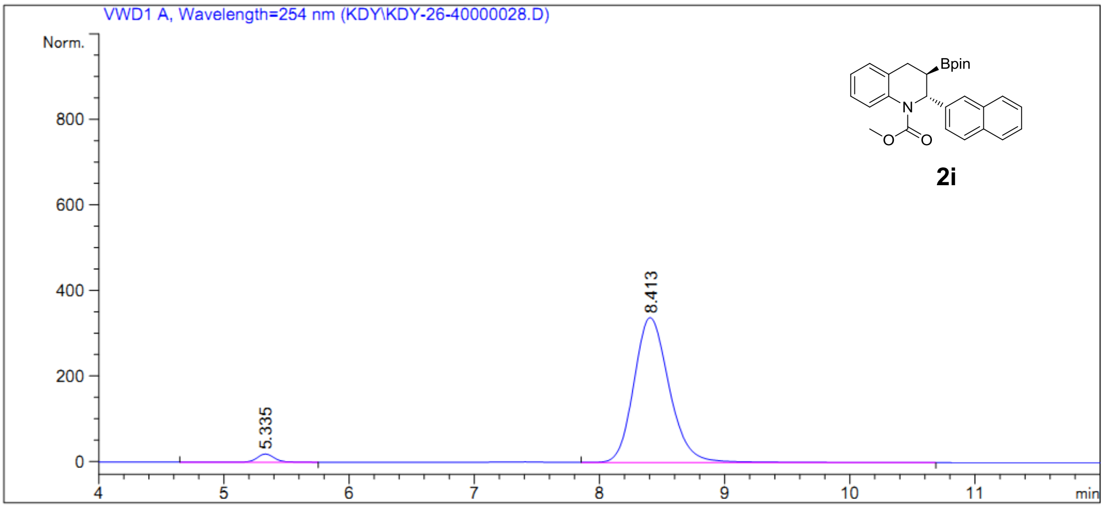

Peak #	RetTime [min]	Type	Width [min]	Area [mAU*s]	Height [mAU]	Area %
1	5.335	BB	0.1955	194.60635	17.27102	2.8585
2	8.413	BB	0.3252	6613.37744	336.63840	97.1415

Totals : 6807.98380 353.90942

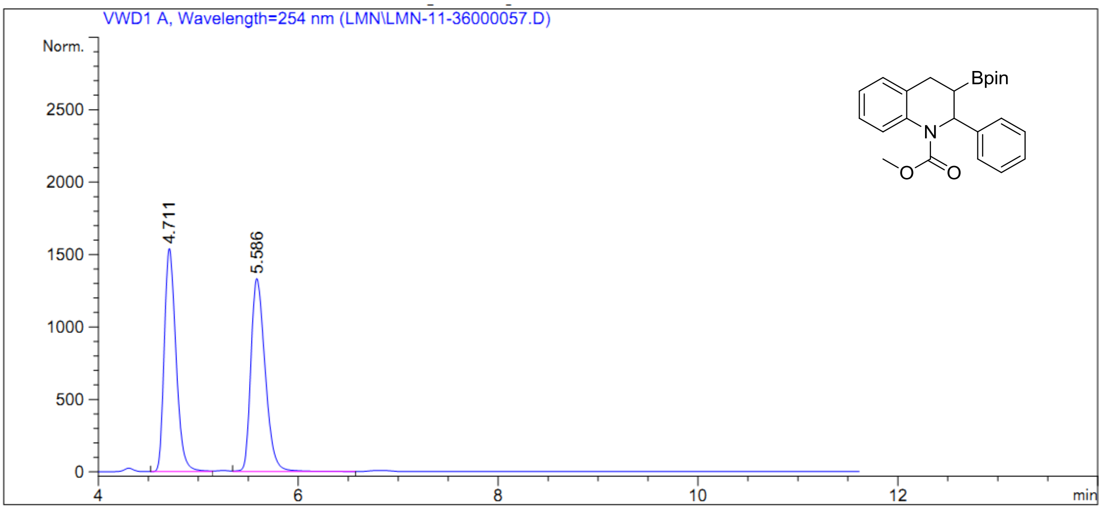

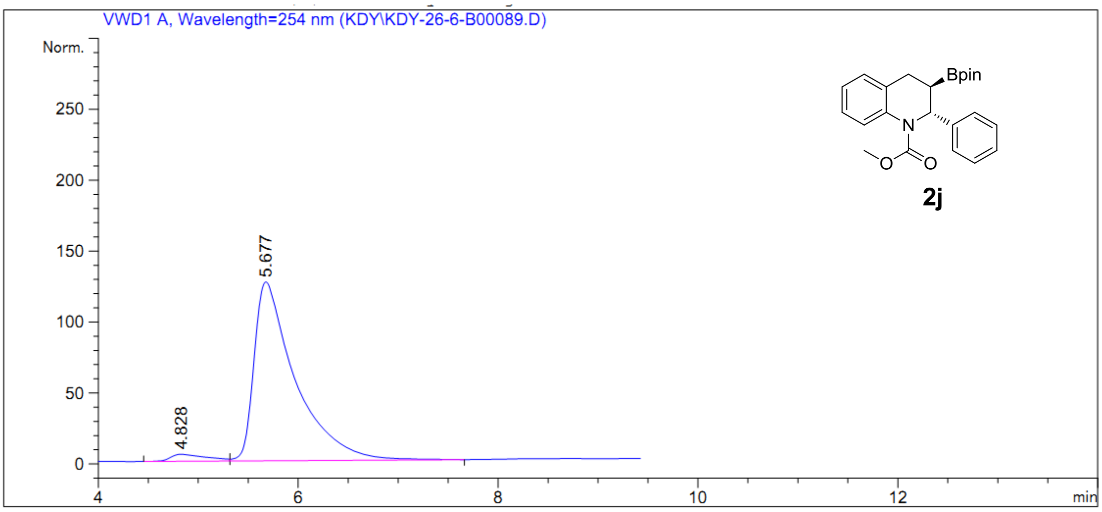

Peak #	RetTime [min]	Type	Width [min]	Area [mAU*s]	Height [mAU]	Area %
1	4.828	BV	0.3429	121.82275	4.95448	3.2658
2	5.677	VB	0.4048	3608.46924	126.10669	96.7342

Totals : 3730.29199 131.06117

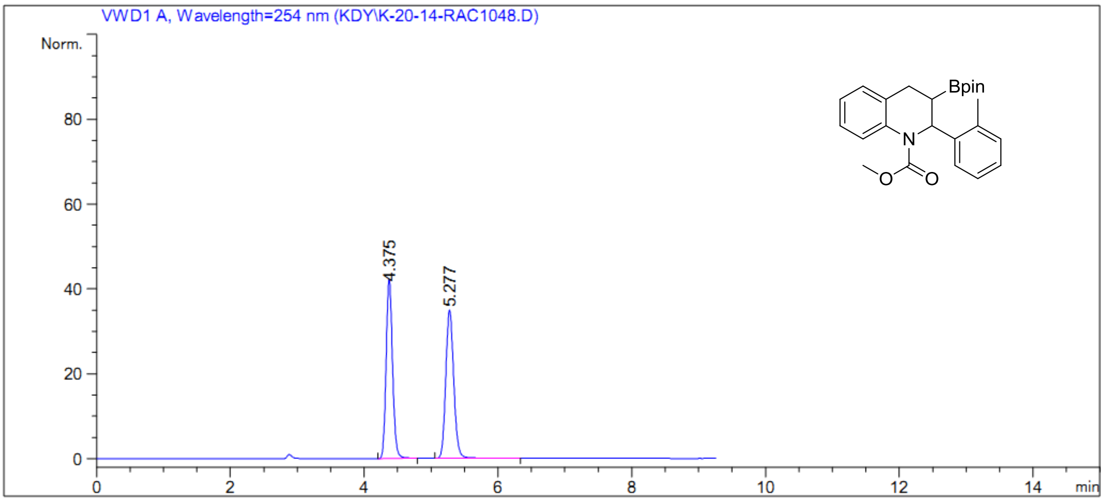

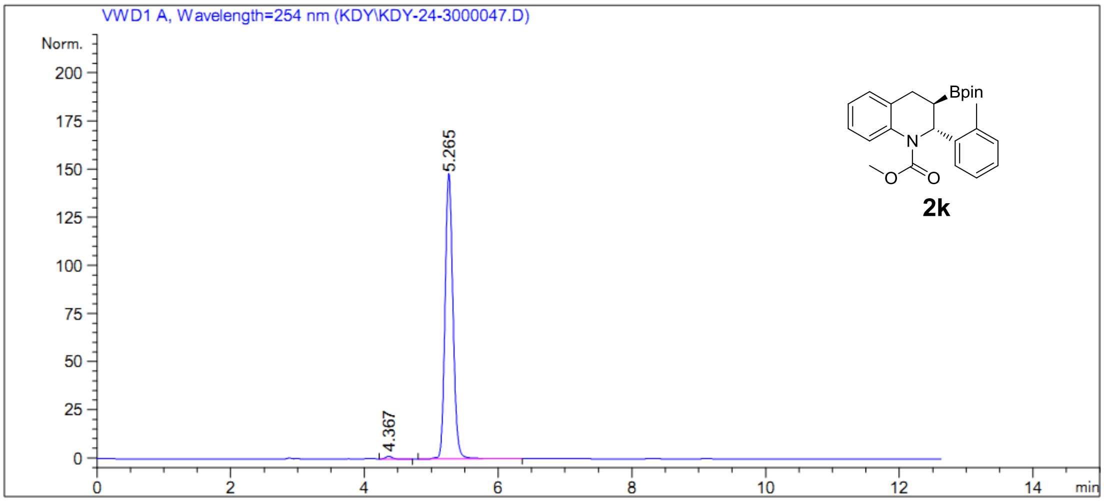

Peak #	RetTime [min]	Type	Width [min]	Area [mAU*s]	Height [mAU]	Area %
1	4.367	BB	0.1093	8.66824	1.30749	0.7085
2	5.265	BB	0.1366	1214.72839	146.67262	99.2915

Totals : 1223.39663 147.98011

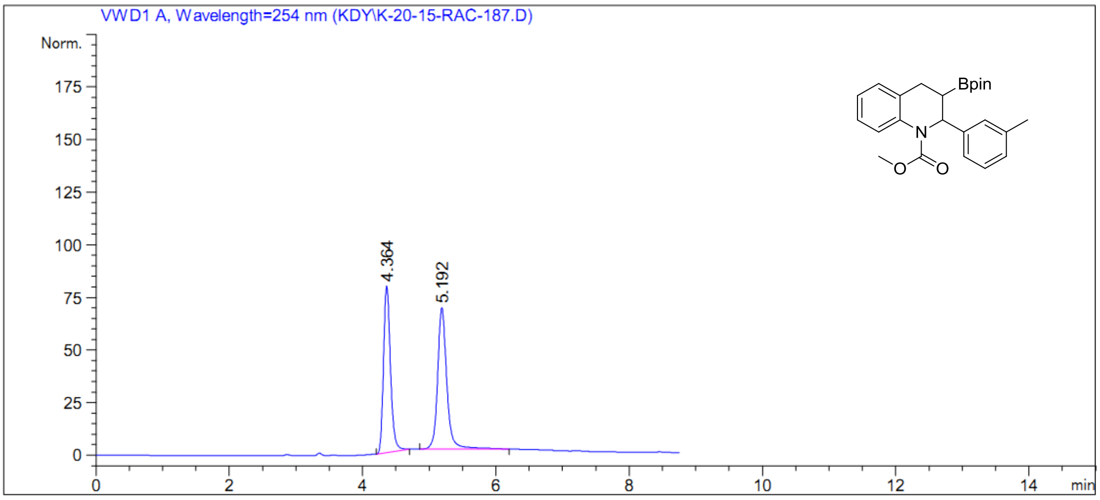

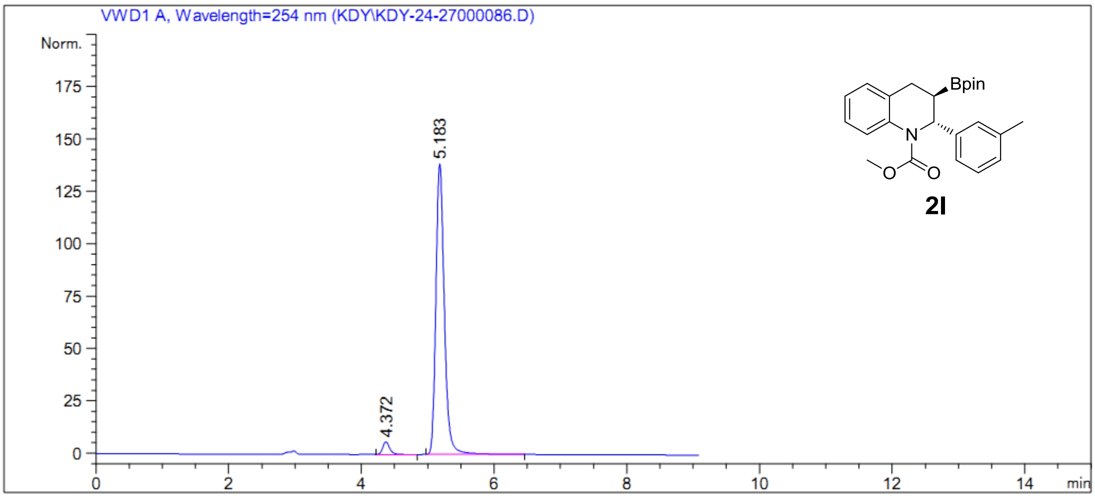

Peak #	RetTime [min]	Type	Width [min]	Area [mAU*s]	Height [mAU]	Area %
1	4.372	BB	0.1131	44.09037	5.97571	3.4811
2	5.183	BB	0.1348	1222.48132	138.79312	96.5189
Totals :				1266.57169	144.76883	

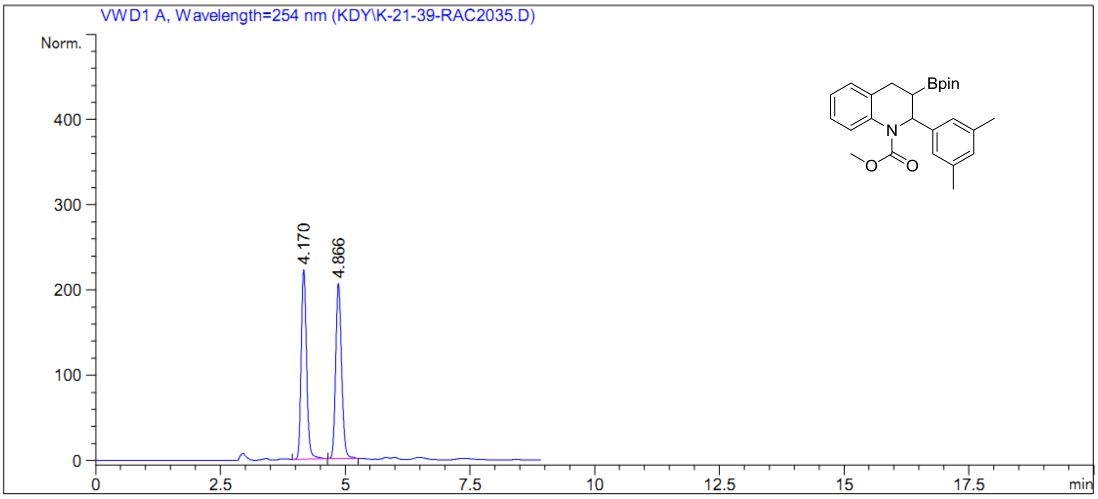

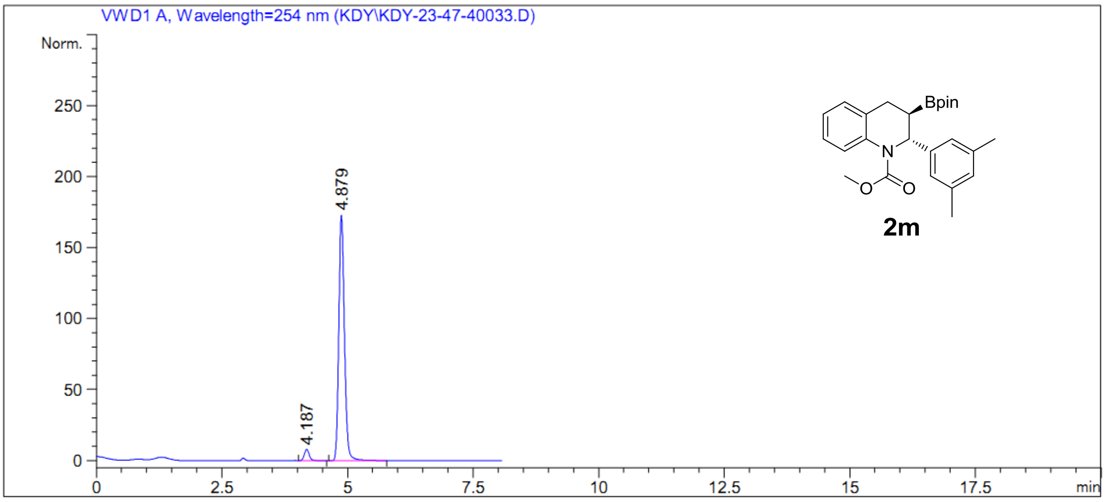

Peak #	RetTime [min]	Type	Width [min]	Area [mAU*s]	Height [mAU]	Area %
1	4.187	BB	0.1056	54.88206	7.94840	3.9020
2	4.879	BB	0.1212	1351.63513	172.87549	96.0980

Totals : 1406.51719 180.82389

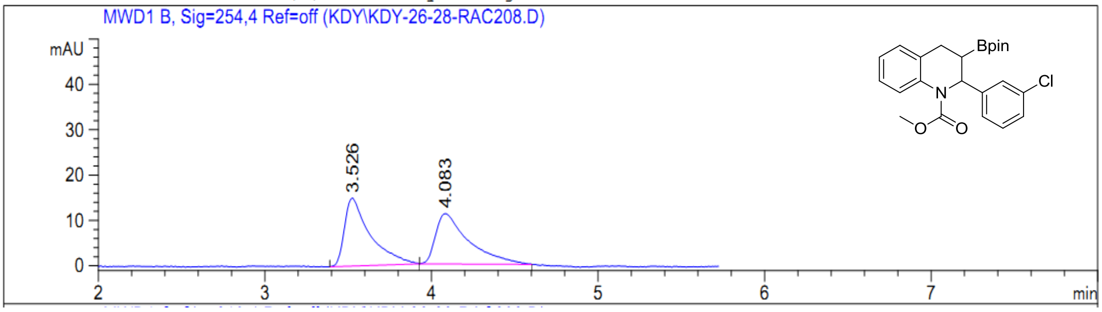

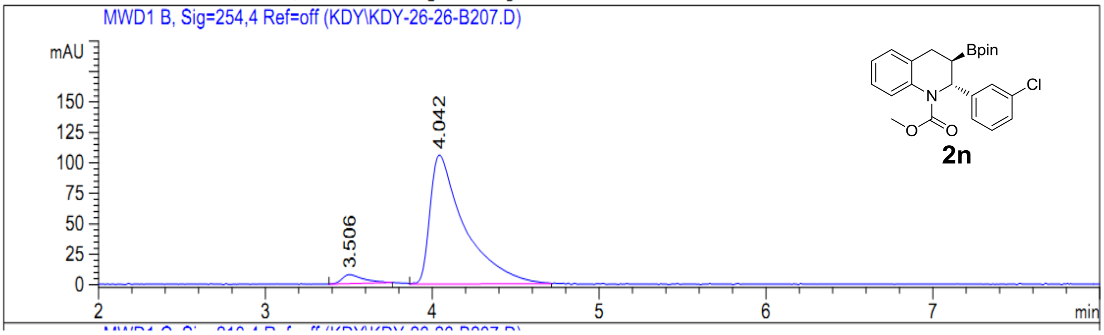

Peak #	RetTime [min]	Type	Width [min]	Area [mAU*s]	Height [mAU]	Area %
1	3.506	BV R	0.1199	66.02846	7.34090	4.1549
2	4.042	VB R	0.1977	1523.12610	105.66371	95.8451

Totals : 1589.15456 113.00462

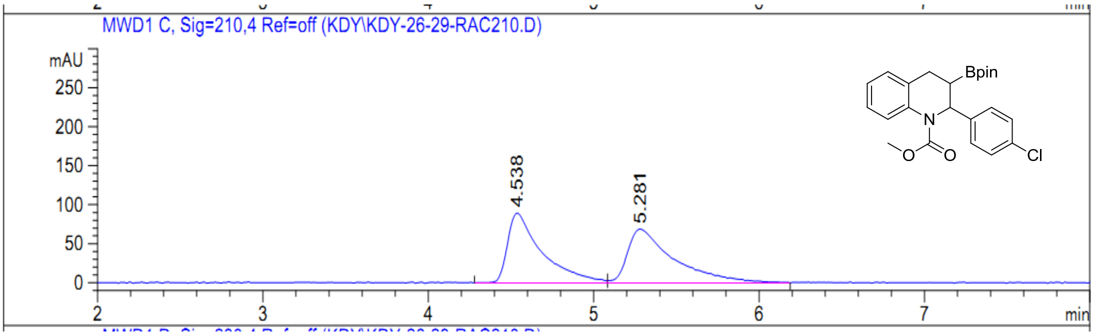

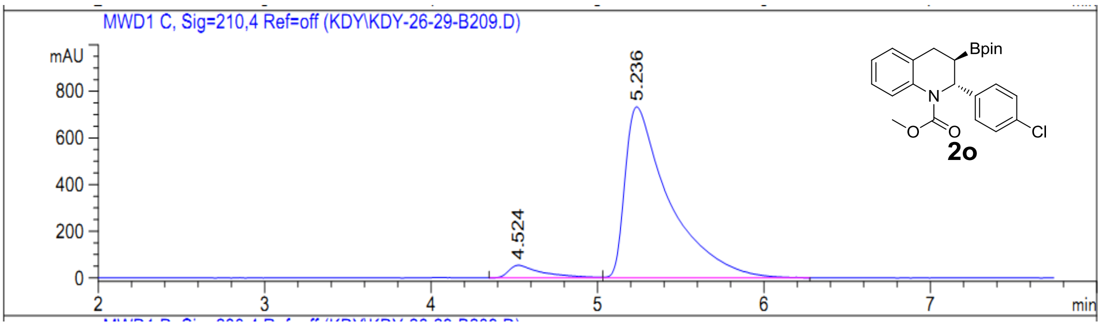

Peak #	RetTime [min]	Type	Width [min]	Area [mAU*s]	Height [mAU]	Area %
1	4.524	BV R	0.1859	755.50800	54.04028	5.2820
2	5.236	VV R	0.2585	1.35479e4	733.19049	94.7180
Totals :				1.43034e4	787.23077	

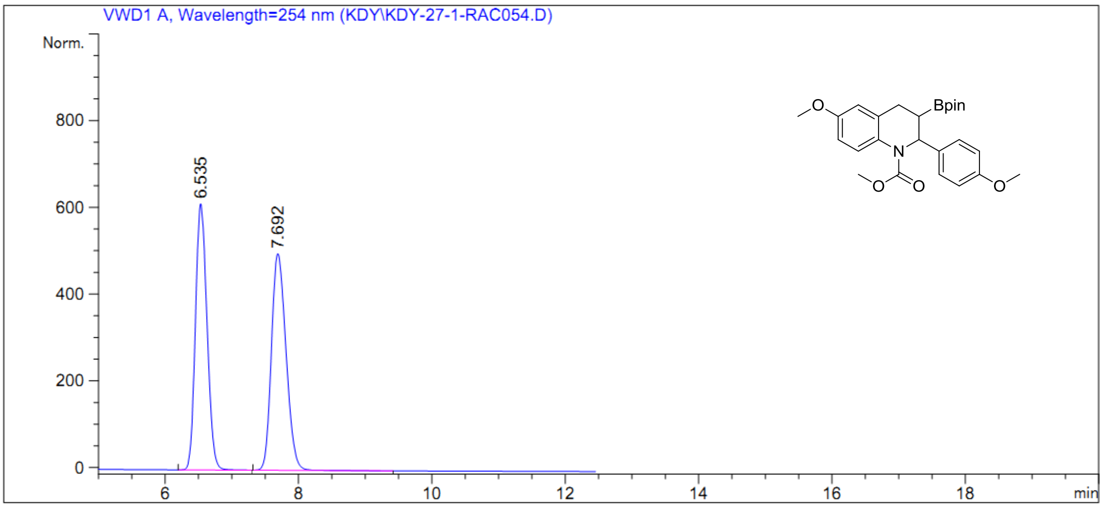

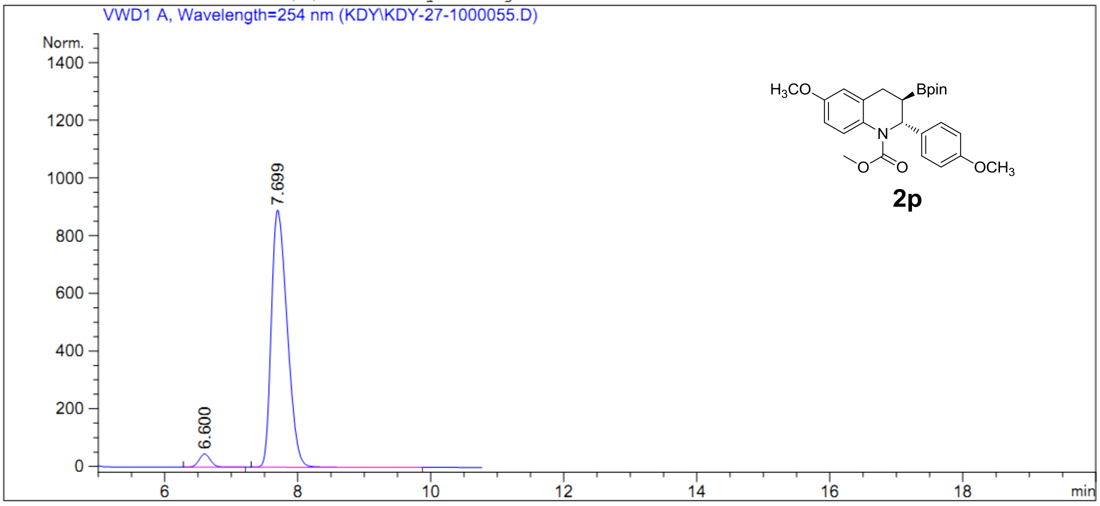

Peak #	RetTime [min]	Type	Width [min]	Area [mAU*s]	Height [mAU]	Area %
1	6.600	BB	0.1841	544.87994	46.02865	3.5704
2	7.699	BBA	0.2606	1.47160e4	891.40393	96.4296

Totals : 1.52609e4 937.43258

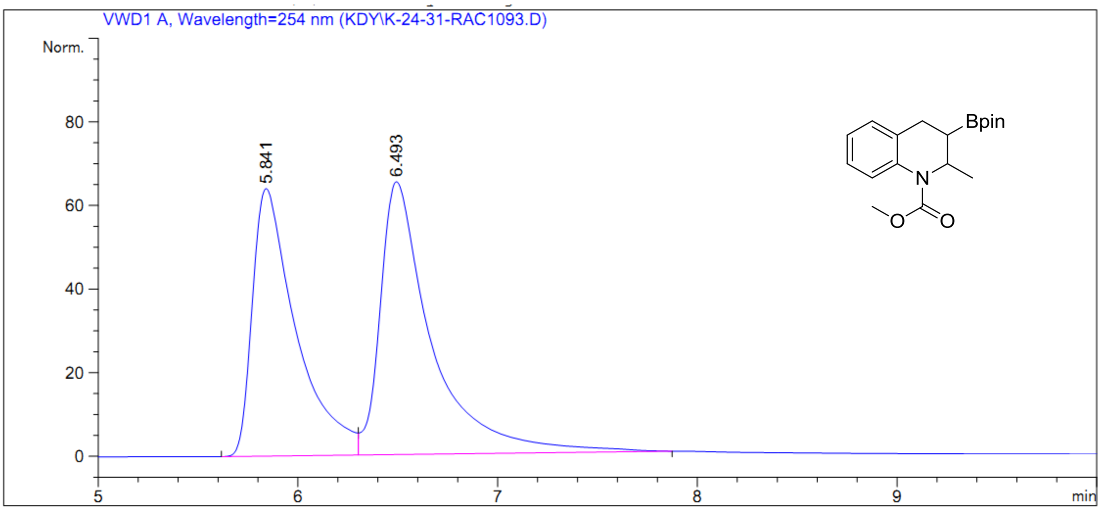

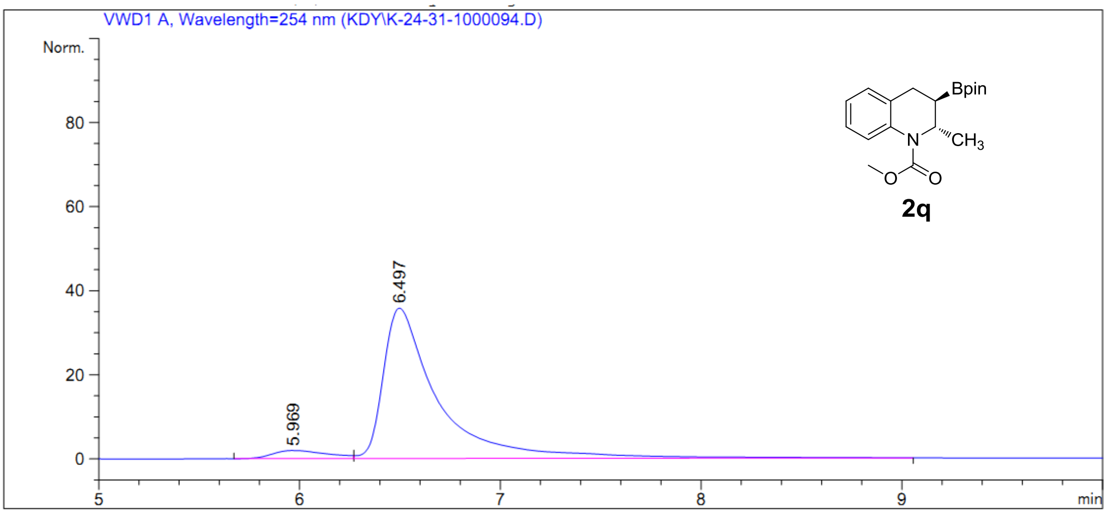

Peak #	RetTime [min]	Type	Width [min]	Area [mAU*s]	Height [mAU]	Area %
1	5.969	BV	0.2803	36.59196	1.96500	5.0831
2	6.497	VB	0.2709	683.28021	35.75909	94.9169
Totals :				719.87218	37.72409	

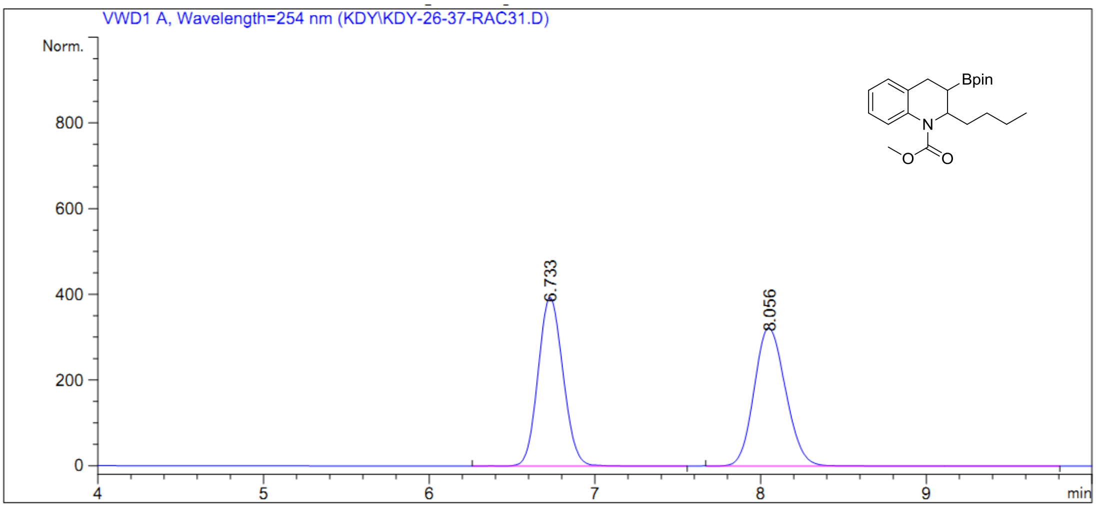

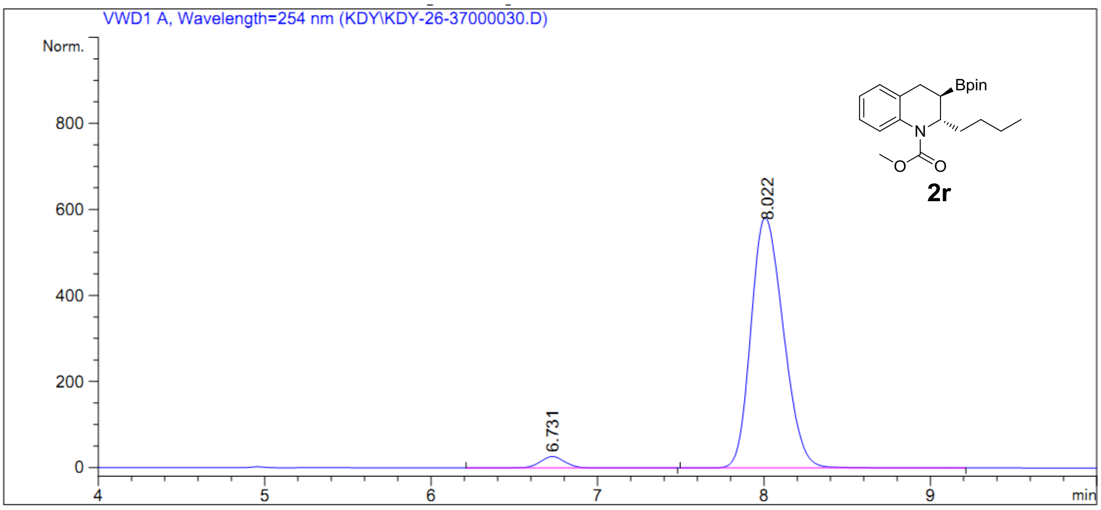

Peak #	RetTime [min]	Type	Width [min]	Area [mAU*s]	Height [mAU]	Area %
1	6.731	BB	0.2060	272.34567	25.20897	3.3420
2	8.022	BB	0.2427	7876.91016	564.92621	96.6580

Totals : 8149.25583 590.13518

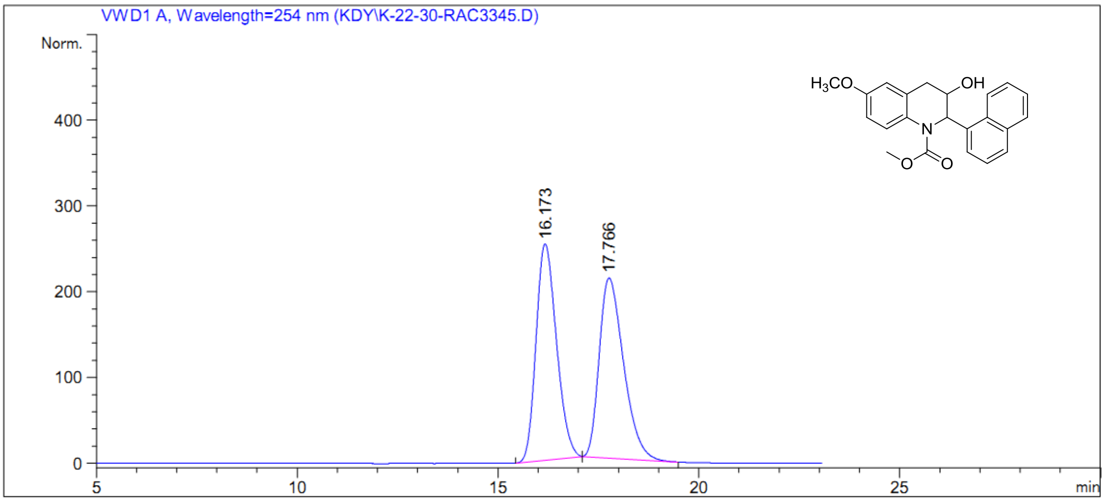

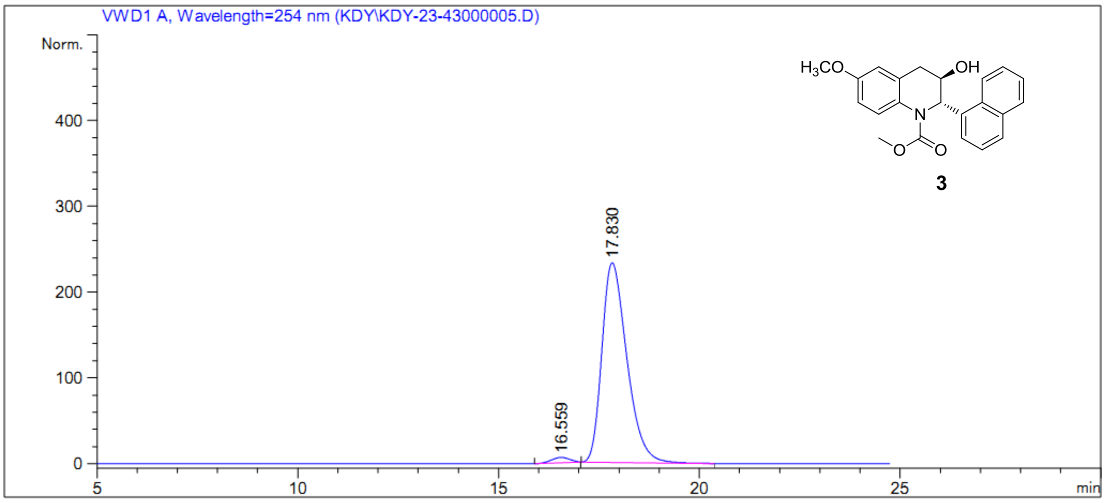

Peak #	RetTime [min]	Type	Width [min]	Area [mAU*s]	Height [mAU]	Area %
1	16.559	BB	0.5138	197.61757	6.26783	1.9247
2	17.830	BB	0.6649	1.00699e4	232.67824	98.0753

Totals : 1.02675e4 238.94607

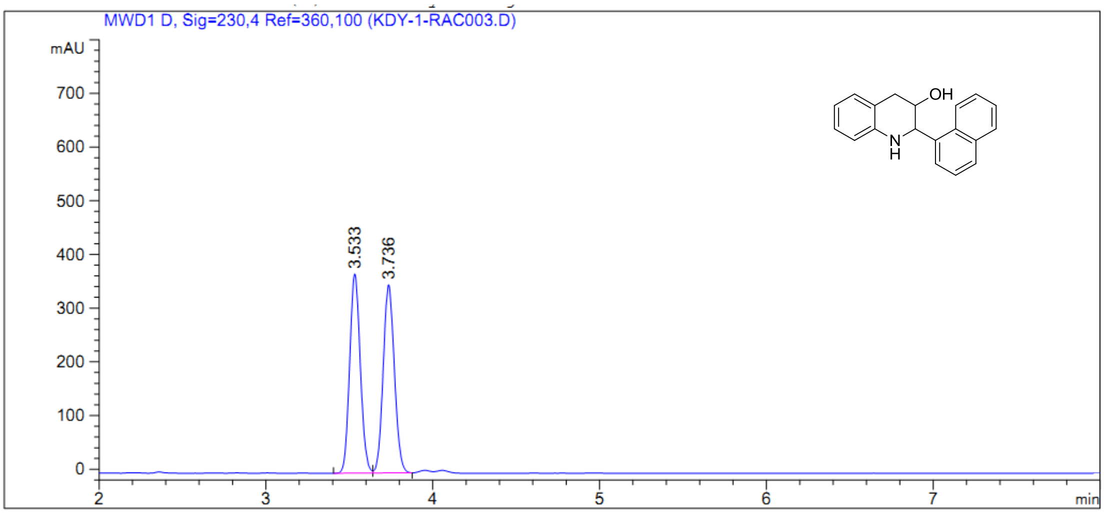

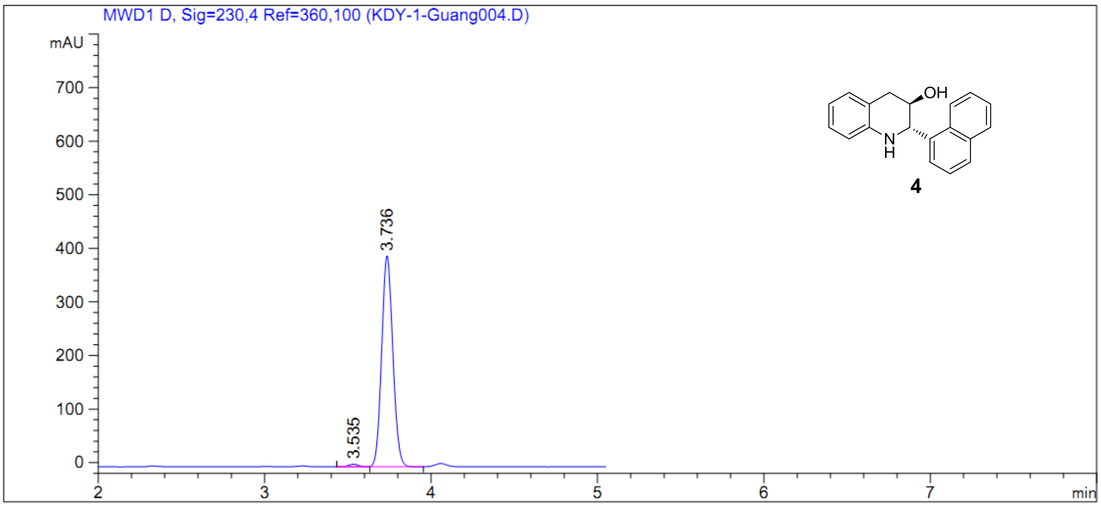

Peak #	RetTime [min]	Type	Width [min]	Area [mAU*s]	Height [mAU]	Area %
1	3.535	BV E	0.0684	20.97322	4.71424	1.1233
2	3.736	VV R	0.0727	1846.19556	393.86246	98.8767

Totals : 1867.16878 398.57670

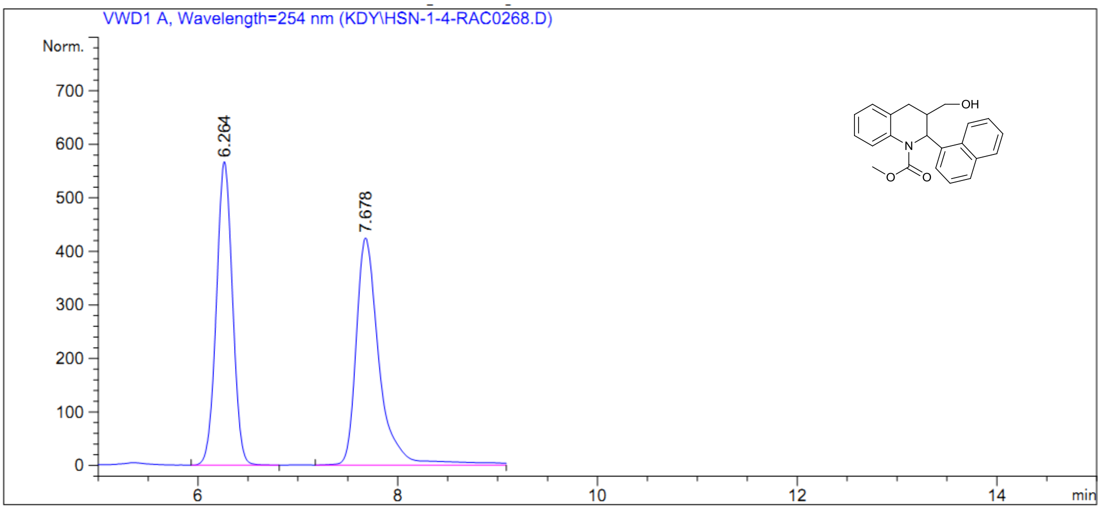

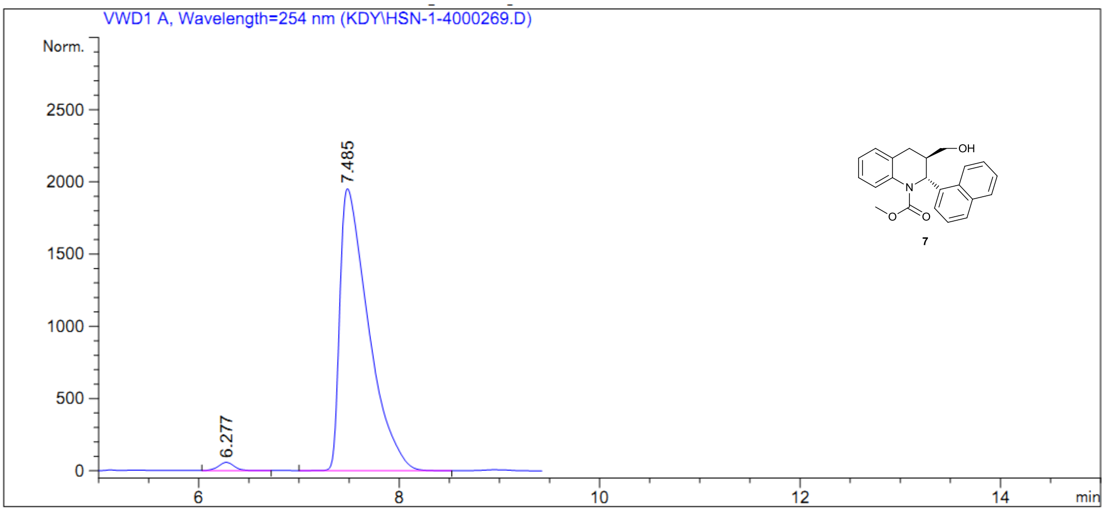

Peak #	RetTime [min]	Type	Width [min]	Area [mAU*s]	Height [mAU]	Area %
1	6.277	VV	0.1680	628.06512	57.23338	1.6013
2	7.485	BB	0.2996	3.85943e4	1952.39661	98.3987

Totals : 3.92224e4 2009.62998

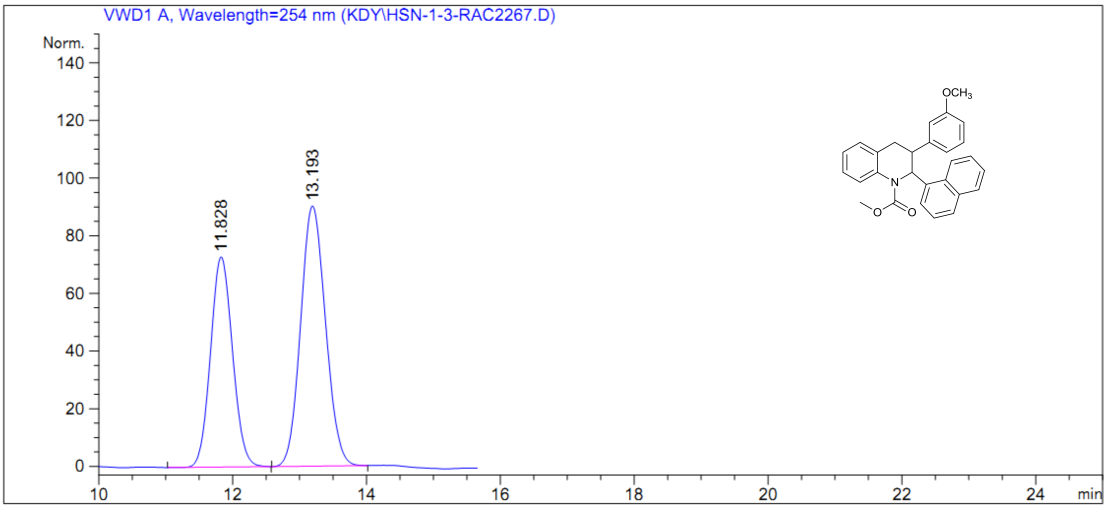

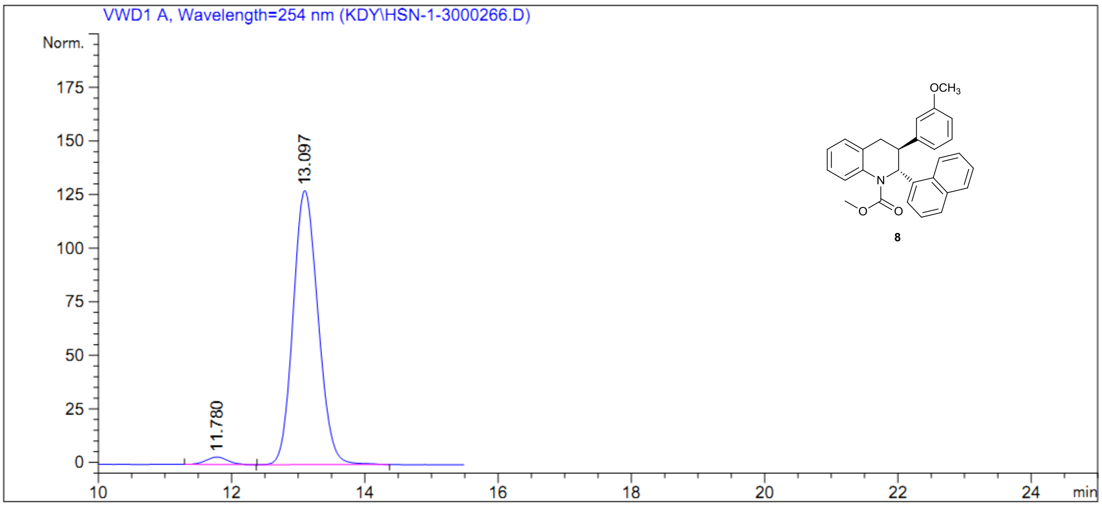

Peak #	RetTime [min]	Type	Width [min]	Area [mAU*s]	Height [mAU]	Area %
1	11.780	BB	0.3499	78.37698	3.46513	2.3083
2	13.097	BB	0.4047	3317.01221	127.84676	97.6917

Totals : 3395.38919 131.31189

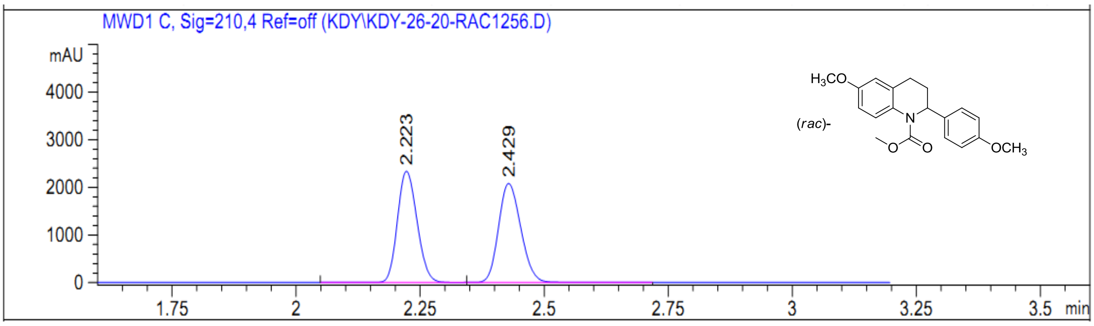

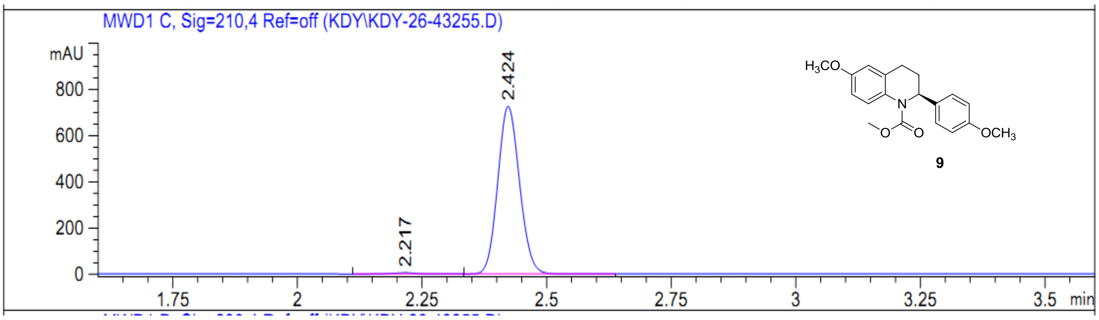

Peak #	RetTime [min]	Type	Width [min]	Area [mAU*s]	Height [mAU]	Area %
1	2.217	VB R	0.0439	15.49684	5.33096	0.6981
2	2.424	BV R	0.0474	2204.23462	725.51215	99.3019

Totals : 2219.73146 730.84311

8. Figures of single-crystals

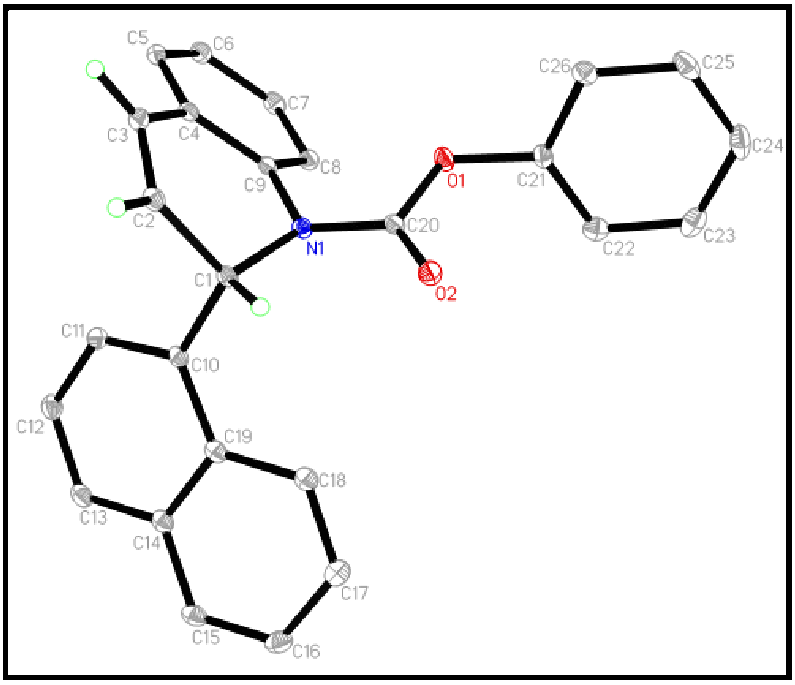

Figure S1. Structure of compound **1b**.

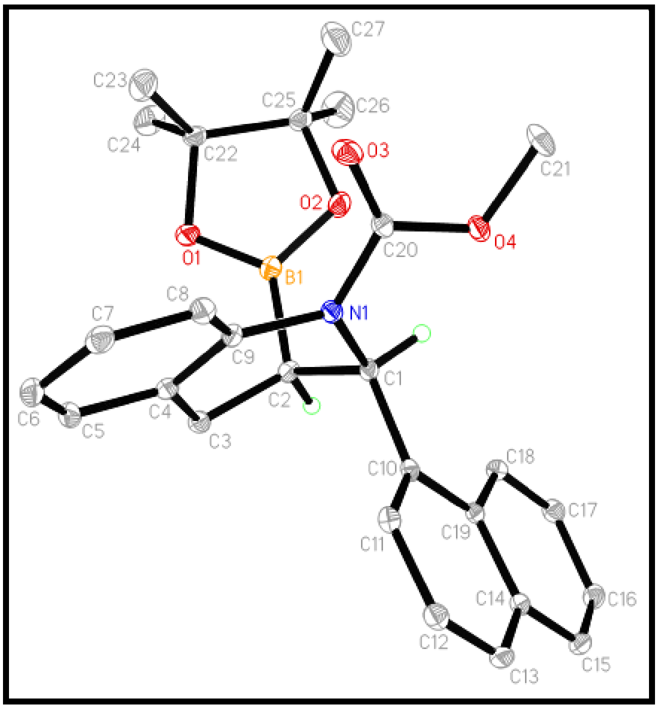

Figure S2. Structure of compound **2a**.

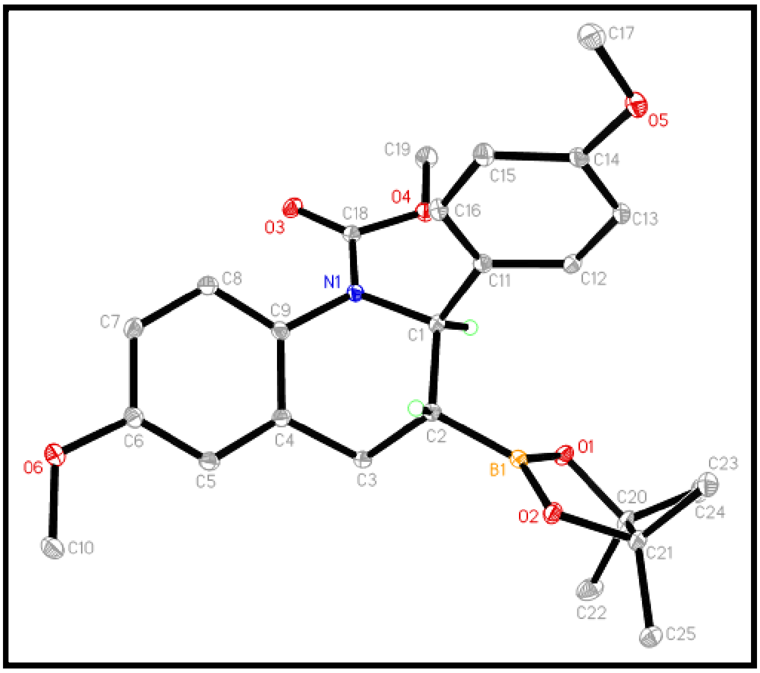

Figure S3. Structure of compound **2p**.
